# Design, Synthesis, and Development of Pyrazolo[1,5-*a*]pyrimidine Derivatives as a Novel Series of Selective PI3K*δ* Inhibitors: Part II—Benzimidazole Derivatives

**DOI:** 10.3390/ph15080927

**Published:** 2022-07-27

**Authors:** Mariola Stypik, Stanisław Michałek, Nina Orłowska, Marcin Zagozda, Maciej Dziachan, Martyna Banach, Paweł Turowski, Paweł Gunerka, Daria Zdżalik-Bielecka, Aleksandra Stańczak, Urszula Kędzierska, Krzysztof Mulewski, Damian Smuga, Wioleta Maruszak, Lidia Gurba-Bryśkiewicz, Arkadiusz Leniak, Wojciech Pietruś, Zbigniew Ochal, Mateusz Mach, Beata Zygmunt, Jerzy Pieczykolan, Krzysztof Dubiel, Maciej Wieczorek

**Affiliations:** 1 Celon Pharma S.A., ul. Marymoncka 15, 05-152 Kazun Nowy, Poland; stanislaw.michalek@celonpharma.com (S.M.); orlowska.nina@gmail.com (N.O.); marcin.zagozda@celonpharma.com (M.Z.); maciejdziachan@gmail.com (M.D.); martyna.banach@celonpharma.com (M.B.); tupaw@wp.pl (P.T.); pgunerka@gmail.com (P.G.); dzdzalik@iimcb.gov.pl (D.Z.-B.); apstanczak@gmail.com (A.S.); ulak54@gmail.com (U.K.); kmulewski91@gmail.com (K.M.); damian.smuga@celonpharma.com (D.S.); wioleta.maruszak@celonpharma.com (W.M.); lidia.gurba@celonpharma.com (L.G.-B.); arkadiusz.leniak@celonpharma.com (A.L.); wojciech.pietrus@celonphamra.com (W.P.); mateusz.mach@celonpharma.com (M.M.); beata.zygmunt@celonpharma.com (B.Z.); jerzy.pieczykolan@celonpharma.com (J.P.); krzysztof.dubiel@celonpharma.com (K.D.); maciej.wieczorek@celonpharma.com (M.W.); 2 Faculty of Chemistry, Warsaw University of Technology, ul. Noakowskiego 3, 00-664 Warsaw, Poland; zbigniew.ochal@pw.edu.pl

**Keywords:** PI3K*δ* inhibitors, anti-inflammatory therapy, 5-benzimidazole-pyrazolo[1,5-*a*]pyrimidine, CPL302415

## Abstract

Phosphoinositide 3-kinase (PI3K) is the family of lipid kinases participating in vital cellular processes such as cell proliferation, growth, migration, or cytokines production. Due to the high expression of these proteins in many human cells and their involvement in metabolism regulation, normal embryogenesis, or maintaining glucose homeostasis, the inhibition of PI3K (especially the first class which contains four subunits: *α*, *β*, *γ*, *δ*) is considered to be a promising therapeutic strategy for the treatment of inflammatory and autoimmune diseases such as systemic lupus erythematosus (SLE) or multiple sclerosis. In this work, we synthesized a library of benzimidazole derivatives of pyrazolo[1,5-*a*]pyrimidine representing a collection of new, potent, active, and selective inhibitors of PI3K*δ*, displaying IC_50_ values ranging from 1.892 to 0.018 μM. Among all compounds obtained, CPL302415 (**6**) showed the highest activity (IC_50_ value of 18 nM for PI3K*δ*), good selectivity (for PI3K*δ* relative to other PI3K isoforms: PI3K*α*/*δ* = 79; PI3K*β*/*δ* = 1415; PI3K*γ*/*δ* = 939), and promising physicochemical properties. As a lead compound synthesized on a relatively large scale, this structure is considered a potential future candidate for clinical trials in SLE treatment.

## 1. Introduction

Phosphoinositide 3-kinase delta (PI3K*δ*), the lipid kinase, is a member of the family of PI3K enzymes divided into three classes: I (PI3K*α*, PI3K*β*, PI3K*γ*, PI3K*δ)*, II, and III. Due to their involvement in catalyzing the phosphorylation of phosphatidylinositol-4,5-diphosphate (PIP2) to phosphatidylinositol-3,4,5-triphosphate (PIP3), PI3Ks start a cascade of downstream activities to induce various types of biological processes such as cell growth, survival, proliferation, or differentiation [1,2,3,4,5,6]. All class I PI3K isoforms occur as a heterodimer of one regulatory subunit (p85) with the corresponding catalytic subunit (p110*α*, p110*β*, and p110*δ*). The p110 subunits of PI3K isoforms have been thoroughly characterized [7]. The ATP-binding site of p110*δ* in PI3K*δ* comprises a few functionalities such as a hinge pocket, an affinity pocket, and a hydrophobic region, which lies below a non-conserved rim of the active site. Interaction with the large and flat hydrophobic face of a conserved tyrosine residue (Tyr-876) was reported for most PI3K inhibitors. Moreover, many of them also have additional hydrophobic interactions with the affinity pocket for the enzyme, where they can form hydrogen bonds with Lys-833 or other hydrophilic residues caused by the presence of adequate heteroatom [8,9]. Most selective PI3K*δ* inhibitors exhibit interaction between crucial amino acids (Trp-760 and Met-752) while entering the active pocket [9,10,11]. Interaction with the tryptophan shelf (Trp-760) impacts the PI3K*δ* selectivity. Steric blockage in the tryptophan region leads to selectivity for PI3K*δ* because of the disfavored binding to other PI3K isoforms. It has been proven that these structural determinants are crucial in the activity and selectivity of PI3K*δ* and thus are used for designing PI3K*δ* inhibitors [5,8,10,11].

In this study, we designed, developed, and described a family of PI3K*δ* inhibitor structures, based on the pyrazolo[1,5-*a*]pyrimidine core with different modifications, which can occupy the affinity pocket of the enzyme.

The basis for inflammatory and autoimmune diseases, such as systemic lupus erythematosus (SLE) or rheumatoid arthritis (RA), is dysregulation, including overactivity of the immune system [8]. These alterations are typically progressive and cause much burdensomeness for the patients. The overproduction of autoantibodies manifests in SLE due to uncontrolled cellular action in which T lymphocytes and B lymphocytes play a crucial role [4,12,13,14,15,16,17]. The activity of PI3K*δ* in T cells of SLE diagnosed patients is risen by approximately 70% [15]. Due to the engagement of the p110*δ* subunit of the PI3K*δ* in human Th17 cells for the production of IL-17, this PI3K subfamily can be viewed as a promising molecular target for future therapies, including SLE [18,19,20,21]. Moreover, a well-recognized mechanism of the PI3K*δ* interaction at the molecular level can be efficiently utilized for the rational design, synthesis, and development of new anti-inflammatory drugs [18,19,20,22].

**Figure 1:**
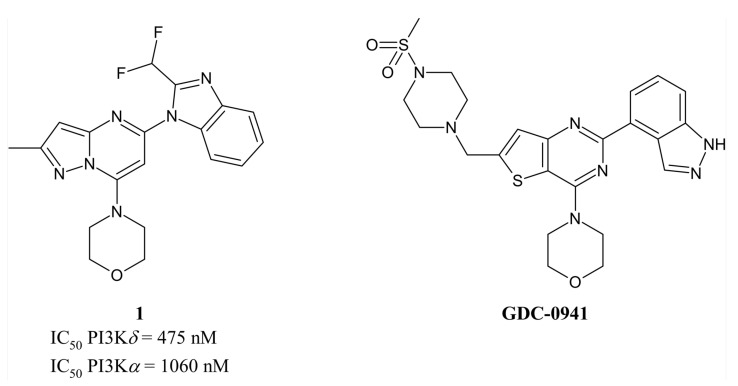
Structure of the PI3Kδ active inhibitor and GDC-0941.

Selective PI3K*δ* inhibitors can be obtained by appropriate modifications of a heterocycle that occupies the affinity pocket of the enzyme [5,10,23]. Despite the formation of hydrogen bonds between the indazole group of well-known inhibitor GDC-0941 (Figure 1) [24] and two amino acids Asp-787 and Tyr-813, the PI3K*γ*/*δ* selectivity was poor [10]. It was reported that changing the indazole group of GDC-0941 for 2-methylbenzimidazole group helped to obtain a more selective inhibitor of PI3K*δ* (PI3K*γ*/*δ* = 29) which demonstrated good potency in cellular assays [5,10]. Moreover, it was shown that optimization of interactions with Trp-760 helped to improve the selectivity of PI3K*δ* inhibitors as candidates for further development with good pharmacokinetic properties [10,11,25,26]. The new compounds with different substituents at the benzimidazole ring’s C(2) position were obtained and described [10]. It was reported that the inclusion of large, bulky groups at the benzimidazole’s C(2) position could reduce the inhibition of PI3K*δ* [10]. These structure–activity relationships highlight the crucial role of the amine and benzimidazole subunit in determining PI3K*δ* activity and selectivity for an obtained series of compounds.

Many bicyclic cores-based compounds were reported as effective and active PI3K inhibitors. Most of them, including thienopyrimidines [23,27] or pyridopyrimidines [10,23,24], were described as pan-PI3K inhibitors. Due to the problems with time-dependent CYP inhibition [5,23], selectivity, bioavailability, or solubility [23,28], other bicyclic cores such as isoxazolopyrimidines [23,29], imidazopyrimidines [23,30], or pyrazolopyrimidines [23,31] have also been designed, synthesized, and reported. A large number of PI3K inhibitors showed the potential of applying morpholine moiety as a *H*-bond acceptor in the hinge-binding motif [23,24]. In our docking studies in the previous paper [25], the crucial role of binding between the morpholine system and Val-828 was observed. Since 2012, many morpholine-based inhibitors of the PI3K kinase have been published [23]. Moreover, in 2012, a group of 2-(difluoromethyl)-1*H*-benzimidazole derivatives enriched a library of known PI3K inhibitors [23,32,33,34]. These structures are based on the 1,3,5-triazine monocyclic core and a morpholine ring in the hinge region. Evaluation of mono-, bi-, or higher-cyclic cores with a different arrangement of the substituents allowed for more active and selective compounds [10,23,28].

In our work, we focused on the pyrazolo[1,5-*a*]pyrimidine core with various amine substituents in position C(2) and different benzimidazole groups in the C(5) position at the core region. It was reported that pyrazolo[1,5-*a*]pyrimidines are promising medical pharmacophores in structures as potential drugs in the treatment of cancer, as well as inflammatory or viral diseases [35,36]. Our previous study [25] described the development of pyrazolo[1,5-*a*] pyrimidine derivatives with different substituents (heteroaromatic systems) at position 5 of the mentioned core. It was reported that 5-indole-pyrazolo [1,5-*a*] pyrimidines as inhibitors of PI3K*δ* were the most selective structures of the obtained series. On the other hand, we identified a 2-difluoromethylbenzimidazole derivative **1** as the most active compound (Figure 1). It was identified as a moderate PI3K*δ* inhibitor (IC_50_ = 475 nM) with poor selectivity toward the alpha isoform. We reported that modifications of a mentioned core with many different substituents could contribute to inhibitors’ activity and selectivity enhancement. In this work, we synthesized and described more than thirty new, active, and potent selective PI3K*δ* inhibitors in the extended structure–active relationship (SAR) study, keeping the scaffold of **1** as a starting point.

## 2. Results and Discussion

### 2.1. Chemistry

#### 2.1.1. Synthesis of Compounds **5**–**9** and **11**–**12**

**Scheme 1:**
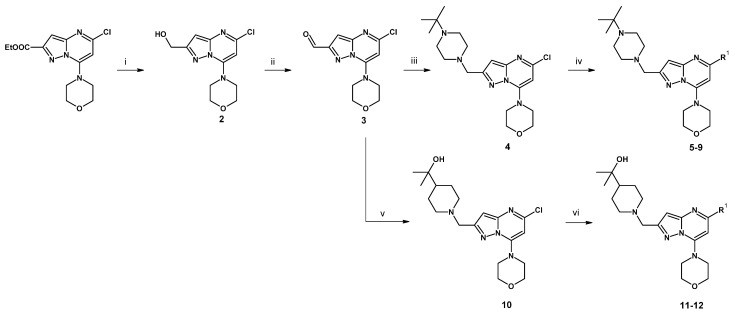
Synthesis of benzimidazole derivatives. Reagents and conditions: (**i**) CaCl_2_, NaBH_4_, EtOH, reflux, 3 h, 99%; (**ii**) Dess–Martin periodinane, DMF, RT, 2 h, 46%; (**iii**) 1-*tert*-butylipiperazine, sodium triacetoxyborohydride, DCM, RT, 18 h, 84%; (**iv**) benzimidazole derivative, tris(dibenzylideneacetone)dipalladium(0), 9,9-dimethyl-4,5-bis(diphenylphosphino)xanthene, Cs_2_CO_3_, toluene, 150 °C, 6 h, 200 W, MW, 4–93%; (**v**) 2-(4-piperidyl)-2-propanol, sodium triacetoxyborohydride, DCM, RT, 63%; (**vi**) benzimidazole derivative, tris(dibenzylideneacetone)dipalladium(0), 9,9-Dimethyl-4,5-bis(diphenylphosphino)xanthene, Cs_2_CO_3_, toluene, 150 °C, 6 h, 200 W, MW, 52–66%.

Our research shows that modifications of benzimidazole groups and amine subunits play a crucial role in the activity and selectivity of PI3K*δ* inhibitors. During the SAR exploration and docking calculations, we found two amine subunits at the C(2) position of the pyrazolo[1,5-*a*]pyrimidine core, *N*-*tert*-butylpiperazin-1-ylmethyl and 2-(4-piperidin-1-ylmethyl)-2-propanol, have the most promising potency in PI3K*δ* inhibition. Due to the observed high activity of the mentioned families of compounds against PI3K*δ* and unexplored chemical space, new benzimidazole derivatives were synthesized according to Scheme 1.

Starting from ethyl 5-chloro-7-(morpholin-4-yl)pyrazolo[1,5-*a*]pyrimidine-2-carboxylate, structures **5**–**9** and **11**–**12** were obtained after four-step synthesis. In the first step, alcohol **2** was synthesized by the ester group reduction with sodium borohydride and an almost quantitative yield (99%, Scheme 1). Next, alcohol **2** was oxidized into the corresponding aldehyde **3** using Dess–Martin periodinane (46% yield). Subsequently, the amine subunits derivatives **4** and **10** were obtained in reductive amination reactions by engaging appropriate amine in the presence of sodium triacetoxyborohydride (good, 84%, and 63% yields, respectively). In the last step, the received substituted 5-chloro-pyrazolo[1,5-*a*]pyrimidines **4** and **10** were transferred into the final structures by utilizing the Buchwald–Hartwig reaction conditions with corresponding benzimidazoles. This palladium-catalyzed reaction, conducted under microwave irradiation, gave compounds **5**–**9** and **11**–**12** (un-optimized yields in the range of 34–93%).

#### 2.1.2. Synthesis of Compounds **16**–**38** and **40**–**54**

**Scheme 2:**
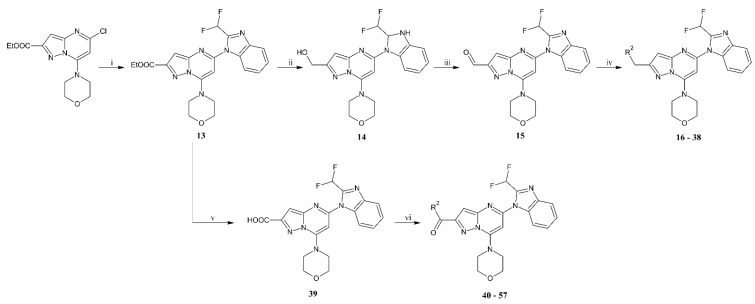
Synthesis of 5-(2-difluoromethylobenzimidazo-1-yl)pyrazolo[1,5-*a*]pyrimidine derivatives. Reagents and conditions: (**i**) 2-(difluoromethyl)-1*H*-benzimidazole, TEACl, K_2_CO_3_, DMA, 160 °C, 3 h, 89%; (**ii**) LiAlH_4_, THF, 0 °C, 3 h, 89%, (**iii**) Dess–Martin periodinane, DMF, RT, 1 h, 78% or MnO_2_, toluene:buthyl acetate, reflux 1.5 h, 68%; (**iv**) amine, sodium triacetoxyborohydride, DCM, 18 h, 38–93%; (**v**) LiOH, MeOH, H_2_O, 98%; (**vi**) amine, HATU, TEA, RT, 2 h, 33–81%.

Benzimidazole derivatives were prepared in a multistep synthesis that branched into two pathways depending on the group selected in the core C(2) position (Scheme 2).

Structures **16**–**38** and **40**–**57** were synthesized according to the general pathway depicted in Scheme 2. Compounds **16**–**38** were obtained after four-step synthesis from 5-chloro-7-(morpholin-4-yl)pyrazolo[1,5-*a*]pyrimidine-2-carboxylate (commercially available) which was coupled with 2-(difluoromethyl)-1*H*-benzimidazole in the presence of tetraethylammonium chloride and potassium carbonate to provide ethyl 5-[2-(difluoromethyl)-1*H*-1,3-benzimidazol-1-yl]-7-(morpholin-4-yl)pyrazolo[1,5-*a*]pyrimidine-2-carboxylate (**13**) as a crucial intermediate (89% yield). Then, the resulting product **13** was reduced to alcohol **14** under treatment with a lithium aluminum hydride solution (89% yield). We observed that the double bond within the imidazole ring of benzimidazole substituent was reduced concomitantly with the ester group. Interestingly, the oxidation of alcohol **14** to aldehyde **15** with Dess–Martin periodinane (or with activated manganese(IV) oxide) was accompanied by full restoration of aromaticity within the benzimidazole heterocycle. Final structures **16**–**38** were obtained by reductive amination reactions (Scheme 2) with different amines such as *N*-*tert*-butylpiperazine, morpholine, or 4-methylpiperidin-4-ol (see tables below for details) with un-optimized yields (38–93%). Based on our previous SAR studies, we observed that the carbonyl group in position C(2) of pyrazolo[1,5-*a*]pyrimidine derivatives may enhance the activity of the obtained structures. While keeping this in mind, further research focused on the replacement of the (-CH_2_) group with a (-CO) group leading to structures **40**–**57** in two steps from intermediate **13**. Unlike for compounds **16**–**38** synthesis, the ester group of **13** was hydrolyzed with the lithium hydroxide to 5-[2-(difluoromethyl)-1*H*-benzimidazol-1-yl]-7-(morpholin-4-yl)pyrazolo[1,5-*a*]pyrimidine-2-carboxylic acid (**39**, 98% yield). Subsequently, carboxyl derivatives were converted into a series of final amides **40**–**57** (see tables below to trace the selection of substituents) using 2-(7-aza-1*H*-benzotriazole-1-yl)-1,1,3,3-tetramethyluronium hexafluorophosphate (HATU) and triethylamine as a base (un-optimized 33–81% yields).

### 2.2. Docking Study

Crystal structure analysis, combined with data from biochemical and cellular assays, has been used to understand the molecular basis of observed inhibitors’ activities and selectivities. Utilizing the Auto-Dock Vina program for docking studies [35], we wished to investigate the binding mode of our compounds with the PI3K*δ* isoform. Based on the available crystallographic structures of PI3K (for example PDB ID: 2WXL) and reference papers regarding in silico calculations [5,8,10,23,24], we gained valuable information about protein–ligand interactions in the active site and chose to focus on the pyrazolo[1,5-*a*]pyrimidine core.

Over the course of our computer-assisted studies, we found that the morpholine ring at position 7 of pyrazolo[1,5-*a*]pyrimidine core is required for interaction with PI3K at the catalytic site. More specifically, the most crucial interaction is the critical hydrogen bond between the oxygen atom of the morpholine group and Val-828 in the hinge region of the enzyme. In our previous work [25], we observed an existing hydrogen bond between the C(5)-indole pyrazolo[1,5-*a*]pyrimidine derivatives and the Asp-787. However, benzimidazole derivatives, presented in this work, lack that interaction when targeted toward this region. Instead, we observed the possibility of hydrogen bond formation between the nitrogen atom at the third position of the benzimidazole ring system and Lys-779.

**Figure 2:**
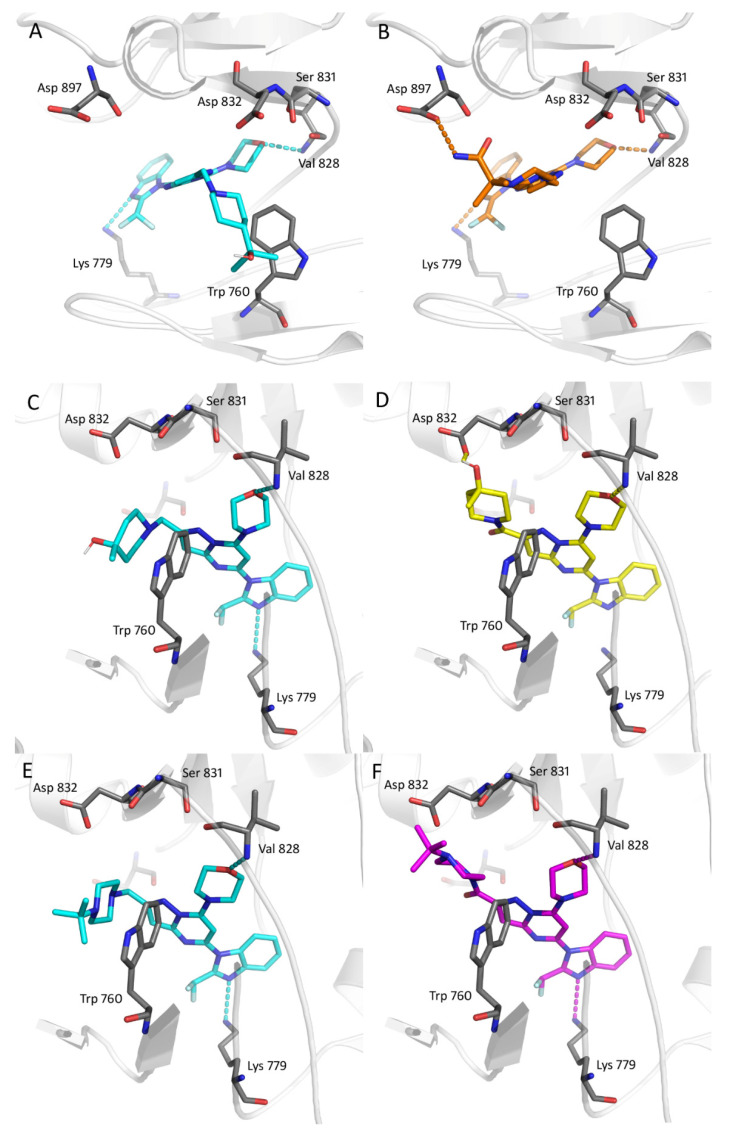
The most important interactions in the PI3K binding site for selected structures. An example of 3D modeling possible interaction found for selected compounds (PDB ID:2WXL): (**A**)—compound **11**; (**B**)—compound **17**; (**C**)—compound **30**; (**D**)—compound **49**; (**E**)—compound **6**; (**F**)—compound **40**. No protons were added, but the appropriate state of protonation was maintained.

In addition, several regions have been identified in the active site of the enzyme that have a profound impact on the activity and selectivity toward PI3K*δ.* Due to critical structural determinants, depending on the substituent type (R^1^, R^2^, Scheme 1 and Scheme 2, respectively), we observed different interactions of our structures with the tryptophan shelf (Trp-760) and selected amino acids within the active pocket. For example, 2-hydroxypropyl residue of compound **11** keeps close proximity to Trp-760 (the tryptophan shelf interaction, Figure 2A) by locating the hydroxyl group conformationally away from the amino acid. On the other hand, the piperazine fragment of compound **17** (Figure 2B) takes the most distant position from the tryptophan shelf, supported by the polar amide group bond with aspartic acid (Asp-897). Moreover, among some structures showing no Trp-760 interaction, due to different types of the amine substituents, a shift towards other amino acids, e.g., Ser-831, was observed.

Compounds containing a donor fragment, such as hydroxyl, amine, or the amide group near the piperazine or piperidine ring (such as **11**, **35**, or **36**), are found to have higher IC_50_ values and therefore lower potential for activity due to the poor interaction between the aliphatic fragment and the tryptophan shelf (Trp-760). A similar situation is observed for structure **30** (Figure 2C), in which the aliphatic component targets the Trp-760 indole ring, but the hydroxyl group is too far to form a hydrogen bond with the polar amino acids located at the opposite side of the enzyme pocket.

A shift beyond the tryptophan region of the piperidine ring was also observed for compound **49**, with the carboxyl group introduced in place of the methylene group. Such arrangement is additionally supported by the formation of a hydrogen bond between the hydroxyl group of the 4-hydroxy-4-methylpiperidinyl subunit and aspartic acid (Asp-832, Figure 2D).

In connection with the described dependencies, our research suggests that due to the shift of the amine ring relative to the Trp-760 and the formation of a hydrogen bonding with the aforementioned Asp-832 and Asp-897, compounds with a carboxyl group in the C(2) position of the pyrazolo[1,5-*a*]pyrimidine (linking the amine group) are more preferred in terms of kinase activity and selectivity than compounds with a methylene group at the same position.

Compound **6** (Figure 2E), containing the *tert*-butyl piperazine ring, gave different outcomes in our docking studies. Interaction between that aliphatic fragment and Trp-760 translates into the properties of this compound, such as potency, activity, and selectivity towards PI3K*δ*. Moreover, in this structure, we observed the characteristic bond between the oxygen atom located at the morpholine ring (playing the role of an *H*-bond acceptor in the hinge-binding motif) and Val-828. Interaction of the benzimidazole residue nitrogen atom and Lys-779 has also been recognized.

A mix of conformational interactions was assigned to compound **40** (Figure 2F), which binds to Val-828 and Lys-779, including compound **6**. However, the replacement of the methylene bridge with the carbonyl function was associated with the loss of Trp-760 interaction and the simultaneous loss of biological activity.

As a result of all relationships described, compound **6** turned out to be the most active and promising structure of the entire library obtained.

### 2.3. Biological Evaluation

#### In Vitro PI3 Kinase Inhibition Assays

All compounds were tested in a biochemical assay that measured the inhibition of phosphatidylinositol (4,5)-bisphosphate (PIP2) production by PI3K isoforms using the ADP-Glo kinase assay (Promega). In addition, the effects of synthesized compounds on B cell proliferation were measured.

All newly synthesized compounds proved to be active PI3K*δ* inhibitors (IC_50_ = 1.892–0.018 μM) and, additionally, eleven of obtained structures turned out to be highly active, reaching the value of IC_50_ below 100 nM. For this reason, PI3K*δ* final inhibitors, which are very potent with drug-like physical properties, are considered drug candidates in SLE and other autoimmune and inflammatory diseases.

Two positions of the pyrazolo[1,5-*a*]pyrimidine core: C(2) (R^1^) and C(5) (R^2^) were optimized and described in this paper. Regarding the first C(2) optimization, many benzimidazole derivatives (for two chosen amine subunits: piperazines and piperidines) were designed and synthesized (Table 1). It was observed that within the nano- and micro-molar IC_50_ value range, the potency of obtained inhibitors is different despite the substituent size at the C(2) position, for example in pairs **5** and **8** or **6** and **9**. On the other hand, a selected pair of examples (compounds 11 and 12) indicates that the selectivity against PI3Kδ activity is relatively insensitive to the steric bulkiness of the substituent placed at the C(5) core’s position. Of the whole series of PI3K*δ* inhibitors obtained, compounds **6** and **11** turned out to be the most potent, with IC_50_ values of 18 and 52 nM, respectively. Moreover, structure **6** shows the best selectivity towards other PI3K isoforms among all the compounds tested. For the above reasons, 2-(difluoromethyl)-1*H*-benzimidazole was selected as the most optimal and promising R^1^ substituent in pyrazolo[1,5-*a*]pyrimidine ring. The next step of our studies was the expansion of the compound library with the determination of R^2^ groups keeping the constant 2-(difluoromethyl)-1*H*-benzimidazole R^1^ substituent at C(5) position.

The R^2^ substituent has been optimized using different substituents which aim to simultaneously increase selectivity and activity. Optimization focused on modifying the amine subunit; thus, piperazines, piperidines, five-member rings, bulky amine groups, and other available amines were used (Table 2). Among all modifications, we found the piperazine and piperidine derivatives as the most promising. Compounds containing an amine group with a five-membered ring showed the IC_50_ values in the 1892–1896 nM range; however, their PI3K*γ*/*δ* selectivities remained lower than for the other amine groups with a six-member ring in their structure (Table 2). This observation suggests that replacing the five- with a six-membered ring is more favorable for PI3K*δ* inhibition. Moreover, heterocycles based on the six-membered ring as R^2^, with a nitrogen atom in the 1- or 1- and 4-position(s), are generally more active and selective. As an excellent example, this thesis can serve compound **27**, with the nitrogen atom shifted outside the six-membered ring. In this individual case, a significant potency drop against PI3K*δ* below 1000 nM threshold was noted (Table 2). Modifications of substituted amine heterocycles are significant because they directly affect the PI3K*δ* enzyme’s affinity pocket interactions. Our preliminary research suggested that the presence of the 2-(4-piperidyl)-2-propanol hydroxylic group could play a crucial role in gaining the inhibition of PI3K*δ*. We believed, based on in silico studies, that the bond formed between the hydroxyl group and amine group of Ser-831 within the selectivity pocket of the enzyme should increase both the activity and selectivity. Therefore, a set of compounds containing proton donor groups was synthesized. Unfortunately, this rationale failed, as it can be clearly seen while searching the IC_50_ values of compounds: **29**–**31** and **34**–**36** (Table 2). The observed loss of potency could be explained by the conformational freedom provided by the methylene linkage, allowing the escape from the tryptophan shelf position and simultaneous polar interactions with side amino acids within the active pocket. To confront this possibility, the methylene bridge was converted into carbonyl function, leading to conformationally restrained cyclic amide derivatives which lack possible conformational changes in the C(2) position of pyrazolo[1,5-*a*]pyrimidine.

For that reason, a library of compounds with multiple amine substituents was designed and synthesized (Table 3). Within this set, the lowest IC_50_ values were observed for examples **40**, **42**, **43**, and **55**, (measured at 84, 74, 63, and 82 nM, respectively). These structures also had good PI3K*γ/δ* selectivity.

The consequence of the methylene bridge to carbonyl group interchange can be tracked separately in groups of compounds divided into those with or without hydrogen bond donor (HBD) capabilities within the R^2^ substituent.

**Scheme 3:**
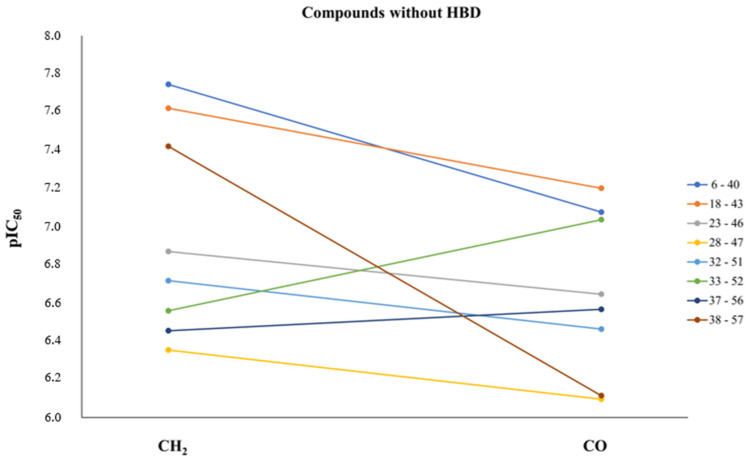
The correlation of the potencies given for the compounds lacking HBD interaction within the heterocyclic system.

The compounds lacking the HBD functionally tend to have better potency when the methylene bridge at the C(2) core position is present within a stable substituent environment. Although some exceptions have been found, including the pairs **33** and **52** or **37** and **56**, for all other pairs, such as **6** and **40** or **38** and **57**, the PI3K*δ* activity drops when the methylene bridge is replaced with its carbonyl structural equivalent (Scheme 3). The measured potency is positively correlated with the increasing spherical lipophilic volume present at position 4 in the six-membered heterocyclic ring of the amino substituent. As an example, compounds **37**, **38** (Table 2), and **6** (Table 1), bearing methyl, *iso*-propyl, and *tert*-butyl motif, can be named, for which the IC_50_ value at concentrations of 351, 38, and 18 nM was measured, respectively.

**Scheme 4:**
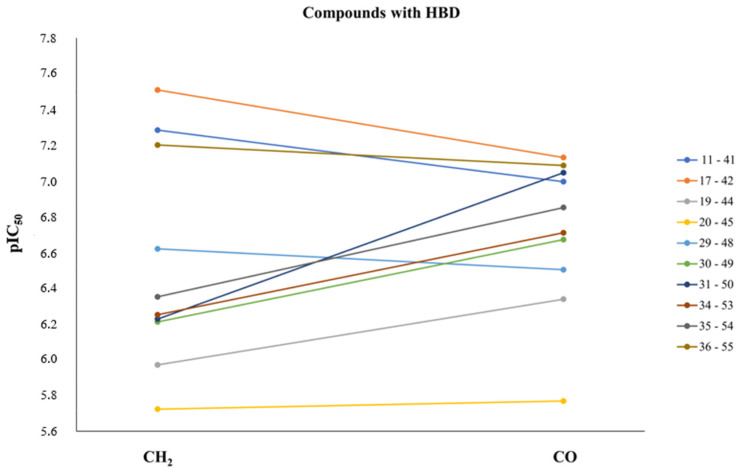
The correlation of the potencies given for the compounds lacking HBD interaction within the heterocyclic system.

The activity dependence on the relationship between the methylene bridge and carbonyl interchange is not so clear for the compounds with structural capabilities of HBD interaction within the R^2^ substituent. Since it is challenging to isolate the bulkiness of the substituent alone and the accompanying HBD interplay with the surrounding polar environment, both those elements might affect the IC_50_ value. As seen in Scheme 4, there is only a slight prevalence of increased activity toward the carbonyl (amide) functionality. Therefore, both structural motifs (the methylene bridge and the carbonyl function) should be considered equally essential modifications for SAR exploration.

A detailed analysis of the entire library of synthesized compounds led to the selection of five the most promising structures: **6**, **11**, **16**, **17**, and **18** (Table 4). All of them turned out to be the derivatives of 2-(difluoromethyl) -1*H*-benzimidazole at the C(5) position of pyrazolo[1,5-*a*] pyrimidine core bearing amines of the six-membered ring as the R^2^ can substitute a methylene bridge (CH_2_) as a linkage (Table 4). Their IC_50_ values against PI3K*δ* were found in the nanomolar range (18–52 nM), good selectivity in relation to other PI3K isoforms, and preserved CD19 cellular activity (for details see Table 4).

Besides the best enzymatic and cellular activity, compound **6** was chosen for further development based on acceptable solubility, microsomal stability, permeability, and the plasma protein binding range (for details see Table 5). Attempts to scale up the synthesis turned out to be chemically and economically viable. As a result, the lead compound **6** (CPL302415) was obtained in the amount exceeding one kilogram and purity suitable for future toxicological studies.

Due to the prediction of metabolism, the calculation of in vitro clearance, or the identification of the correlation of metabolites with hepatic stability in the ADMET studies [37,38], many important parameters were determined for our lead compound CPL302415 (Table 5). The metabolic stability was evaluated by measuring the intrinsic clearance and t_1/2_ in mice and human liver microsomes (MLM and HLM), which were reported as very promising for CPL302415 (details in Table 5). Moreover, this structure has good solubility, permeability, and optimistic plasma protein binding parameters ((PPBs) in the range of 79–83% depending on the species; Table 5). Additionally, CPL302415 (**6**) was checked for bioavailability in mice (F > 55%) and dogs (F > 90%), depending on the formulation form. All these parameters make CPL302415 ready for toxicological studies and, hopefully, for future clinical trials.

## 3. Materials and Methods

### 3.1. Chemistry

#### 3.1.1. General Information

Chemicals (at least 95% purity) were purchased from ABCR (Dallas, TX, USA), Acros (Geel, Belgium), Alfa Aesar (Haverhill, MA, USA), Combi-Blocks (San Diego, CA, USA), Fluorochem (Hadfield, UK), (Buchs, Switzerland), Merck (Darmstadt, Germany), and Sigma Aldrich (Saint Louis, MI, USA), and were used without additional purification. Solvents were purified according to standard procedures if required. Air- or moisture-sensitive reactions were carried out under an argon atmosphere. All reaction progresses were routinely checked by thin-layer chromatography (TLC). TLC was performed using silica-gel-coated plates (Kieselgel F254) and visualized using UV light. Flash chromatography was performed using Merck silica gel 60 (230–400 mesh ASTM). ^1^H NMR spectra were acquired using a Varian Inova 300 MHz NMR spectrometer, a JOEL JNMR-ECZS 400 MHz spectrometer, a JOEL JNMR-ECZR 600 MHz spectrometer, and a Bruker DRX 500 NMR spectrometer with ^1^H being observed at 300 MHz, 400 MHz, 600 MHz, and 500 MHz, respectively. ^13^C NMR spectra were recorded similarly at 75 MHz, 101 MHz, 151 MHz, and 126 MHz frequencies for ^13^C, respectively. Due to the poor solubility of some final compounds, usual characterization was omitted using ^13^C NMR. Chemical shifts for ^1^H and ^13^C NMR spectra were reported in *δ* (ppm) using tetramethylsilane as an internal standard or according to the residual undeuterated solvent signal (2.50 ppm for DMSO-*d_6_* and 7.26 ppm for CDCl_3_). The abbreviations for spin interaction coupled ^1^H signals are as follows: s (singlet), d (doublet), t (triplet), m (multiplet), dd (doublet of doublets), dt (doublet of triplet), and q (quartet). Coupling constants (J) are expressed in Hertz. The ^13^C NMR spectrum was recorded with the use of the JEOL Royal HFX probehead that allows measurements to be taken with the simultaneous decoupling of both ^1^H and ^19^F nuclei [39]. Mass spectra (atmospheric pressure ionization electrospray (API-ES) and electrospray ionization (ESI-MS)) were obtained using the Agilent 6130 LC/MSD spectrometer or Agilent 1290 UHPLC coupled with the Agilent QTOF 6545 mass spectrometer. All spectra of final compounds are in Supplementary Materials.

#### 3.1.2. Synthesis

##### Procedure for 5-chloro-7-(morpholin-4-yl)pyrazolo[1,5-a]pyrimidin-2-yl]methanol (**2**)

Calcium chloride (10.4 g, 93.6 mmol) and sodium borohydride (7.56 g, 190 mmol) were added to the suspension of ethyl 5-chloro-7-(morpholin-4-yl)pyrazolo[1,5-*a*]pyrimidine-2-carboxylate (10.0 g, 31.2 mmol) in EtOH (150 mL). The mixture was stirred and heated to reflux for 3 h. Then, the reaction was cooled to RT and quenched with NH_4_Cl_aq_ (150 mL) and 1 M HCl (150 mL). The aqueous phase was extracted three times with AcOEt. The combined extracts were washed with water and dried over Na_2_SO_4_, filtered, and concentrated to give **2** as a white solid (8.36 g, 31.1 mmol) with a 99% yield. ^1^H NMR (600 MHz, DMSO-*d_6_*) *δ* 6.43 (s, 1H, Ar-H), 6.35 (s, 1H, Ar-H), 5.32 (t, *J* = 5.9 Hz, 1H, -OH), 4.59 (dd, *J* = 5.9, 0.3 Hz, 2H, CH_2_), 3.82–3.80 (m, 4H, morph.), 3.78–3.77 (m, 4H, morph.).

##### Procedure for 5-chloro-7-(morpholin-4-yl)pyrazolo[1,5-a]pyrimidine-2-carbaldehyde (**3**)

To a solution of compound **2** (3.00 g, 10.9 mmol) in DMF (30.0 mL) in argon atmosphere, Dess–Martin periodinane (97%, 5.74 g, 13.1 mmol) was added. The resulting mixture was stirred at room temperature for 2 h. The solvent was evaporated. The residue was washed with AcOEt and filtered. The filtrate was concentrated and the crude product was purified by flash chromatography (0–100% AcOEt gradient in heptane) to give **3** (1.34 g, 5.02 mmol) with a 46% yield. ^1^H NMR (500 MHz, DMSO-*d_6_*) *δ* 10.09 (s, 1H, -CHO), 6.97 (s, 1H, Ar-H), 6.63 (s, 1H, Ar-H), 3.90–3.85 (m, 4H, morph.), 3.86–3.78 (m, 4H, morph.).

##### General Procedure for the Reductive Amination Reaction

Amine derivative (1.2 eq) was added to the solution of the corresponding aldehyde (1.0 eq) in dry DCM (10 mL/1 g corresponding aldehyde) and then stirred at room temperature. After 1 h, sodium triacetoxyborohydride (1.5 eq) was added and the mixture was stirred at room temperature for a further 15 h. Water was added to the reaction mixture and phases were separated. The aqueous phase was extracted three times with DCM. Combined organic phases were dried over anhydrous sodium sulfate, filtered, and concentrated. The residue was purified by flash chromatography.

4-{2-[(4-*tert*-butylpiperazin-1-yl)methyl]-5-chloropyrazolo[1,5-*a*]pyrimidin-7-yl}morpholine (**4**)

Compound **4** was prepared from aldehyde **3** (1.70 g, 2.15 mmol), 1-*tert*-butylpiperazine (0.36 g, 2.58 mmol), and DCM (17.0 mL) with sodium triacetoxyborohydride (0.68 g, 3.22 mmol), according to the general procedure for the reductive amination reaction. The crude product was purified by flash chromatography (0–10% MeOH gradient in AcOEt) to give **4** (1.42 g, 3.61 mmol) as a white solid with a 84% yield. ^1^H NMR (600 MHz, DMSO-*d_6_*) *δ* 6.38 (s, 1H, Ar-H), 6.36 (s, 1H, Ar-H), 3.83–3.80 (m, 4H, morph.), 3.80–3.78 (m, 4H, morph.), 3.59 (s, 2H, -CH_2_), 2.50–2.46 (m, 4H, piperaz.), 2.45–2.37 (m, 4H, piperaz.), 0.98 (s, 9H, t-Bu.).

##### General Procedure for the Buchwald–Hartwig Reaction

To a pressure, microwave vessel 5-chloro-pyrazolo[1,5-*a*]pyrimidine (1.0 eq), amine (1.5 eq), tris(dibenzylideneacetone)dipalladium (0.05 eq), 9,9-dimethyl-4,5-bis(diphenylphosphino)xanthene (0.1 eq), cesium carbonate (2.0 eq), and solvent (10 mL/1 g pyrazolo[1,5-*a*]pyrimidine) were simultaneously added. The reaction vessel was then sealed and heated to 150 °C for 6 h in a microwave (power 200 W). Then, the reaction mixture was filtered through Celite^®^ and concentrated, and the crude product was purified using flash chromatography.

1-{2-[(4-*tert*-butylpiperazin-1-yl)methyl]-7-(morpholin-4-yl)pyrazolo[1,5-*a*]pyrimidin-5-yl}-2-methyl-1*H*-benzimidazole (**5**)

Compound **5** was synthesized from **4** (0.22 g, 0.56 mmol) and 2-methyl-benzimidazole (0.11 g, 0.83 mmol) as an amine, tris(dibenzylideneacetone)dipalladium (26.3 mg, 0.027 mmol), 9,9-dimethyl-4,5-bis(diphenylphosphino)xanthene (33.9 mg, 0.055 mmol), cesium carbonate (0.37 g, 1.11 mmol), and *o*-xylene (2.20 mL), according to the general procedure for the Buchwald–Hartwig reaction. The crude product was purified by flash chromatography (0–100% AcOEt gradient in heptane) to give the title compound **5** as a white solid (0.19 g, 0.38 mmol) with a 69% yield. ^1^H NMR (300 MHz, CDCl_3_) *δ* 7.79–7.70 (m, 1H, Ar-H), 7.50–7.43 (m, 1H, Ar-H), 7.35–7.21 (m, 2H, Ar-H), 6.60 (s, 1H, Ar-H), 6.17 (s, 1H,Ar-H), 4.02–3.95 (m, 4H, morph.), 3.90–3.83 (m, 4H, morph.), 3.82 (s, 2H, CH_2_), 2.76 (s, 3H, CH_3_), 2.71–2.59 (m, 8H, piperaz.), 1.08 (s, 9H, t-Bu.). ^13^C{^1^H}NMR (75 MHz, CDCl_3_) *δ* 155.1, 151.4, 151.2, 151.0, 150.2, 148.5, 142.7, 134.5, 122.9, 119.4, 110.3, 96.5, 87.6, 66.1, 56.3, 53.6, 48.4, 45.5, 25.8, 15.6. HRMS (ESI/MS): *m*/*z* calculated for C_27_H_36_N_8_O [M + H]^+^ 489.3084 found 489.3088.

1-{2-[(4-*tert*-butylpiperazin-1-yl)methyl]-7-(morpholin-4-yl)pyrazolo[1,5-*a*]pyrimidin-5-yl}-2-(difluoromethyl)-1*H*-benzimidazole (**6**)

Compound **6** was synthesized from **4** (0.27 g, 0.68 mmol), 2-(difluoromethyl)benzimidazole (0.17 g, 1.01 mmol), tris(dibenzylideneacetone)dipalladium (31.8 mg, 0.033 mmol), 9,9-dimethyl-4,5-bis(diphenylphosphino)xanthene (39.0 mg, 0.067 mmol), cesium carbonate (0.44 g, 1.35 mmol), and *o*-xylene (2.70 mL), according to the general procedure for the Buchwald–Hartwig reaction. The crude product was purified by flash chromatography (0–100% AcOEt gradient in heptane; amino-functionalized gel column) and crystallization (AcOEt) to give **6** (0.33 g, 0.63 mmol) as a white solid with a 93% yield. ^1^H NMR (600 MHz, CDCl_3_) *δ* 7.92–7.90 (m, 1H, Ar-H), 7.65–7.64 (m, 1H, Ar-H), 7.43–7.38 (m, 2H, Ar-H), 7.25 (t, *J* = 53.5 Hz, 1H, CHF_2_), 6.59 (s, 1H, Ar-H), 6.29 (s, 1H, Ar-H), 3.99–3.97 (m, 4H, morph.), 3.90–3.89 (m, 4H, morph.), 3.80 (s, 2H, CH_2_), 2.64 (s, 8H), 1.07 (s, 9H, t-Bu.). ^13^C{^1^H, ^19^F}NMR (151 MHz, CDCl_3_) *δ* 155.5, 151.3, 150.0, 147.5, 144.7, 141.8, 134.6, 125.7, 124.1, 121.5, 111.7, 109.2 (CF_2_), 96.7, 87.2, 66.2, 56.3, 53.9, 53.6, 48.5, 45.6, 25.9 (t-Bu.). HRMS (ESI/MS): *m*/*z* calculated for C_27_H_34_F_2_N_8_O [M + H]^+^ 525.2896 found 525.2904.

1-{2-[(4-*tert*-butylpiperazin-1-yl)methyl]-7-(morpholin-4-yl)pyrazolo[1,5-*a*]pyrimidin-5-yl}-2-ethyl-1*H*-benzimidazole (**7**)

Compound **7** was synthesized from **4** (0.17 g, 0.42 mmol), 2-ethyl-benzimidazole (93.0 mg, 0.64 mmol), tris(dibenzylideneacetone)dipalladium (20.0 mg, 0.021 mmol), 9,9-dimethyl-4,5-bis(diphenylphosphino)xanthene (25.8 mg, 0.042 mmol), cesium carbonate (0.28 g, 0.85 mmol), and *o*-xylene (1.7 mL), according to the general procedure for the Buchwald–Hartwig reaction. The crude product was purified by flash chromatography (0–100% AcOEt gradient in heptane; amino-functionalized gel column) to give the title compound **7** as a white solid (85.0 mg, 0.17 mmol) with a 40% yield. ^1^H NMR (300 MHz, CDCl_3_) *δ* 7.77 (dd, *J* = 6.9, 1.6 Hz, 1H, Ar-H), 7.42 (dd, *J* = 6.8, 1.5 Hz, 1H, Ar-H), 7.26 (qd, *J* = 7.3, 3.7 Hz, 2H, Ar-H), 6.57 (s, 1H, Ar-H), 6.16 (s, 1H, Ar-H), 4.00–3.94 (m, 4H, morph.), 3.87–3.79 (m, 6H), 3.10 (q, *J* = 7.5 Hz, 2H, CH_2_), 2.77 (s, 8H), 1.40 (t, *J* = 7.5 Hz, 3H, CH_3_), 1.17 (s, 9H, t-Bu.). ^13^C{^1^H}NMR (75 MHz, CDCl_3_) *δ* 156.2, 154.9, 151.2, 150.3, 148.6, 142.7, 134.6, 122.9, 119.5, 110.2, 99.8, 96.4, 87.9, 66.1, 56.0, 52.5, 48.4, 45.6, 25.4, 22.1, 11.9. HRMS (ESI/MS): *m*/*z* calculated for C_28_H_38_N_8_O [M + H]^+^ 503.3241 found 503.3242.

1-{2-[(4-*tert*-butylpiperazin-1-yl)methyl]-7-(morpholin-4-yl)pyrazolo[1,5-*a*]pyrimidin-5-yl}-2-cyclopropyl-1*H*-benzimidazole (**8**)

Compound **8** was synthesized from **4** (100 mg, 0.25 mmol), 2-cyclopropyl-benzimidazole (59.2 mg, 0.37 mmol), tris(dibenzylideneacetone)dipalladium (11.8 mg, 0.012 mmol), 9,9-dimethyl-4,5-bis(diphenylphosphino)xanthene (15.2 mg, 0.025 mmol), cesium carbonate (168 mg, 0.51 mmol), and *o*-xylene (1.0 mL), according to the general procedure for the Buchwald–Hartwig reaction. The crude product was purified by flash chromatography (0–20% MeOH gradient in AcOEt) to give the title compound **8** as a white solid (93.0 mg, 0.18 mmol) with a 72% yield. ^1^H NMR (400 MHz, CDCl_3_) *δ* 7.70–7.68 (m, 1H, Ar-H), 7.53–7.51 (m, 1H, Ar-H), 7.29–7.20 (m, 2H, Ar-H), 6.61 (s, 1H, Ar-H), 6.28 (s, 1H, Ar-H), 3.99–3.97 (m, 4H, morph.), 3.87–3.84 (m, 4H, morph.), 3.82 (s, 2H, CH_2_), 2.66 (s, 8H), 2.39–2.32 (m, 1H, CH), 1.39–1.35 (m, 2H, CH_2_), 1.12–1.06 (m, 11H). ^13^C{^1^H}NMR (101 MHz, CDCl_3_) *δ* 156.4, 155.1, 151.2, 150.4, 148.8, 142.7, 134.9, 123.0, 122.8, 119.2, 110.6, 96.6, 88.4, 66.2, 56.3, 53.7, 48.5, 45.7, 25.8 (t-Bu.), 9.8, 8.9. HRMS (ESI/MS): *m*/*z* calculated for C_29_H_38_N_8_O [M + H]^+^ 515.3241 found 515.3239.

1-{2-[(4-*tert*-butylpiperazin-1-yl)methyl]-7-(morpholin-4-yl)pyrazolo[1,5-*a*]pyrimidin-5-yl}-2-(trifluoromethyl)-1*H*-benzimidazole (**9**)

Compound **9** was synthesized from **4** (0.20 g, 0.51 mmol), 2-(trifluoromethyl)-benzimidazole (0.14 g, 0.76 mmol), tris(dibenzylideneacetone)dipalladium (24.1 mg, 0.025 mmol), 9,9-dimethyl-4,5-bis(diphenylphosphino)xanthene (29.5 mg, 0.051 mmol), cesium carbonate (0.34 g, 1.02 mmol), and toluene (2.0 mL), according to the general procedure for the Buchwald–Hartwig reaction. The crude product was purified by flash chromatography (0–100% AcOEt gradient in heptane; amino-functionalized gel column) to give the title compound **9** as a white solid (12.0 mg, 0.02 mmol) with a 4% yield. ^1^H NMR (600 MHz, CDCl_3_) *δ* 7.94–7.93 (m, 1H, Ar-H), 7.55–7.53 (m, 1H, Ar-H), 7.46–7.42 (m, 2H, Ar-H), 6.63 (s, 1H, Ar-H), 6.16 (s, 1H, Ar-H), 3.98–3.97 (m, 4H, morph.), 3.90–3.89 (m, 4H, morph.), 3.82 (s, 2H, CH_2_), 2.67 (d, *J* = 2.1 Hz, 8H), 1.13–1.06 (m, 9H, t-Bu.). ^13^C{^1^H, ^19^F}NMR (151 MHz, CDCl_3_) *δ* 151.2, 150.2, 147.0, 141.0, 139.9, 135.4, 126.3, 124.5, 121.7, 119.7, 118.0, 111.9, 97.2, 88.1, 66.2, 56.3, 53.8, 48.6, 45.7, 45.0, 29.7, 25.9, 25.8. HRMS (ESI/MS): *m*/*z* calculated for C_27_H_33_F_3_N_8_O [M + H]^+^ 543.2802 found 543.2806.

2-(1-{[5-chloro-7-(morpholin-4-yl)pyrazolo[1,5-*a*]pyrimidin-2-yl]methyl}piperidin-4-yl)propan-2-ol (**10**)

Compound **10** was prepared from **3** (3.4 g, 12.5 mmol), 2-(4-piperidyl)-2-propanol (2.24 g, 15.0 mmol) as an amine, DCM (34.0 mL), and sodium triacetoxyborohydride (4.09 g, 18.7 mmol), according to the general procedure for the reductive amination reaction. The crude product was purified by flash chromatography (0–10% MeOH gradient in AcOEt) to give **10** (3.1 g, 7.87 mmol) with a 63% yield. ^1^H NMR (300 MHz, CDCl_3_) *δ* 6.48 (s, 1H, Ar-H), 6.03 (s, 1H, Ar-H), 3.94 (dd, *J* = 5.9, 3.6 Hz, 4H), 3.78 (dd, *J* = 5.9, 3.6 Hz, 4H), 3.72 (s, 2H, CH_2_), 3.11–3.02 (m, 2H, CH_2_), 2.09–1.98 (m, 2H,CH_2_), 1.78–1.66 (m, 4H), 1.50–1.21 (m, 4H), 1.16 (s, 6H, 2xCH_3_).

2-[1-({5-[2-(difluoromethyl)-1*H*-benzimidazol-1-yl]-7-(morpholin-4-yl)pyrazolo[1,5-*a*]pyrimidin-2-yl}methyl)piperidin-4-yl]propan-2-ol (**11**)

Compound **11** was synthesized from **10** (0.50 g, 1.24 mmol), 2-(difluoromethyl)-benzimidazole (0.31 g, 1.87 mmol), tris(dibenzylideneacetone)dipalladium (58.7 mg, 0.63 mmol), 9,9-dimethyl-4,5-bis(diphenylphosphino)xanthene (75.8 mg, 0.12 mmol), cesium carbonate (0.82 g, 2.49 mmol), and toluene (5.0 mL), according to the general procedure for the Buchwald–Hartwig reaction. The crude product was purified by flash chromatography (0–15% MeOH gradient in AcOEt) and crystallization (AcOEt) to give **11** (0.43 g, 0.81 mmol) as a white solid with a 66% yield. ^1^H NMR (300 MHz, DMSO-*d_6_*) *δ* 7.85 (dd, *J =* 20.1, 7.3 Hz, 2H, Ar-H), 7.55 (s, *J =* 54.0, 1H, Ar-H), 7.51–7.40 (m, 2H), 6.66 (s, 1H, Ar-H), 6.53 (s, 1H, Ar-H), 3.94 (s, 4H, morph.), 3.84 (s, 4H, morph.), 3.65 (s, 2H, CH_2_), 2.97 (d, *J =* 10.5 Hz, 2H, CH_2_), 1.93 (d, *J =* 10.8 Hz, 1H), 1.65 (d, *J =* 11.8 Hz, 2H), 1.36–1.10 (m, 3H), 1.02 (s, 6H, 2xCH_3_). ^13^C{^1^H}NMR (75 MHz, DMSO-*d_6_*) *δ* 155.4, 150.8, 149.6, 146.9, 144.6 (t, *J =* 47.5 Hz), 141.1, 134.0, 125.4, 123.8, 120.6, 112.3, 108.5 (t, *J =* 177.7 Hz), 95.2, 87.6, 70.1, 65.5, 56.1, 53.8, 48.1, 46.8, 26.8, 26.5. HRMS (ESI/MS): *m*/*z* calculated for C_27_H_33_F_2_N_7_O_2_ [M + H]^+^ 526.2736 found 526.2741.

2-(1-{[5-(2-methyl-1*H*-1,3-benzimidazol-1-yl)-7-(morpholin-4-yl)pyrazolo[1,5-*a*]pyrimidin-2-yl]methyl}piperidin-4-yl)propan-2-ol (**12**)

Compound **12** was synthesized from **10** (0.15 g, 0.38 mmol), 2-methyl-benzimidazole (75.5 mg, 0.57 mmol), tris(dibenzylideneacetone)dipalladium (18.0 mg, 0.019 mmol), 9,9-dimethyl-4,5-bis(diphenylphosphino)xanthene (23.2 mg, 0.038 mmol), cesium carbonate (0.25 g, 0.56 mmol), and *o*-xylene (1.5 mL), according to the general procedure for the Buchwald–Hartwig reaction. The crude product was purified by flash chromatography (0–20% MeOH gradient in AcOEt) to give the title compound **12** as a white solid (97.0 mg, 0.20 mmol) with a 52% yield. ^1^H NMR (300 MHz, CDCl_3_) *δ* 7.79–7.72 (m, 1H, Ar-H), 7.52–7.45 (m, 1H, Ar-H), 7.34–7.20 (m, 2H), 6.61 (s, 1H, Ar-H), 6.18 (s, 1H, Ar-H), 4.04–3.95 (m, 4H, morph.), 3.91–3.82 (m, 4H, morph.), 3.79 (s, 2H, CH_2_), 3.18–3.09 (m, 2H), 2.77 (s, 3H, CH_3_), 2.16–2.03 (m, 2H, CH_2_), 1.82–1.71 (m, 2H, CH_2_), 1.55–1.37 (m, 2H), 1.37–1.23 (m, 1H), 1.19 (s, 6H, 2xCH_3_). ^13^C{^1^H}NMR (75 MHz, CDCl_3_) *δ* 155.2, 151.5, 151.2, 150.2, 148.5, 142.6, 134.4, 123.0, 119.3, 110.3, 96.5, 87.6, 72.3, 66.1, 56.4, 53.9, 48.4, 47.0, 29.6, 26.7, 15.5. HRMS (ESI/MS): *m*/*z* calculated for C_27_H_35_N_7_O_2_ [M + H]^+^ 490.2925 found 490.2956.

##### Procedure for 5-[2-(difluoromethyl)-1*H*-1,3-benzimidazol-1-yl]-7-(morpholin-4-yl)pyrazolo[1,5-*a*]pyrimidine-2-carboxylate (**13**)

Compound **13** was synthesized from ethyl 5-chloro-7-(morpholin-4-yl)pyrazolo[1,5-*a*]pyrimidine-2-carboxylate (100 mg, 0.31 mmol), 2-(difluoromethyl)-1*H*-benzimidazole (79.5 mg, 0.47 mmol), tris(dibenzylideneacetone)dipalladium (14.4 mg, 0.015 mmol), 9,9-dimethyl-4,5-bis(diphenylphosphino)xanthene (19.2 mg, 0.03 mmol), cesium carbonate (0.21 g, 0.63 mmol), and toluene (2.0 mL), according to the general procedure for the Buchwald–Hartwig reaction. The crude product was purified by flash chromatography (0–50% AcOEt gradient in heptane; amino-functionalized gel column) to give the title compound **13** as a light yellow solid (65.0 mg, 0.31 mmol) with a 47% yield.

Alternatively, **13** can be synthesized as follows. Ethyl 5-chloro-7-(morpholin-4-yl)pyrazolo[1,5-*a*]pyrimidine-2-carboxylate (100 g, 312 mmol), 2-(difluoromethyl)-1*H*-benzimidazole (79.5 g, 473 mmol), tetra ethyl ammonium chloride (78.0 g, 471 mmol), potassium carbonate (87.0 g, 623 mmol), and DMF (1000 mL) were added to a reactor (2000 mL volume). The reaction was heated at 160 °C for 3 h. Then, the reaction was cooled to room temperature, filtered through Celite^®^, and washed with AcOEt (1.5 l). Water (3.0 mL) was added to the filtrate and phases were separated. The organic layer was concentrated. The solid was dissolved in 20% MeOH in DCM and the crude product was then purified by filtration through silica gel (0.72 kg) (20% MeOH in DCM) and macerated in TBME to give **13** (123 g, 312 mmol) as a light yellow solid with an 89% yield. ^1^H NMR (300 MHz, CDCl_3_) *δ* 7.95–7.89 (m, 1H, Ar-H), 7.71–7.65 (m, 1H, Ar-H), 7.46–7.39 (m, 2H, Ar-H), 7.30 (t, *J =* 54.0 Hz, 1H, CHF_2_), 7.11 (s, 1H,Ar-H), 6.47 (s, 1H), Ar-H, 4.48 (q, *J =* 7.1 Hz, 2H, CH_22_), 4.03–3.99 (m, 4H, morph.), 3.98–3.93 (m, 4H, morph.), 1.45 (dd, *J =* 8.1, 6.2 Hz, 3H, CH_3_).

##### Procedure for {5-[2-(difluoromethyl)-2,3-dihydro-1*H*-1,3-benzodiazol-1-yl]-7-(morpholin-4-yl)pyrazolo[1,5-*a*]pyrimidin-2-yl}methanol (**14**)

Lithium aluminum hydride solution (1M in THF, 8.00 mL, 7.20 g, 8.00 mmol) was added to the suspension of **13** (2.40 g, 5.30 mmol) in dry THF (28.0 mL) at 0 °C. The suspension was stirred at 0 °C for 3 h. The reaction was quenched with 1.0 M HCl (14.0 mL). Then, water (70 mL) and AcOEt (90 mL) were added and the mixture was then allowed to warm to room temperature and stirred for 0.5 h. The organic layer was separated, dried over Na_2_SO_4_, and filtered. The solvent was removed under reduced pressure. The solid was macerated with DCM to give the title compound **14** (1.90 g, 4.72 mmol) as a light yellow solid with an 89% yield. ^1^H NMR (300 MHz, DMSO-*d_6_*) *δ* 7.70 (d, *J =* 7.7 Hz, 1H, Ar-H), 6.74 (td, *J =* 7.6, 1.2 Hz, 1H), 6.62 (ddd, *J =* 8.7, 6.9, 2.4 Hz, 3H), 6.12 (s, *J =* 54.0 Hz, 1H, Ar-H), 6.10 (s, 1H, Ar-H), 5.13 (t, *J =* 5.9 Hz, 1H, OH), 4.47 (d, *J =* 5.9 Hz, 2H, CH_2_), 3.74 (t, *J =* 4.5 Hz, 4H, morph.), 3.65–3.54 (m, 4H, morph.).

##### Procedure for 5-[2-(difluoromethyl)-1*H*-1,3-benzimidazol-1-yl]-7-(morpholin-4-yl)pyrazolo[1,5-*a*]pyrimidine-2-carbaldehyde (**15**)

Dess–Martin reagent (2.90 g, 6.63 mmol) was added to the solution of **14** (1.30 g, 3.23 mmol) in dry DMF (33 mL). The whole mixture was stirred at room temperature for 1 h. The solid was filtered off and then washed with ethyl acetate (25 mL). The obtained solution was concentrated under reduced pressure. The crude product was purified by flash chromatography (0–70% ethyl acetate gradient in heptane) to give **15** (1.02 g, 2.56 mmol) as a white solid with a 78% yield.

Alternatively, **15** can be synthesized as follows. Activated toluene:butyl acetate 1:1 (700 mL) manganese(IV) oxide (58.4 g, 667 mmol) was added to the solution of **14** (27.2 g, 67.1 mmol). The mixture was stirred at reflux (set temp: 120 °C) for 1.5 h. The reaction was then filtered through Celite^®^_._ Celite^®^ was washed with DCM (200 mL). Organic phases were combined, and concentrated to give **15** (18.2 g, 45.7 mmol) as a creamy solid with a 68% yield. ^1^H NMR (300 MHz, CDCl_3_) *δ* 10.21 (s, 1H, CHO), 7.97–7.90 (m, 1H, Ar-H), 7.73–7.67 (m, 1H, Ar-H), 7.48–7.42 (m, 2H, Ar-H), 7.29 (t, *J =* 54.0 Hz, 1H, CHF_2_), 7.11 (s, 1H, Ar-H), 6.53 (s, 1H, Ar-H), 4.03 (dd, *J =* 6.1, 2.7 Hz, 4H, morph.), 3.96 (dd, *J =* 6.3, 2.9 Hz, 4H, morph.).

##### General Procedure for the Amidation Reaction

Corresponding amine (1.05 eq), 2-(7-aza-1*H*-benzotriazole-1-yl)-1,1,3,3-tetramethyluronium hexafluorophosphate (HATU) (1.1 eq), and triethylamine (1.5 eq) were added to the solution of substituted 5-chloro-pyrazolo[1,5-*a*]pyrimidine derivative (1.0 eq) in solvent (10 mL/1 g pyrazolo[1,5-*a*]pyrimidine derivative). The mixture was stirred at room temperature for 2 h. Water was added to the reaction mixture and phases were separated. The aqueous phase was extracted three times with the solvent. Combined organic phases were dried over anhydrous sodium sulfate, filtered, and concentrated. The residue was purified by flash chromatography.

2-(difluoromethyl)-1-{2-[(4-methanesulfonylpiperazin-1-yl)methyl]-7-(morpholin-4-yl)pyrazolo[1,5-*a*]pyrimidin-5-yl}-1*H*-1,3-benzimidazole (**16**)

Compound **16** was prepared from aldehyde **15** (0.48 g, 1.18 mmol), 1-methanesulfonylpiperazine (0.24 g, 1.42 mmol) as an amine, DCM (4.80 mL), and sodium triacetoxyborohydride (0.38 g, 1.87 mmol), according to the general procedure for the reductive amination reaction. The crude product was purified by flash chromatography (0–100% AcOEt gradient in heptane) and crystallization (AcOEt) to give the title compound **16** (0.35 g, 0.63 mmol) as a white solid with a 54% yield. ^1^H NMR (600 MHz, CDCl_3_) *δ* 7.93–7.91 (m, 1H, Ar-H), 7.66–7.65 (m, 1H, Ar-H), 7.45–7.40 (m, 2H, Ar-H), 7.29 (t, *J* = 52.6 Hz, 1H, CHF_2_), 6.58 (s, 1H, Ar-H), 6.33 (s, 1H, Ar-H), 4.00–3.98 (m, 4H, morph.), 3.91–3.89 (m, 4H, morph.), 3.83 (s, 2H, CH_2_), 3.29 (t, *J* = 4.6 Hz, 4H), 2.78 (s, 3H, CH_3_), 2.70 (t, *J* = 4.8 Hz, 4H). ^13^C{^1^H, ^19^F}NMR (151 MHz, CDCl_3_) *δ* 155.1, 151.4, 150.2, 147.7, 144.6, 141.9, 134.6, 125.7, 124.2, 121.6, 111.7, 109.3 (CF_2_), 96.3, 87.5, 66.2, 56.2, 52.4, 48.6, 45.9, 34.3. HRMS (ESI/MS): *m*/*z* calculated for C_24_H_28_F_2_N_8_O_3_S [M + H]^+^ 547.2045 found 547.2048.

2-[4-({5-[2-(difluoromethyl)-1*H*-1,3-benzimidazol-1-yl]-7-(morpholin-4-yl)pyrazolo[1,5-*a*]pyrimidin-2-yl}methyl)piperazin-1-yl]-2-methylpropanamide (**17**)

Compound **17** was prepared from aldehyde **15** (0.50 g, 1.23 mmol), 2-methyl-2-(piperazin-1-yl)propanamide dihydrochloride (0.38 g, 1.48 mmol) as an amine, DCM (5.0 mL), and sodium triacetoxyborohydride (0.40 g, 1.85 mmol), according to the general procedure for the reductive amination reaction. The crude product was purified by flash chromatography (0–15% MeOH gradient in AcOEt) to give **17** (0.49 g, 0.88 mmol) as a light yellow solid with a 72% yield. ^1^H NMR (400 MHz, CDCl_3_) *δ* 7.93–7.90 (m, 1H, Ar-H), 7.67–7.63 (m, 1H, Ar-H), 7.45–7.39 (m, 2H, Ar-H), 7.23 (t, *J* = 53.6 Hz, 1H, CHF_2_), 7.13 (d, *J* = 5.2 Hz, 1H), 6.59 (s, 1H, Ar-H), 6.32 (s, 1H, Ar-H), 5.46 (d, *J* = 5.1 Hz, 1H), 4.00–3.96 (m, 4H, morph.), 3.93–3.89 (m, 4H, morph.), 3.79 (s, 2H), 2.60 (s, 8H), 1.22 (s, 6H, 2xCH_3_). ^13^C{^1^H, ^19^F}NMR (101 MHz, CDCl_3_) *δ* 180.1, 155.5, 151.3, 150.1, 147.6, 144.7, 141.8, 134.5, 125.7, 124.2, 121.6, 111.7, 109.3 (CF_2_), 96.5, 87.3, 66.2, 63.5, 56.4, 53.9, 48.5, 46.6, 20.6. HRMS (ESI/MS): *m*/*z* calculated for C_27_H_33_F_2_N_9_O_2_ [M + H]^+^ 554.2798 found 554.2800.

2-{2-[(4-cyclopropanecarbonylpiperazin-1-yl)methyl]-7-(morpholin-4-yl)pyrazolo[1,5-*a*]pyrimidin-5-yl}-2-(difluoromethyl)-1*H*-1,3-benzimidazole (**18**)

Compound **18** was prepared from aldehyde **15** (0.50 g, 1.23 mmol), 1-(cyclopropylcarbonyl)piperazine (0.21 mL, 0.23 g, 1.48 mmol) as an amine, DCM (5.0 mL), and sodium triacetoxyborohydride (0.40 g, 1.85 mmol), according to the general procedure for the reductive amination reaction. The crude product was purified by flash chromatography (0–15% MeOH gradient in AcOEt) and crystallization (AcOEt) to give **18** (0.45 g, 0.84 mmol) as a white solid with a 68% yield. ^1^H NMR (400 MHz, CDCl_3_) *δ* 7.93–7.90 (m, 1H, Ar-H), 7.67–7.64 (m, 1H, Ar-H), 7.46–7.39 (m, 2H, Ar-H), 7.23 (t, *J* = 53.6 Hz, 1H, CHF_2_), 6.61 (s, 1H, Ar-H), 6.33 (s, 1H, Ar-H), 4.00–3.96 (m, 4H, morph.), 3.93–3.89 (m, 4H, morph.), 3.82 (s, 2H, CH_2_), 3.73–3.69 (m, 4H), 2.60 (d, *J* = 24.3 Hz, 4H), 1.76–1.70 (m, 1H, CH), 1.00–0.96 (m, 2H, CH_2_), 0.77–0.73 (m, 2H, CH_2_). ^13^C{^1^H, ^19^F}NMR (101 MHz, CDCl_3_) *δ* 171.9, 155.3, 151.4, 150.1, 147.7, 144.6, 141.9, 134.6, 125.7, 124.2, 121.6, 111.7, 109.3 (CF_2_), 96.4, 87.4, 66.2, 56.4, 48.5, 10.9, 7.4. HRMS (ESI/MS): *m*/*z* calculated for C_27_H_30_F_2_N_8_O_2_ [M + H]^+^ 537.2532 found 537.2541.

[1-({5-[2-(difluoromethyl)-1*H*-1,3-benzimidazol-1-yl]-7-(morpholin-4-yl)pyrazolo[1,5-*a*]pyrimidin-2-yl}methyl)pyrrolidin-2-yl]methanol (**19**)

Compound **19** was prepared from aldehyde **15** (0.50 g, 1.23 mmol), 2-pyrrolidinylmethanol (0.14 mL, 0.15 g, 1.48 mmol) as an amine, DCM (5.0 mL), and sodium triacetoxyborohydride (0.40 g, 1.85 mmol), according to the general procedure for the reductive amination reaction. The crude product was purified by flash chromatography (0–20% MeOH gradient in AcOEt) to give **19** (0.33 g, 0.68 mmol) as a white solid with a 55% yield. ^1^H NMR (400 MHz, CDCl_3_) *δ* 7.93–7.90 (m, 1H, Ar-H), 7.67–7.65 (m, 1H, Ar-H), 7.45–7.38 (m, 2H, Ar-H), 7.29 (t, *J* = 53.6 Hz, 1H, CHF_2_), 6.56 (s, 1H, Ar-H), 6.33 (s, 1H, Ar-H), 4.14 (d, *J* = 14.3 Hz, 1H, OH), 4.00–3.98 (m, 4H), 3.90–3.82 (m, 6H), 3.70 (dd, *J* = 11.0, 3.5 Hz, 1H), 3.47 (dd, *J* = 11.0, 3.2 Hz, 1H), 3.20–3.16 (m, 1H), 2.90–2.85 (m, 1H), 2.73–2.72 (m, 1H), 2.59–2.54 (m, 1H), 1.97–1.92 (m, 1H), 1.82–1.75 (m, 2H, CH_2_). ^13^C{^1^H, ^19^F}NMR (101 MHz, CDCl_3_) *δ* 156.5, 151.4, 150.1, 147.6, 144.7, 141.8, 134.5, 125.7, 124.2, 121.6, 111.7, 109.3 (CF_2_), 96.2, 87.5, 66.2, 64.3, 62.3, 54.8, 51.7, 48.6, 27.7, 25.3, 23.5. HRMS (ESI/MS): *m*/*z* calculated for C_24_H_27_F_2_N_7_O_2_ [M + H]^+^ 484.2267 found 484.2271.

1-({5-[2-(difluoromethyl)-1*H*-1,3-benzimidazol-1-yl]-7-(morpholin-4-yl)pyrazolo[1,5-*a*]pyrimidin-2-yl}methyl)pyrrolidin-3-amine (**20**)

Compound *tert*-butyl *N*-[1-({5-[2-(difluoromethyl)-1*H*-1,3-benzodiazol-1-yl]-7-(morpholin-4-yl)pyrazolo[1,5-*a*]pyrimidin-2-yl}methyl)pyrrolidin-3-yl]carbamate (**Boc-20**) was prepared from aldehyde **15** (0.50 g, 1.23 mmol), 3-(Boc-amino)pyrrolidine (0.27 g, 1.48 mmol) as an amine, DCM (5.0 mL), and sodium triacetoxyborohydride (0.40 g, 1.85 mmol), according to the general procedure for the reductive amination reaction. The crude product was purified by flash chromatography (0–10% MeOH gradient in AcOEt) to give **Boc-20** (0.50 g, 0.88 mmol) as a white solid with a 71% yield. ^1^H NMR (400 MHz, CDCl_3_) *δ* 7.93–7.91 (m, 1H, Ar-H), 7.67–7.65 (m, 1H, Ar-H), 7.45–7.39 (m, 2H, Ar-H), 7.23 (t, *J* = 52.5 Hz, 1H, CHF_2_), 6.58 (s, 1H, Ar-H), 6.32 (s, 1H, Ar-H), 4.91–4.90 (m, 1H), 4.20 (d, *J* = 3.4 Hz, 1H), 4.00–3.96 (m, 4H), 3.93–3.82 (m, 6H), 2.97 (d, *J* = 3.0 Hz, 1H), 2.72 (s, 2H, CH_2_), 2.47 (d, *J* = 8.0 Hz, 1H), 2.32–2.27 (m, 1H), 1.67–1.63 (m, 1H), 1.43 (s, 9H, t-Bu.). ^13^C{^1^H, ^19^F}NMR (101 MHz, CDCl_3_) *δ* 156.0, 155.4, 151.4, 150.1, 147.6, 144.7, 141.8, 134.6, 125.7, 124.2, 121.5, 111.7, 109.3 (CF_2_), 96.2, 87.4, 66.2, 61.0, 53.4, 52.8, 48.5, 32.7, 28.4 (t-Bu). HRMS (ESI/MS): *m*/*z* calculated for C_28_H_34_F_2_N_8_O_3_ [M + H]^+^ 569.2794 found 569.2803.

The solution of **Boc-20** (0.40 g, 0.69 mmol) in trifluoroacetic acid (2.08 mL, 3.09 g, 26.9 mmol) was heated at 50 °C for 3 h. The reaction was then cooled to room temperature, stopped with 15% NaOH (15 mL). The aqueous mixture was extracted with DCM (3 × 15 mL). The combined organic extracts were washed with water and dried over Na_2_SO_4_, filtered, and concentrated to give **20** as a white solid (0.30 g, 0.64 mmol) with a 93% yield. ^1^H NMR (400 MHz, DMSO-*d_6_*) *δ* 8.04 (s, 2H, NH_2_), 7.89–7.87 (m, 1H, Ar-H), 7.81 (dd, *J* = 6.9, 1.4 Hz, 1H, Ar-H), 7.59 (t, *J* = 52.6 Hz, 1H, CHF_2_), 7.49–7.41 (m, 2H, Ar-H), 6.69 (s, 1H, Ar-H), 6.66 (s, 1H, Ar-H), 3.94–3.89 (m, 6H), 3.84–3.82 (m, 5H), 3.73 (d, *J* = 5.0 Hz, 1H), 2.80 (s, 2H, CH_2_), 2.13–2.23 (1H), 1.68–1.80 (1H), 1.22 (s, 1H). ^13^C{^1^H, ^19^F}NMR (101 MHz, DMSO-*d_6_*) *δ* 158.0, 150.9, 149.7, 147.2, 144.7, 141.2, 134.1, 125.5, 124.0, 120.7, 117.3, 112.4, 108.6, 95.5, 88.0, 65.6, 51.9, 48.3, 29.2. HRMS (ESI/MS): *m*/*z* calculated for C_23_H_26_F_2_N_8_O [M + H]^+^ 469.2270 found 469.2273.

(3*S*)-1-({5-[2-(difluoromethyl)-1*H*-1,3-benzimidazol-1-yl]-7-(morpholin-4-yl)pyrazolo[1,5-*a*]pyrimidin-2-yl}methyl)pyrrolidin-3-ol (**21**)

Compound **21** was prepared from aldehyde **15** (0.50 g, 1.23 mmol), (*S*)-3-pyrrolidinol (0.16 mL, 0.17 g, 1.85 mmol) as an amine, DCM (5.0 mL), and sodium triacetoxyborohydride (0.40 g, 1.85 mmol), according to the general procedure for the reductive amination reaction. The crude product was purified by flash chromatography (0–100% AcOEt gradient in heptane, amino-functionalized gel column) to give **21** (0.22 g, 0.47 mmol) as a light yellow solid with a 38% yield. ^1^H NMR (400 MHz, CDCl_3_) *δ* 7.94–7.90 (m, 1H, Ar-H), 7.67–7.64 (m, 1H, Ar-H), 7.45–7.39 (m, 2H, Ar-H), 7.23 (t, *J* = 52.5 Hz, 1H, CHF_2_), 6.60 (s, 1H, Ar-H), 6.32 (s, 1H, Ar-H), 4.41–4.37 (m, 1H, OH), 4.00–3.96 (m, 4H, morph.), 3.93–3.89 (m, 6H), 3.07–3.01 (m, 1H), 2.84 (dd, *J* = 10.1, 1.6 Hz, 1H, CH), 2.74 (dd, *J* = 10.1, 5.2 Hz, 1H), 2.53 (td, *J* = 8.9, 6.2 Hz, 1H), 2.46–2.31 (m, 1H), 2.27–2.19 (m, 1H), 1.84–1.77 (m, 1H). ^13^C{^1^H, ^19^F}NMR (101 MHz, CDCl_3_) *δ* 156.0, 151.4, 150.1, 147.6, 144.7, 141.8, 134.6, 125.7, 124.2, 121.6, 111.7, 109.3 (CF_2_), 96.3, 87.4, 71.5, 66.2, 62.8, 53.3, 52.4, 48.5, 35.1. HRMS (ESI/MS): *m*/*z* calculated for C_23_H_25_F_2_N_7_O_2_ [M + H]^+^ 470.2110 found 470.2134.

(3*R*)-1-({5-[2-(difluoromethyl)-1*H*-1,3-benzimidazol-1-yl]-7-(morpholin-4-yl)pyrazolo[1,5-*a*]pyrimidin-2-yl}methyl)pyrrolidin-3-ol (**22**)

Compound **22** was prepared from aldehyde **15** (0.50 mg, 1.23 mmol), (*R*)-3-pyrrolidinol (0.16 mL, 0.17 g, 1.85 mmol) as an amine, DCM (5.0 mL), and sodium triacetoxyborohydride (0.40 g, 1.85 mmol), according to the general procedure for the reductive amination reaction. The crude product was purified by flash chromatography (0–100% AcOEt gradient in heptane, amine gel column) to give **22** (0.34 g, 0.73 mmol) as a white solid with a 60% yield. ^1^H NMR (400 MHz, CDCl_3_) *δ* 7.94–7.90 (m, 1H, Ar-H), 7.68–7.63 (m, 1H, Ar-H), 7.45–7.39 (m, 2H, Ar-H), 7.23 (t, *J* = 52.5 Hz, 1H, CHF_2_), 6.60 (s, 1H, Ar-H), 6.31 (s, 1H, Ar-H), 4.40–4.36 (m, 1H, OH), 4.00–3.97 (m, 4H, morph.), 3.92–3.88 (m, 6H), 3.04–2.99 (m, 1H), 2.82 (dd, *J* = 10.0, 2.1 Hz, 1H), 2.73 (dd, *J* = 10.1, 5.1 Hz, 1H), 2.51 (td, *J* = 8.9, 6.2 Hz, 1H), 2.27–2.18 (m, 2H), 1.83–1.75 (m, 1H). ^13^C{^1^H, ^19^F}NMR (101 MHz, CDCl_3_) *δ* 156.2, 151.4, 150.1, 147.6, 144.7, 141.8, 134.6, 125.7, 124.2, 121.5, 111.7, 109.3 (CF_2_), 96.3, 87.4, 71.5, 66.2, 62.9, 53.4, 52.4, 48.5, 35.1. HRMS (ESI/MS): *m*/*z* calculated for C_23_H_25_F_2_N_7_O_2_ [M + H]^+^ 470.2110 found 470.2115.

2-(difluoromethyl)-1-[7-(morpholin-4-yl)-2-(morpholin-4-ylmethyl)pyrazolo[1,5-*a*]pyrimidin-5-yl]-1*H*-1,3-benzimidazole (**23**)

Compound **23** was prepared from aldehyde **15** (0.50 g, 1.23 mmol), morpholine (0.13 mL, 0.13 g, 1.48 mmol) as an amine, DCM (5.00 mL), and sodium triacetoxyborohydride (0.40 g, 1.85 mmol), according to the general procedure for the reductive amination reaction. The crude product was purified by flash chromatography (0–15% AcOEt gradient in heptane, amino-functionalized gel column) and crystallization) (AcOEt) to give the title compound **23** (0.28 g, 0.60 mmol) as a white solid with a 50% yield. ^1^H NMR (300 MHz, CDCl_3_) *δ* 7.89–7.81 (m, 1H, Ar-H), 7.63–7.54 (m, 1H, Ar-H), 7.38–7.30 (m, 2H, Ar-H), 7.23 (d, *J* = 54.0 Hz, 1H, CHF_2_), 6.55 (s, 1H, Ar-H), 6.25 (s, 1H, Ar-H), 3.92 (dd, *J* = 6.0, 2.9 Hz, 4H, morph.), 3.83 (dd, *J* = 6.1, 3.0 Hz, 4H, morph.), 3.72 (s, 2H, CH_2_), 3.71–3.66 (m, 4H, morph.), 2.58–2.49 (m, 4H, morph.). ^13^C{^1^H}NMR (75 MHz, CDCl_3_) *δ* 155.6, 151.5, 150.3, 147.8, 144.84 (t, *J* = 26.2 Hz), 142.1, 134.8, 125.0, 124.3, 121.7, 111.9, 109.5 (t, *J* = 238.5 Hz) (CF_2_), 96.7, 87.5, 77.6, 77.2, 76.7, 67.1, 66.4, 57.1, 53.8, 48.7, 31.0. HRMS (ESI/MS): *m*/*z* calculated for C_23_H_25_F_2_N_7_O_2_ [M + H]^+^ 470.2110 found 470.2113.

2-(difluoromethyl)-1-(2-{[(3*S*)-3-methylmorpholin-4-yl]methyl}-7-(morpholin-4-yl)pyrazolo[1,5-*a*]pyrimidin-5-yl)-1*H*-1,3-benzimidazole (**24**)

Compound **24** was prepared from aldehyde **15** (0.50 g, 1.23 mmol), (*R*)-3-methylmorpholine (0.15 g, 1.48 mmol) as an amine, DCM (5.0 mL), and sodium triacetoxyborohydride (0.40 g, 1.85 mmol), according to the general procedure for the reductive amination reaction. The crude product was purified by flash chromatography (0–100% AcOEt gradient in heptane) and crystallization (AcOEt) to give **24** (0.32 g, 0.67 mmol) as a white solid with a 54% yield. ^1^H NMR (400 MHz, CDCl_3_) *δ* 7.93–7.90 (m, 1H, Ar-H), 7.67–7.64 (m, 1H, Ar-H), 7.45–7.39 (m, 2H, Ar-H), 7.23 (t, *J* = 52.5 Hz, 1H, CHF_2_), 6.57 (s, 1H, Ar-H), 6.32 (s, 1H, Ar-H), 4.07 (d, *J* = 14.4 Hz, 1H, CH), 4.00–3.97 (m, 4H, morph.), 3.93–3.89 (m, 4H, morph.), 3.84 (d, *J* = 14.4 Hz, 1H), 3.79 (dt, *J* = 11.2, 2.6 Hz, 1H), 3.73–3.63 (m, 2H), 3.30 (dd, *J* = 11.2, 9.3 Hz, 1H), 2.78 (dt, *J* = 11.8, 2.5 Hz, 1H), 2.60–2.51 (m, 2H), 1.15 (d, *J* = 6.3 Hz, 3H, CH_3_). ^13^C{^1^H, ^19^F}NMR (101 MHz, CDCl_3_) *δ* 155.1, 151.2, 150.0, 147.6, 144.7, 141.9, 134.5, 125.7, 124.2, 121.6, 111.7, 109.3 (CF_2_), 96.8, 87.3, 73.0, 67.4, 66.2, 54.5, 51.7, 51.6, 49.3, 48.5, 14.4. HRMS (ESI/MS): *m*/*z* calculated for C_24_H_27_F_2_N_7_O_2_ [M + H]^+^ 484.2267 found 484.2269.

2-(difluoromethyl)-1-(2-{[(3*R*)-3-methylmorpholin-4-yl]methyl}-7-(morpholin-4-yl)pyrazolo[1,5-*a*]pyrimidin-5-yl)-1*H*-1,3-benzimidazole (**25**)

Compound **25** was prepared from aldehyde **15** (0.50 g, 1.23 mmol), (*S*)-3-methylmorpholine (0.16 g, 1.51 mmol) as an amine, DCM (5.0 mL), and sodium triacetoxyborohydride (0.40 g, 1.85 mmol), according to the general procedure for the reductive amination reaction. The crude product was purified by flash chromatography (0–100% AcOEt gradient in heptane) and crystallization (AcOEt) to give **25** (0.33 g, 0.68 mmol) as a white solid with a 55% yield. ^1^H NMR (400 MHz, CDCl_3_) *δ* 7.93–7.90 (m, 1H, Ar-H), 7.68–7.64 (m, 1H, Ar-H), 7.45–7.39 (m, 2H, Ar-H), 7.23 (t, *J* = 52.5 Hz, 1H, CHF_2_), 6.58 (s, 1H, Ar-H), 6.32 (s, 1H, Ar-H), 4.07 (d, *J* = 14.4 Hz, 1H, CH), 4.00–3.97 (m, 4H, morph.), 3.93–3.89 (m, 4H, morph.), 3.85 (d, *J* = 14.4 Hz, 1H), 3.81–3.77 (m, 1H), 3.73–3.63 (m, 2H), 3.30 (dd, *J* = 11.1, 9.3 Hz, 1H), 2.78 (dt, *J* = 11.8, 2.5 Hz, 1H), 2.61–2.51 (m, 2H, CH_2_), 1.15 (d, *J* = 6.3 Hz, 3H, CH_3_). ^13^C{^1^H, ^19^F}NMR (101 MHz, CDCl_3_) *δ* 155.1, 151.3, 150.0, 147.6, 144.7, 141.9, 134.6, 125.7, 124.2, 121.6, 111.7, 109.3 (CF_2_), 96.8, 87.3, 73.0, 67.4, 66.2, 54.5, 51.7, 51.6, 48.5, 14.4. HRMS (ESI/MS): *m*/*z* calculated for C_24_H_27_F_2_N_7_O_2_ [M + H]^+^ 484.2267 found 484.2268.

1-({5-[2-(difluoromethyl)-1*H*-1,3-benzodiazol-1-yl]-7-(morpholin-4-yl)pyrazolo[1,5-*a*]pyrimidin-2-yl}methyl)piperidine-3-carboxylate (**26**)

Compound **26** was prepared from aldehyde **15** (0.50 g, 1.23 mmol), ethyl piperidine-3-carboxylate (0.23 mL, 0.24 g, 1.48 mmol) as an amine, DCM (5.0 mL), and sodium triacetoxyborohydride (0.40 g, 1.85 mmol), according to the general procedure for the reductive amination reaction. The crude product was purified by flash chromatography (0–100% AcOEt gradient in heptane) to give **25** (0.56 g, 1.04 mmol) as a white solid with a 84% yield. ^1^H NMR (400 MHz, CDCl_3_) *δ* 7.93–7.91 (m, 1H, Ar-H), 7.67–7.64 (m, 1H, Ar-H), 7.45–7.40 (m, 2H, Ar-H), 7.24 (t, *J* = 53.6 Hz, 1H, CHF_2_), 6.59 (s, 1H, Ar-H), 6.30 (s, 1H, Ar-H), 4.15–4.10 (m, 2H, CH2), 4.00–3.98 (m, 4H, morph.), 3.91–3.89 (m, 4H, morph.), 3.80 (s, 2H), 3.09 (dd, *J* = 10.9, 2.9 Hz, 1H), 2.88–2.86 (m, 1H), 2.65–2.58 (m, 1H), 2.38–2.33 (m, 1H), 2.20 (td, *J* = 10.9, 2.9 Hz, 1H), 1.98–1.93 (m, 1H), 1.80–1.73 (m, 1H), 1.69–1.58 (m, 1H), 1.26–1.23 (m, 3H, CH_3_). ^13^C{^1^H, ^19^F}NMR (101 MHz, CDCl_3_) *δ* 174.1, 156.0, 151.3, 150.0, 147.5, 144.7, 141.9, 134.6, 125.7, 124.1, 121.5, 111.7, 109.2 (CF_2_), 96.4, 87.2, 66.2, 60.3, 56.8, 55.4, 53.8, 48.5, 41.9, 26.8, 24.6, 14.2. HRMS (ESI/MS): *m*/*z* calculated for C_28_H_34_F_2_N_8_O_2_ [M + H]^+^ 540.2529 found 540.2536.

*N*-({5-[2-(difluoromethyl)-1*H*-1,3-benzimidazol-1-yl]-7-(morpholin-4-yl)pyrazolo[1,5-*a*]pyrimidin-2-yl}methyl)oxan-4-amine (**27**)

Compound **27** was prepared from aldehyde **15** (0.50 g, 1.23 mmol), 4-aminotetrahydropyran (0.16 mL, 0.16 g, 1.51 mmol) as an amine, DCM (5.0 mL), and sodium triacetoxyborohydride (0.40 g, 1.85 mmol), according to the general procedure for the reductive amination reaction. The crude product was purified by flash chromatography (0–10% MeOH gradient in AcOEt) to give **27** (0.45 g, 0.95 mmol) as a white solid with a 77% yield. ^1^H NMR (600 MHz, DMSO-*d_6_*) *δ* 7.88 (d, *J* = 7.6 Hz, 1H, Ar-H), 7.82–7.80 (m, 1H, Ar-H), 7.58 (t, *J* = 52.6 Hz, 1H, CHF_2_), 7.47–7.42 (m, 2H, Ar-H), 6.64 (s, 1H, Ar-H), 6.60 (s, 1H, Ar-H), 3.93 (d, *J* = 7.7 Hz, 7H), 3.84–3.81 (m, 6H), 3.26 (td, *J* = 11.4, 2.1 Hz, 2H, CH_2_), 2.71–2.66 (m, 1HCH), 1.80 (dd, *J* = 12.5, 1.7 Hz, 2H, CH_2_), 1.32–1.26 (m, 2HCH_2_). ^13^C{^1^H, ^19^F}NMR (151 MHz, DMSO-*d_6_*) *δ* 157.9, 150.9, 149.6, 146.9, 144.7, 141.2, 134.1, 125.5, 123.9, 120.7, 112.4, 108.6, 94.5, 87.6, 65.7, 65.6, 52.4, 48.1, 43.6, 33.0. HRMS (ESI/MS): *m*/*z* calculated for C_24_H_27_F_2_N_7_O_2_ [M + H]^+^ 484.2267 found 484.2266.

2-(difluoromethyl)-1-{2-[(4,4-difluoropiperidin-1-yl)methyl]-7-(morpholin-4-yl)pyrazolo[1,5-*a*]pyrimidin-5-yl}-1*H*-1,3-benzimidazole (**28**)

Compound **28** was prepared from aldehyde **15** (0.50 g, 1.23 mmol), 4,4-difluoropiperidine (0.24 g, 1.48 mmol) as an amine, DCM (5.0 mL), and sodium triacetoxyborohydride (0.40 g, 1.85 mmol), according to the general procedure for the reductive amination reaction. The crude product was purified by flash chromatography (0–100% AcOEt gradient in heptane) and crystallization (AcOEt) to give **28** (0.41 g, 0.81 mmol) as a white solid with a 66% yield. ^1^H NMR (400 MHz, CDCl_3_) *δ* 7.93–7.91 (m, 1H, Ar-H), 7.67–7.65 (m, 1H, Ar-H), 7.46–7.39 (m, 2H, Ar-H), 7.23 (t, *J* = 54.0 Hz, 1H,CHF_2_), 6.59 (s, 1H, Ar-H), 6.33 (s, 1H, Ar-H), 4.00–3.98 (m, 4H, morph.), 3.91–3.89 (m, 4H, morph.), 3.84 (s, 2H, CH_2_), 2.70 (t, *J* = 5.6 Hz, 4H), 2.09–1.99 (m, 4H). ^13^C{^1^H, ^19^F}NMR (101 MHz, CDCl_3_) *δ* 155.9, 151.5, 150.3, 147.8, 144.8, 142.0, 134.7, 125.9, 124.4, 121.8, 111.9, 109.5 (CF_2_), 96.5, 96.4, 87.6, 66.4, 55.9, 50.2, 48.7, 34.2. HRMS (ESI/MS): *m*/*z* calculated for C_24_H_25_F_4_N_7_O [M + H]^+^ 504.2194 found 504.2131.

1-({5-[2-(difluoromethyl)-1*H*-1,3-benzimidazol-1-yl]-7-(morpholin-4-yl)pyrazolo[1,5-*a*]pyrimidin-2-yl}methyl)piperidine-4-carboxamide (**29**)

Compound **29** was prepared from aldehyde **15** (0.50 g, 1.23 mmol), 4-piperidinecarboxamide (0.19 g, 1.48 mmol) as an amine, DCM (5.0 mL) and sodium triacetoxyborohydride (0.40 g, 1.85 mmol), according to the general procedure for the reductive amination reaction. The crude product was purified by crystallization (AcOEt/DCM, 90:10, *v*/*v*) to give **29** (0.40 g, 0.78 mmol) as a white solid with a 64% yield. ^1^H NMR (300 MHz, CDCl_3_) *δ* 7.95–7.88 (m, 1H, Ar-H), 7.70–7.62 (m, 1H, Ar-H), 7.46–7.37 (m, 2H,Ar-H), 7.30 (t, *J* = 54.0 Hz, 1H, CHF_2_), 6.59 (s, 1H, Ar-H), 6.31 (s, 1H, Ar-H), 5.48 (s, 2H, NH_2_), 3.98 (m, 4H, morph.), 3.90 (m, 4H, morph.), 3.78 (s, 2H, CH_2_), 3.06 (d, *J* = 11.7 Hz, 2H, CH_2_), 2.18 (t, *J* = 11.3 Hz, 3H), 1.96–1.73 (m, 4H). ^13^C{^1^H}NMR (75 MHz, CDCl_3_) *δ* 177.3, 156.1, 151.5, 150.3, 147.7, 144.8 (t, *J* = 26.2 Hz), 142.0, 134.8, 125.9, 124.3, 121.7, 111.9, 109.5 (t, *J* = 237.0 Hz, CF_2_), 96.6, 87.5, 66.4, 56.8, 53.3, 48.7, 31.7, 29.1, 22.8. HRMS (ESI/MS): *m*/*z* calculated for C_25_H_28_F_2_N_8_O_2_ [M + H]^+^ 511.2376 found 511.2377.

1-({5-[2-(difluoromethyl)-1*H*-1,3-benzimidazol-1-yl]-7-(morpholin-4-yl)pyrazolo[1,5-*a*]pyrimidin-2-yl}methyl)-4-methylpiperidin-4-ol (**30**)

Compound **30** was prepared from aldehyde **15** (0.50 g, 1.23 mmol), 4-methylpiperidin-4-ol (0.18 g, 1.48 mmol) as an amine, DCM (5.0 mL), and sodium triacetoxyborohydride (0.40 g, 1.85 mmol), according to the general procedure for the reductive amination reaction. The crude product was purified by flash chromatography (50–100% AcOEt gradient in heptane) and crystallization (AcOEt) to give **30** (0.33 g, 0.66 mmol) as a white solid with a 54% yield. ^1^H NMR (400 MHz, CDCl_3_) *δ* 7.93–7.91 (m, 1H, Ar-H), 7.67–7.65 (m, 1H, Ar-H), 7.45–7.40 (m, 2H, Ar-H), 7.23 (t, *J* = 54.0 Hz, 1H, CHF_2_), 6.60 (s, 1H, Ar-H), 6.30 (s, 1H, Ar-H), 4.00–3.96 (m, 4H, morph.), 3.93–3.89 (m, 4H, morph.), 3.81 (s, 2H, CH_2_), 2.74–2.69 (m, 2H, CH_2_), 2.57–2.51 (m, 2H, CH_2_), 1.77–1.70 (m, 2H, CH_2_), 1.63 (d, *J* = 13.2 Hz, 2H, CH_2_), 1.26 (s, 3H, CH_3_). ^13^C{^1^H, ^19^F} NMR (101 MHz, CDCl_3_) *δ* 156.1, 151.3, 150.1, 147.5, 144.7, 141.9, 134.6, 125.7, 124.1, 121.6, 111.7, 109.3 (CF_2_), 96.5, 87.2, 67.7, 66.2, 56.6, 49.8, 48.5, 38.8. HRMS (ESI/MS): *m*/*z* calculated for C_25_H_29_F_2_N_7_O_2_ [M + H]^+^ 498.2423 found 498.2422.

[1-({5-[2-(difluoromethyl)-1*H*-1,3-benzimidazol-1-yl]-7-(morpholin-4-yl)pyrazolo[1,5-*a*]pyrimidin-2-yl}methyl)piperidin-4-yl]methanol (**31**)

Compound **31** was prepared from aldehyde **15** (0.50 g, 1.23 mmol), 4-piperidinemethanol (0.17 g, 1.48 mmol) as an amine, DCM (5.0 mL), and sodium triacetoxyborohydride (0.40 g, 1.85 mmol), according to the general procedure for the reductive amination reaction. The crude product was purified by flash chromatography (0–10% MeOH gradient in AcOEt) and crystallization (*i*-PrOH) to give **31** (0.34 g, 0.68 mmol) as a white solid with a 56% yield. ^1^H NMR (600 MHz, CDCl_3_) *δ* 7.93–7.91 (m, 1H, Ar-H), 7.67–7.64 (m, 1H, Ar-H), 7.44–7.39 (m, 2H, Ar-H), 7.26 (t, *J* = 26.8 Hz, 1H, CHF_2_), 6.60 (s, 1H, Ar-H), 6.30 (s, 1H, OH), 3.99–3.98 (m, 4H, morph.), 3.91–3.89 (m, 4H, morph.), 3.78 (s, 2H, CH2), 3.51 (d, *J* = 6.5 Hz, 2H, CH_2_), 3.04 (d, *J* = 11.5 Hz, 2H CH_2_ 2.16–2.11 (m, 2H, CH_2_), 1.76–1.74 (m, 2H, CH_2_), 1.70–1.59 (m, 1H), 1.56–1.48 (m, 1H), 1.37–1.31 (m, 2H). ^13^C{^1^H, ^19^F} NMR (151 MHz, CDCl_3_) *δ* 156.2, 151.3, 150.1, 147.5, 144.7, 141.8, 134.6, 125.7, 124.1, 121.5, 111.7, 109.3 (CF_2_), 96.5, 87.2, 67.9, 66.2, 56.9, 53.6, 48.5, 38.4, 28.8. HRMS (ESI/MS): *m*/*z* calculated for C_25_H_29_F_2_N_7_O_2_ [M + H]^+^ 498.2423 found 498.2426.

1-({5-[2-(difluoromethyl)-1*H*-1,3-benzimidazol-1-yl]-7-(morpholin-4-yl)pyrazolo[1,5-*a*]pyrimidin-2-yl}methyl)piperidine-4-carbonitrile (**32**)

Compound **32** was prepared from aldehyde **15** (0.50 g, 1.23 mmol), 4-cyanopiperidine (0.17 mL, 0.16 g, 1.48 mmol) as an amine, DCM (5.0 mL), and sodium triacetoxyborohydride (0.40 g, 1.85 mmol), according to the general procedure for the reductive amination reaction. The crude product was purified by flash chromatography (0–100% AcOEt gradient in heptane) and crystallization (AcOEt) to give **32** (0.50 g, 1.01 mmol) as a white solid with a 82% yield. ^1^H NMR (600 MHz, CDCl_3_) *δ* 7.93–7.91 (m, 1H, Ar-H), 7.67–7.65 (m, 1H, Ar-H), 7.45–7.41 (m, 2H, Ar-H), 7.30 (t, *J* = 53.4 Hz, 1H, CHF_2_), 6.58 (s, 1H, Ar-H), 6.32 (s, 1H, Ar-H), 4.00–3.98 (m, 4H, morph.), 3.91–3.89 (m, 4H, morph.), 3.79 (s, 2H, CH_2_), 2.79 (s, 2H, CH_2_), 2.69 (s, 1H, CH), 2.51 (d, *J* = 1.3 Hz, 2H,CH_2_), 2.00–1.89 (m, 4H). ^13^C{^1^H, ^19^F} NMR (151 MHz, CDCl_3_) *δ* 155.6, 151.4, 150.2, 147.7, 144.7, 141.9, 134.6, 125.7, 124.2, 121.7, 121.6, 111.7, 109.3 (CF_2_), 96.4, 87.4, 66.2, 56.7, 51.3, 48.6, 28.8, 26.0. HRMS (ESI/MS): *m*/*z* calculated for C_25_H_26_F_2_N_8_O [M + H]^+^ 493.2270 found 493.2281.

2-(difluoromethyl)-1-[7-(morpholin-4-yl)-2-{[4-(morpholin-4-yl)piperidin-1-yl]methyl}pyrazolo[1,5-*a*]pyrimidin-5-yl]-1*H*-1,3-benzimidazole (**33**)

Compound **33** was prepared from aldehyde **15** (0.50 g, 1.23 mmol), 4-morpholinopiperidine (0.26 g, 1.48 mmol) as an amine, DCM (5.0 mL), and sodium triacetoxyborohydride (0.40 g, 1.85 mmol), according to the general procedure for the reductive amination reaction. The crude product was purified by flash chromatography (0–20% MeOH gradient in AcOEt) and crystallization (*i*-PrOH) to give **33** (0.37 g, 0.66 mmol) as a white solid with a 54% yield. ^1^H NMR (600 MHz, CDCl_3_) *δ* 7.92–7.91 (m, 1H, Ar-H), 7.66–7.65 (m, 1H, Ar-H), 7.44–7.39 (m, 2H, Ar-H), 7.26 (t, *J* = 53.4 Hz, 1H, CHF_2_), 6.59 (s, 1H, Ar-H), 6.30 (s, 1H, Ar-H), 3.99–3.98 (m, 4H, morph.), 3.91–3.89 (m, 4H, morph.), 3.77 (s, 2H, CH_2_), 3.72 (t, *J* = 4.6 Hz, 4H, morph.), 3.07 (d, *J* = 11.9 Hz, 2H, CH_2_), 2.55 (t, *J* = 4.6 Hz, 4H, morph.), 2.21 (t, *J* = 11.4, 3.8 Hz, 1H, CH), 2.13 (td, *J* = 11.8, 2.1 Hz, 2H, CH_2_), 1.84–1.82 (m, 2H, CH_2_), 1.64–1.57 (m, 2H, CH_2_). ^13^C{^1^H, ^19^F} NMR (151 MHz, CDCl_3_) *δ* 156.2, 151.3, 150.1, 147.5, 144.7, 141.9, 134.6, 125.7, 124.2, 121.6, 111.7, 109.3 (CF_2_), 96.5, 87.3, 67.3, 66.2, 62.1, 56.5, 53.2, 49.8, 48.5, 28.2. HRMS (ESI/MS): *m*/*z* calculated for C_28_H_34_F_2_N_8_O_2_ [M + H]^+^ 553.2845 found 553.2846.

1-({5-[2-(difluoromethyl)-1*H*-1,3-benzimidazol-1-yl]-7-(morpholin-4-yl)pyrazolo[1,5-*a*]pyrimidin-2-yl}methyl)piperidin-4-ol (**34**)

Compound **34** was prepared from aldehyde **15** (0.50 g, 1.23 mmol), 4-hydroxypiperidine (0.15 g, 1.48 mmol) as an amine, DCM (5.0 mL), and sodium triacetoxyborohydride (0.40 g, 1.85 mmol), according to the general procedure for the reductive amination reaction. The crude product was purified by flash chromatography (0–20% MeOH gradient in AcOEt) and crystallization (*i*-PrOH) to give **34** (0.32 g, 0.66 mmol) as a white solid with a 54% yield. ^1^H NMR (300 MHz, CDCl_3_) *δ* 7.96–7.87 (m, 1H, Ar-H), 7.70–7.60 (m, 1H, Ar-H), 7.46–7.36 (m, 2H, Ar-H), 7.29 (t, *J* = 54.0 Hz, 1H, CHF_2_), 6.60 (s, 1H, Ar-H), 6.30 (s, 1H, Ar-H), 4.02–3.94 (m, 4H, morph.), 3.94–3.86 (m, 4H morph. 3.79 (s, 2H, CH_2_), 3.77–3.66 (m, 1H), 2.95–2.81 (m, 2H, CH_2_), 2.40–2.25 (m, 2H, CH_2_), 1.99–1.85 (m, 2H, CH_2_), 1.74–1.55 (m, 3H). ^13^C{^1^H} NMR (75 MHz, CDCl_3_) *δ* 156.3, 151.5, 150.3, 147.7, 144.8 (t, *J* = 27.0 Hz), 142.0, 134.8, 125.8, 124.3, 121.7, 109.4 (t, *J* = 240.0 Hz, CF_2_), 96.6, 87.4, 67.9, 66.3, 56.6, 51.2, 48.7, 34.6. HRMS (ESI/MS): *m*/*z* calculated for C_24_H_27_F_2_N_7_O_2_ [M + H]^+^ 484.2267 found 484.2272.

*N*-[1-({5-[2-(difluoromethyl)-1*H*-1,3-benzimidazol-1-yl]-7-(morpholin-4-yl)pyrazolo[1,5-*a*]pyrimidin-2-yl}methyl)piperidin-4-yl]acetamide (**35**)

Compound **35** was prepared from aldehyde **15** (0.50 g, 1.23 mmol), 4-acetylamino-piperidine (0.22 g, 1.48 mmol) as an amine, DCM (5.0 mL), and sodium triacetoxyborohydride (0.40 g, 1.85 mmol), according to the general procedure for the reductive amination reaction. The crude product was purified by flash chromatography (0–15% MeOH gradient in AcOEt) and crystallization (AcOEt) to give **35** (0.51 g, 0.97 mmol) as a white solid with a 79% yield. ^1^H NMR (600 MHz, CDCl_3_) *δ* 7.92–7.91 (m, 1H, Ar-H), 7.66–7.64 (m, 1H, Ar-H), 7.44–7.39 (m, 2H, Ar-H), 7.25 (t, *J* = 53.4 Hz, 1H, CHF_2_), 6.58 (s, 1H,Ar-H), 6.31 (s, 1H, Ar-H), 5.39 (d, *J* = 8.0 Hz, 1H, NH), 3.99–3.97 (m, 4H, morph.), 3.90–3.89 (m, 4H, morph.), 3.84–3.78 (m, 1H, CH),3.76 (s, 2H, CH_2_), 2.95 (d, *J* = 11.8 Hz, 2H, CH_2_), 2.29–2.25 (m, 2H, CH_2_), 1.96 (s, 3H, CH_3_), 1.96–1.93 (m, 2H, CH_2_), 1.52–1.45 (m, 2H, CH_2_). ^13^C{^1^H, ^19^F} NMR (151 MHz, CDCl_3_) *δ* 169.3, 156.0, 151.3, 150.1, 147.6, 144.7, 141.9, 134.6, 125.7, 124.2, 121.5, 111.7, 109.3 (CF_2_), 96.4, 87.3, 66.2, 56.5, 52.5, 48.5, 46.5, 32.4, 23.5. HRMS (ESI/MS): *m*/*z* calculated for C_26_H_30_F_2_N_8_O_2_ [M + H]^+^ 525.2532 found 525.2890.

2-[4-({5-[2-(difluoromethyl)-1*H*-1,3-benzimidazol-1-yl]-7-(morpholin-4-yl)pyrazolo[1,5-*a*]pyrimidin-2-yl}methyl)piperazin-1-yl]-*N*-methylacetamide (**36**)

Compound **36** was prepared from aldehyde **15** (0.50 g, 1.23 mmol), *N*-methyl-2-(1-piperazinyl)acetamide (0.24 g, 1.48 mmol) as an amine, DCM (5.0 mL), and sodium triacetoxyborohydride (0.40 g, 1.85 mmol), according to the general procedure for the reductive amination reaction. The crude product was purified by flash chromatography (0–20% MeOH gradient in AcOEt) and crystallization (AcOEt) to give **36** (0.44 g, 0.82 mmol) as a white solid with a 66% yield. ^1^H NMR (600 MHz, CDCl_3_) *δ* 7.92–7.90 (m, 1H, Ar-H), 7.66–7.64 (m, 1H, Ar-H), 7.44–7.39 (m, 2H,Ar-H), 7.25 (t, *J* = 53.4 Hz, 1H, CHF_2_), 7.10–7.08 (m, 1H, NH), 6.59–6.58 (m, 1H, Ar-H), 6.32 (s, 1H, Ar-H), 3.99–3.98 (m, 4H, morph.), 3.91–3.89 (m, 4H, morph.), 3.80 (s, 2H, CH_2_), 3.02 (s, 2H, CH_2_), 2.83 (d, *J* = 5.1 Hz, 3H, CH_3_), 2.61 (d, *J* = 17.8 Hz, 8H). ^13^C{^1^H, ^19^F} NMR (151 MHz, CDCl_3_) *δ* 170.8, 155.6, 151.3, 150.1, 147.6, 144.6, 141.9, 134.6, 125.7, 124.2, 121.6, 111.7, 109.3 (CF_2_), 96.5, 87.3, 66.2, 61.5, 56.4, 53.6, 53.2, 48.5, 25.7. HRMS (ESI/MS): *m*/*z* calculated for C_26_H_31_F_2_N_9_O_2_ [M + H]^+^ 540.2641 found 540.2644.

2-(difluoromethyl)-1-{2-[(4-methylpiperazin-1-yl)methyl]-7-(morpholin-4-yl)pyrazolo[1,5-*a*]pyrimidin-5-yl}-1*H*-1,3-benzimidazole (**37**)

Compound **37** was prepared from aldehyde **15** (0.20 g, 0.49 mmol), 1-methylpiperazine (0.066 mL, 59.7 mg, 0.59 mmol) as an amine, DCM (2.0 mL), and sodium triacetoxyborohydride (0.17 g, 0.80 mmol), according to the general procedure for the reductive amination reaction. The crude product was purified by flash chromatography (0–30% AcOEt gradient in heptane, amino-functionalized gel column) to give **37** (0.19 g, 0.40 mmol) as a white solid with a 81% yield. ^1^H NMR (600 MHz, CDCl_3_) *δ* 7.92–7.91 (m, 1H, Ar-H), 7.66–7.64 (m, 1H, Ar-H), 7.44–7.39 (m, 2H,Ar-H), 7.29 (t, *J* = 54.0 Hz, 1H, CHF_2_), 6.60 (s, 1H, Ar-H), 6.30 (s, 1H, Ar-H), 3.99–3.98 (m, 4H, morph.), 3.91–3.89 (m, 4H, morph.), 3.79 (s, 2H, CH_2_), 2.56 (d, *J* = 85.2 Hz, 8H, piperaz.), 2.30 (s, 3H, CH_3_). ^13^C{^1^H, ^19^F} NMR (151 MHz, CDCl_3_) *δ* 155.7, 151.2, 150.0, 147.4, 144.6, 141.7, 134.5, 125.6, 124.0, 121.4, 111.6, 109.1 (CF_2_), 96.4, 87.1, 66.1, 56.3, 55.0, 53.0, 48.4, 45.9. HRMS (ESI/MS): *m*/*z* calculated for C_24_H_28_F_2_N_8_O [M + H]^+^ 483.2426 found 483.2429.

2-(difluoromethyl)-1-[7-(morpholin-4-yl)-2-{[4-(propan-2-yl)piperazin-1-yl]methyl}pyrazolo[1,5-*a*]pyrimidin-5-yl]-1*H*-1,3-benzimidazole (**38**)

Compound **38** was prepared from aldehyde **15** (1.50 g, 3.69 mmol), 1-isopropylpiperazine (0.65 mL, 0.58 g, 4.43 mmol) as an amine, DCM (15.0 mL), and sodium triacetoxyborohydride (1.25 g, 5.90 mmol), according to the general procedure for the reductive amination reaction. The crude product was purified by flash chromatography (0–30% AcOEt gradient in heptane, amino-functionalized gel column) and crystallization (AcOEt) to give **38** (1.30 g, 3.45 mmol) as a white solid with a 93% yield. ^1^H NMR (400 MHz, DMSO-*d_6_*) *δ* 7.89–7.87 (m, 1H, Ar-H), 7.82–7.80 (m, 1H, Ar-H), 7.60 (t, *J* = 52.6 Hz, 1H, CHF_2_), 7.46–7.40 (m, 2H, Ar-H), 6.65 (s, 1H, Ar-H), 6.52 (s, 1H, Ar-H), 3.94–3.92 (m, 4H, morph.), 3.84–3.82 (m, 4H, morph.), 3.66 (s, 2H, CH_2_), 2.58 (t, *J* = 6.5 Hz, 1H, CH), 2.44 (s, 8H, piperaz.), 0.94 (d, *J* = 6.5 Hz, 6H, 2xCH_3_). ^13^C{^1^H, ^19^F} NMR (101 MHz, DMSO-*d_6_*) *δ* 155.0, 150.9, 149.6, 147.0, 144.7, 141.2, 134.0, 125.4, 123.9, 120.7, 112.4, 108.5, 108.5, 95.4, 87.8, 65.6, 55.9, 53.5, 53.1, 48.2, 47.9, 18.2. HRMS (ESI/MS): *m*/*z* calculated for C_26_H_32_F_2_N_8_O [M + H]^+^ 511.2739 found 511.2743.

##### Procedure for 5-[2-(difluoromethyl)-1H-1,3-benzimidazol-1-yl]-7-(morpholin-4-yl)pyrazolo[1,5-a]pyrimidine-2-carboxylic acid (**39**)

The solution of lithium hydroxide monohydrate (28.4 g, 678 mmol) was added to the suspension of **13** (60.0 g, 136 mmol) in MeOH (1000 mL) in water (200 mL). The mixture was stirred at room temperature for 18 h. The solvent was removed under reduced pressure and the reaction was quenched with water (600 mL) and 2.5 M HCl (60 mL). The solid was collected by filtration and dried to give **39** (52.1 g, 126 mmol) as a white solid with a 93% yield. ^1^H NMR (300 MHz, DMSO-*d_6_*) *δ* 7.93–7.81 (m, 2H, Ar-H), 7.56 (t, *J =* 54.0 Hz, 1H, CHF_2_), 7.47 (ddd, *J =* 10.4, 5.2, 3.6 Hz, 2H, Ar-H), 7.03 (s, 1H), 6.86 (s, 1H), 4.02–3.96 (m, 4H, morph.), 3.90–3.77 (m, 6H), 3.57 (s, 1H).

1-[2-(4-*tert*-butylpiperazine-1-carbonyl)-7-(morpholin-4-yl)pyrazolo[1,5-*a*]pyrimidin-5-yl]-2-(difluoromethyl)-1*H*-1,3-benzimidazole (**40**)

Compound **40** was prepared from **39** (0.20 g, 0.48 mmol), *N*-*t*-butylpiperazine (0.10 g, 0.72 mmol), HATU (0.21 g, 0.57 mmol), TEA (0.13 mL, 96.5 mg, 0.75 mmol), and DMF (2.0 mL), according to the general procedure for the amidation reaction. The crude product was purified by flash chromatography (0–100% AcOEt gradient in heptane, amino-functionalized gel column) to give **40** (0.13 g, 0.23 mmol) as a white solid with a 51% yield. ^1^H NMR (400 MHz, DMSO-*d_6_*) *δ* 7.88 (dd, *J* = 7.1, 1.5 Hz, 1H, Ar-H), 7.84–7.82 (m, 1H, Ar-H), 7.61 (t, *J* = 52.5 Hz, 1H, CHF_2_), 7.48–7.41 (m, 2H, Ar-H), 6.83 (s, 1H, Ar-H), 6.80 (s, 1H, Ar-H), 3.93–3.91 (m, 4H), 3.86–3.83 (m, 4H), 3.72–3.64 (m, 4H), 2.59–2.51 (m, 4H), 1.02 (s, 9H, t-Bu). ^13^C{^1^H, ^19^F} NMR (101 MHz, DMSO-*d_6_*) *δ* 161.5, 151.2, 150.4, 149.2, 147.8, 144.7, 141.2, 134.0, 125.6, 124.1, 120.7, 112.4, 108.5, 108.5, 96.7, 89.3, 65.6, 59.7, 48.5, 46.1, 25.6, 20.7, 14.1. HRMS (ESI/MS): *m*/*z* calculated for C_27_H_32_F_2_N_8_O_2_ [M + H]^+^ 539.2689 found 539.2736.

2-(4-{5-[2-(difluoromethyl)-1*H*-1,3-benzimidazol-1-yl]-7-(morpholin-4-yl)pyrazolo[1,5-*a*]pyrimidine-2-carbonyl}piperazin-1-yl)propan-2-ol (**41**)

Compound **41** was prepared from **39** (1.50 g, 3.62 mmol), 2-(4-piperidyl)-2-propanol (0.57 g, 3.83 mmol), HATU (1.51 mg, 3.98 mmol), TEA (0.76 mL, 0.55, 5.44 mmol), and DCM (15.0 mL), according to the general procedure for the amidation reaction. The crude product was purified by flash chromatography (0–10% MeOH gradient in AcOEt) to give **41** (0.66 g, 1.22 mmol) as a light yellow solid with a 34% yield. ^1^H NMR (300 MHz, CDCl_3_) *δ* 7.94–7.89 (m, 1H, Ar-H), 7.69–7.65 (m, 1H, Ar-H), 7.46–7.39 (m, 2H, Ar-H), 7.29 (t, *J =* 54.0 Hz, 1H, CHF_2_), 6.86 (s, 1H, Ar-H), 6.42 (s, 1H, Ar-H), 4.91 (d, *J =* 13.0 Hz, 1H), 4.49 (d, *J =* 13.5 Hz, 1H), 4.00–3.95 (m, 4H, morph.), 3.94–3.86 (m, 4H, morph.), 3.08 (t, *J =* 11.8 Hz, 1H), 2.76 (td, *J =* 12.8, 2.6 Hz, 1H), 1.97–1.78 (m, 2H, CH_2_), 1.68–1.56 (m, 1H, CH), 1.49–1.34 (m, 3H), 1.22 (d, *J =* 6.0 Hz, 6H, 2xCH_3_). ^13^C{^1^H} NMR (75 MHz, CDCl_3_) *δ* 162.7, 151.6, 149.7, 148.5, 144.7(t, *J* = 27.0 Hz), 142.0, 134.7, 126.0, 124.5, 121.7, 111.9 (t, *J* = 238.5 Hz, CF_2_), 97.8, 88.6, 72.2, 66.3, 48.9, 47.8, 43.2, 27.6 (d, *J* = 16.8 Hz), 26.9 (d, *J* = 5.1 Hz). HRMS (ESI/MS): *m*/*z* calculated for C_27_H_31_F_2_N_7_O_3_ [M + H]^+^ 540.2529 found 540.2533.

2-(4-{5-[2-(difluoromethyl)-1*H*-1,3-benzimidazol-1-yl]-7-(morpholin-4-yl)pyrazolo[1,5-*a*]pyrimidine-2-carbonyl}piperazin-1-yl)-2-methylpropanamide (**42**)

Compound **42** was prepared from **39** (0.20 g, 0.48 mmol), 2-methyl-2-(piperazin-1-yl)propanamide dihydrochloride (0.13 g, 0.49 mmol), HATU (0.20 g, 0.52 mmol), TEA (0.23 mL, 0.16 g, 1.66 mmol), and DCM (2.0 mL), according to the general procedure for the amidation reaction. The crude product was purified by flash chromatography (0–20% MeOH gradient in AcOEt) to give **42** (0.18 g, 0.31 mmol) as a white solid with a 67% yield. ^1^H NMR (600 MHz, DMSO-*d_6_*) *δ* 7.89 (d, *J* = 7.6 Hz, 1H, Ar-H), 7.82 (d, *J* = 7.9 Hz, 1H, Ar-H), 7.58 (t, *J* = 52.6 Hz, 1H, CHF_2_), 7.49–7.42 (m, 2H), 7.25 (d, *J* = 2.9 Hz, 1H), 6.99 (d, *J* = 2.8 Hz, 1H, Ar-H), 6.84 (s, 1H, Ar-H), 6.80 (s, 1H), 3.93–3.91 (m, 4H, morph.), 3.85–3.84 (m, 4H, morph.), 3.79 (s, 2H, CH_2_), 3.73 (s, 2H, CH_2_), 2.52–2.50 (m, 2H, CH_2_), 2.46 (t, *J* = 4.4 Hz, 2H, CH_2_), 1.09 (s, 6H, 2xCH_3_). ^13^C{^1^H, ^19^F}NMR (151 MHz, DMSO-*d6*) *δ* 177.7, 161.6, 151.1, 150.3, 149.2, 147.7, 144.6, 141.2, 134.0, 125.5, 124.0, 120.6, 112.3, 108.5, 96.6, 89.2, 79.1, 65.5, 62.6, 48.4, 47.1, 46.4, 42.2, 20.5. HRMS (ESI/MS): *m*/*z* calculated for C_27_H_31_F_2_N_9_O_3_ [M + H]^+^ 568.2590 found 568.2586.

1-[2-(4-cyclopropanecarbonylpiperazine-1-carbonyl)-7-(morpholin-4-yl)pyrazolo[1,5-*a*]pyrimidin-5-yl]-2-(difluoromethyl)-1*H*-1,3-benzimidazole (**43**)

Compound **43** was prepared from **39** (0.30 g, 0.71 mmol), 1-(cyclopropylcarbonyl)piperazine (0.11 mL, 0.11 g, 0.74 mmol), HATU (0.30 g, 0.78 mmol), TEA (0.15 mL, 100 mg, 1.06 mmol), and DCM (3.0 mL), according to the general procedure for the amidation reaction. The crude product was purified by flash chromatography (0–10% MeOH gradient in AcOEt) to give **43** (0.24 g, 0.44 mmol) as a white solid with a 62% yield. ^1^H NMR (600 MHz, DMSO-*d_6_*) *δ* 7.89–7.88 (m, 1H, Ar-H), 7.83 (d, *J* = 7.8 Hz, 1H, Ar-H), 7.59 (t, *J* = 52.5 Hz, 1H, CHF_2_), 7.49–7.43 (m, 2H, Ar-H), 6.89 (s, 1H, Ar-H), 6.82 (s, 1H, Ar-H), 3.92 (d, *J* = 4.5 Hz, 4H), 3.85 (s, 4H), 3.78 (s, 4H), 3.67–3.56 (m, 3H), 1.98 (s, 1H, CH), 1.16 (d, *J* = 7.1 Hz, 1H), 0.77–0.73 (m, 4H). ^13^C{^1^H, ^19^F} NMR (151 MHz, DMSO-*d_6_*) *δ* 171.3, 151.2, 150.1, 149.3, 147.8, 144.7, 141.2, 134.0, 125.6, 124.1, 120.7, 112.4, 108.5, 97.0, 89.4, 65.6, 48.5, 45.8, 10.4, 7.1. HRMS (ESI/MS): *m*/*z* calculated for C_27_H_28_F_2_N_8_O_3_ [M + H]^+^ 551.2332 found 551.2333.

(1-{5-[2-(difluoromethyl)-1*H*-1,3-benzodiazol-1-yl]-7-(morpholin-4-yl)pyrazolo[1,5-*a*]pyrimidine-2-carbonyl}pyrrolidin-2-yl)methanol (**44**)

Compound **44** was prepared from **39** (0.50 g, 1.18 mmol), pyrrolidin-2-ylmethanol (0.13 mL, 0.13 g, 1.31 mmol), HATU (0.49 g, 1.30 mmol), TEA (0.25 mL, 0.18 g, 1.79 mmol), and DCM (5.0 mL), according to the general procedure for the amidation reaction. The crude product was purified by flash chromatography (0–10% MeOH gradient in AcOEt) to give **44** (0.16 g, 0.33 mmol) as a white solid with a 28% yield. ^1^H NMR (600 MHz, DMSO-*d_6_*) *δ* 7.89 (d, *J* = 7.8 Hz, 1H, Ar-H), 7.85–7.83 (m, 1H,Ar-H), 7.60 (t, *J* = 52.3 Hz, 1H, CHF_2_), 7.49–7.42 (m, 2H, Ar-H), 6.92–6.90 (m, 1HAr-H) 6.81 (s, 1H, Ar-H), 4.85–4.81 (m, 1H, OH), 4.24–4.17 (m, 1H), 3.96–3.90 (m, 4H, morph.), 3.87–3.83 (m, 4H, morph.), 3.68–3.59 (m, 1H), 3.54–3.45 (m, 1H), 3.37–3.35 (m, 2H, CH_2_), 2.09–1.82 (m, 4H). ^13^C{^1^H, ^19^F} NMR (151 MHz, DMSO-*d_6_*) *δ* 161.0, 160.7, 151.2, 149.0, 144.7, 141.2, 134.0, 125.6, 124.1, 120.7, 112.4, 108.5, 97.3, 89.2, 65.7, 60.8, 59.7, 59.1, 49.1, 48.5, 26.3, 24.2. HRMS (ESI/MS): *m*/*z* calculated for C_24_H_25_F_2_N_7_O_3_ [M + H]^+^ 498.2059 found 498.2066.

1-{5-[2-(difluoromethyl)-1*H*-1,3-benzodiazol-1-yl]-7-(morpholin-4-yl)pyrazolo[1,5-*a*]pyrimidine-2-carbonyl}pyrrolidin-3-amine (**45**)

Compound *N*-(1-{5-[2-(difluoromethyl)-1*H*-1,3-benzodiazol-1-yl]-7-(morpholin-4-yl)pyrazolo[1,5-*a*]pyrimidine-2-carbonyl}pyrrolidin-3-yl)carbamate (**Boc-45**) was prepared from **39** (0.50 g, 1.18 mmol), 3-(Boc-amino)pyrrolidine (0.24 g, 1.30 mmol), HATU (0.49 g, 1.30 mmol), TEA (0.25 mL, 0.18 g, 1.79 mmol), and DCM (5.0 mL), according to the general procedure for the amidation reaction. The crude product was purified by flash chromatography (0–10% MeOH gradient in AcOEt) to give **Boc-45** (0.37 g, 0.64 mmol) as a light yellow solid with a 55% yield. ^1^H NMR (300 MHz, DMSO-*d_6_*) *δ* 7.93–7.82 (m, 2H, Ar-H), 7.56 (t, *J* = 54.0 Hz, 1H, CHF_2_), 7.52–7.41 (m, 2H, Ar-H), 6.92 (d, *J* = 0.6 Hz, 1H, Ar-H), 6.82 (s, 1H, Ar-H), 4.87–4.79 (m, 1H), 4.71–4.16 (m, 1H), 3.99–3.77 (m, 11H), 3.71–3.58 (m, 1H), 3.58–3.32 (m, 2H), 2.14–1.79 (m, 9H, t-Bu).

The solution of **Boc-45** (0.35 g, 0.60 mmol) in trifluoroacetic acid (2.09 mL, 3.11 g, 27.0 mmol, 45.0 eq) was heated at 50 °C for 3 h. The reaction was then cooled to room temperature, and stopped with 15% NaOH (15.0 mL). The aqueous mixture was extracted with DCM (3 × 15 mL). The combined organic extracts were washed with water and dried over Na_2_SO_4_, filtered, and concentrated. The crude product was purified by flash chromatography (50–100% AcOEt gradient in heptane, amino-functionalized gel column) to give **45** (0.14 g, 0.29 mmol) as a white solid with a 48% yield. ^1^H NMR (600 MHz, DMSO-*d_6_*) *δ* 7.89 (d, *J* = 7.6 Hz, 1H, Ar-H), 7.84–7.83 (m, 1H, Ar-H), 7.60 (t, *J* = 52.5 Hz, 1H, CHF_2_), 7.47–7.42 (m, 2H,Ar-H), 6.91 (d, *J* = 3.5 Hz, 1H, Ar-H), 6.80 (s, 1H, Ar-H), 4.00–3.93 (m, 5H), 3.90–3.83 (m, 5H), 3.71–3.66 (m, 1H), 3.62 (dd, *J* = 12.2, 5.8 Hz, 1H), 3.55–3.48 (m, 2H), 3.24–3.15 (m, 1H, CH), 2.03–1.94 (m, 1H, CH), 1.73–1.61 (m, 1H, CH). ^13^C{^1^H, ^19^F} NMR (151 MHz, DMSO-*d_6_*) *δ* 160.5, 151.1, 148.9, 147.6, 144.6, 141.1, 133.9, 125.4, 123.9, 120.6, 112.3, 108.4, 97.1, 89.1, 65.5, 56.7, 54.8, 51.3, 48.9, 46.7, 45.0, 34.7, 32.0. HRMS (ESI/MS): *m*/*z* calculated for C_23_H_24_F_2_N_8_O_2_ [M + H]^+^ 483.2063 found 483.2063.

2-(difluoromethyl)-1-[7-(morpholin-4-yl)-2-(morpholine-4-carbonyl)pyrazolo[1,5-*a*]pyrimidin-5-yl]-1*H*-1,3-benzimidazole (**46**)

Compound **46** was prepared from **39** (1.50 g, 3.62 mmol), morpholine (0.34 mL, 0.34 g, 3.90 mmol), HATU (1.52 mg, 3.99 mmol), TEA (0.76 mL, 0.55 g, 5.44 mmol), and DCM (15.0 mL), according to the general procedure for the amidation reaction. The crude product was purified by flash chromatography (50–100% AcOEt gradient in heptane) to give **46** (937.0 mg, 1.94 mmol) as a light yellow solid with a 53% yield. ^1^H NMR (600 MHz, CDCl_3_) *δ* 7.93–7.91 (m, 1H, Ar-H), 7.68–7.66 (m, 1H, Ar-H), 7.46–7.41 (m, 2H, Ar-H), 7.28 (t, *J* = 54.0 Hz, 1H, CHF_2_), 6.91 (s, 1H, Ar-H), 6.45 (s, 1H, Ar-H), 3.98–3.96 (m, 4H, morph.), 3.92–3.86 (m, 8H, morph.), 3.84–3.82 (m, 2H, morph.), 3.73–3.72 (m, 2H, morph.). ^13^C{^1^H, ^19^F} NMR (151 MHz, CDCl_3_) *δ* 162.9, 151.7, 150.9, 149.9, 148.7, 144.7, 142.1, 134.7, 126.2, 124.6, 121.8, 112.0, 109.7 (CF_2_), 98.4, 89.0, 67.2, 66.4, 48.9, 43.1. HRMS (ESI/MS): *m*/*z* calculated for C_23_H_23_F_2_N_7_O_3_ [M + H]^+^ 484.1903 found 484.1912.

2-(difluoromethyl)-1-[2-(4,4-difluoropiperidine-1-carbonyl)-7-(morpholin-4-yl)pyrazolo[1,5-*a*]pyrimidin-5-yl]-1*H*-1,3-benzimidazole (**47**)

Compound **47** was prepared from **39** (1.50 g, 3.62 mmol), 4,4-difluoropiperidine (0.63 g, 3.99 mmol), HATU (1.52 mg, 3.99 mmol), TEA (1.31 mL, 0.95 g, 9.35 mmol), and DCM (15.0 mL), according to the general procedure for the amidation reaction. The crude product was purified by flash chromatography (0–50% AcOEt gradient in heptane, amino-functionalized gel column) to give **47** (934.0 mg, 1.80 mmol) as a light yellow solid with a 50% yield. ^1^H NMR (300 MHz, CDCl_3_) *δ* 7.96–7.88 (m, 1H, Ar-H), 7.71–7.66 (m, 1H, Ar-H), 7.48–7.41 (m, 2H, Ar-H), 7.29 (t, *J =* 54.0 Hz, 1H, CHF_2_), 6.93 (s, 1H, Ar-H), 6.47 (s, 1H, Ar-H), 4.03–3.95 (m, 8H), 3.93–3.87 (m, 4H, morph.), 2.22–1.98 (m, 4H). ^13^C{^1^H} NMR (75 MHz, CDCl_3_) *δ* 163.3, 152.1, 150.3, 149.1, 145.1 (t, *J* = 27.0 Hz), 142.5, 135.2, 126.5, 125.0, 122.0 (t, *J* = 240.7 Hz), 112.4, 110.1 (t, *J* = 238.5 Hz), 98.9, 89.6, 66.7, 49.3, 44.4, 40.1, 35.6 (t, *J* = 24.7 Hz), 34.5 (t, *J* = 22.5 Hz). HRMS (ESI/MS): *m*/*z* calculated for C_24_H_23_F_4_N_7_O_2_ [M + H]^+^ 518.1922 found 518.1924.

1-{5-[2-(difluoromethyl)-1*H*-1,3-benzimidazol-1-yl]-7-(morpholin-4-yl)pyrazolo[1,5-*a*]pyrimidine-2-carbonyl}piperidine-4-carboxamide (**48**)

Compound **48** was prepared from **39** (1.50 g, 3.62 mmol), 4-piperidinecarboxamide (0.51 g, 3.90 mmol), HATU (1.52 mg, 3.99 mmol), TEA (0.76 mL, 0.55 g, 5.44 mmol), and DCM (15.0 mL), according to the general procedure for the amidation reaction. The crude product was purified by flash chromatography (0–20% MeOH gradient in AcOEt) to give **48** (0.73 g, 1.39 mmol) as a light yellow solid with a 39% yield. ^1^H NMR (600 MHz, DMSO-*d_6_*) *δ* 7.89–7.88 (m, 1H, Ar-H), 7.84–7.82 (m, 1H, Ar-H), 7.59 (t, *J* = 52.5 Hz, 1H, CHF_2_), 7.49–7.42 (m, 2H), 7.31 (s, 1H, Ar-H), 6.83 (s, 1H, Ar-H), 6.82 (s, 1H), 6.80 (s, 1H), 4.48 (d, *J* = 13.0 Hz, 2H, CH_2_), 4.25 (d, *J* = 13.6 Hz, 2H, CH_2_), 3.93–3.91 (m, 4H, morph.), 3.84 (t, *J* = 4.7 Hz, 4H, morph.), 3.18 (s, 2H), 2.90 (d, *J* = 2.5 Hz, 2H), 2.43 (s, 1H). ^13^C{^1^H, ^19^F} NMR (151 MHz, DMSO-*d_6_*) *δ* 175.8, 161.8, 151.2, 150.6, 149.2, 147.7, 144.7, 141.2, 134.0, 125.5, 124.0, 120.7, 112.4, 108.5, 96.4, 89.3, 65.6, 48.4, 46.2, 41.4, 29.0, 28.2. HRMS (ESI/MS): *m*/*z* calculated for C_25_H_26_F_2_N_8_O_3_ [M + H]^+^ 525.2168 found 525.2171.

1-{5-[2-(difluoromethyl)-1*H*-1,3-benzimidazol-1-yl]-7-(morpholin-4-yl)pyrazolo[1,5-*a*]pyrimidine-2-carbonyl}-4-methylpiperidin-4-ol (**49**)

Compound **49** was prepared from **39** (1.50 g, 3.62 mmol), 4-methylpiperidin-4-ol (0.47 g, 3.89 mmol), HATU (1.52 mg, 3.99 mmol), TEA (0.76 mL, 0.55 g, 5.44 mmol), and DCM (15.0 mL), according to the general procedure for the amidation reaction. The crude product was purified by flash chromatography (0–10% MeOH gradient in AcOEt) to give **49** (0.90 g, 1.76 mmol) as a light yellow solid with a 49% yield. ^1^H NMR (600 MHz, CDCl_3_) *δ* 7.93–7.91 (m, 1H, Ar-H), 7.67 (dd, *J* = 7.4, 1.8 Hz, 1H,Ar-H), 7.46–7.41 (m, 2H, Ar-H), 7.29 (t, *J* = 54.0 Hz, 1H, CHF_2_), 6.87 (s, 1H, Ar-H), 6.42 (s, 1H, Ar-H), 4.45–4.41 (m, 1H), 4.12–4.09 (m, 1H), 3.98–3.96 (m, 4H, morph.) 3.91–3.90 (m, 4H, morph.), 3.65–3.61 (m, 1H), 3.42–3.38 (m, 1H), 1.78–1.71 (m, 2H, CH_2_), 1.68–1.62 (m, 3H, CH_3_), 1.36 (s, 1H), 1.34 (s, 3H). ^13^C{^1^H, ^19^F} NMR (151 MHz, CDCl_3_) *δ* 162.8, 151.7, 149.8, 148.5, 144.8, 142.1, 134.8, 126.1, 124.5, 121.8, 112.0, 109.7 (CF_2_), 97.9, 88.8, 68.4, 66.4, 48.9, 43.8, 39.5, 39.1, 38.6, 30.6. HRMS (ESI/MS): *m*/*z* calculated for C_25_H_27_F_2_N_7_O_3_ [M + H]^+^ 512.2216 found 512.2222.

(1-{5-[2-(difluoromethyl)-1*H*-1,3-benzimidazol-1-yl]-7-(morpholin-4-yl)pyrazolo[1,5-*a*]pyrimidine-2-carbonyl}piperidin-4-yl)methanol (**50**)

Compound **50** was prepared from **39** (1.50 g, 3.62 mmol), 4-piperidinemethanol (0.46 g, 3.95 mmol), HATU (1.52 mg, 3.99 mmol), TEA (0.76 mL, 0.55 g, 5.44 mmol), and DCM (15.0 mL), according to the general procedure for the amidation reaction. The crude product was purified by flash chromatography (0–10% MeOH gradient in AcOEt) to give **50** (0.76 g, 1.54 mmol) as a light yellow solid with a 43% yield. ^1^H NMR (600 MHz, DMSO-*d_6_*) *δ* 7.90–7.87 (m, 1H, Ar-H), 7.83 (dd, *J* = 7.0, 1.3 Hz, 1H, Ar-H), 7.61 (t, *J* = 52.5 Hz, 1H, CHF_2_), 7.49–7.41 (m, 2H, Ar-H), 6.81 (s, 1H, Ar-H), 6.80 (s, 1H), 4.53 (t, *J* = 5.3 Hz, 2H, CH_2_), 4.24 (d, *J* = 13.3 Hz, 1H), 3.92–3.91 (m, 4H, morph.), 3.85–3.83 (m, 5H), 3.30–3.28 (m, 2H), 3.15–3.08 (m, 1H), 2.85–2.78 (m, 1H), 1.78 (d, *J* = 13.1 Hz, 1H), 1.70 (d, *J* = 10.4 Hz, 2H), 1.12–1.09 (m, 1H). ^13^C{^1^H, ^19^F} NMR (151 MHz, DMSO-*d_6_*) *δ* 170.6, 161.9, 151.4, 149.5, 148.0, 144.9, 141.4, 134.2, 125.8, 124.3, 120.9, 112.7, 108.7, 96.6, 89.4, 65.8, 60.0, 49.0, 46.9, 42.0, 38.6, 29.5, 28.7. HRMS (ESI/MS): *m*/*z* calculated for C_25_H_27_F_2_N_7_O_3_ [M + H]^+^ 512.2216 found 512.2218.

1-{5-[2-(difluoromethyl)-1*H*-1,3-benzimidazol-1-yl]-7-(morpholin-4-yl)pyrazolo[1,5-*a*]pyrimidine-2-carbonyl}piperidine-4-carbonitrile (**51**)

Compound **51** was prepared from **39** (1.50 g, 3.62 mmol), 4-cyanopiperidine (0.44 mL, 0.43 g, 3.96 mmol), HATU (1.52 mg, 3.99 mmol), TEA (0.76 mL, 0.55 g, 5.44 mmol), and DCM (15.0 mL), according to the general procedure for the amidation reaction. The crude product was purified by flash chromatography (50–100% AcOEt gradient in heptane) to give **51** (0.86 g, 1.69 mmol) as a light yellow solid with a 47% yield. ^1^H NMR (400 MHz, CDCl_3_) *δ* 7.93–7.91 (m, 1H, Ar-H), 7.69–7.66 (m, 1H, Ar-H), 7.47–7.40 (m, 2H, Ar-H), 7.28 (t, *J* = 54.0 Hz, 1H, CHF_2_), 6.90 (s, 1H, Ar-H), 6.46 (s, 1H, Ar-H), 4.08–3.97 (m, 6H), 3.91–3.83 (m, 6H), 3.02–2.98 (m, 1H, CH), 2.08–1.92 (m, 4H). ^13^C{^1^H, ^19^F} NMR (101 MHz, CDCl_3_) *δ* 162.6, 151.5, 150.6, 149.7, 148.5, 144.5, 141.8, 134.5, 126.0, 124.4, 121.6, 120.6, 111.8, 109.5 (CF_2_), 98.1, 88.9, 66.1, 48.7, 45.1, 40.4, 29.3, 28.3, 26.4. HRMS (ESI/MS): *m*/*z* calculated for C_25_H_24_F_2_N_8_O_2_ [M + H]^+^ 507.2063 found 507.2068.

2-(difluoromethyl)-1-[7-(morpholin-4-yl)-2-[4-(morpholin-4-yl)piperidine-1-carbonyl]pyrazolo[1,5-*a*]pyrimidin-5-yl]-1*H*-1,3-benzimidazole (**52**)

Compound **52** was prepared from **39** (1.50 g, 3.62 mmol), 4-morpholinopiperidine (0.68 g, 3.90 mmol), HATU (1.52 mg, 3.99 mmol), TEA (0.76 mL, 0.55 g, 5.44 mmol), and DCM (15.0 mL), according to the general procedure for the amidation reaction. The crude product was purified by flash chromatography (0–20% MeOH gradient in AcOEt) to give **52** (0.70 g, 1.23 mmol) as a light yellow solid with a 34% yield. ^1^H NMR (400 MHz, DMSO-*d_6_*) *δ* 7.90–7.88 (m, 1H, Ar-H), 7.83 (dd, *J* = 7.2, 1.3 Hz, 1H, Ar-H), 7.61 (t, *J* = 52.5 Hz, 1H, CHF_2_), 7.49–7.44 (m, 2H), 6.85 (s, 1H, Ar-H), 6.82 (s, 1H, Ar-H), 4.21–4.65 (m, 2H, CH_2_), 3.92 (d, *J* = 3.3 Hz, 5H), 3.86–3.84 (m, 5H), 3.66–3.61 (m, 4H, morph.), 3.15–3.07 (m, 4H, morph.), 2.86 (d, *J* = 1.2 Hz, 1H, CH), 1.78–2.06 (m, 2H, CH_2_), 1.37–1.57 (m, 2H, CH_2_). ^13^C{^1^H, ^19^F} NMR (101 MHz, DMSO-*d_6_*) *δ* 161.6, 151.2, 149.2, 147.8, 144.7, 141.2, 134.0, 125.5, 124.1, 120.7, 112.4, 108.5, 108.5, 89.3, 65.6, 48.7, 48.4, 45.7, 26.8, 8.6. HRMS (ESI/MS): *m*/*z* calculated for C_28_H_32_F_2_N_8_O_3_ [M + H]^+^ 567.2638 found 567.2643.

1-{5-[2-(difluoromethyl)-1*H*-1,3-benzimidazol-1-yl]-7-(morpholin-4-yl)pyrazolo[1,5-*a*]pyrimidine-2-carbonyl}piperidin-4-ol (**53**)

Compound **53** was prepared from **39** (1.50 g, 3.62 mmol), 4-hydroxypiperidine (0.41 g, 4.06 mmol), HATU (1.52 mg, 3.99 mmol), TEA (0.76 mL, 0.55 g, 5.44 mmol), and DCM (15.0 mL), according to the general procedure for the amidation reaction. The crude product was purified by flash chromatography (0–15% MeOH gradient in AcOEt) to give **53** (0.86 g, 1.72 mmol) as a light yellow solid with a 48% yield. ^1^H NMR (600 MHz, DMSO-*d_6_*) *δ* 7.90–7.88 (m, 1H, Ar-H), 7.84–7.82 (m, 1H, Ar-H), 7.59 (t, *J*=52.5 Hz, 1H, CHF_2_), 7.49–7.43 (m, 2H, Ar-H), 6.83 (s, 1H, Ar-H), 6.81 (s, 1H, Ar-H), 4.83 (d, *J* = 4.0 Hz, 1H, OH), 4.08–4.03 (m, 2H, CH_2_), 4.02–3.97 (m, 2H, CH_2_), 3.93–3.92 (m, 4H, morph.), 3.85–3.84 (m, 4H, morph.), 3.81–3.77 (m, 1H, CH), 3.44–3.37 (m, 2H, CH_2_), 3.32–3.28 (m, 2H, CH_2_). ^13^C{^1^H, ^19^F} NMR (151 MHz, DMSO-*d_6_*) *δ* 162.0, 151.5, 150.9, 149.5, 148.0, 145.0, 141.5, 134.3, 125.8, 124.3, 121.0, 112.7, 108.8, 96.7, 89.5, 65.9, 65.7, 48.7, 44.3, 35.0, 34.2. HRMS (ESI/MS): *m*/*z* calculated for C_24_H_25_F_2_N_7_O_3_ [M + H]^+^ 498.2059 found 498.2060.

*N*-(1-{5-[2-(difluoromethyl)-1*H*-1,3-benzimidazol-1-yl]-7-(morpholin-4-yl)pyrazolo[1,5-*a*]pyrimidine-2-carbonyl}piperidin-4-yl)acetamide (**54**)

Compound **54** was prepared from **39** (1.50 g, 3.62 mmol), 4-acetylamino-piperidine (0.57 g, 3.89 mmol), HATU (1.52 mg, 3.99 mmol), TEA (0.76 mL, 0.55 g, 5.44 mmol), and DCM (15.0 mL), according to the general procedure for amidation reaction. The crude product was purified by flash chromatography (0–20% MeOH gradient in AcOEt) to give **54** (0.80 g, 1.48 mmol) as a light yellow solid with a 41% yield. ^1^H NMR (600 MHz, CDCl_3_) *δ* 7.93–7.91 (m, 1H, Ar-H), 7.68–7.66 (m, 1H, Ar-H), 7.46–7.42 (m, 2H, Ar-H), 7.29 (t, *J* = 54.0 Hz, 1H, CHF_2_), 6.87 (s, 1H, Ar-H), 6.44 (s, 1H, Ar-H), 5.55 (d, *J* = 7.8 Hz, 1H, NH), 4.74 (d, *J* = 13.7 Hz, 2H, CH_2_), 4.43 (d, *J* = 15.1 Hz, 2H CH_2_ 4.13–4.07 (m, 1H, CH), 3.98–3.96 (m, 4H, morph.), 3.91–3.89 (m, 4H, morph.), 3.31–3.27 (m, 1H), 3.03–2.99 (m, 1H), 2.10–2.05 (m, 1H), 2.00 (s, 3H, CH_3_), 1.55–1.40 (m, 1H). ^13^C{^1^H, ^19^F} NMR (151 MHz, CDCl_3_) *δ* 169.3, 162.6, 151.4, 150.9, 149.5, 148.3, 144.4, 141.7, 134.4, 125.8, 124.2, 121.5, 111.7, 109.4 (CF_2_), 97.8, 88.6, 66.1, 48.6, 46.7, 45.9, 41.4, 32.9, 31.7, 23.3. HRMS (ESI/MS): *m*/*z* calculated for C_26_H_28_F_2_N_8_O_3_ [M + H]^+^ 539.2352 found 539.2325.

2-(4-{5-[2-(difluoromethyl)-1*H*-1,3-benzimidazol-1-yl]-7-(morpholin-4-yl)pyrazolo[1,5-*a*]pyrimidine-2-carbonyl}piperazin-1-yl)-*N*-methylacetamide (**55**)

Compound **55** was prepared from **39** (1.50 g, 3.62 mmol), *N*-methyl-2-(1-piperazinyl)acetamide (0.65 g, 3.97 mmol), HATU (1.52 mg, 3.99 mmol), TEA (0.76 mL, 0.55 g, 5.44 mmol), and DCM (15.0 mL), according to the general procedure for the amidation reaction. The crude product was purified by flash chromatography (0–15% MeOH gradient in AcOEt) to give **55** (0.66 g, 1.18 mmol) as a light yellow solid with a 33% yield. ^1^H NMR (600 MHz, DMSO-*d_6_*) *δ* 7.89–7.88 (m, 1H, Ar-H), 7.83–7.82 (m, 1H, Ar-H), 7.79 (s, 1H, Ar-H), 7.59 (t, *J* = 52.5 Hz, 1H, CHF_2_), 7.49–7.42 (m, 2H, Ar-H), 6.84 (s, 1H, Ar-H), 6.81 (s, 1H, Ar-H), 3.92–3.91 (m, 4H, morph.), 3.85–3.83 (m, 4H), 3.80–3.73 (m, 4H), 2.98 (s, 2H), 2.62 (d, *J* = 4.7 Hz, 3H, CH_3_), 2.54 (s, 2H, CH_2_), 1.09 (s, 2H, CH_2_). ^13^C{^1^H, ^19^F} NMR (151 MHz, DMSO-*d_6_*) *δ* 161.6, 151.0, 150.1, 149.1, 147.6, 144.5, 141.1, 133.9, 125.4, 123.9, 120.6, 112.3, 108.4, 96.6, 89.2, 71.9, 65.4, 53.0, 52.4, 48.6, 48.3, 45.7, 26.6, 25.2. HRMS (ESI/MS): *m*/*z* calculated for C_26_H_29_F_2_N_9_O_3_ [M + H]^+^ 554.2434 found 554.2434.

2-(difluoromethyl)-1-[2-(4-methylpiperazine-1-carbonyl)-7-(morpholin-4-yl)pyrazolo[1,5-*a*]pyrimidin-5-yl]-1*H*-1,3-benzimidazole (**56**)

Compound **56** was prepared from **39** (0.20 g, 0.48 mmol), 1-methylpiperazine (0.05 mL, 50.2 mg, 0.49 mmol), HATU (0.20 g, 0.52 mmol), TEA (0.09 mL, 72.2 mg, 0.71 mmol), and DCM (2.0 mL), according to the general procedure for the amidation reaction. The crude product was purified by flash chromatography (0–35% MeOH gradient in AcOEt) to give **56** (0.13 g, 0.27 mmol) as a white solid with a 57% yield. ^1^H NMR (600 MHz, DMSO-*d_6_*) *δ* 7.89 (m, 1H, Ar-H), 7.83–7.81 (m, 1H, Ar-H), 7.58 (t, *J* = 52.4 Hz, 1H, CHF_2_), 7.49–7.42 (m, 2H, Ar-H), 6.83 (s, 1H, Ar-H), 6.81 (s, 1H, Ar-H), 3.92 (t, *J* = 4.7 Hz, 4H, morph.), 3.84 (t, *J* = 4.7 Hz, 4H, morhp.), 3.76–3.69 (m, 4H, morph.), 2.43–2.37 (m, 4H, morph.), 2.23 (s, 3H, CH_3_). ^13^C{^1^H, ^19^F} NMR (151 MHz, DMSO-*d_6_*) *δ* 161.7, 151.1, 150.3, 149.2, 147.7, 144.6, 141.2, 134.0, 125.5, 124.0, 120.6, 112.3, 108.5, 96.6, 89.2, 65.5, 54.8, 48.4, 46.5, 45.5, 41.7, 30.8. HRMS (ESI/MS): *m*/*z* calculated for C_24_H_26_F_2_N_8_O_2_ [M + H]^+^ 497.2219 found 497.2229.

2-(difluoromethyl)-1-[7-(morpholin-4-yl)-2-[4-(propan-2-yl)piperazine-1-carbonyl]pyrazolo[1,5-*a*]pyrimidin-5-yl]-1*H*-1,3-benzimidazole (**57**)

Compound **57** was prepared from **39** (0.20 g, 0.48 mmol), 1-isopropylpiperazine (0.07 mL, 65.7 mg, 0.49 mmol), HATU (0.20 g, 0.52 mmol), TEA (0.09 mL, 72.2 mg, 0.71 mmol), and DCM (2.0 mL), according to the general procedure for the amidation reaction. The crude product was purified by flash chromatography (0–30% MeOH gradient in AcOEt) to give **57** (0.20 g, 0.38 mmol) as a white solid with a 81% yield. ^1^H NMR (600 MHz, DMSO-*d_6_*) *δ* 7.89–7.88 (m, 1H, Ar-H), 7.83–7.82 (m, 1H, Ar-H), 7.58 (t, *J* = 52.5 Hz, 1H, CHF_2_), 7.49–7.42 (m, 2H, Ar-H), 6.83 (s, 1H, Ar-H), 6.80 (s, 1H, Ar-H), 3.93–3.91 (m, 4H, morph., morph.), 3.85–3.84 (m, 4H, morph.), 3.73–3.71 (m, 2H, CH_2_), 3.67–3.66 (m, 2H, CH_2_), 2.72–2.68 (m, 1H, CH), 2.53–2.50 (m, 2H, CH_2_), 2.47 (d, *J* = 4.8 Hz, 2H, CH_2_), 0.98 (d, *J* = 6.6 Hz, 6H, 2xCH_3_). ^13^C NMR (151 MHz, DMSO-*d_6_*) *δ* 161.6, 151.1, 150.4, 149.2, 147.7, 144.6, 141.2, 134.0, 125.5, 124.0, 120.6, 112.3, 108.4, 96.6, 89.2, 65.5, 53.6, 48.5, 48.4, 47.9, 47.0, 42.2, 18.0. HRMS (ESI/MS): *m*/*z* calculated for C_26_H_30_F_2_N_8_O_2_ [M + H]^+^ 525.2532 found 525.2544.

### 3.2. Docking Study

The docking procedure was performed using the PI3K*δ* protein from the Protein Data Bank (PDB: 2WXL) with the Auto-Dock Vina program [40]. All figures with examples of 3D modeling of a possible binding mode of selected compounds were prepared based on the calculated pK_a_ from the Instant JChem 21.13.0 program [39]. More specifically, all structures depicted in the respective figures have not had protons added, but the appropriate state of protonation has been maintained.

### 3.3. Biology

#### 3.3.1. In Vitro PI3K Inhibition Assays

The potency and selectivity of compounds were assessed by measuring the ability of PI3K kinases to convert ATP to ADP during an enzymatic reaction in the presence of decreasing doses of tested compounds. The experiments were carried out using the ADP-Glo kinase assay kit (Promega), according to the manufacturer’s protocol. PI3K*α*, PI3K*β*, PI3K*δ*, and PI3K*γ* have been purchased from Merck Millipore and phosphoinositol-4,5-bisphosphate (PIP2) lipid vesicles with phosphoserine from ThermoFisher Scientific were used as a substrate in the enzymatic reaction. The composition of the reaction mixture and reaction conditions for individual kinases are listed in the table below (Table 6).

After the reaction, the ADP-Glo reagent and the kinase detection reagent were added sequentially with 40 min of incubation (25 °C, 600 rpm) after adding each reagent. Finally, the luminescence intensity was measured and the IC_50_ was calculated using GraphPad Prism 7 software. The presented results are the mean value of IC_50_ from at least two independent experiments.

#### 3.3.2. Influence of Selected Compounds on B Cells Proliferation

CD19 cells were isolated from PBMCs using magnetic beads (Stem Cell (Vancouver, Canada)) and then labeled with 2 µM of CFSE (Invitrogen (Waltham, MA, USA)).

Then, 1 × 105 cells were seeded on 96-well plates, activated by 2 µg/mL of αIgM (Jackson ImmunoResearch (West Grove, PA, USA)) and 1 µg/mL of ODN2006 (InvivoGen (San Diego, CA, USA)), and incubated with increasing concentrations of drugs (0.1, 0.3, 1.0, 3.3, 10, 33, 100, 333, 1000, 3333, and 10000 nM). After 4 days, cells were stained with LIVE/DEAD™ kit (Invitrogen (Waltham, MA, USA)). Samples were acquired using Attune NxT flow cytometer (Invitrogen) and analyzed using FlowJo software. Each biological assay was performed with cells isolated from a different donor. The presented results constitute the average percentage values of proliferating cells from 3 independent experiments.

### 3.4. Metabolic Stability and Solubility

#### 3.4.1. Metabolic Stability Assay

The metabolic phase I stability in mouse (CD-1™) and human microsomes (Thermo-Fisher Scientific (Waltham, MA, USA)) was assessed on 96-well non-binding plates (Greiner (Kremsmuster, Austria)) at a 1 μM concentration for verapamil (positive control) and donepezil (negative control) and tested compounds. Unless otherwise stated, all chemicals and materials were ordered from Merck Life Science (Sheboygan, WI, USA). Each biological replicate was prepared in triplicates [40,41]. Briefly, compounds were incubated in 100 mM potassium phosphate buffer with microsomes (0.5 mg/mL) and NADPH (1–1.2 mM) on a plate shaker (500 rpm) in the dark at 37 °C. On a 4× solution of NADPH, the cofactor for metabolic enzymes was prepared directly prior to the experiment by reducing NADP with G6P dehydrogenase (13.2 mM MgCl_2_, 13.2 mM G6P, 5.2 mM NADP, 3.2 U/mL G6P dehydrogenase, 20 min at 30 °C, 500 rpm). The negative control contained buffer instead of NADPH solution. Samples were collected at 0, 10, 20, and 40 min or 0 and 40 min for the negative and double negative controls. The reaction was stopped by protein precipitation in 2 volumes of ice-cold MeOH with 200 nM of imipramine (as an internal standard for LC-MS analysis). Then, the extract was mixed (1 min, 1000 rpm), filtered through a 0.22 μm filter on a 96-well plate vacuum manifold, and subjected to LC-MS analysis.

#### 3.4.2. Lymphocyte B Proliferation Assessment

Human PBMCs were isolated from buffy coats of healthy donors obtained from the Regional Blood Donation and Blood Medicine Center in Warsaw. Lymphocytes B: CD19+ cells were isolated from PBMCs on magnetic beads (Stem Cell), labeled with 2 µM CFSE (Invitrogen), and seeded on a 96-well plate (1 × 10^5^ cells/well). B cells were activated by 2 µg/mL of *α*IgM (Jackson ImmunoResearch) and 1 µg/mL of ODN2006 (InvivoGen). Cells were incubated with increasing concentrations of compounds (range 0.03–10,000 nM). After 4 days, B cells were stained with LIVE/DEAD™ kit (Invitrogen), acquired using the Attune NxT flow cytometer (Invitrogen), and analyzed using FlowJo10 software. For data normalization, the percentage value of proliferating cells in each sample was divided by the average percentage value of positive control in a single experiment. IC_50_ values were calculated using a three-parameter dose–response inhibition function in GraphPad Prism.

#### 3.4.3. Kinetic Stability Assay

The kinetic solubility was determined using the shake-flask protocol [42,43]. The appropriate compounds (500 µM) were incubated in an aqueous buffer (0.1 M phosphate-buffered saline pH 7.4) at 25 °C with stirring at 500 rpm. The samples were taken at the start time and after 24 h of incubation, filtered through 0.22 µm filters, and diluted with 2 volumes of acetonitrile. Sample concentrations were determined by UHPLC-UV/Vis. A calibration curve was prepared in order to quantify the contents of the compound in the test solution.

## 4. Conclusions

A new family of substituted pyrazolo[1,5-*a*]pyrimidines was prepared in multi-step synthesis utilizing the Buchwald–Hartwig reaction or reductive amination as the crucial synthetic steps. The SAR studies were performed firstly at the C(5) position of the pyrazolo[1,5-*a*]pyrimidine and then the final optimization was turned up using a careful sterical amino group at the C(2) position adjustment. The biological activities were measured for each new compound against four PI3K isoforms: *α*, *β*, *γ*, and *δ*, providing comprehensive information on the selectivity of the obtained structures. Eleven compounds with an IC_50_ value below the 100 nM threshold within the new compounds’ library were synthesized in this work. Five of them, with an IC_50_ value below or equal to 52 nM, were assumed as hits. Molecular modeling studies provide a rational explanation for the interaction of active structures within the PI3K*δ* ATP binding site. CPL302415 (1-{2-[(4-*tert*-butylpiperazin-1-yl)methyl]-7-(morpholin-4-yl)pyrazolo[1,5-*a*]pyrimidin-5-yl}-2-(difluoromethyl)-1*H*-benzimidazole, compound **6**) proved to be the most potent structure with excellent activity (IC_50_ = 18 nM), good selectivity (PI3K*α*/PI3K*δ* = 79; PI3K*b*/*δ* = 1415; PI3K*γ*/PI3K*δ* = 939), and other promising parameters (Table 5). Therefore, CPL302415 was selected as a lead compound for toxicological studies and as a candidate for further development in phase I clinical trials in SLE treatment. More detailed biological and physicochemical studies and their outcomes are the subjects of a separate paper under preparation.

## Figures and Tables

**Table 1:** Activity and selectivity of benzimidazole derivatives (**5**–**12**).

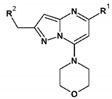									
Compound	R^1^	R^2^	IC_50_ PI3K*δ* [nM]	IC_50_ PI3K*α* [nM]	IC_50_ PI3K*β* [nM]	IC_50_ PI3K*γ* [nM]	Fold Selectivity		
							*α/* *δ*	*β/* *δ*	*γ* */* *δ*
**5**	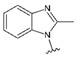	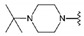	236	10,642	298,400	175,187	45	1264	742
**6**	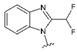	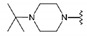	18	1428	25,475	16,904	79	1415	939
**7**	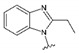	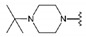	385	28,677	265,699	258,277	74	690	671
**8**	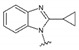	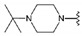	1072	82,502	259,143	271,097	77	242	253
**9**	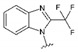	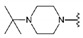	907			135,500			149
**11**	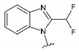	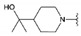	52	1729		6347	33		122
**12**	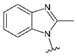	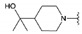	878	30,511	194,624	119,254	35	222	136

IC_50_ values were determined as the mean based on two independent experiments.

**Table 2:** PI3K*δ* and PI3K*g* activity of pyrazolo[1,5-*a*]pyrimidine derivatives with (-CH_2_) groups.

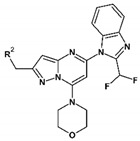				
Compound	R^2^	IC_50_ PI3K*δ* [nM]	IC_50_ PI3K*γ* [nM]	Fold Selectivity *γ/δ*
**16**	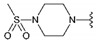	43	111	2.6
**17**	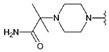	31	2197	71
**18**	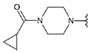	24	156	6.5
**19**	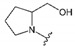	1070	4474	4.2
**20**	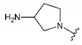	1892	22,134	12
**21**	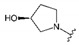	956	10,944	11
**22**	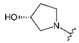	979	8420	8.6
**23**	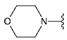	135	1380	10
**24**	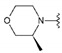	254	588	2.3
**25**	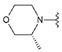	647	1972	3.0
**26**	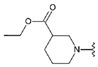	203	4283	21
**27**	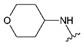	1805	20,526	11
**28**	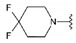	443	1709	3.9
**29**	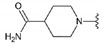	240	1855	7.7
**30**	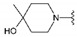	615	7553	12
**31**	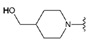	593	8318	14
**32**	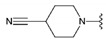	192	3155	16
**33**	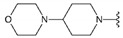	276	4800	17
**34**	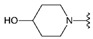	561	4317	7.7
**35**	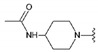	445	1503	3.4
**36**	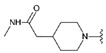	63	1247	20
**37**	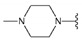	351	17,658	50
**38**	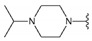	38	13,330	351

IC_50_ values were determined as the mean based on two independent experiments.

**Table 3:** PI3K*δ* and PI3K*g* activity of pyrazolo[1,5-*a*]pyrimidine derivatives with (-CO) groups.

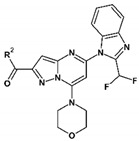				
Compound	R^2^	IC_50_ PI3K*δ* [nM]	IC_50_ PI3K*γ* [nM]	Fold Selectivity *γ/δ*
**40**	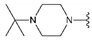	84	48,777	581
**41**	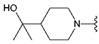	101	2483	25
**42**	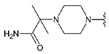	74	3593	48
**43**	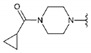	63	3831	61
**44**	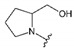	459	2024	4.4
**45**	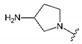	1704	23,290	14
**46**	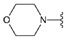	226	8536	38
**47**	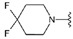	801	35,750	45
**48**	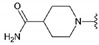	314	2793	9
**49**	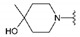	213	1818	8.5
**50**	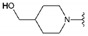	90	1782	20
**51**	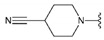	344	1629	4.7
**52**	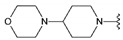	92	5946	65
**53**	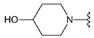	195	2081	11
**54**	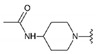	141	1689	12
**55**	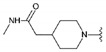	82	2170	26
**56**	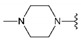	768	11,397	94
**57**	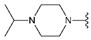	271	25,527	15

IC_50_ values were determined as the mean based on two independent experiments.

**Table 4:** Activity and selectivity of the most promising compounds.

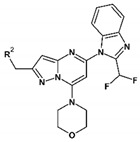									
Compound	R^2^	IC_50_ PI3K*δ* [nM]	IC_50_ PI3K*α* [nM]	IC_50_ PI3K*β* [nM]	IC_50_ PI3K*γ* [nM]	*α/* *δ*	*β/* *δ*	*γ* */* *δ*	IC_50_ CD19 [nM]
**6**	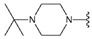	18	1428	25,475	16,904	79	1415	939	41
**11**	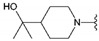	52	1729		6347	33		122	
**16**	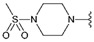	43	44	13,577	111	1.0	316	2.6	114
**17**	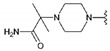	31	624	44,753	2197	20	1444	71	52
**18**	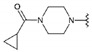	24	73	47,360	156	3.0	1973	6.5	58

IC_50_ values were determined as the mean based on two independent experiments.

**Table 5:** Selected parameters measured for CPL302415 (**6**).

	Kinetic Solubility pH 7.4 [mM]	Metabolic Stability				PAMPA $\left[10^{-6}\;\mathrm{cm}\times s^{-1}\right]$	Plasma Protein Binding [%]			
		MLM t_½_ [min]	MLM CL $\left[\mathrm{mL}\times {\mathrm{mn}}^{-1}\times {\mathrm{mg}}^{-1}\right]$	HLM t_½_ [min]	HLM CL $\left[\mathrm{mL}\times \min^{-1}\times {\mathrm{mg}}^{-1}\right]$		Human	Monkey	Mice	Rat
**6**	>500	378	3.7	145	9.6	13.3	79	81	83	82

**Table 6:** Reaction conditions and compositions of mixtures for individual kinases.

KINASE	Kinase Concentration [ng per Reaction]	Reaction Temperature and Time	Substrate PIP2 [Final Concentration μM]	Reaction Buffer
PI3K*α* (Carna Biosciences)	7.5 ng	25 °C, 1 h	30 μM	50 mM of HEPES pH 7.5 50 mM of NaCl 3 mM of MgCl_2_ 0.025 mg/mL of BSA
PI3K*δ* (Merck Millipore)	10 ng	25 °C, 1 h	30 μM	50 mM of HEPES pH 7.5 50 mM of NaCl 3 mM of MgCl_2_ 0.025 mg/mL of BSA
PI3K*β* (Merck Millipore)	15 ng	30 °C, 1 h	50 μM	50 mM of HEPES pH 7.5 50 mM of NaCl 3 mM of MgCl_2_ 0.025 mg/mL of BSA
PI3K*α* (Merck Millipore)	30 ng	30 °C, 1 h	50 μM	40 nM of Tris pH 7.5 20 mM of MgCl_2_ 0.1 mg/mL of BSA 1 mM of DTT

## Data Availability

Data is contained within the article and supplementary material.

## Supplementary Materials

The following supporting information can be downloaded at: https://www.mdpi.com/article/10.3390/ph15080927/s1, 1H NMR, 13C NMR and HRMS supplementary figures of the final compounds **5**–**9**, **11**–**12**, **16**–**38**, and **40**–**54**.
