# Supplementary material for: Design, Synthesis, and Development of Pyrazolo[1,5-a]pyrimidine Derivatives as a Novel Series of Selective PI3Kδ Inhibitors: Part II—Benzimidazole Derivatives
Source: Pharmaceuticals (Basel). 2022 Jul 27;15(8):927. doi: 10.3390/ph15080927 (PMC9415947; doi:10.3390/ph15080927)
Supplement: Supplementary file 1 [file pharmaceuticals-15-00927-s001.zip › pharmaceuticals-1805734-supplementary.pdf]

Supplementary data

for

Design, synthesis, and development of pyrazolo[1,5-*a*]pyrimidine derivatives as a novel series of selective PI3K*δ* inhibitors. Part II – Benzimidazole derivatives.

Mariola Stypik[1,2]*, Stanisław Michałek[1,2], Nina Orłowska[1,2], Marcin Zagozda[1], Maciej Dziachan[1], Martyna Banach[1], Paweł Turowski[1], Paweł Gunerka[1], Daria Zdżalik-Bielecka[1], Aleksandra Stańczak[1], Urszula Kędzierska[1], Krzysztof Mulewski[1], Damian Smuga[1], Wioleta Maruszak[1], Lidia Gurba-Bryśkiewicz[1], Zbigniew Ochal[2], Mateusz Mach[1], Beata Zygmunt[1], Jerzy Pieczykolan[1], Krzysztof Dubiel[1], Maciej Wieczorek[1]

[1] Celon Pharma S.A., R&D Centre, Marymoncka 15, 05-152 Kazuń Nowy, Poland
[2] Warsaw University of Technology, Faculty of Chemistry, Noakowskiego 3, 00-664 Warsaw, Poland
*corresponding author: mariola.stypik@celonpharma.com

^{1}H NMR, ^{13}C NMR and HRMS supplementary figures of the final compounds **5-9**, **11-12**, **16-38** and **40-54**

INDEX

Compound 5 ^{1}H NMR and ^{13}C NMR 5

Compound 5 HRMS 6

Compound 6 ^{1}H NMR and ^{13}C NMR 7

Compound 6 HRMS 8

Compound 7 ^{1}H NMR and ^{13}C NMR 9

Compound 7 HRMS 10

Compound 8 ^{1}H NMR and ^{13}C NMR 11

Compound 8 HRMS 12

Compound 9 ^{1}H NMR and ^{13}C NMR 13

Compound 9 HRMS 14

Compound 11 ^{1}H NMR and ^{13}C NMR 15

Compound 11 HRMS 16

Compound 12 ^{1}H NMR and ^{13}C NMR 17

Compound 12 HRMS 18

Compound 16 ^{1}H NMR and ^{13}C NMR 19

Compound 16 HRMS 20

Compound 17 ^{1}H NMR and ^{13}C NMR 21

Compound 17 HRMS 22

Compound 18 ^{1}H NMR and ^{13}C NMR 23

Compound 18 HRMS 24

Compound 19 ^{1}H NMR and ^{13}C NMR 25

Compound 19 HRMS 26

Compound 20 ^{1}H NMR and ^{13}C NMR 27

Compound 20 HRMS 28

Compound 21 ^{1}H NMR and ^{13}C NMR 29

Compound 21 HRMS 30

Compound 22 ^{1}H NMR and ^{13}C NMR 31

Compound 22 HRMS 32

Compound 23 ^{1}H NMR and ^{13}C NMR 33

Compound 23 HRMS 34

Compound 24 ^{1}H NMR and ^{13}C NMR 35

Compound 24 HRMS 36

Compound 25 ^{1}H NMR and ^{13}C NMR 37

Compound 25 HRMS 38

Compound 26 ^{1}H NMR and ^{13}C NMR 39

Compound 26 HRMS 40

Compound 27 ^{1}H NMR and ^{13}C NMR 41
Compound 27 HRMS 42
Compound 28 ^{1}H NMR and ^{13}C NMR 43
Compound 28 HRMS 44
Compound 29 ^{1}H NMR and ^{13}C NMR 45
Compound 29 HRMS 46
Compound 30 ^{1}H NMR and ^{13}C NMR 47
Compound 30 HRMS 48
Compound 31 ^{1}H NMR and ^{13}C NMR 49
Compound 31 HRMS 50
Compound 32 ^{1}H NMR and ^{13}C NMR 51
Compound 32 HRMS 52
Compound 33 ^{1}H NMR and ^{13}C NMR 53
Compound 33 HRMS 54
Compound 34 ^{1}H NMR and ^{13}C NMR 55
Compound 34 HRMS 56
Compound 35 ^{1}H NMR and ^{13}C NMR 57
Compound 35 HRMS 58
Compound 36 ^{1}H NMR and ^{13}C NMR 59
Compound 36 HRMS 60
Compound 37 ^{1}H NMR and ^{13}C NMR 61
Compound 37 HRMS 62
Compound 38 ^{1}H NMR and ^{13}C NMR 63
Compound 38 HRMS 64
Compound 40 ^{1}H NMR and ^{13}C NMR 65
Compound 40 HRMS 66
Compound 41 ^{1}H NMR and ^{13}C NMR 67
Compound 41 HRMS 68
Compound 42 ^{1}H NMR and ^{13}C NMR 69
Compound 42 HRMS 70
Compound 43 ^{1}H NMR and ^{13}C NMR 71
Compound 43 HRMS 72
Compound 44 ^{1}H NMR and ^{13}C NMR 73
Compound 44 HRMS 74
Compound 45 ^{1}H NMR and ^{13}C NMR 75
Compound 45 HRMS 76
Compound 46 ^{1}H NMR and ^{13}C NMR 77

Compound 46 HRMS 78
Compound 47 ^{1}H NMR and ^{13}C NMR 79
Compound 47 HRMS 80
Compound 48 ^{1}H NMR and ^{13}C NMR 81
Compound 48 HRMS 82
Compound 49 ^{1}H NMR and ^{13}C NMR 83
Compound 49 HRMS 84
Compound 50 ^{1}H NMR and ^{13}C NMR 85
Compound 50 HRMS 86
Compound 51 ^{1}H NMR and ^{13}C NMR 87
Compound 51 HRMS 88
Compound 52 ^{1}H NMR and ^{13}C NMR 89
Compound 52 HRMS 90
Compound 53 ^{1}H NMR and ^{13}C NMR 91
Compound 53 HRMS 92
Compound 54 ^{1}H NMR and ^{13}C NMR 93
Compound 54 HRMS 94
Compound 55 ^{1}H NMR and ^{13}C NMR 95
Compound 55 HRMS 96
Compound 56 ^{1}H NMR and ^{13}C NMR 97
Compound 56 HRMS 98
Compound 57 ^{1}H NMR and ^{13}C NMR 99
Compound 57 HRMS 100

Compound 5

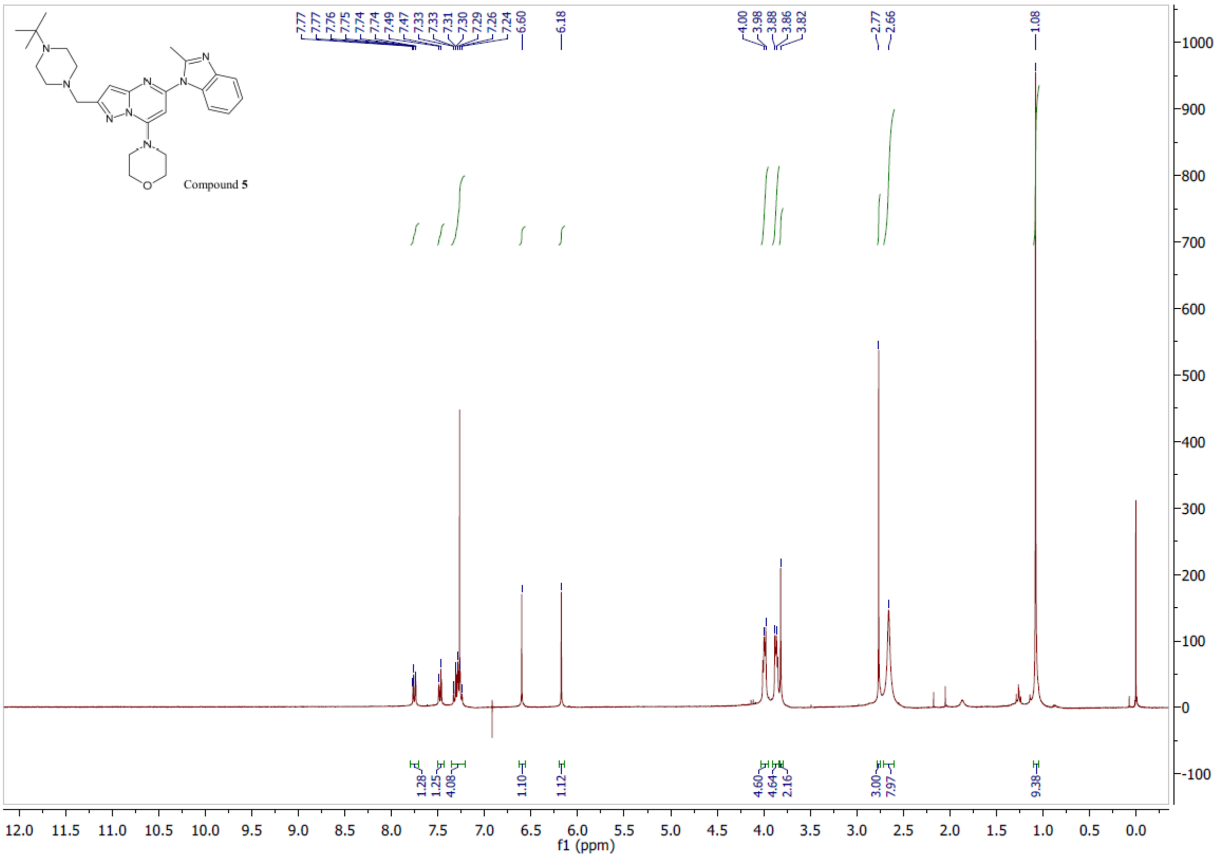

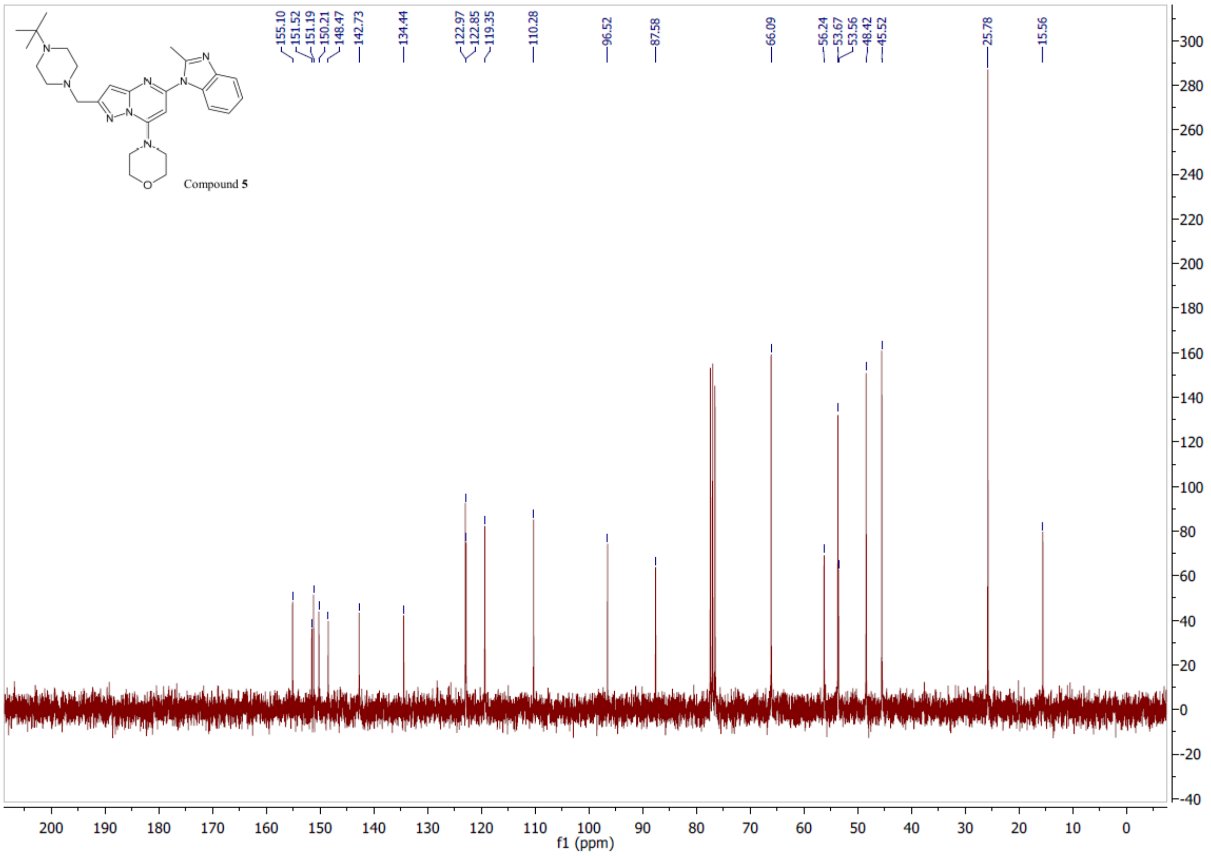

Compound 5

Compound Label	Name	*m/z*	RT	Algorithm	Mass
Compound **5**	Compound **5**	489.30884	0.24	Find by Formula	488.30154

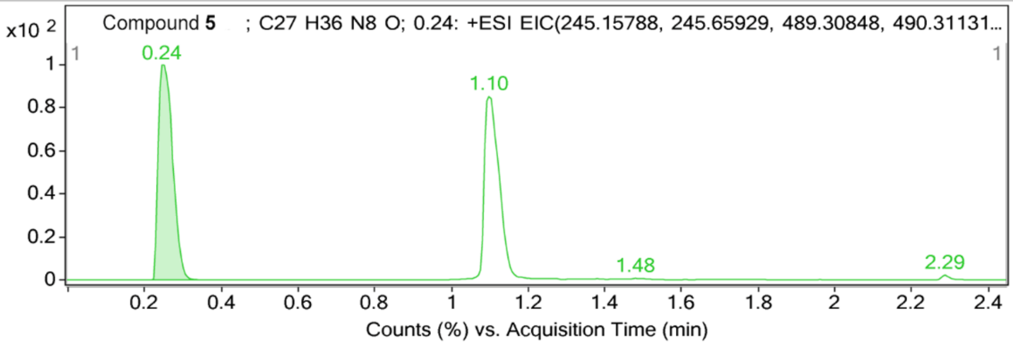

MS Zoomed Spectrum

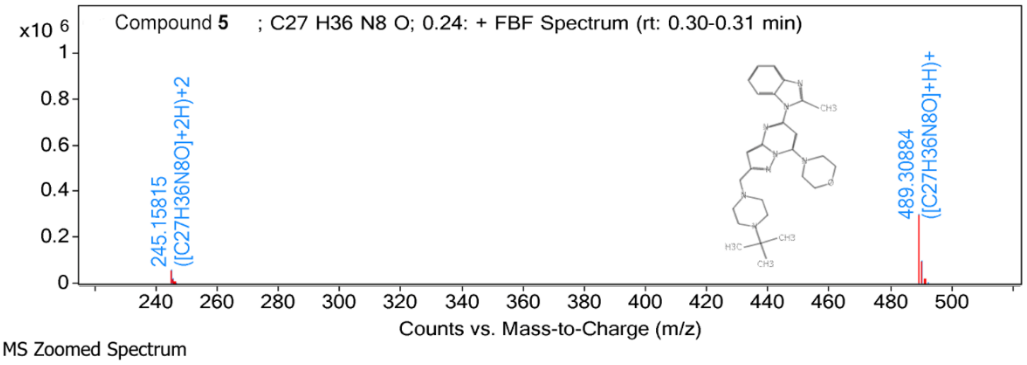

MS Zoomed Spectrum

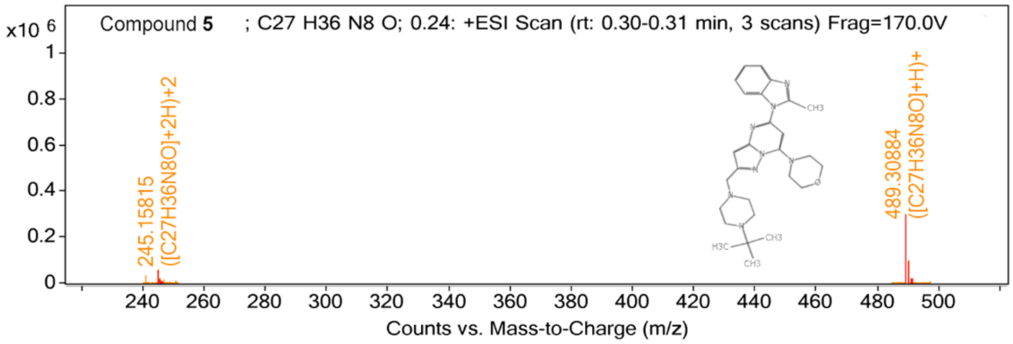

MS Spectrum Peak List

m/z	*Calc m/z*	Diff(ppm)	z	Abund	Formula	Ion
245.15815	245.15788	1.12	2	56190	C27H36N8O	(M+2H)+2
245.65963	245.65929	1.38	2	18164.55	C27H36N8O	(M+2H)+2
246.16121	246.16067	2.2	2	2663.72	C27H36N8O	(M+2H)+2
246.66092	246.66202	-4.46	2	280.06	C27H36N8O	(M+2H)+2
489.30884	489.30848	0.72	1	298324.84	C27H36N8O	(M+H)+
490.31149	490.31131	0.37	1	97178.29	C27H36N8O	(M+H)+
491.31367	491.31407	-0.81	1	14949.13	C27H36N8O	(M+H)+
492.31654	492.31675	-0.42	1	1609.93	C27H36N8O	(M+H)+

--- End Of Report ---

Compound 6

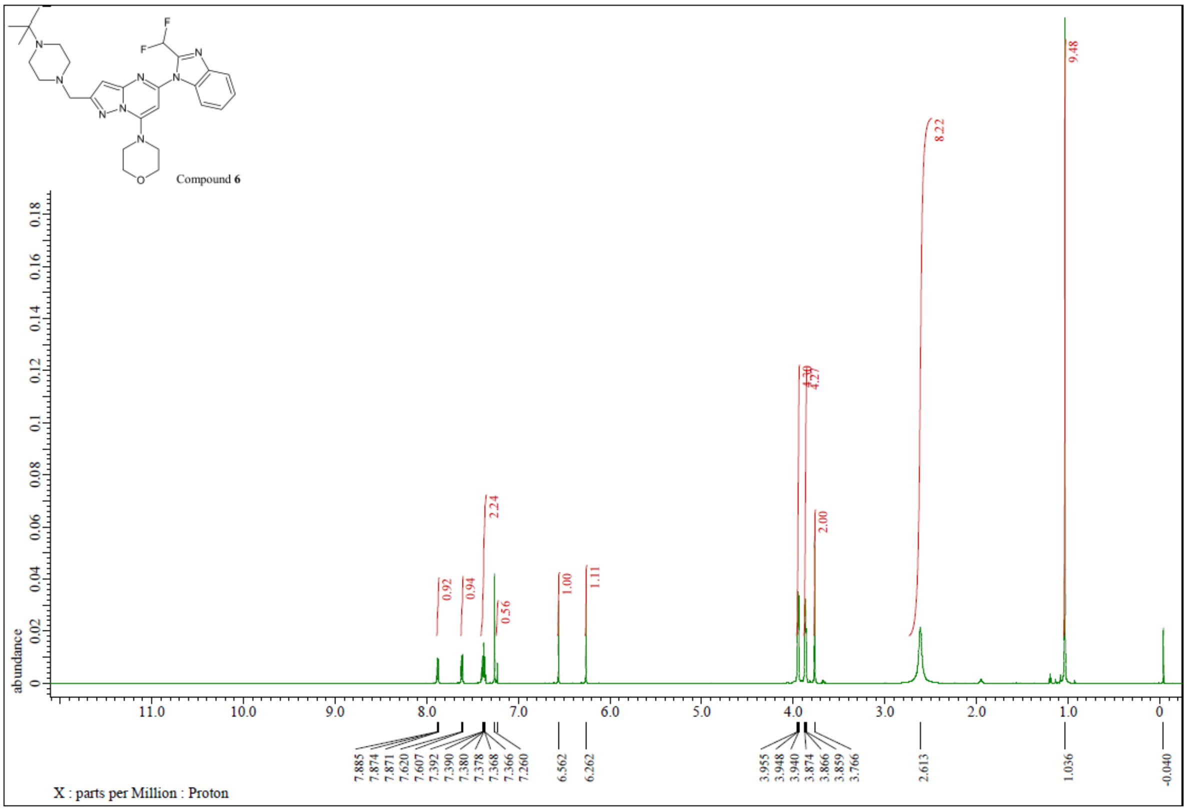
Compound 6
abundance
X : parts per Million : Proton

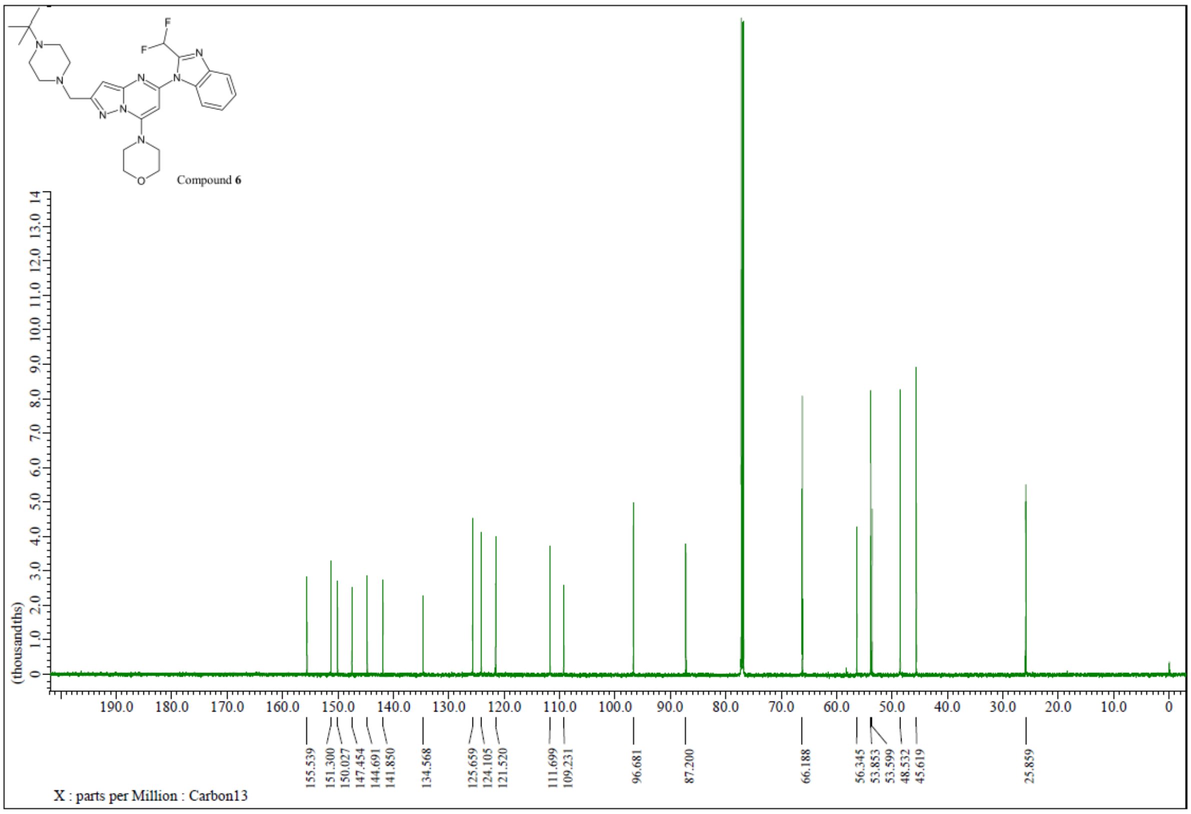
Compound 6
(thousandths)
X : parts per Million : Carbon13

Compound 6

Compound Label	Name	*m/z*	RT	Algorithm	Mass
Compound **6**	Compound **6**	525.29001	2.414	Find by Formula	524.28269

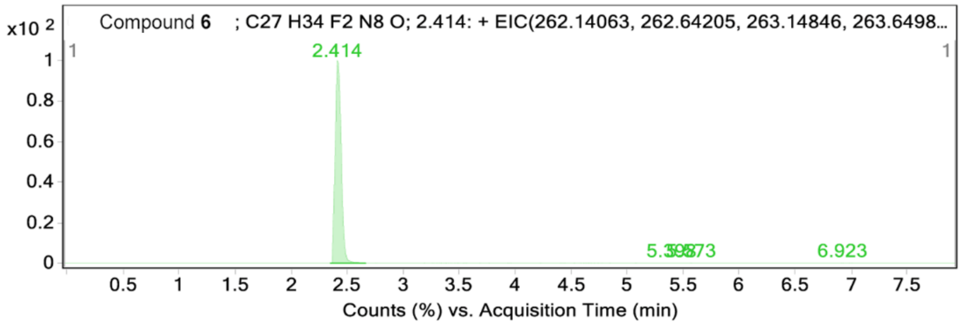

MS Zoomed Spectrum

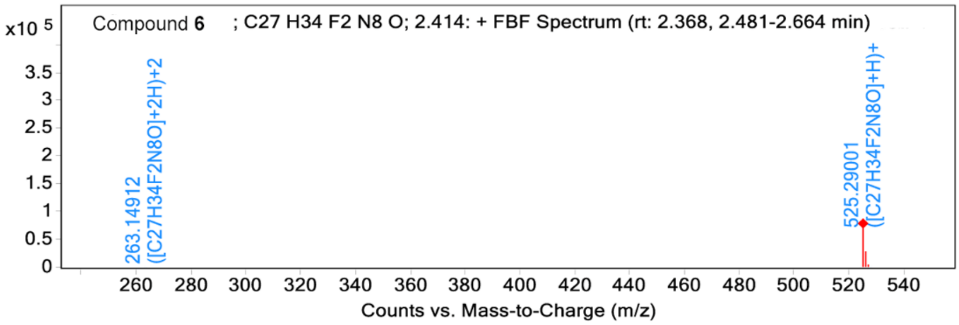

MS Zoomed Spectrum

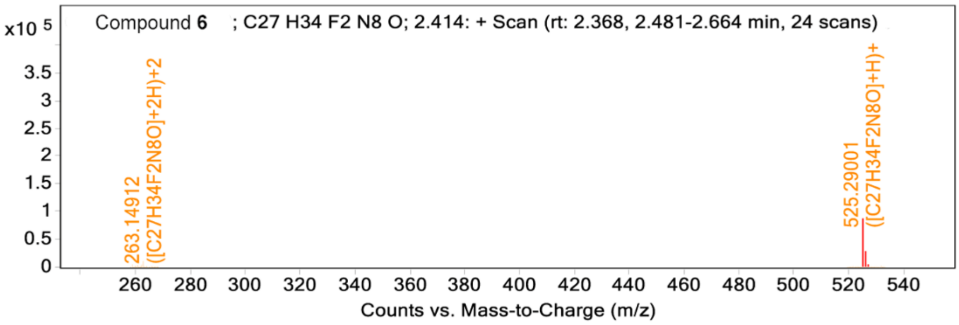

MS Spectrum Peak List

m/z	*Calc m/z*	Diff(ppm)	z	Abund	Formula	Ion
263.14912	263.14846	2.5	2	359.79	C27H34F2N8O	(M+2H)+2
263.64952	263.64987	-1.32	2	96.69	C27H34F2N8O	(M+2H)+2
524.27521	524.28182	-12.6	1	183.57	C27H34F2N8O	M+
525.29001	525.28964	0.7	1	86308.49	C27H34F2N8O	(M+H)+
526.29271	526.29247	0.47	1	27103.87	C27H34F2N8O	(M+H)+
527.29545	527.29522	0.45	1	3988.21	C27H34F2N8O	(M+H)+
528.29851	528.2979	1.16	1	428.02	C27H34F2N8O	(M+H)+

Compound 7

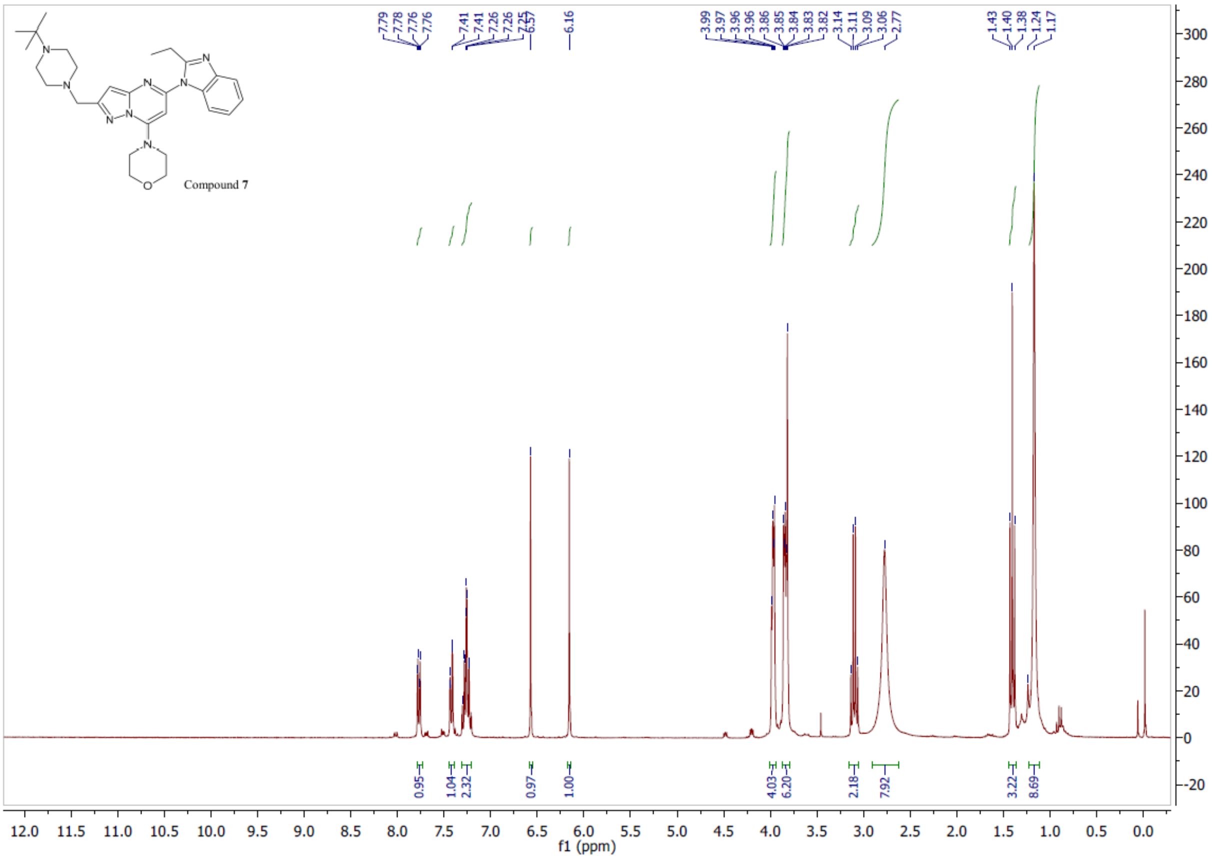
Compound 7
7.79
7.78
7.76
7.76
7.41
7.41
7.26
7.26
7.25
6.57
6.16
3.99
3.97
3.96
3.96
3.86
3.85
3.84
3.83
3.82
3.14
3.11
3.09
3.06
2.77
1.43
1.40
1.38
1.24
1.17
0.95
1.04
2.32
0.97
1.00
4.03
6.20
2.18
7.92
3.22
8.69
f1 (ppm)

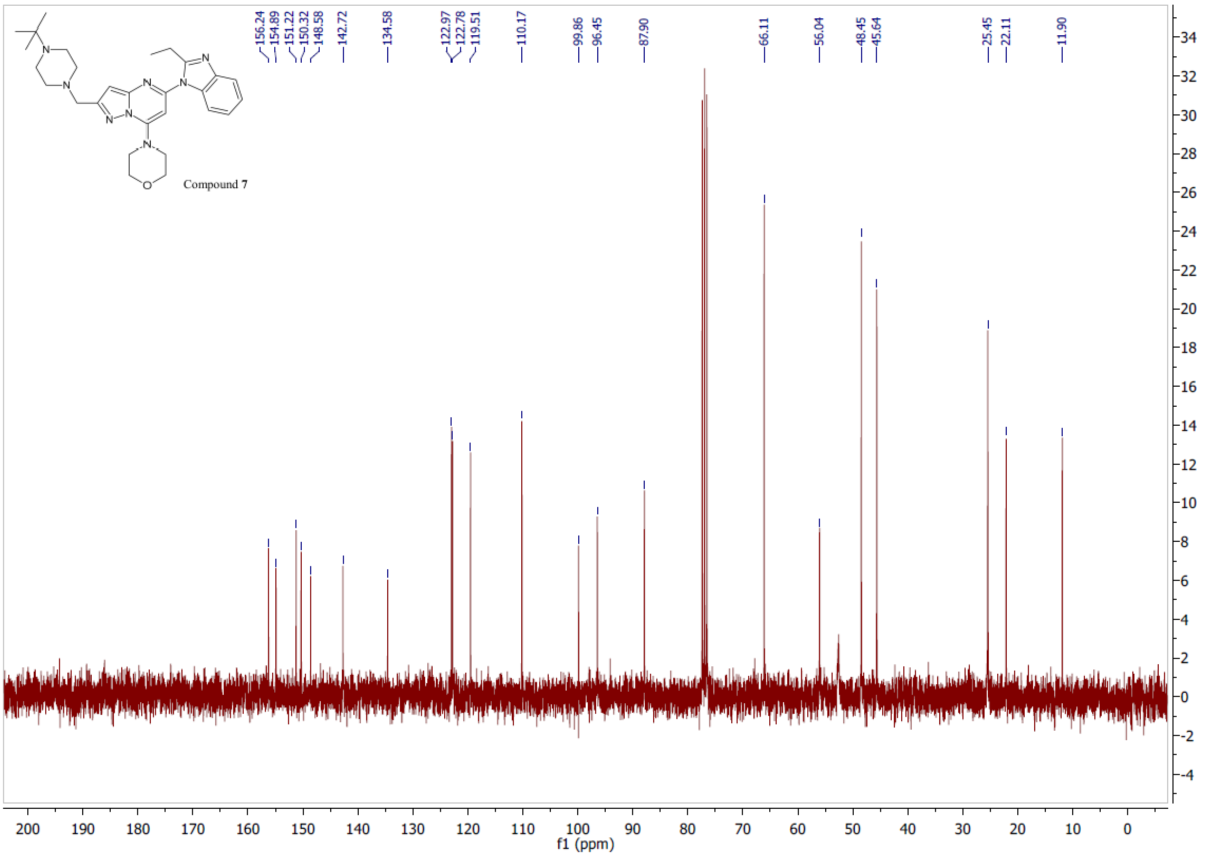
Compound 7
156.24
154.89
151.22
150.32
148.58
142.72
134.58
122.97
122.78
119.51
110.17
99.86
96.45
87.90
66.11
56.04
48.45
45.64
25.45
22.11
11.90
f1 (ppm)

Compound 7

Compound Details

Cpd. 12: Compound 7

Name	Formula	RT	RI	Mass	Diff (Tgt, ppm)	CAS	ID Source	Score	Algorithm
Compound 7	C28 H38 N8 O	1.100		502.3171	0.41		FBF	99.96	FBF

Species	m/z	Score (Tgt)	Score (Lib)	Score (DB)	Score (MFG)	Score (RT)
(M+2H)+2 (M+2K)+2 (M+H)+	252.1661 290.1263 503.3243	99.96				

Compound Chromatograms (overlaid)

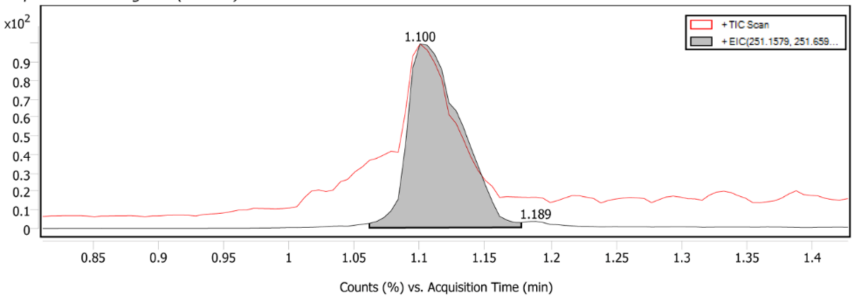

Structure

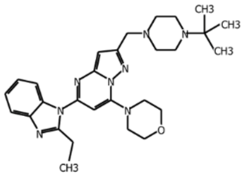

Compound Spectra (overlaid)

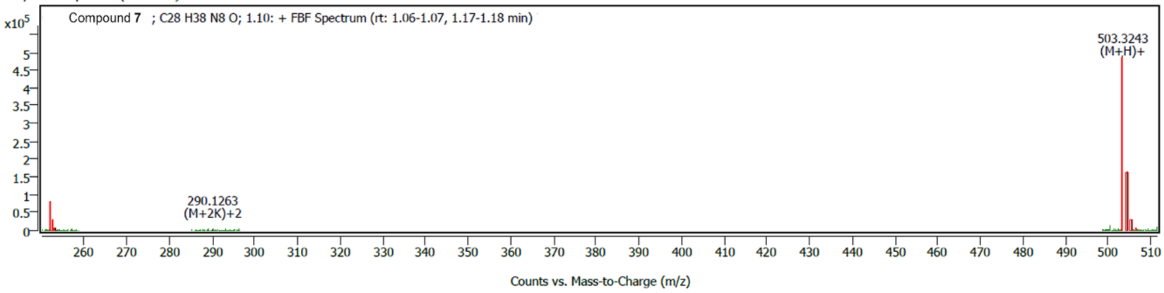

Compound ID Table

Name	Formula	Species	RT	RT Diff	Mass	CAS	ID Source	Score	Score (Lib)	Score (Tgt)
Compound 7	C28 H38 N8 O	(M+2H)+2 (M+2K)+2 (M+H)+	1.100		502.3171		FBF	99.96		99.96

Compound 8

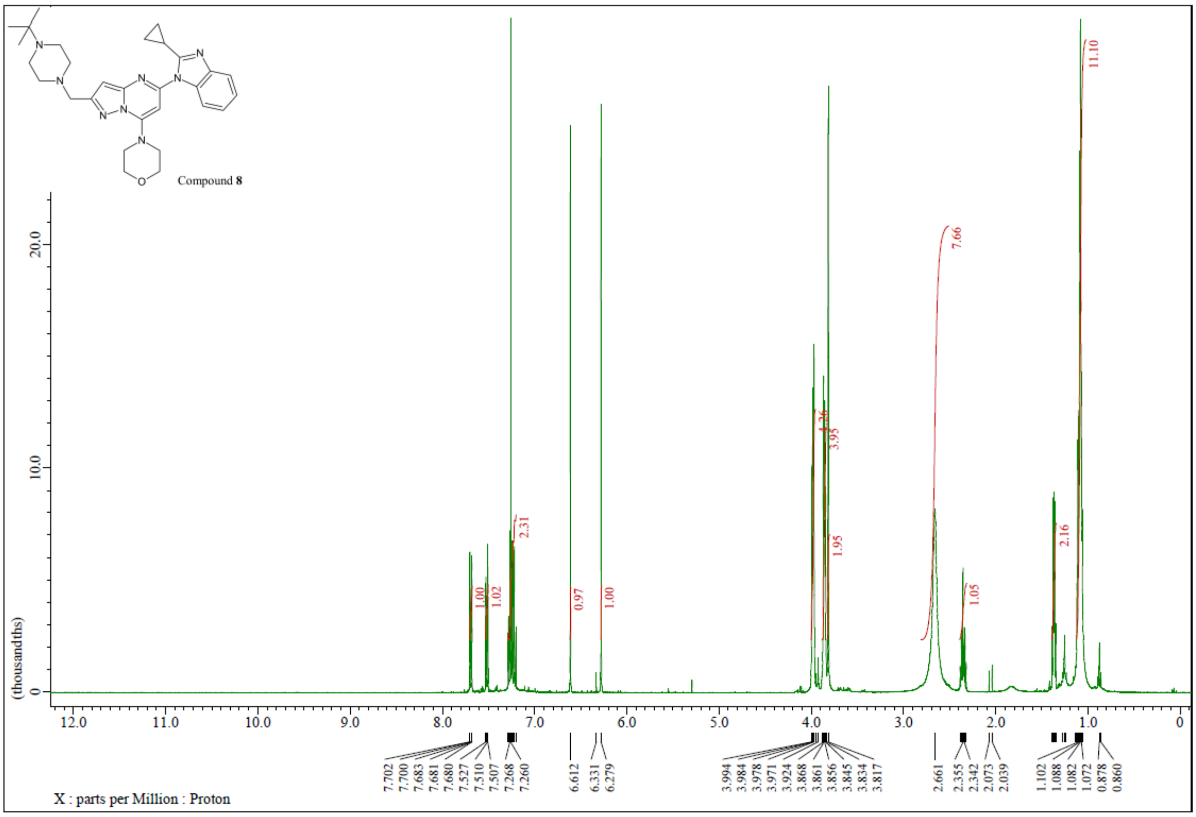

Compound 8
X : parts per Million : Proton
(thousandths)

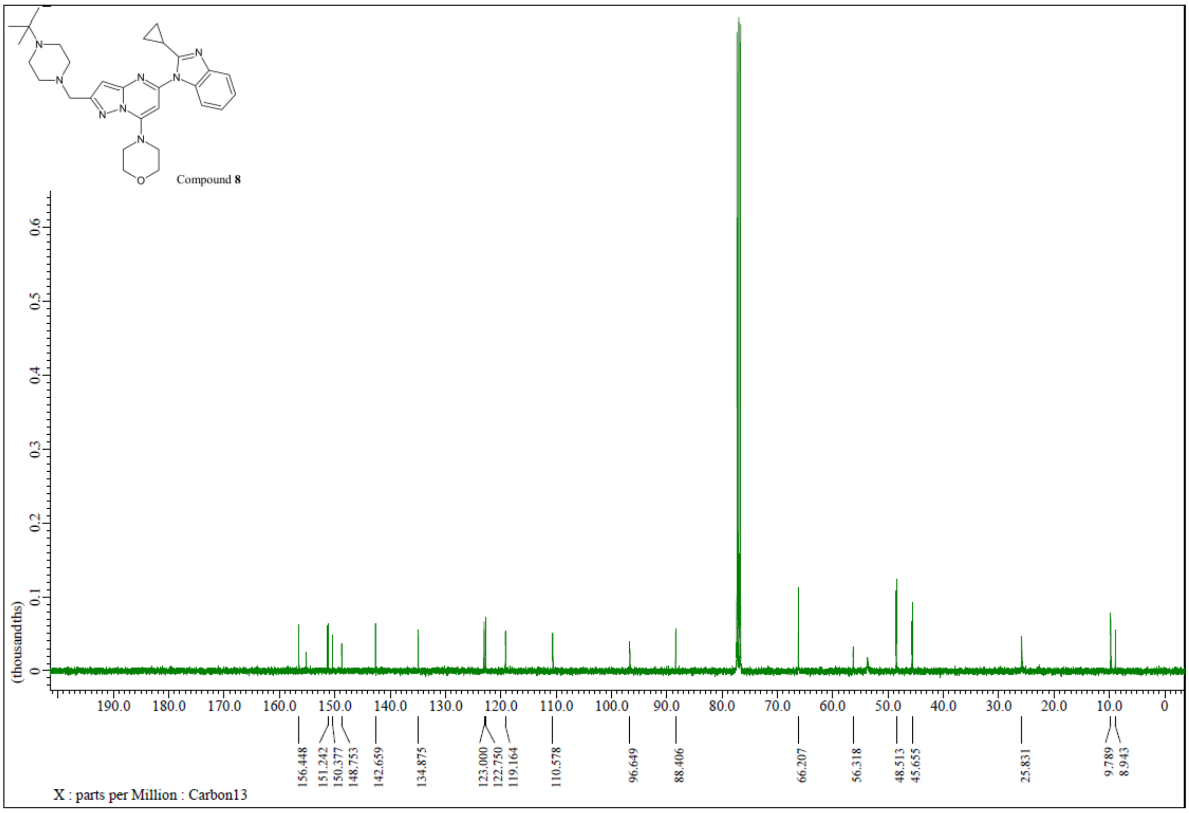

Compound 8
X : parts per Million : Carbon13
(thousandths)

Compound 8

Compound Table

Compound Label

Compound **8**

Compound Label	Algorithm
Compound 8	Spectrum Extraction

MS Spectrum

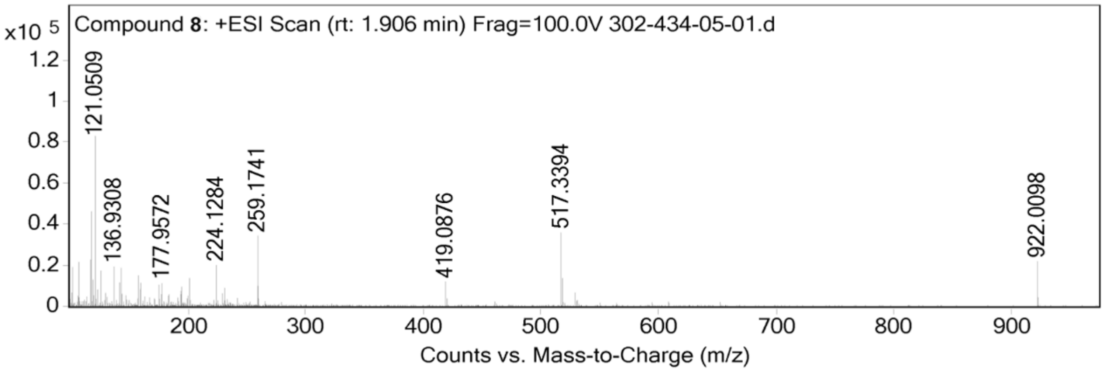

MS Zoomed Spectrum

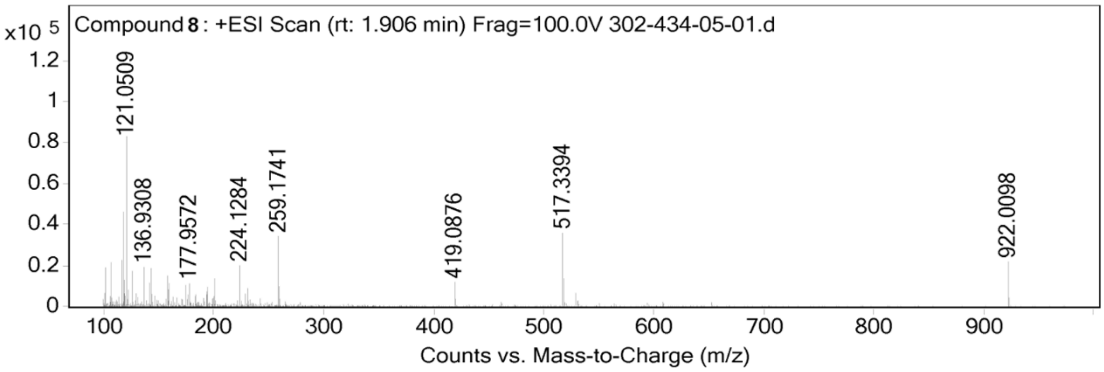

MS Spectrum Peak List

m/z	z	Abund
102.1278		18882.98
107.0437		21485.96
117.0909		22488.31
118.0865		46076.47
121.0509		82922.48
136.9308		19114.65
224.1284		19938.67
259.1741	2	34202.22
517.3394	1	35841.52
922.0098		21757.48

--- End Of Report ---

Compound 9

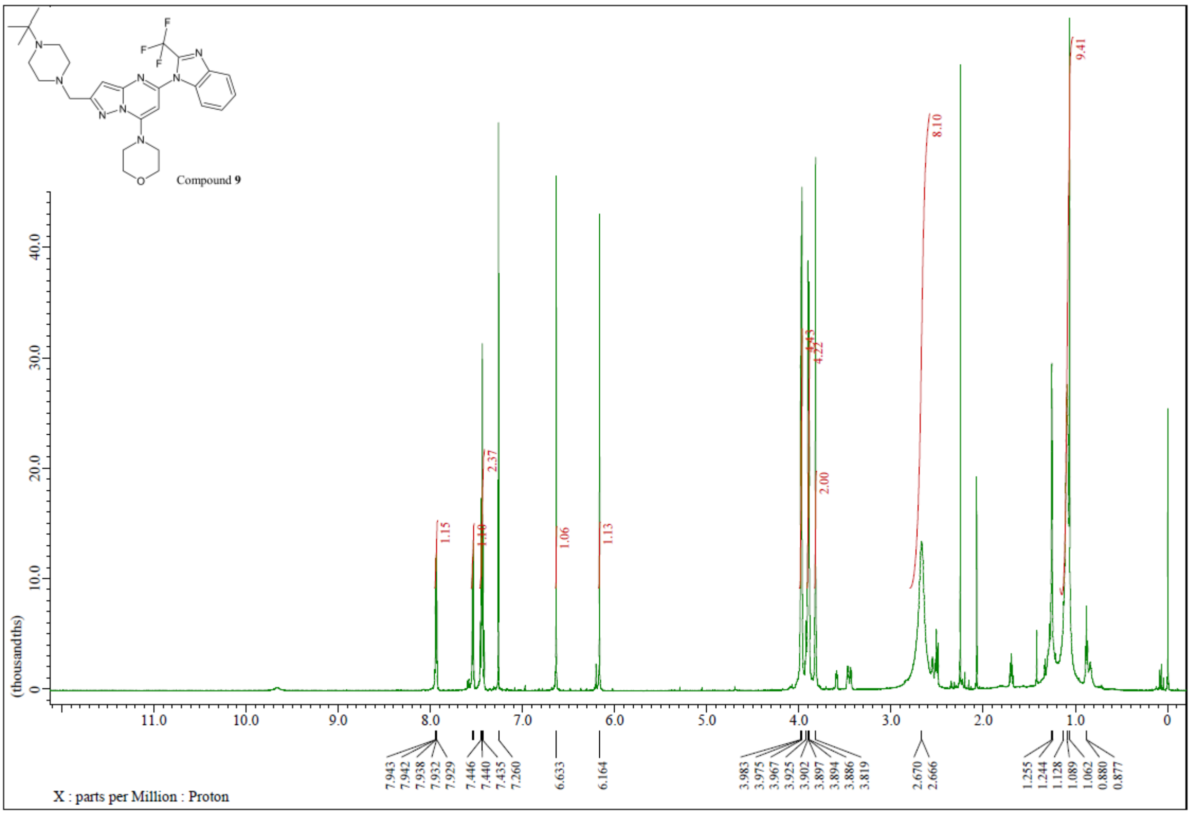

Compound 9
X : parts per Million : Proton
(thousandths)
7.943
7.942
7.938
7.932
7.929
7.446
7.440
7.435
7.260
6.633
6.164
3.983
3.975
3.967
3.925
3.902
3.897
3.894
3.886
3.819
2.670
2.666
1.255
1.244
1.128
1.089
1.062
0.880
0.877
1.15
1.18
2.37
1.06
1.13
4.22
2.00
8.10
9.41

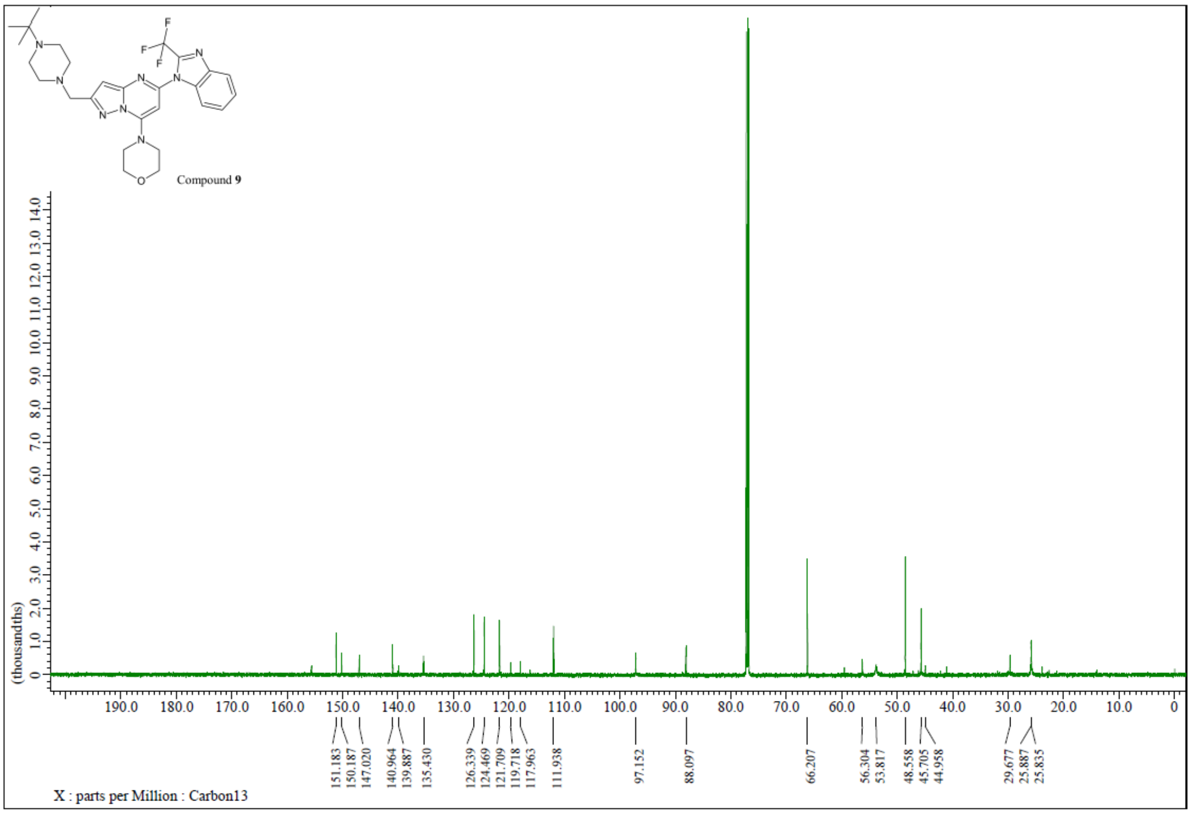

Compound 9
X : parts per Million : Carbon13
(thousandths)
151.183
150.187
147.020
140.964
139.887
135.430
126.339
124.469
121.709
119.718
117.963
111.938
97.152
88.097
66.207
56.304
53.817
48.558
45.705
44.958
29.677
25.887
25.835

Compound 9

Name	*Obs. m/z*	Obs. RT	Obs. Mass	Tgt Formula	Tgt Mass	Tgt Mass Error	Find Cpds Algorit
Compound **9**	272.14376	1.41	542.27339	C27 H33 F3 N8 O	542.27294	0.83	Find by Formula

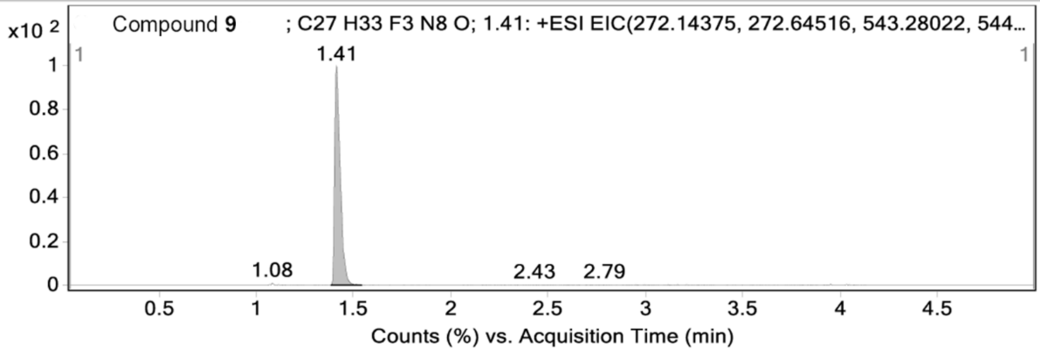

MS Zoomed Spectrum

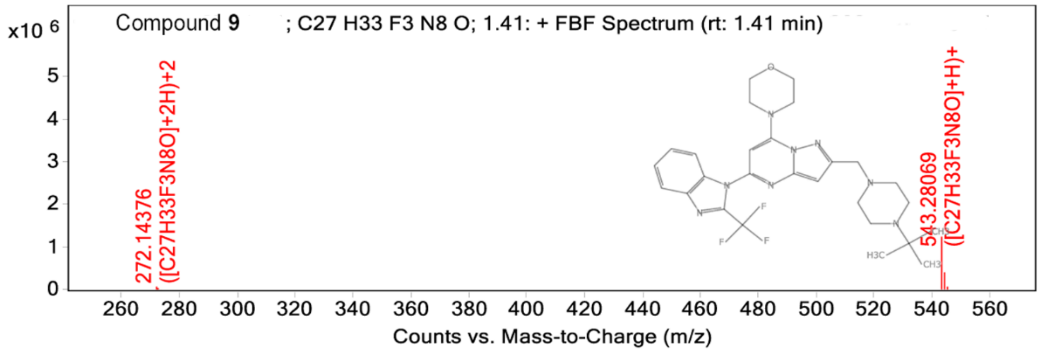

MS Zoomed Spectrum

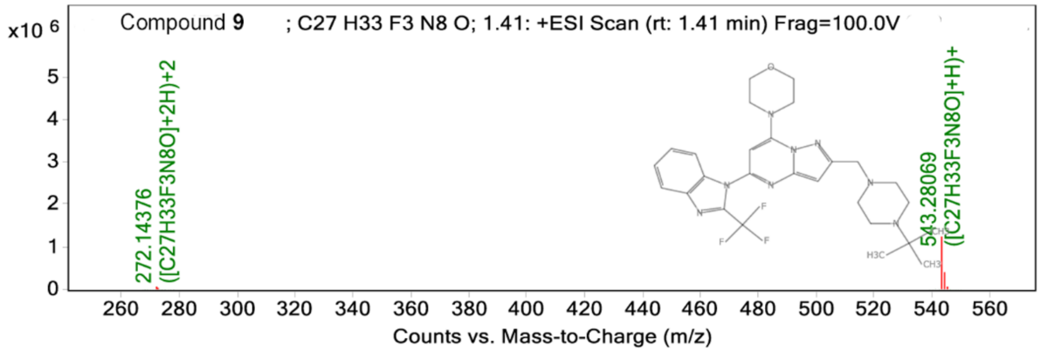

MS Spectrum Peak List

Obs. m/z	Charge	Abund	Formula	Ion/Isotope	Tgt Mass Error (ppm)
272.14376	2	65592.5	C27H33F3N8O	(M+2H)+2	
272.6453	2	23443.74	C27H33F3N8O	(M+2H)+2	
273.14618	2	4575.98	C27H33F3N8O	(M+2H)+2	
273.64635	2	210.29	C27H33F3N8O	(M+2H)+2	
543.28069	1	1223762.88	C27H33F3N8O	(M+H)+	
544.28358	1	331950.72	C27H33F3N8O	(M+H)+	
545.28592	1	53670.24	C27H33F3N8O	(M+H)+	
272.14376	2	65592.5	C27H33F3N8O	(M+2H)+2	0.03
272.6453	2	23443.74	C27H33F3N8O	(M+2H)+2	0.53
273.14618	2	4575.98	C27H33F3N8O	(M+2H)+2	-1.32
273.64635	2	210.29	C27H33F3N8O	(M+2H)+2	-5.59
543.28069	1	1223762.88	C27H33F3N8O	(M+H)+	0.87
544.28358	1	331950.72	C27H33F3N8O	(M+H)+	0.99
545.28592	1	53670.24	C27H33F3N8O	(M+H)+	0.23

--- End Of Report ---

Compound 11

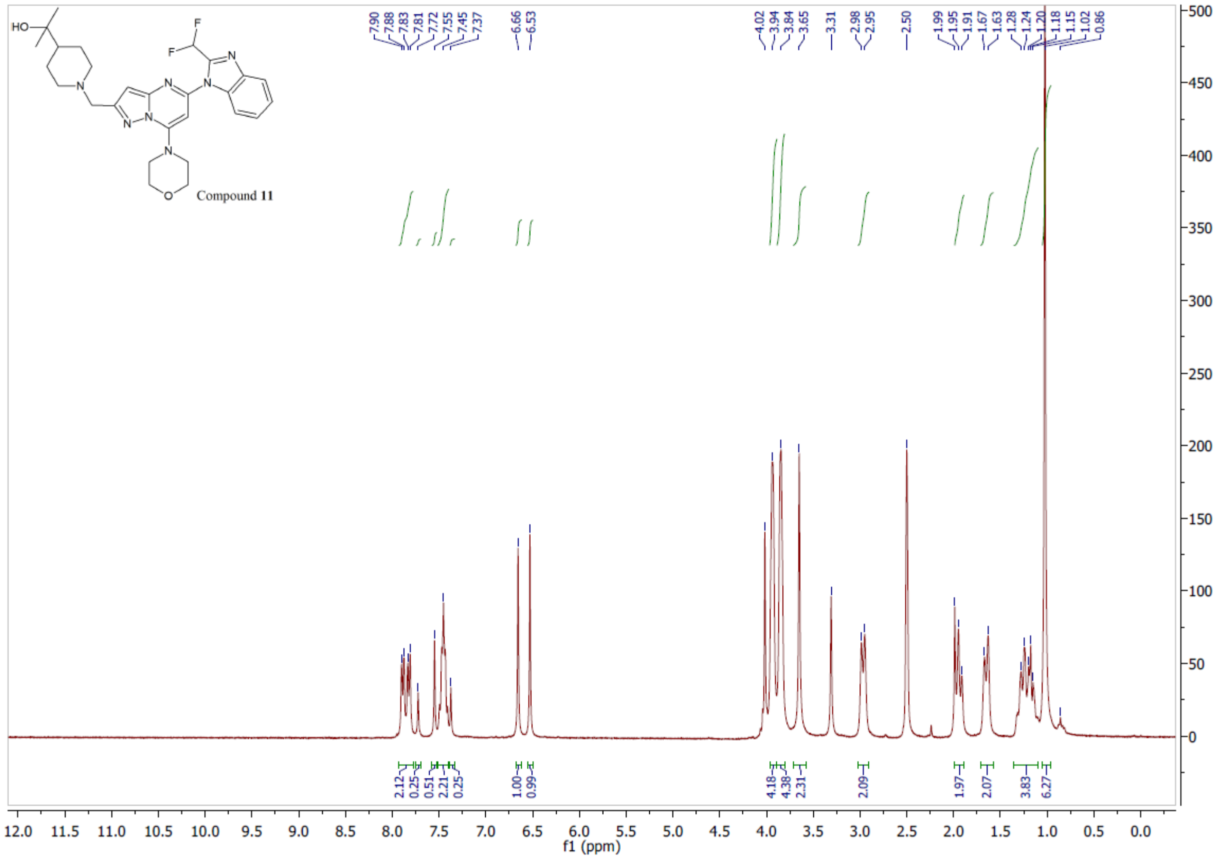

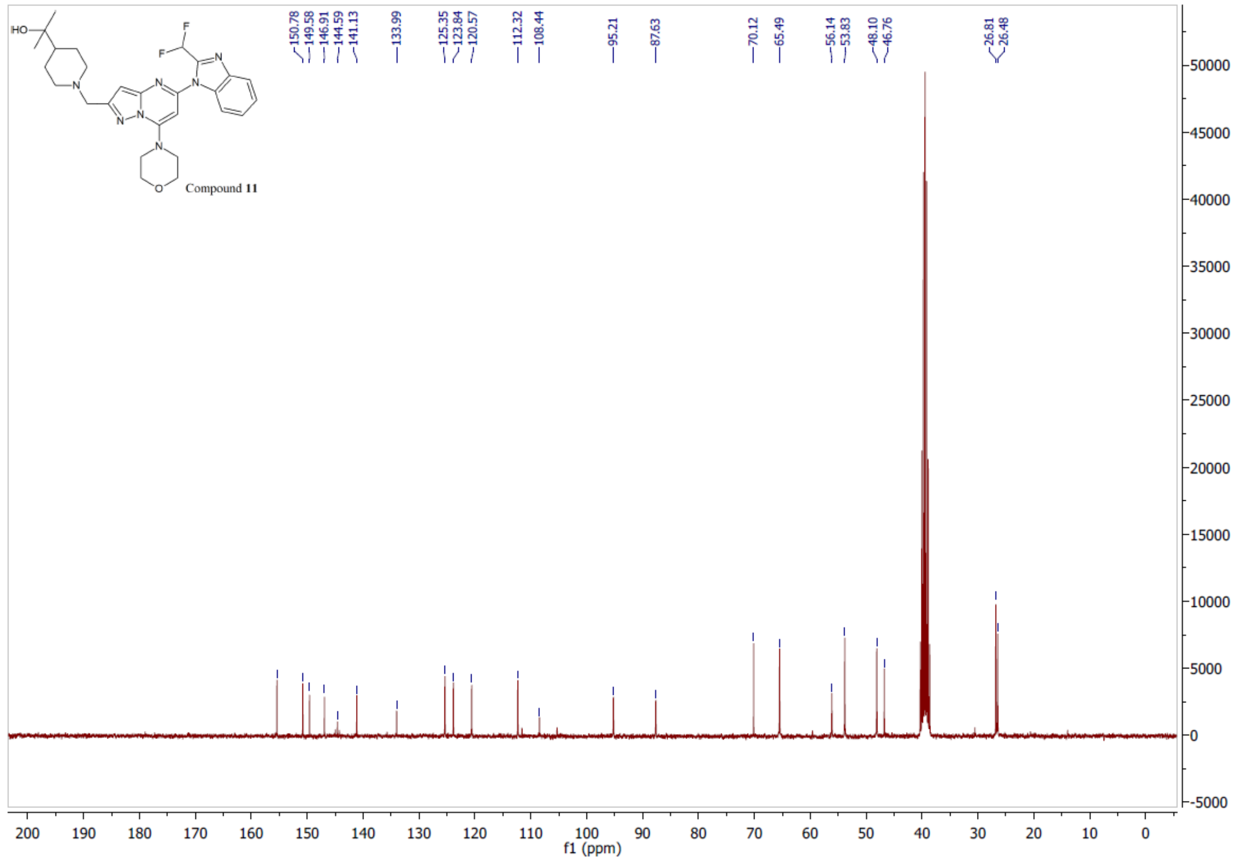

Compound 11

Name	*Obs. m/z*	Obs. RT	Obs. Mass	Tgt Formula	Tgt Mass	Tgt Mass Error	Find Cpds Algorit
Compound **11**	526.2764	1.16	525.2691	C27 H33 F2 N7 O2	525.2664	5.12	Find by Formula

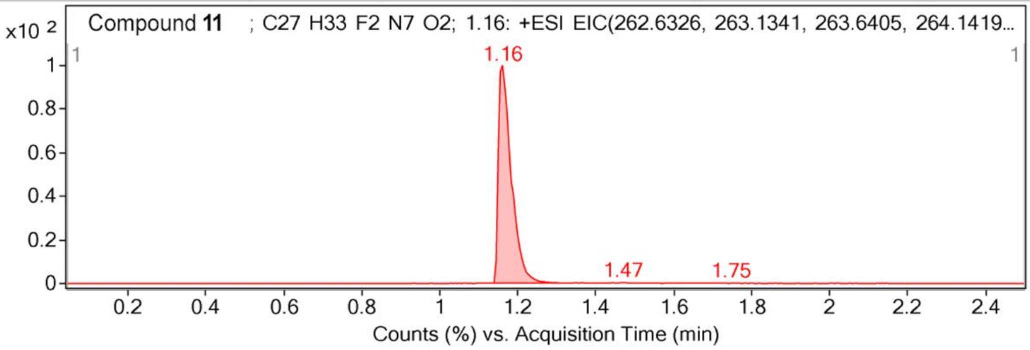

MS Zoomed Spectrum

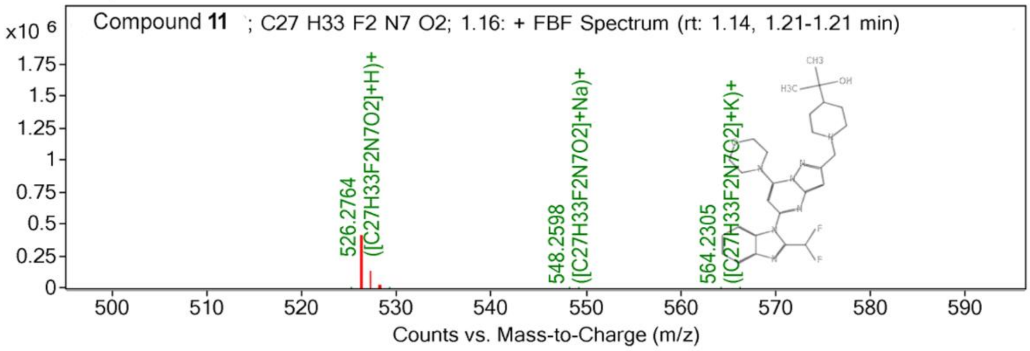

MS Zoomed Spectrum

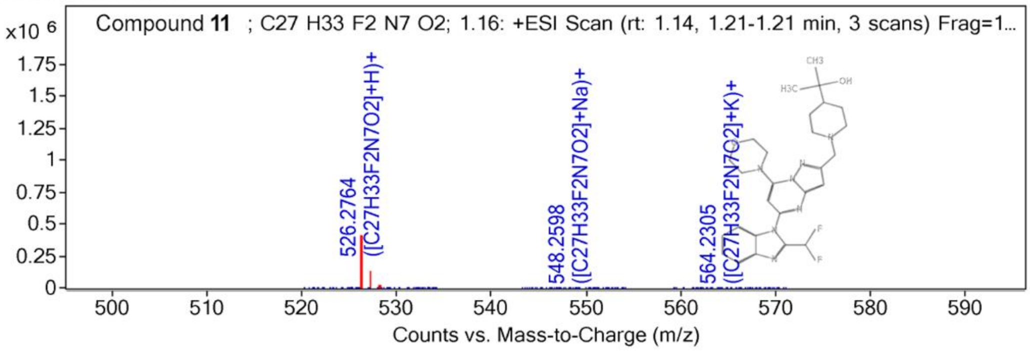

MS Spectrum Peak List

Obs. m/z	Charge	Abund	Formula	Ion/Isotope	Tgt Mass Error (ppm)
526.2764	1	404616.13	C27H33F2N7O2	(M+H)+	
526.2764	1	404616.13	C27H33F2N7O2	(M+H)+	5.14

--- End Of Report ---

Compound 12

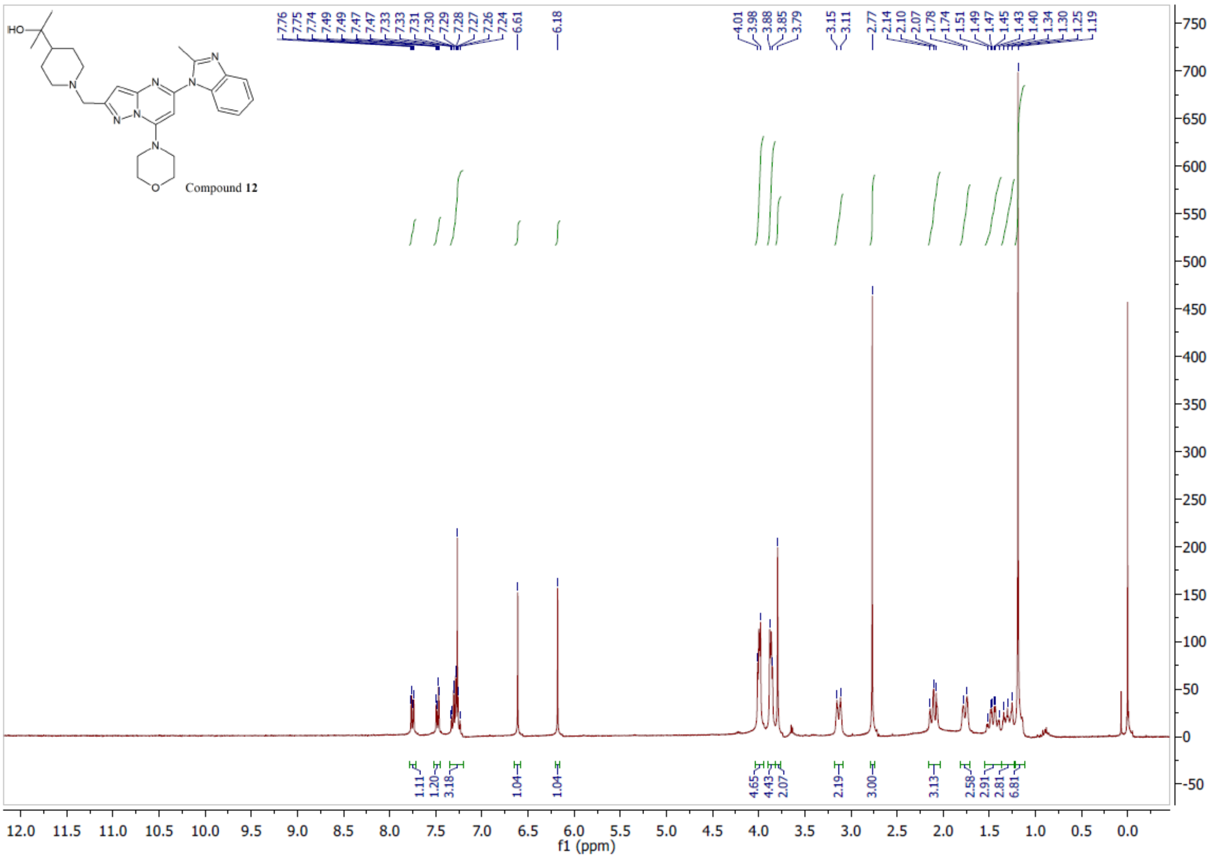
HO
N
Compound 12
7.76 7.75 7.74 7.49 7.49 7.47 7.47 7.33 7.33 7.31 7.30 7.29 7.28 7.27 7.26 7.24 6.61 6.18
4.01 3.98 3.88 3.85 3.79 3.15 3.11 2.77 2.14 2.10 2.07 1.78 1.74 1.51 1.49 1.47 1.45 1.43 1.40 1.34 1.30 1.25 1.19
1.11 1.20 3.18 1.04 1.04 4.65 4.43 2.07 2.19 3.00 3.13 2.58 2.91 2.81 6.81
f1 (ppm)

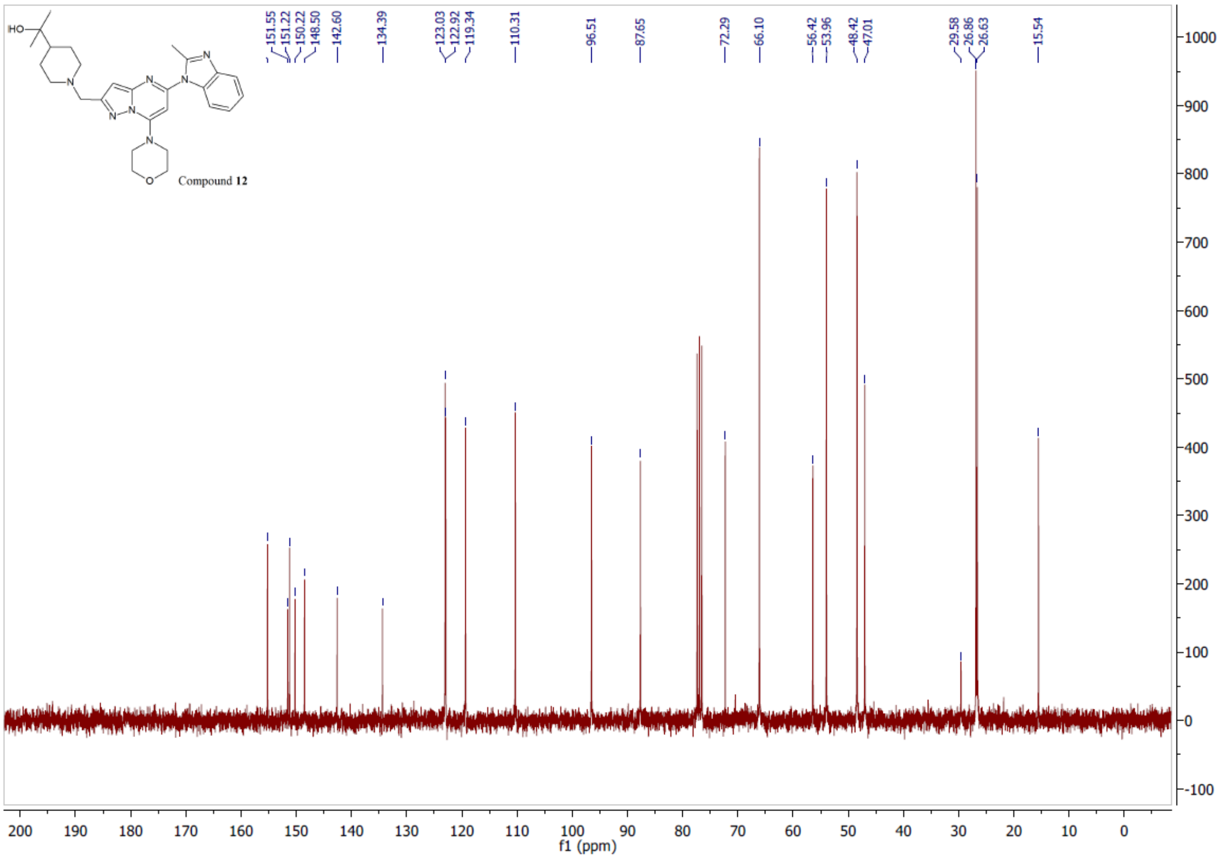
HO
N
Compound 12
151.55 151.22 150.22 148.50 142.60 134.39 123.03 122.92 119.34 110.31 96.51 87.65 72.29 66.10 56.42 53.96 48.42 47.01 29.58 26.86 26.63 15.54
f1 (ppm)

Compound 12

Compound Label	Name	*m/z*	RT	Algorithm	Mass
Compound **12**	Compound **12**	245.65023	1.09	Find by Formula	489.28538

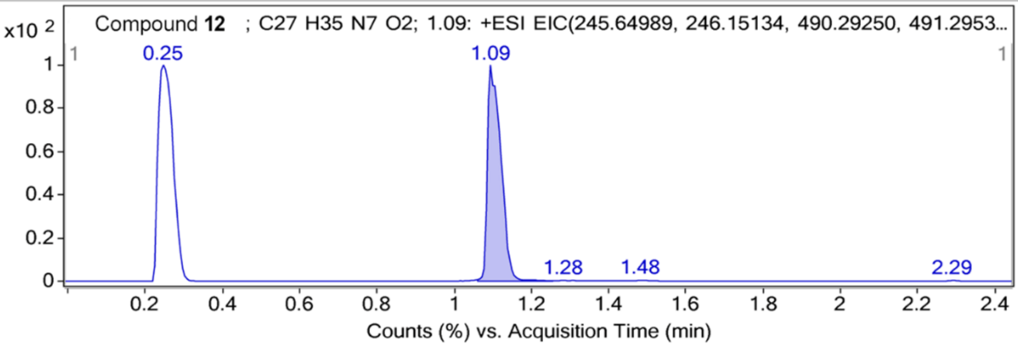

MS Zoomed Spectrum

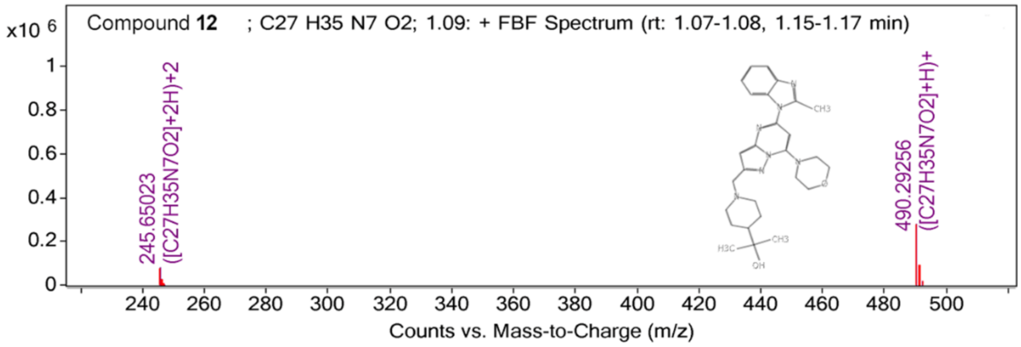

MS Zoomed Spectrum

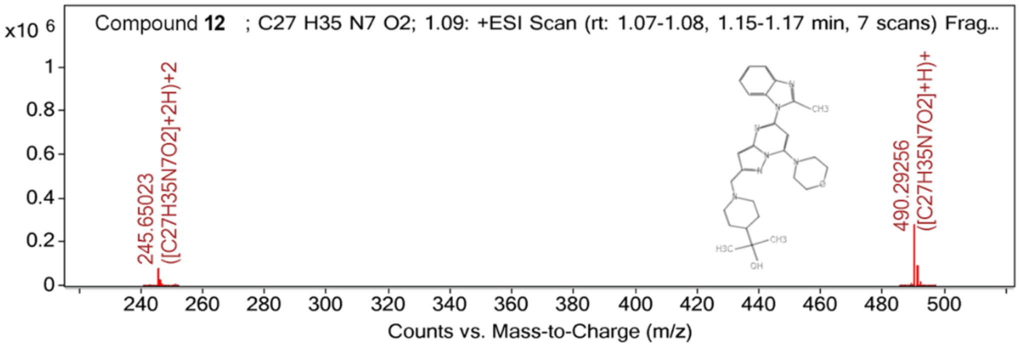

MS Spectrum Peak List

m/z	*Calc m/z*	Diff(ppm)	z	Abund	Formula	Ion
245.65023	245.64989	1.38	2	79116.77	C27H35N7O2	(M+2H)+2
246.15176	246.15134	1.74	2	26046.49	C27H35N7O2	(M+2H)+2
246.65239	246.65272	-1.31	2	4306.85	C27H35N7O2	(M+2H)+2
247.15487	247.15405	3.34	2	683.01	C27H35N7O2	(M+2H)+2
490.29256	490.2925	0.12	1	277845.75	C27H35N7O2	(M+H)+
491.29534	491.29539	-0.1	1	84677.91	C27H35N7O2	(M+H)+
492.29744	492.29815	-1.45	1	16256.98	C27H35N7O2	(M+H)+

--- End Of Report ---

Compound 16

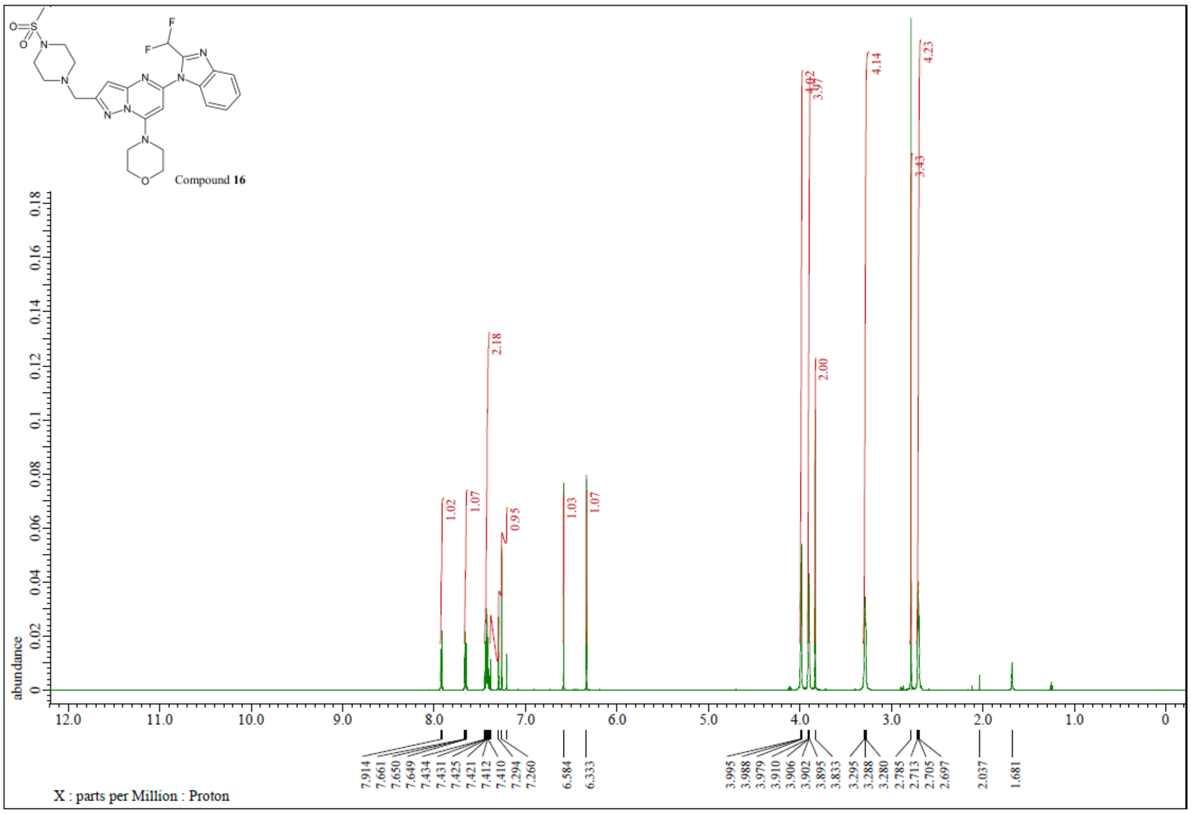
Compound 16
abundance
X : parts per Million : Proton

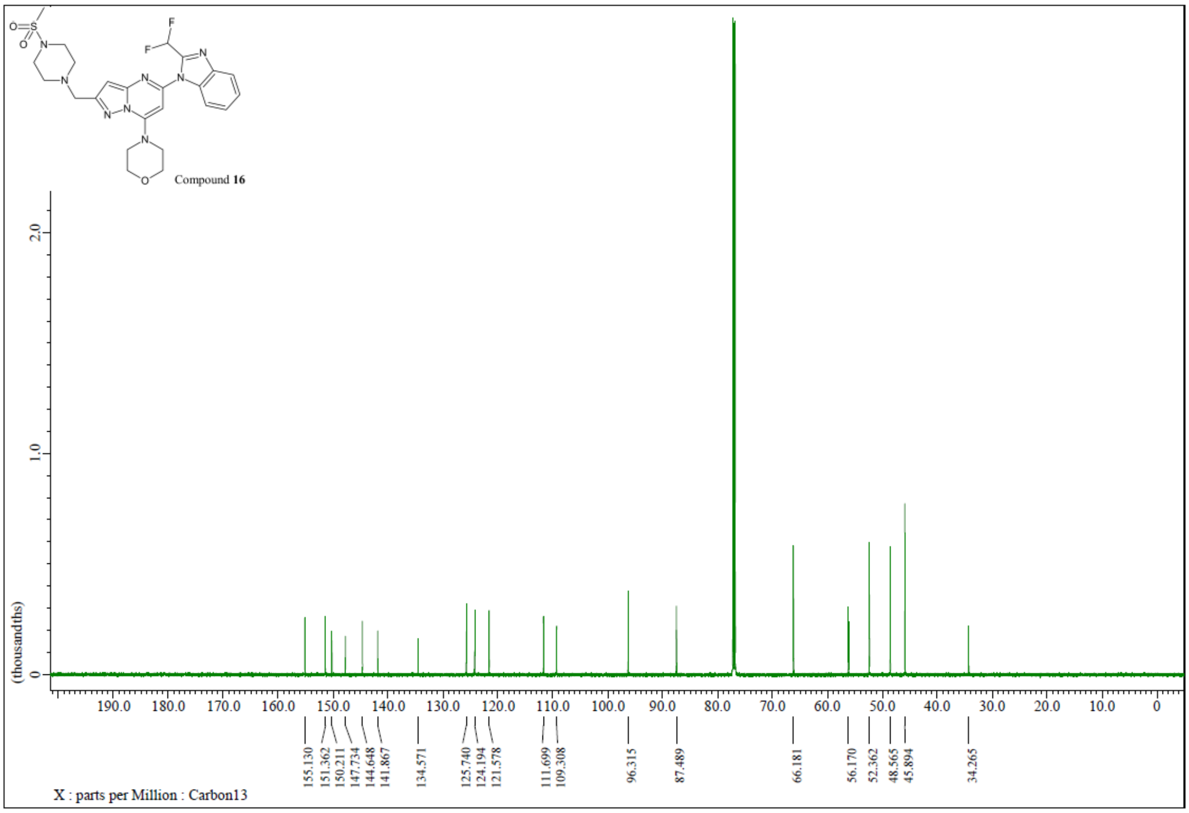
Compound 16
(thousandths)
X : parts per Million : Carbon13

Compound 16

Compound Label	Name	*m/z*	RT	Algorithm	Mass
Compound **16**	Compound **16**	547.20487	1.19	Find by Formula	546.19768

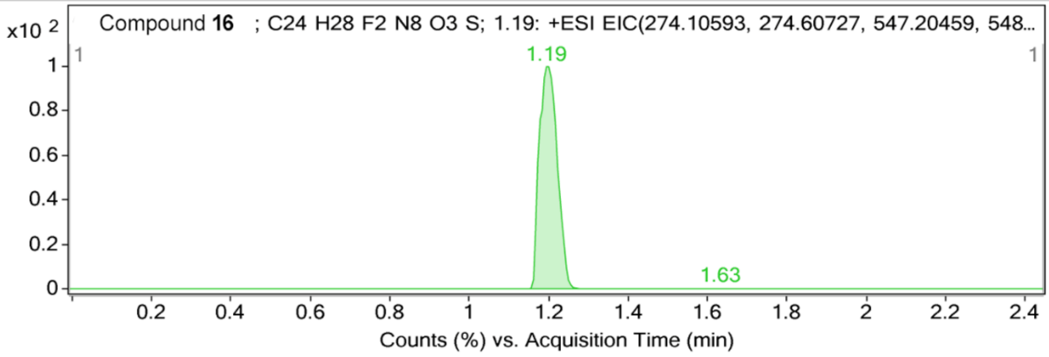

MS Zoomed Spectrum

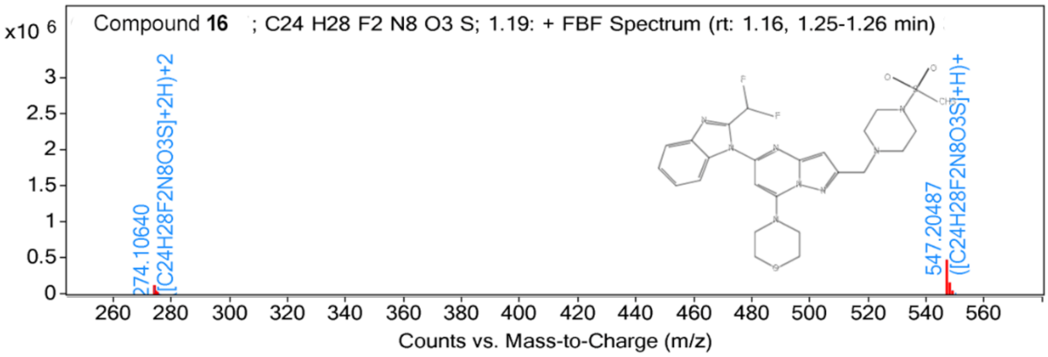

MS Zoomed Spectrum

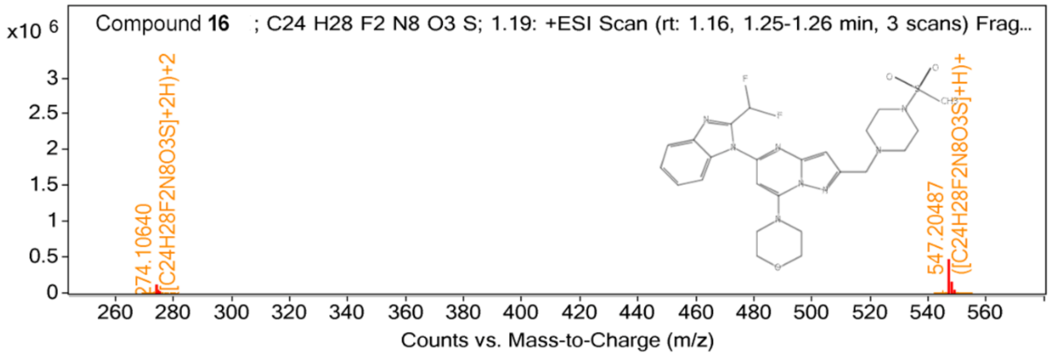

MS Spectrum Peak List

m/z	*Calc m/z*	Diff(ppm)	z	Abund	Formula	Ion
274.1064	274.10593	1.68	2	105049.91	C24H28F2N8O3S	(M+2H)+2
274.60756	274.60727	1.04	2	33865.99	C24H28F2N8O3S	(M+2H)+2
275.1073	275.10631	3.58	2	10859.48	C24H28F2N8O3S	(M+2H)+2
275.60631	275.60663	-1.16	2	2302.53	C24H28F2N8O3S	(M+2H)+2
547.20487	547.20459	0.52	1	465451.59	C24H28F2N8O3S	(M+H)+
548.2075	548.20726	0.44	1	130005.94	C24H28F2N8O3S	(M+H)+
549.20534	549.20534	-0.01	1	38941.63	C24H28F2N8O3S	(M+H)+
550.20543	550.20598	-1	1	8620.2	C24H28F2N8O3S	(M+H)+

--- End Of Report ---

Compound 17

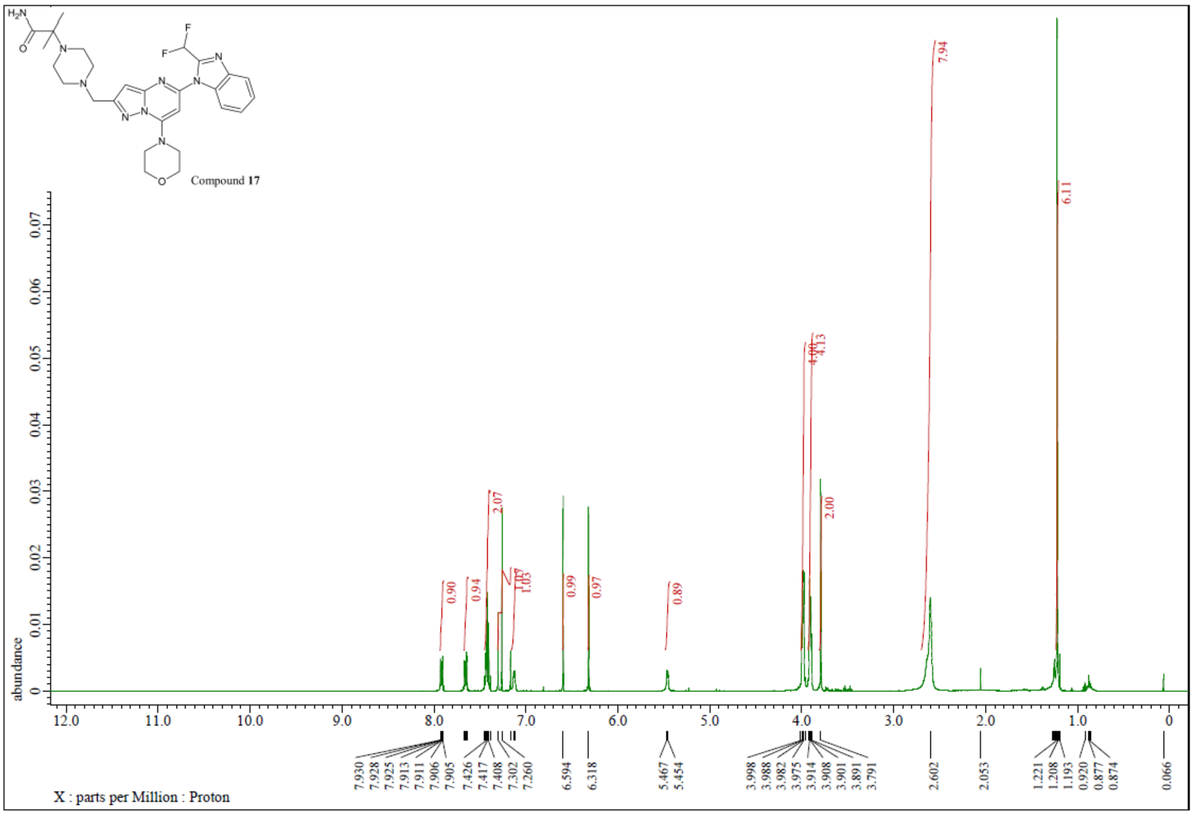
Compound 17
abundance
X : parts per Million : Proton
7.930
7.928
7.925
7.913
7.911
7.906
7.905
7.426
7.417
7.408
7.302
7.260
6.594
6.318
5.467
5.454
3.998
3.988
3.982
3.975
3.914
3.908
3.901
3.891
3.791
2.602
2.053
1.221
1.208
1.193
0.920
0.877
0.874
0.066

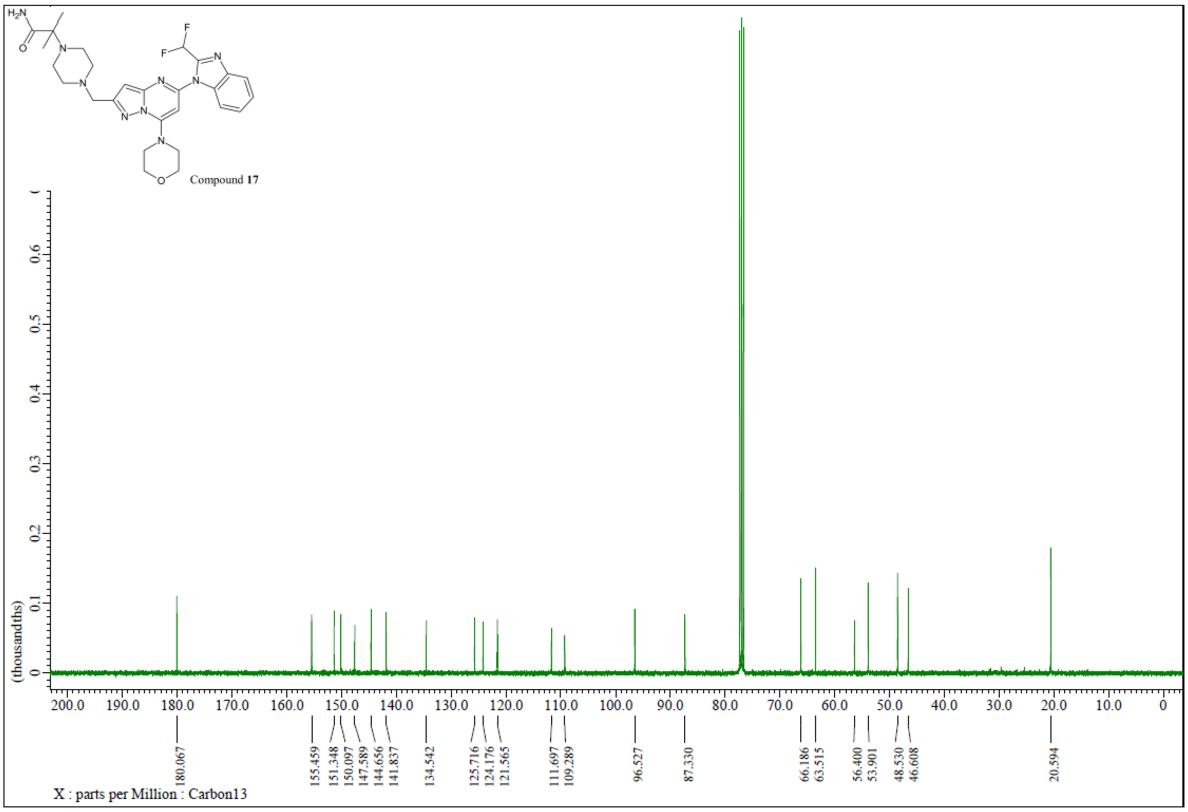
Compound 17
(thousandths)
X : parts per Million : Carbon13
180.067
155.459
151.348
150.097
147.589
144.656
141.837
134.542
125.716
124.176
121.565
111.697
109.289
96.527
87.330
66.186
63.515
56.400
53.901
48.530
46.608
20.594

Compound 17

Name	*Obs. m/z*	Obs. RT	Obs. Mass	Tgt Formula	Tgt Mass	Tgt Mass Error	Find Cpds Algorit
Compound **17**	554.28	1.24	553.27265	C27 H33 F2 N9 O2	553.27253	0.23	Find by Formula

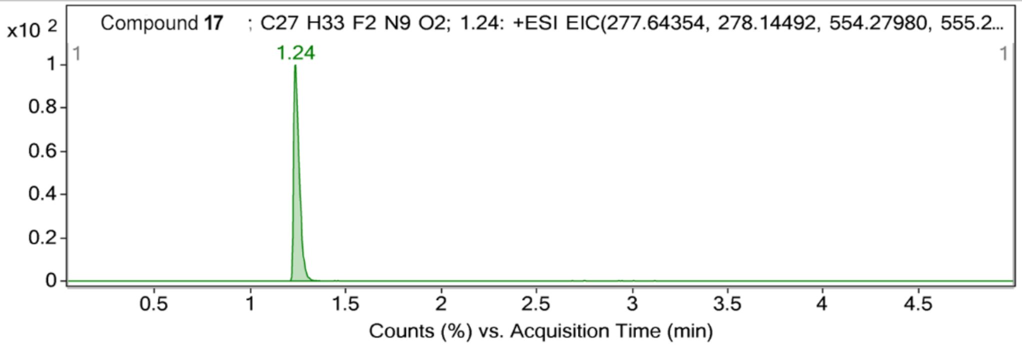

MS Zoomed Spectrum

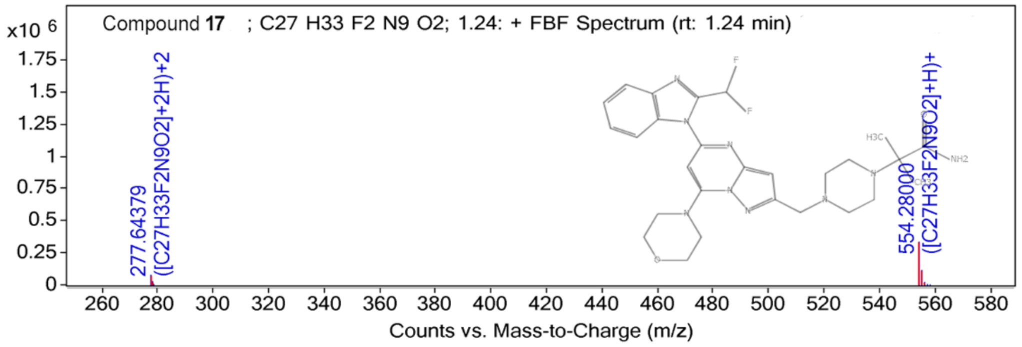

MS Zoomed Spectrum

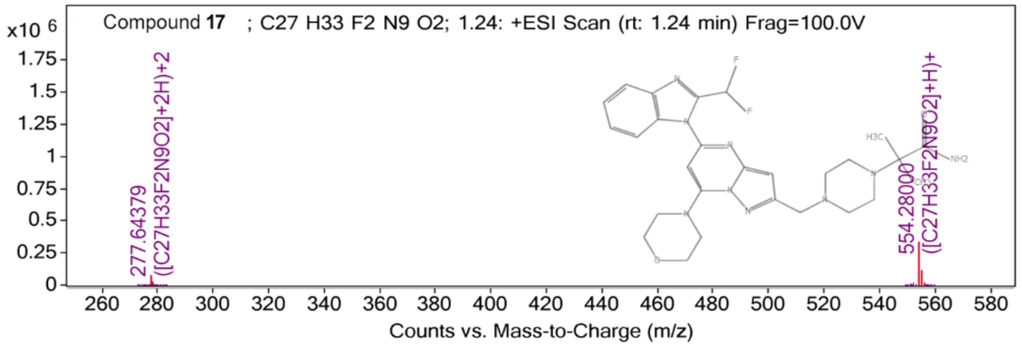

MS Spectrum Peak List

Obs. m/z	Charge	Abund	Formula	Ion/Isotope	Tgt Mass Error (ppm)
277.64379	2	74236.3	C27H33F2N9O2	(M+2H)+2	
278.14494	2	24665.33	C27H33F2N9O2	(M+2H)+2	
278.64678	2	3142.95	C27H33F2N9O2	(M+2H)+2	
554.28	1	331615.38	C27H33F2N9O2	(M+H)+	
555.28243	1	113299.23	C27H33F2N9O2	(M+H)+	
556.28444	1	17136.88	C27H33F2N9O2	(M+H)+	
557.28512	1	1819.13	C27H33F2N9O2	(M+H)+	
558.2883	1	234.87	C27H33F2N9O2	(M+H)+	
277.64379	2	74236.3	C27H33F2N9O2	(M+2H)+2	0.91
278.14494	2	24665.33	C27H33F2N9O2	(M+2H)+2	0.07
278.64678	2	3142.95	C27H33F2N9O2	(M+2H)+2	1.91
554.28	1	331615.38	C27H33F2N9O2	(M+H)+	0.35
555.28243	1	113299.23	C27H33F2N9O2	(M+H)+	-0.25
556.28444	1	17136.88	C27H33F2N9O2	(M+H)+	-1.38
557.28512	1	1819.13	C27H33F2N9O2	(M+H)+	-4.77
558.2883	1	234.87	C27H33F2N9O2	(M+H)+	-3.57

--- End Of Report ---

Compound 18

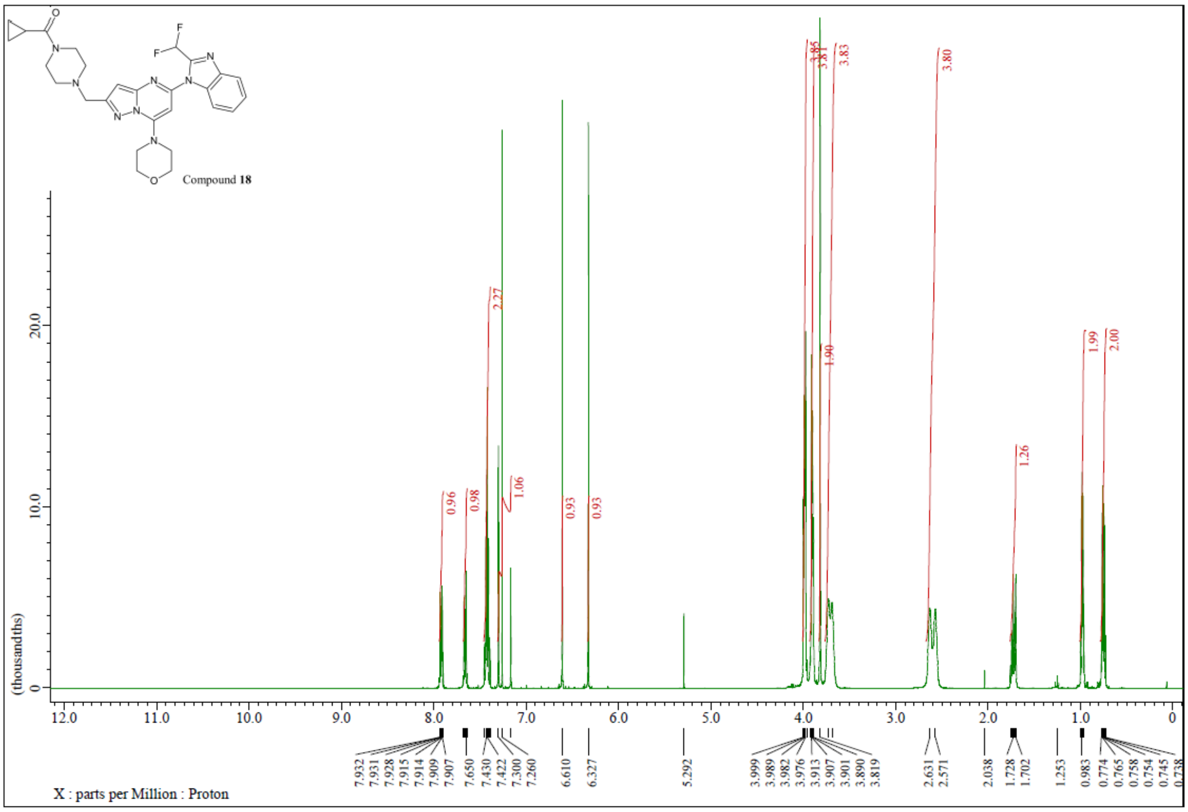
Compound 18
X : parts per Million : Proton
(thousandths)

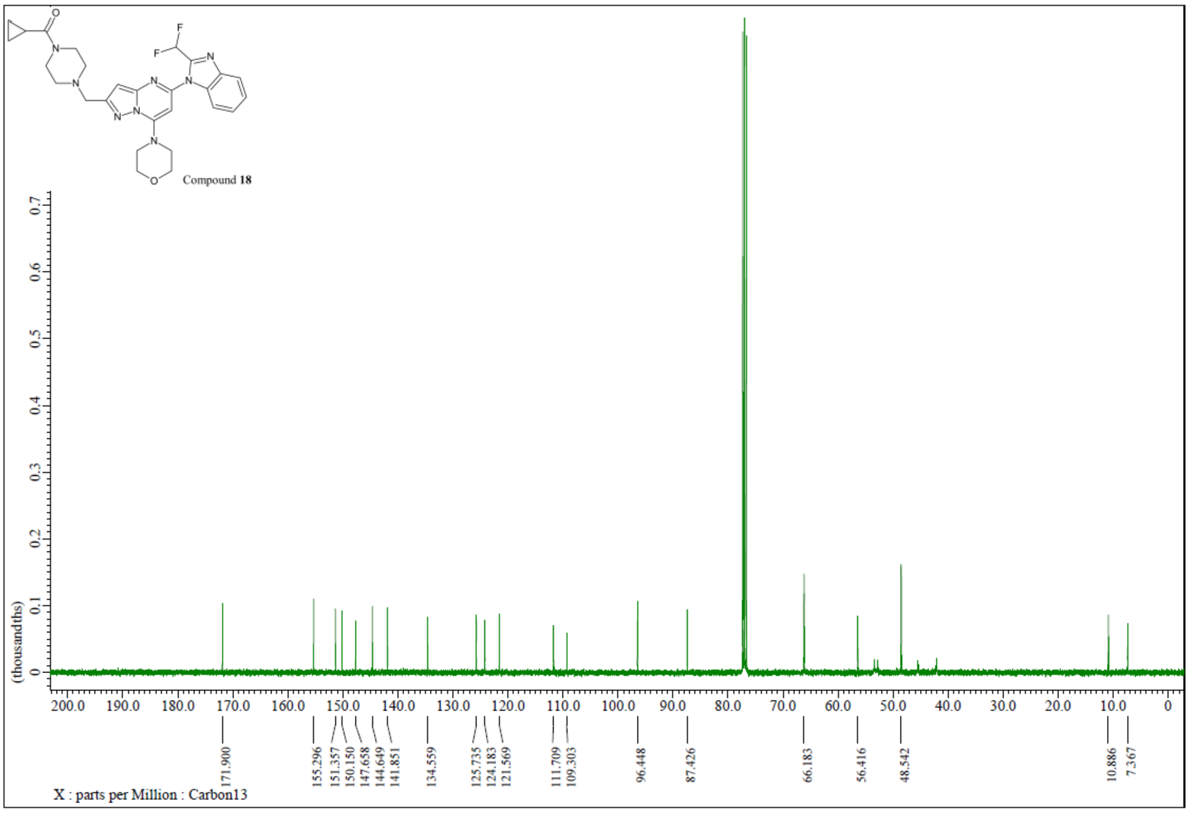
Compound 18
X : parts per Million : Carbon13
(thousandths)

Compound 18

Name	*Obs. m/z*	Obs. RT	Obs. Mass	Tgt Formula	Tgt Mass	Tgt Mass Error	Find Cpds Algorit
Compound **18**	269.13057	1.31	536.24666	C27 H30 F2 N8 O2	536.24598	1.28	Find by Formula

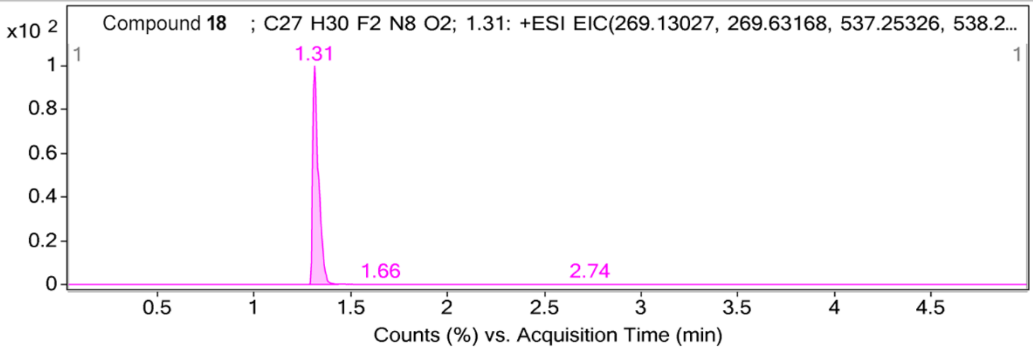

MS Zoomed Spectrum

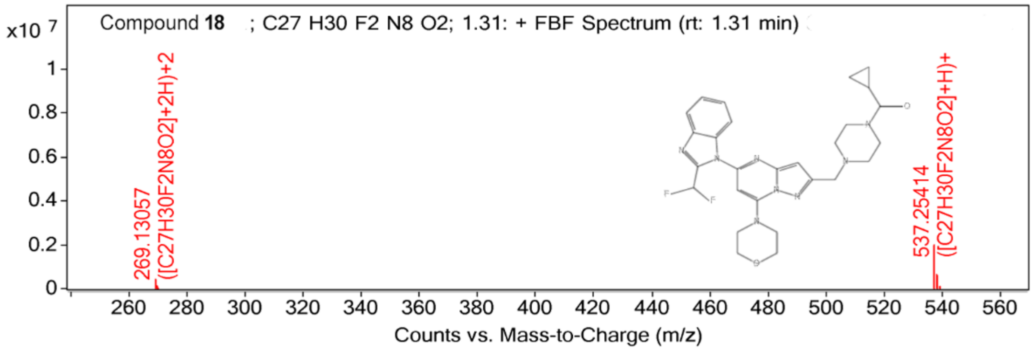

MS Zoomed Spectrum

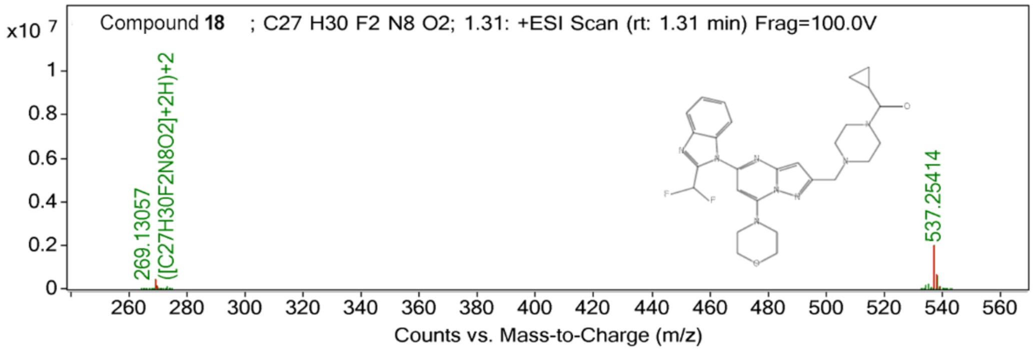

MS Spectrum Peak List

Obs. m/z	Charge	Abund	Formula	Ion/Isotope	Tgt Mass Error (ppm)
269.13057	2	425649.31	C27H30F2N8O2	(M+2H)+2	
269.63216	2	122896.92	C27H30F2N8O2	(M+2H)+2	
270.13329	2	19970.75	C27H30F2N8O2	(M+2H)+2	
537.25414	1	1985956.63	C27H30F2N8O2	(M+H)+	
538.25617	1	592174.13	C27H30F2N8O2	(M+H)+	
539.25893	1	86057.74	C27H30F2N8O2	(M+H)+	
269.13057	2	425649.31	C27H30F2N8O2	(M+2H)+2	1.14
269.63216	2	122896.92	C27H30F2N8O2	(M+2H)+2	1.78
270.13329	2	19970.75	C27H30F2N8O2	(M+2H)+2	0.96
537.25414	1	1985956.63	C27H30F2N8O2	(M+H)+	1.64
537.25414		1988175.75			
538.25617	1	592174.13	C27H30F2N8O2	(M+H)+	0.17
539.25893	1	86057.74	C27H30F2N8O2	(M+H)+	0.29

--- End Of Report ---

Compound 19

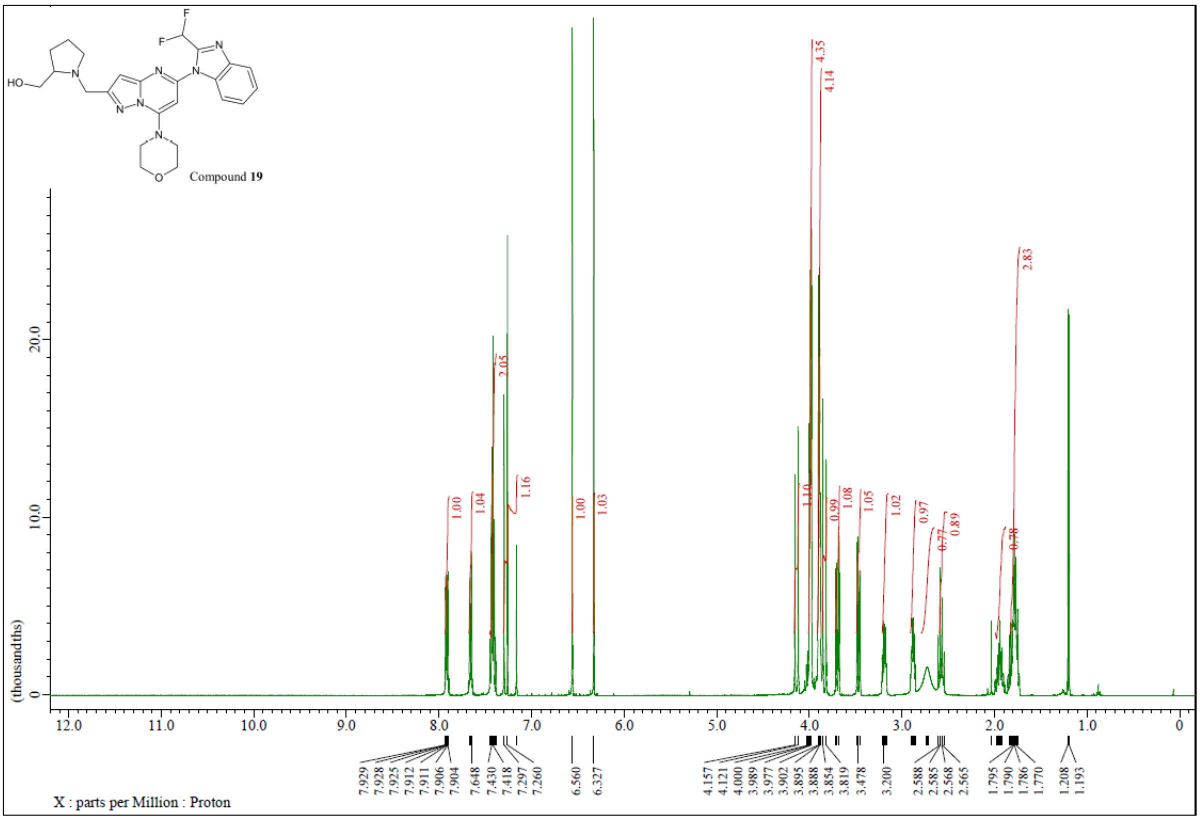
Compound 19
X : parts per Million : Proton
(thousandths)

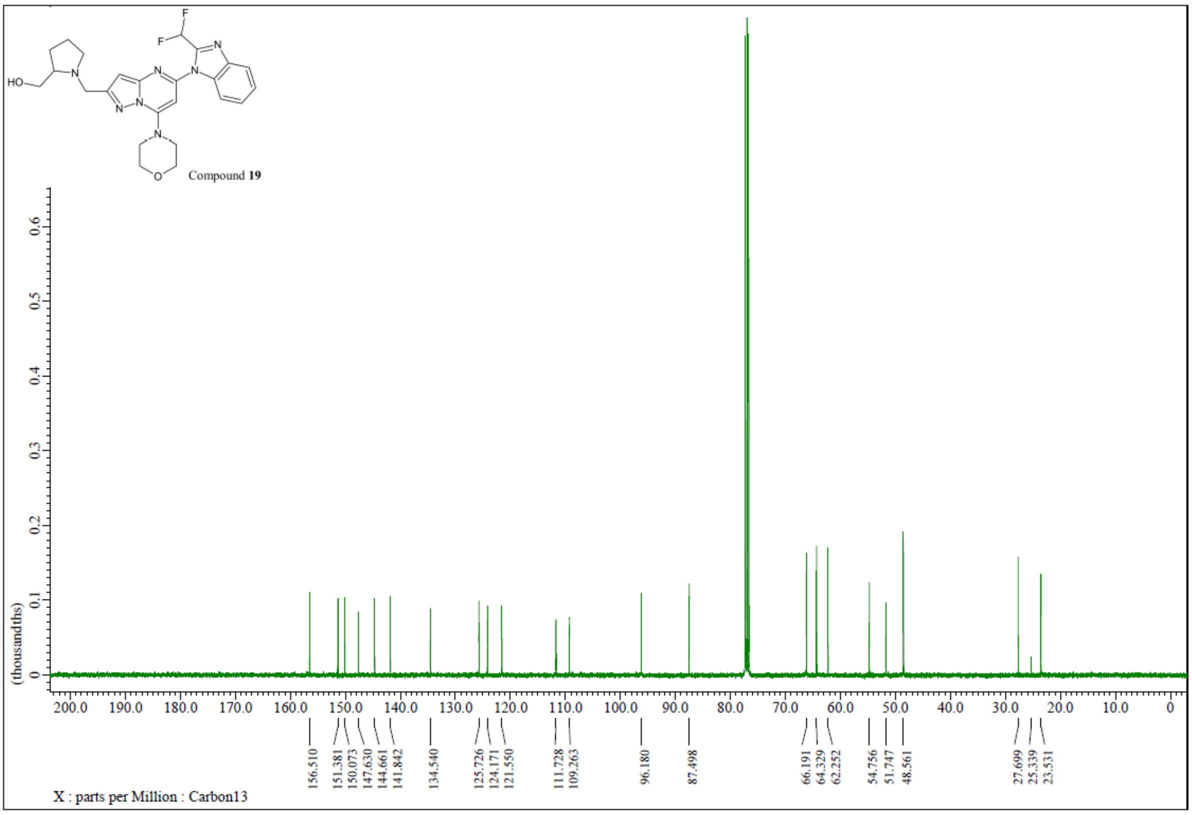
Compound 19
X : parts per Million : Carbon13
(thousandths)

Compound 19

Name	*Obs. m/z*	Obs. RT	Obs. Mass	Tgt Formula	Tgt Mass	Tgt Mass Error	Find Cpds Algorit
Compound **19**	484.22718	1.23	483.21997	C24 H27 F2 N7 O2	483.21943	1.11	Find by Formula

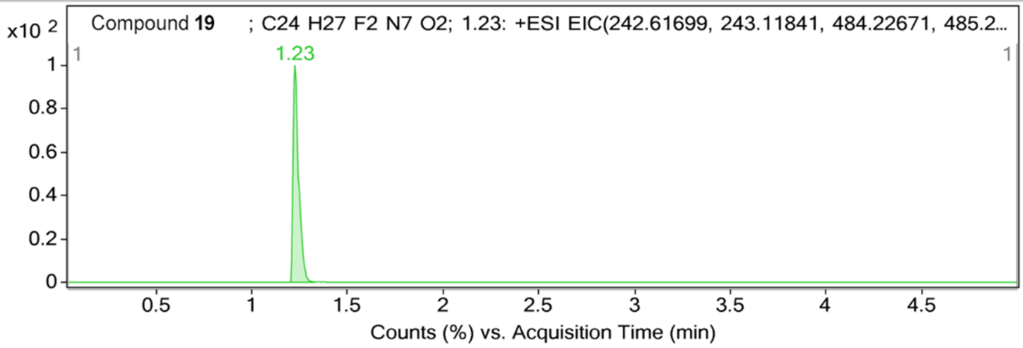

MS Zoomed Spectrum

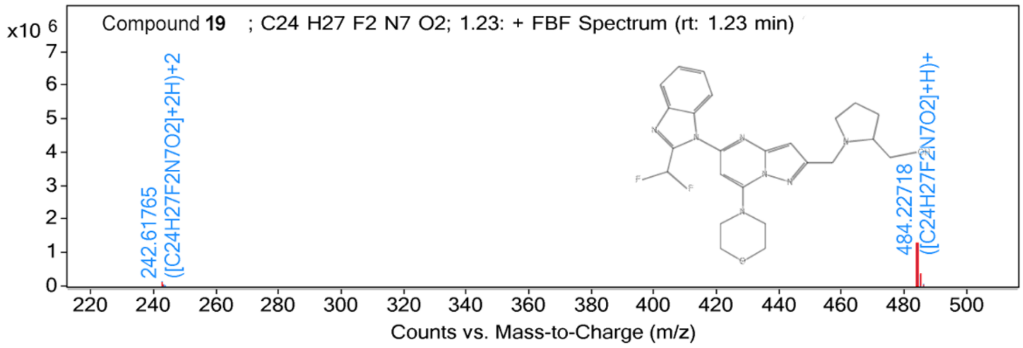

MS Zoomed Spectrum

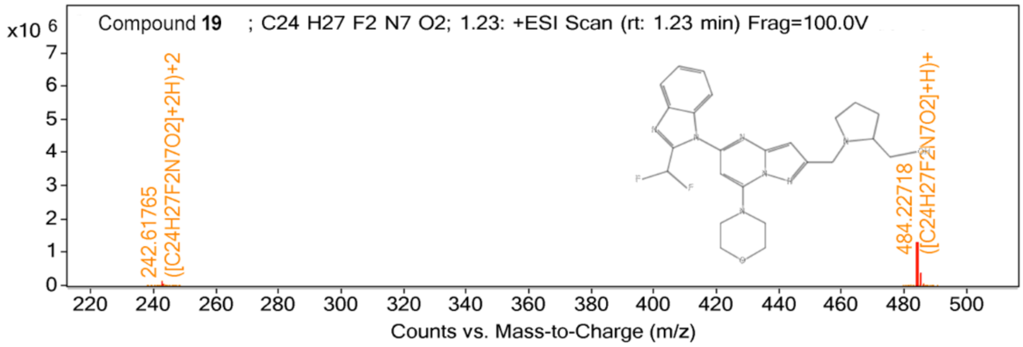

MS Spectrum Peak List

Obs. m/z	Charge	Abund	Formula	Ion/Isotope	Tgt Mass Error (ppm)
242.61765	2	128896.13	C24H27F2N7O2	(M+2H)+2	
243.11881	2	36127.81	C24H27F2N7O2	(M+2H)+2	
243.62073	2	7105.86	C24H27F2N7O2	(M+2H)+2	
484.22718	1	1284613.5	C24H27F2N7O2	(M+H)+	
485.23003	1	333370.47	C24H27F2N7O2	(M+H)+	
486.23205	1	49131.52	C24H27F2N7O2	(M+H)+	
242.61765	2	128896.13	C24H27F2N7O2	(M+2H)+2	2.73
243.11881	2	36127.81	C24H27F2N7O2	(M+2H)+2	1.66
243.62073	2	7105.86	C24H27F2N7O2	(M+2H)+2	4.03
484.22718	1	1284613.5	C24H27F2N7O2	(M+H)+	0.99
485.23003	1	333370.47	C24H27F2N7O2	(M+H)+	1.02
486.23205	1	49131.52	C24H27F2N7O2	(M+H)+	-0.34

--- End Of Report ---

Compound 20

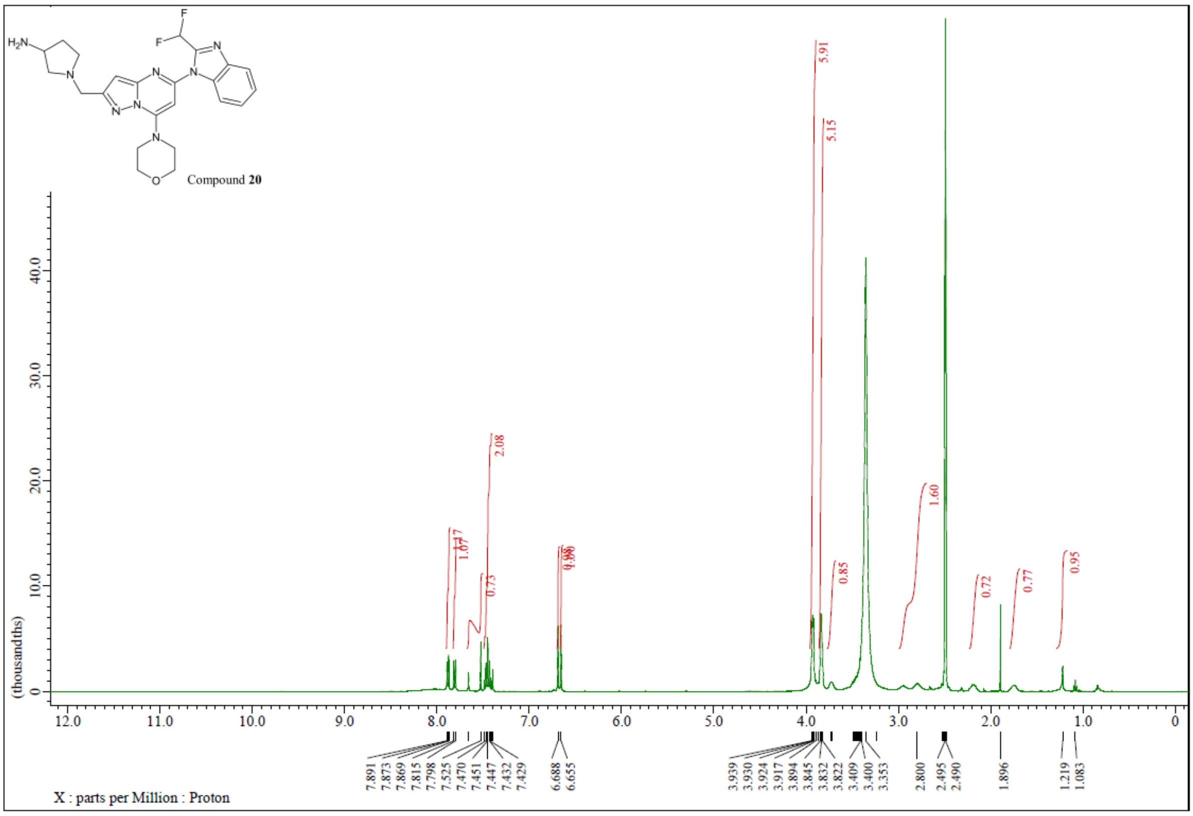

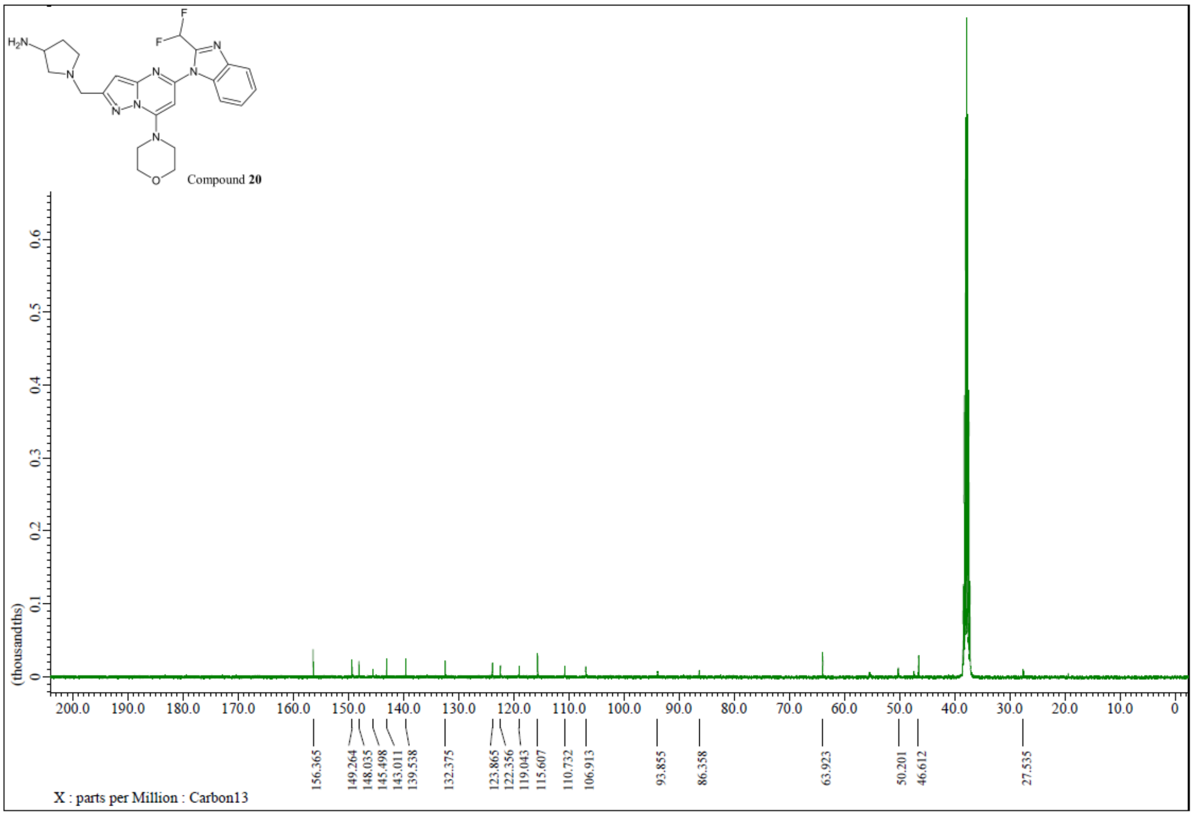

Compound 20

Name	*Obs. m/z*	Obs. RT	Obs. Mass	Tgt Formula	Tgt Mass	Tgt Mass Error	Find Cpds Algorit
Compound **20**	235.11741	0.887	468.22001	C23 H26 F2 N8 O	468.21976	0.54	Find by Formula

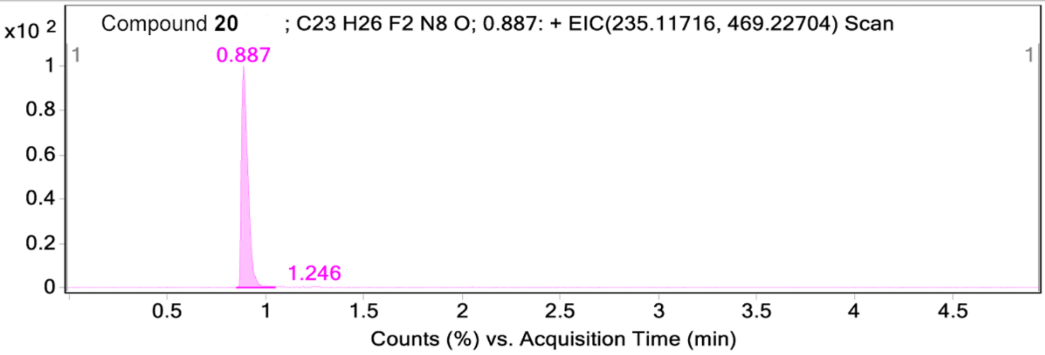

MS Zoomed Spectrum

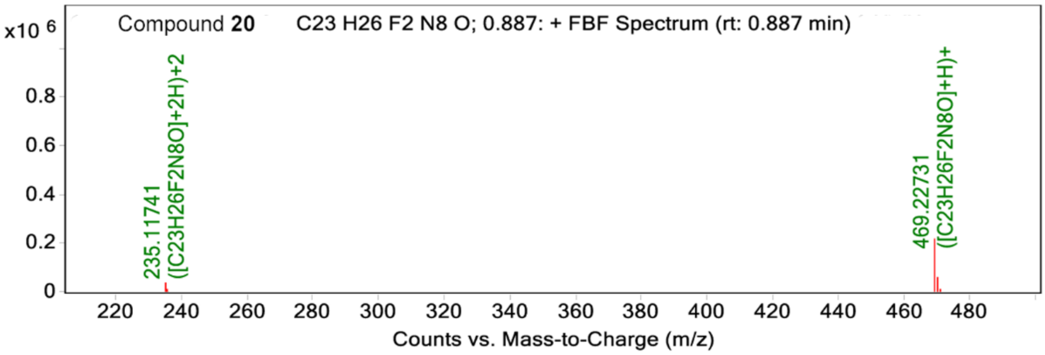

MS Zoomed Spectrum

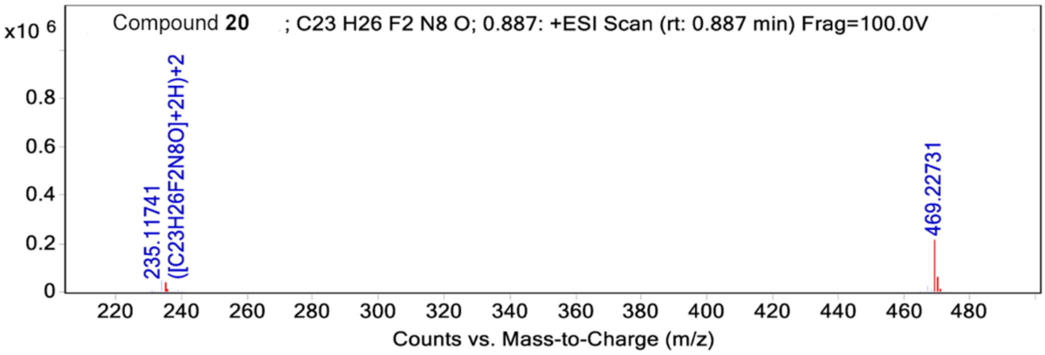

MS Spectrum Peak List

Obs. m/z	Charge	Abund	Formula	Ion/Isotope	Tgt Mass Error (ppm)
235.11741	2	37955.73	C23H26F2N8O	(M+2H)+2	
235.61897	2	10801.02	C23H26F2N8O	(M+2H)+2	
236.12072	2	1863.32	C23H26F2N8O	(M+2H)+2	
469.22731	1	217069.95	C23H26F2N8O	(M+H)+	
470.22977	1	53954.65	C23H26F2N8O	(M+H)+	
471.23136	1	7749.8	C23H26F2N8O	(M+H)+	
235.11741	2	37955.73	C23H26F2N8O	(M+2H)+2	1.06
235.61897	2	10801.02	C23H26F2N8O	(M+2H)+2	1.88
236.12072	2	1863.32	C23H26F2N8O	(M+2H)+2	3.7
469.22731		217402.84			0.58
469.22731	1	217069.95	C23H26F2N8O	(M+H)+	0.58
470.22977	1	53954.65	C23H26F2N8O	(M+H)+	-0.01
471.23136	1	7749.8	C23H26F2N8O	(M+H)+	-2.23

--- End Of Report ---

Compound 21

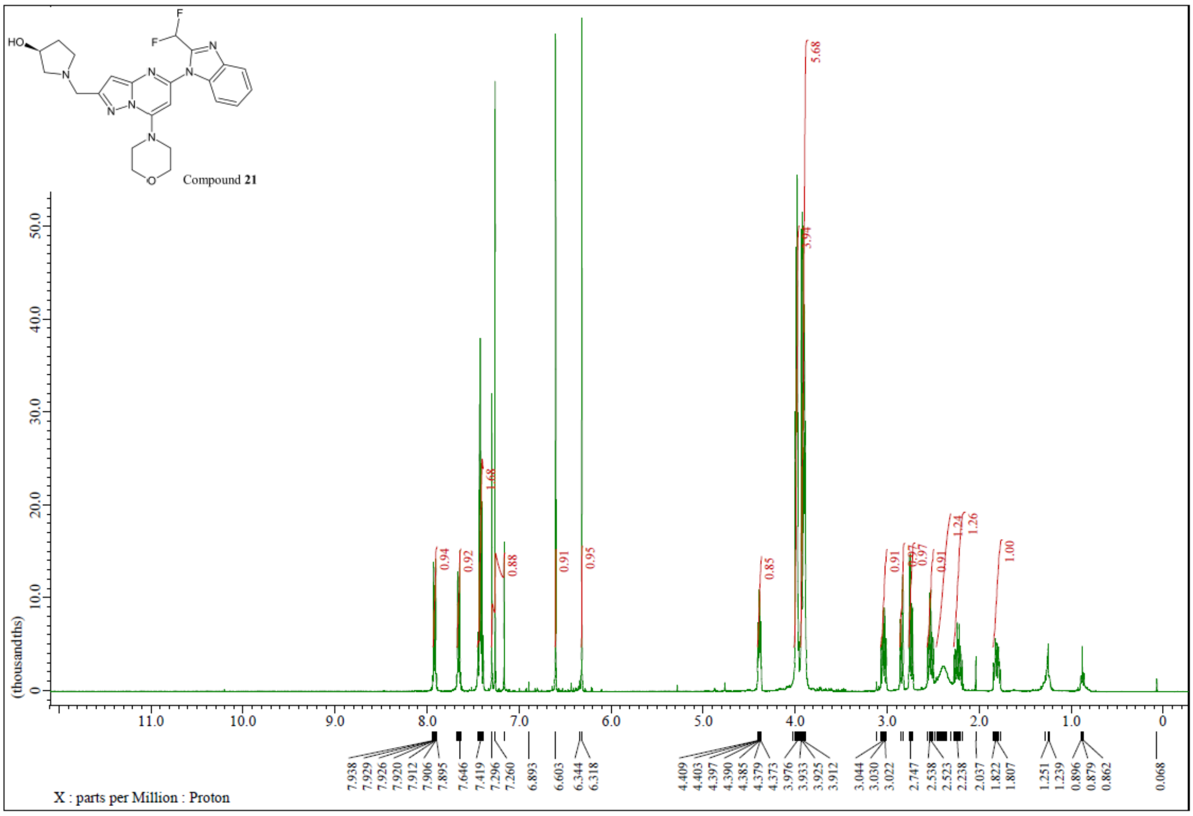
HO
F
F
N
N
N
N
N
N
O
Compound 21
(thousandths)
X : parts per Million : Proton
5.68
3.94
1.68
0.94
0.92
0.88
0.91
0.95
0.85
0.91
0.97
0.97
0.91
1.24
1.26
1.00
7.938
7.929
7.926
7.920
7.912
7.906
7.895
7.646
7.419
7.296
7.260
6.893
6.603
6.344
6.318
4.409
4.403
4.397
4.390
4.385
4.379
4.373
3.976
3.933
3.925
3.912
3.044
3.030
3.022
2.747
2.538
2.523
2.238
2.037
1.822
1.807
1.251
1.239
0.896
0.879
0.862
0.068

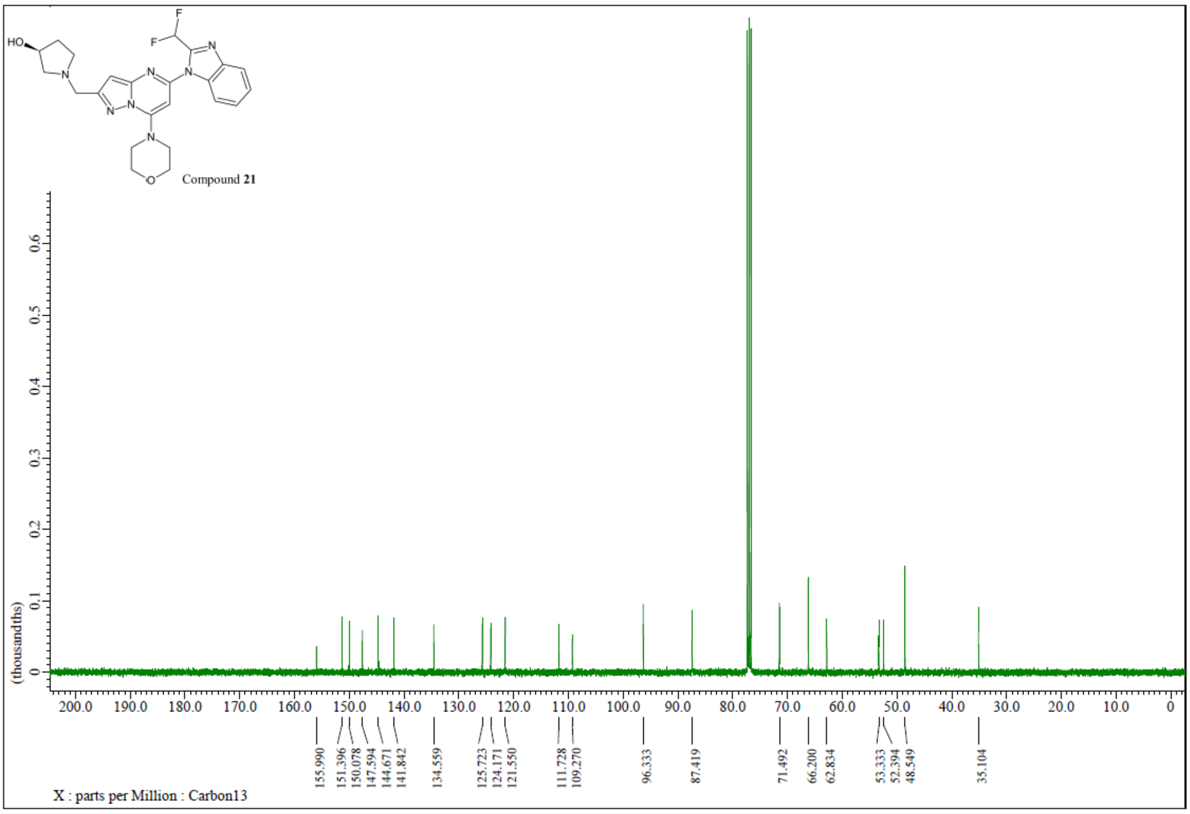
HO
F
F
N
N
N
N
N
N
O
Compound 21
(thousandths)
X : parts per Million : Carbon13
155.990
151.396
150.078
147.594
144.671
141.842
134.559
125.723
124.171
121.550
111.728
109.270
96.333
87.419
71.492
66.200
62.834
53.333
52.394
48.549
35.104

Compound 21

Name	*Obs. m/z*	Obs. RT	Obs. Mass	Tgt Formula	Tgt Mass	Tgt Mass Error	Find Cpds Algorith
Compound **21**	235.60959	1.18	469.20435	C23 H25 F2 N7 O2	469.20378	1.21	Find by Formula

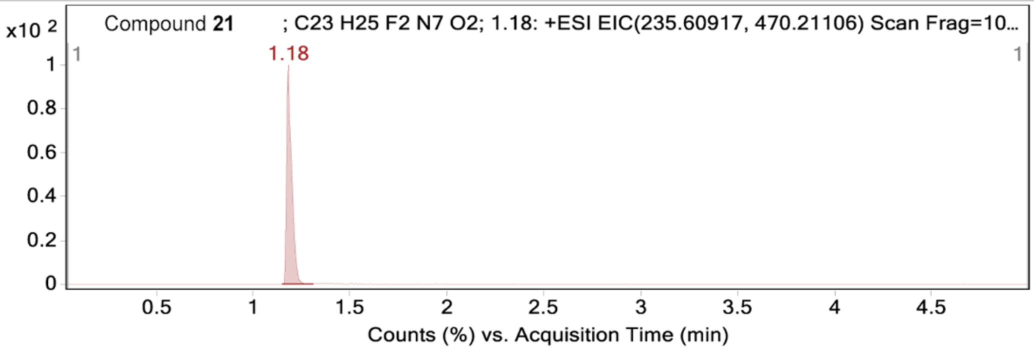

MS Zoomed Spectrum

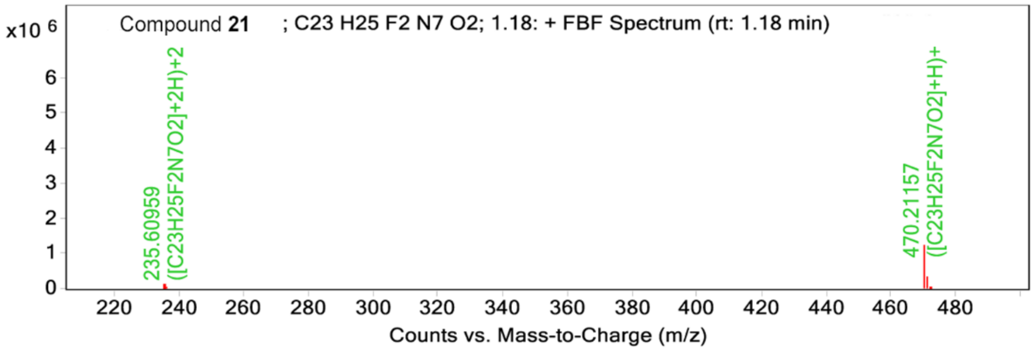

MS Zoomed Spectrum

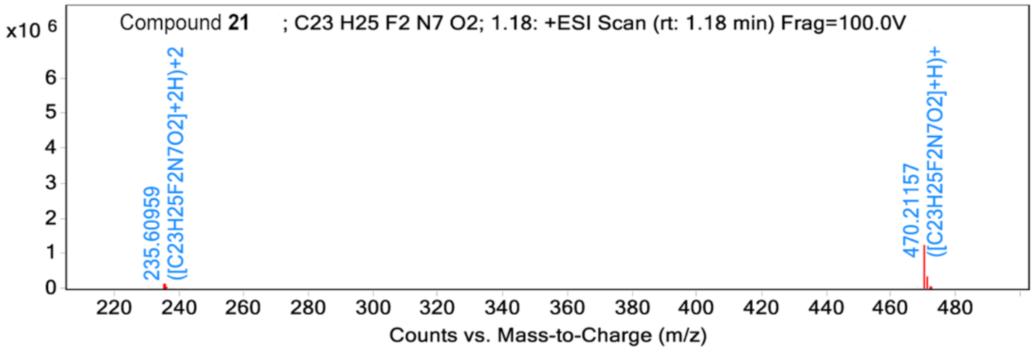

MS Spectrum Peak List

Obs. m/z	Charge	Abund	Formula	Ion/Isotope	Tgt Mass Error (ppm)
235.60959	2	125434.16	C23H25F2N7O2	(M+2H)+2	
236.11085	2	38404.56	C23H25F2N7O2	(M+2H)+2	
236.61302	2	4518.14	C23H25F2N7O2	(M+2H)+2	
237.11164	2	465.8	C23H25F2N7O2	(M+2H)+2	
470.21157	1	1232616.63	C23H25F2N7O2	(M+H)+	
471.21462	1	290558.94	C23H25F2N7O2	(M+H)+	
472.21628	1	40423.04	C23H25F2N7O2	(M+H)+	
235.60959	2	125434.16	C23H25F2N7O2	(M+2H)+2	1.79
236.11085	2	38404.56	C23H25F2N7O2	(M+2H)+2	1.19
236.61302	2	4518.14	C23H25F2N7O2	(M+2H)+2	4.73
237.11164	2	465.8	C23H25F2N7O2	(M+2H)+2	-6.44
470.21157	1	1232616.63	C23H25F2N7O2	(M+H)+	1.1
471.21462	1	290558.94	C23H25F2N7O2	(M+H)+	1.6
472.21628	1	40423.04	C23H25F2N7O2	(M+H)+	-0.49

--- End Of Report ---

Compound 22

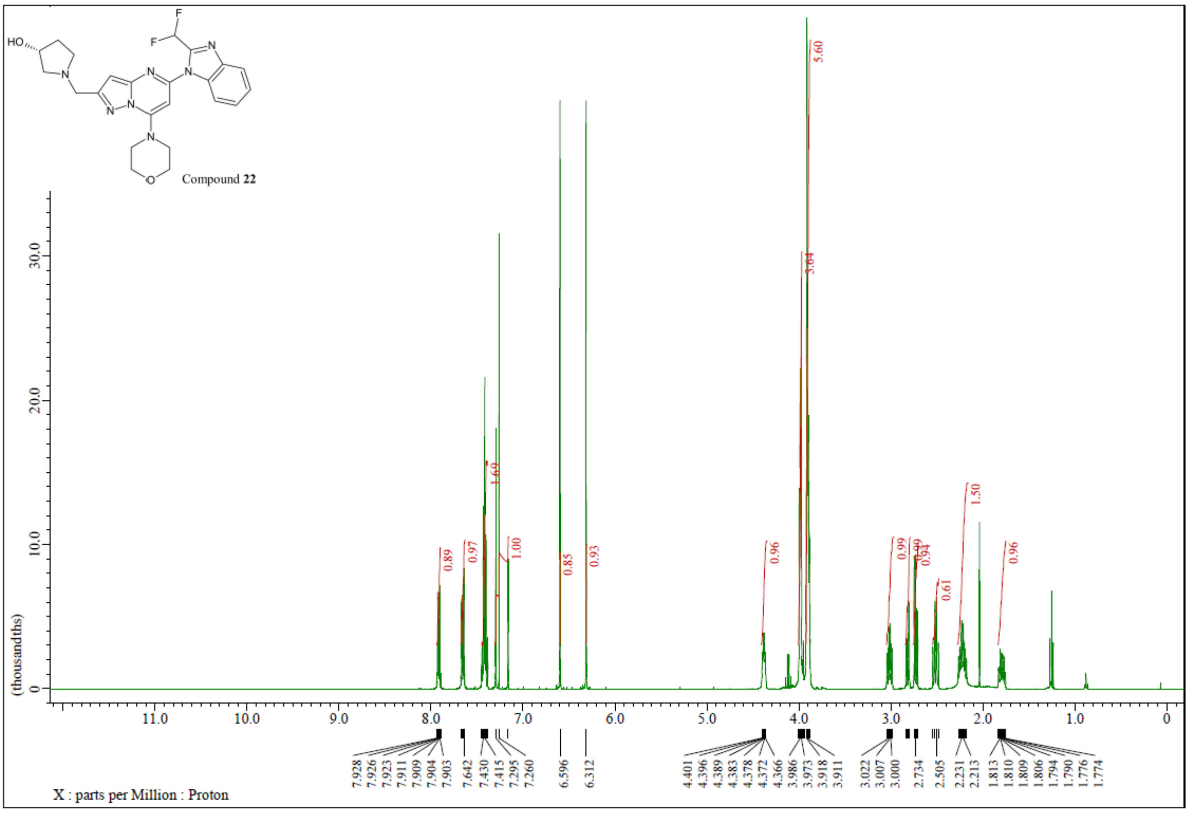
Compound 22
X : parts per Million : Proton
(thousandths)

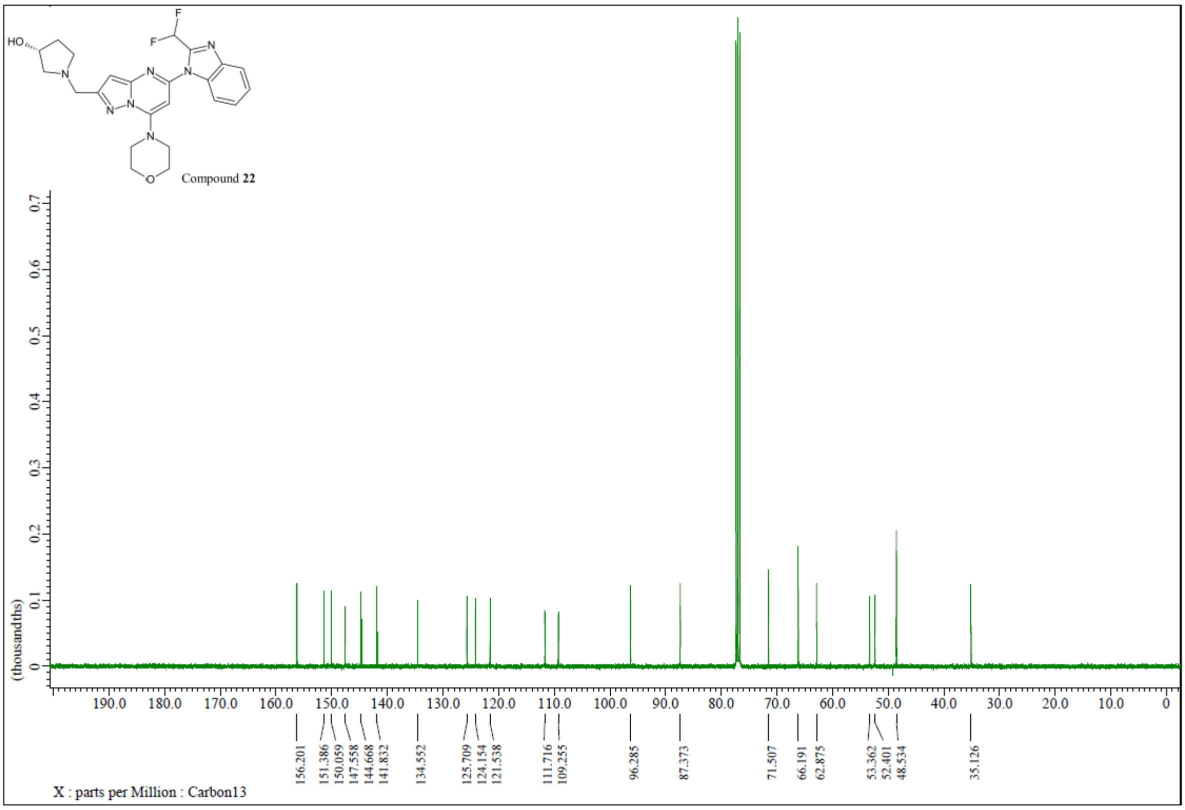
Compound 22
X : parts per Million : Carbon13
(thousandths)
156.201
151.386
150.059
147.558
144.668
141.832
134.552
125.709
124.154
121.538
111.716
109.255
96.285
87.373
71.507
66.191
62.875
53.362
52.401
48.534
35.126

Compound 22

Name	Obs. m/z	Obs. RT	Obs. Mass	Tgt Formula	Tgt Mass	Tgt Mass Error	Find Cpds Algorith
Compound **22**	235.60958	1.19	469.20426	C23 H25 F2 N7 O2	469.20378	1.03	Find by Formula

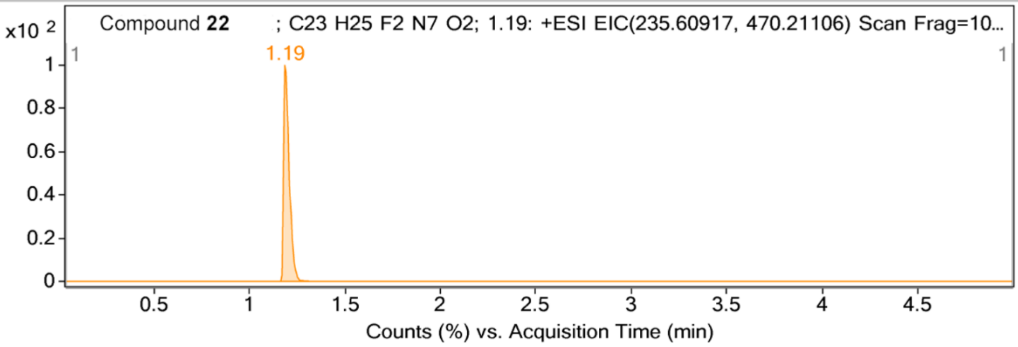

MS Zoomed Spectrum

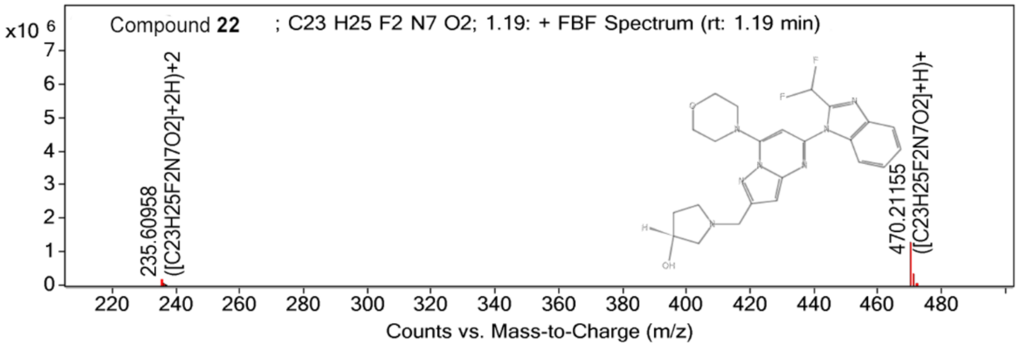

MS Zoomed Spectrum

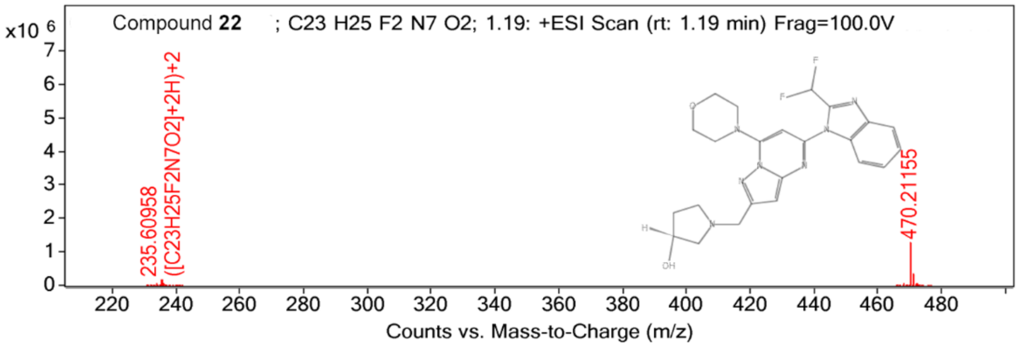

MS Spectrum Peak List

Obs. m/z	Charge	Abund	Formula	Ion/Isotope	Tgt Mass Error (ppm)
235.60958	2	145539.42	C23H25F2N7O2	(M+2H)+2	
236.11083	2	42062.38	C23H25F2N7O2	(M+2H)+2	
236.61221	2	5847.81	C23H25F2N7O2	(M+2H)+2	
237.11271	2	301.78	C23H25F2N7O2	(M+2H)+2	
470.21155	1	1273827.38	C23H25F2N7O2	(M+H)+	
471.21422	1	320760.09	C23H25F2N7O2	(M+H)+	
472.21647	1	44783.03	C23H25F2N7O2	(M+H)+	
235.60958	2	145539.42	C23H25F2N7O2	(M+2H)+2	1.74
236.11083	2	42062.38	C23H25F2N7O2	(M+2H)+2	1.11
236.61221	2	5847.81	C23H25F2N7O2	(M+2H)+2	1.32
237.11271	2	301.78	C23H25F2N7O2	(M+2H)+2	-1.95
470.21155	1	1273827.38	C23H25F2N7O2	(M+H)+	1.06
470.21155		1274626.38			
471.21422	1	320760.09	C23H25F2N7O2	(M+H)+	0.75
472.21647	1	44783.03	C23H25F2N7O2	(M+H)+	-0.09

--- End Of Report ---

Compound 23

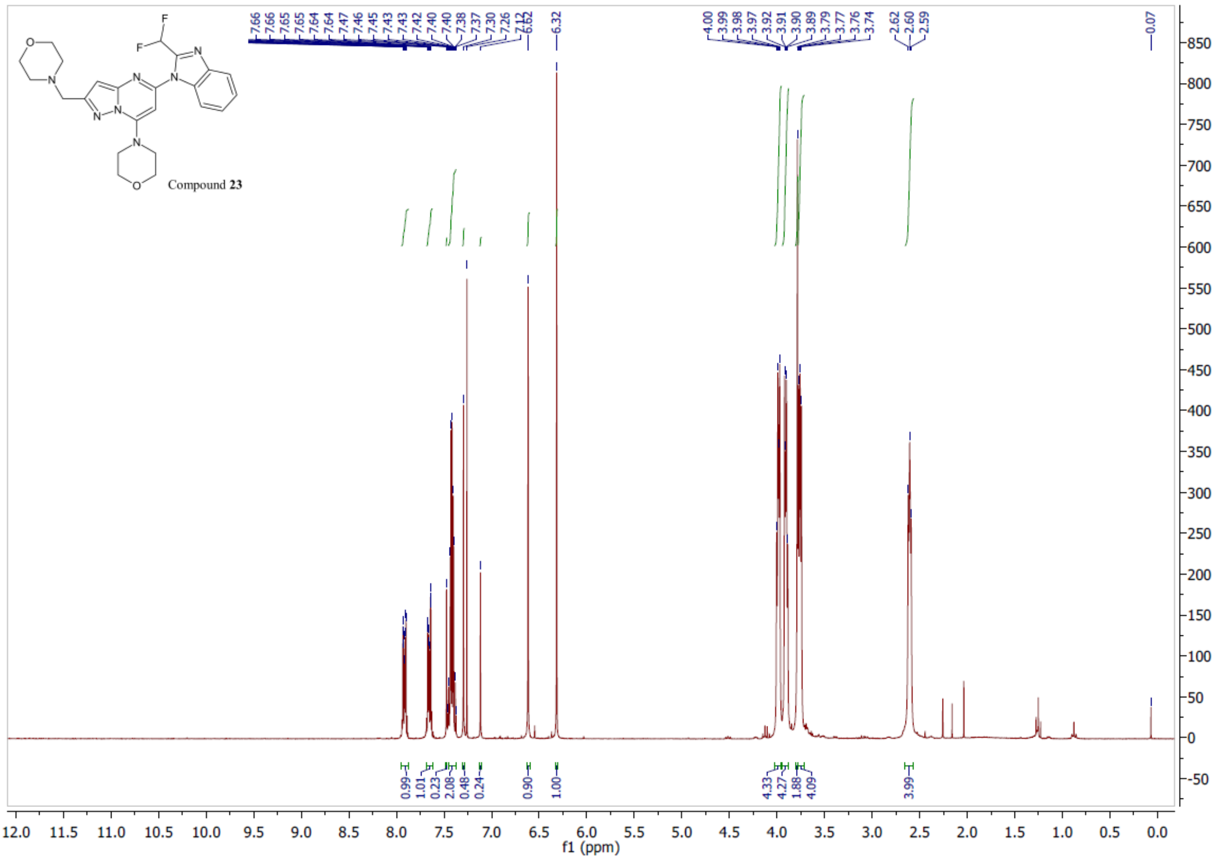

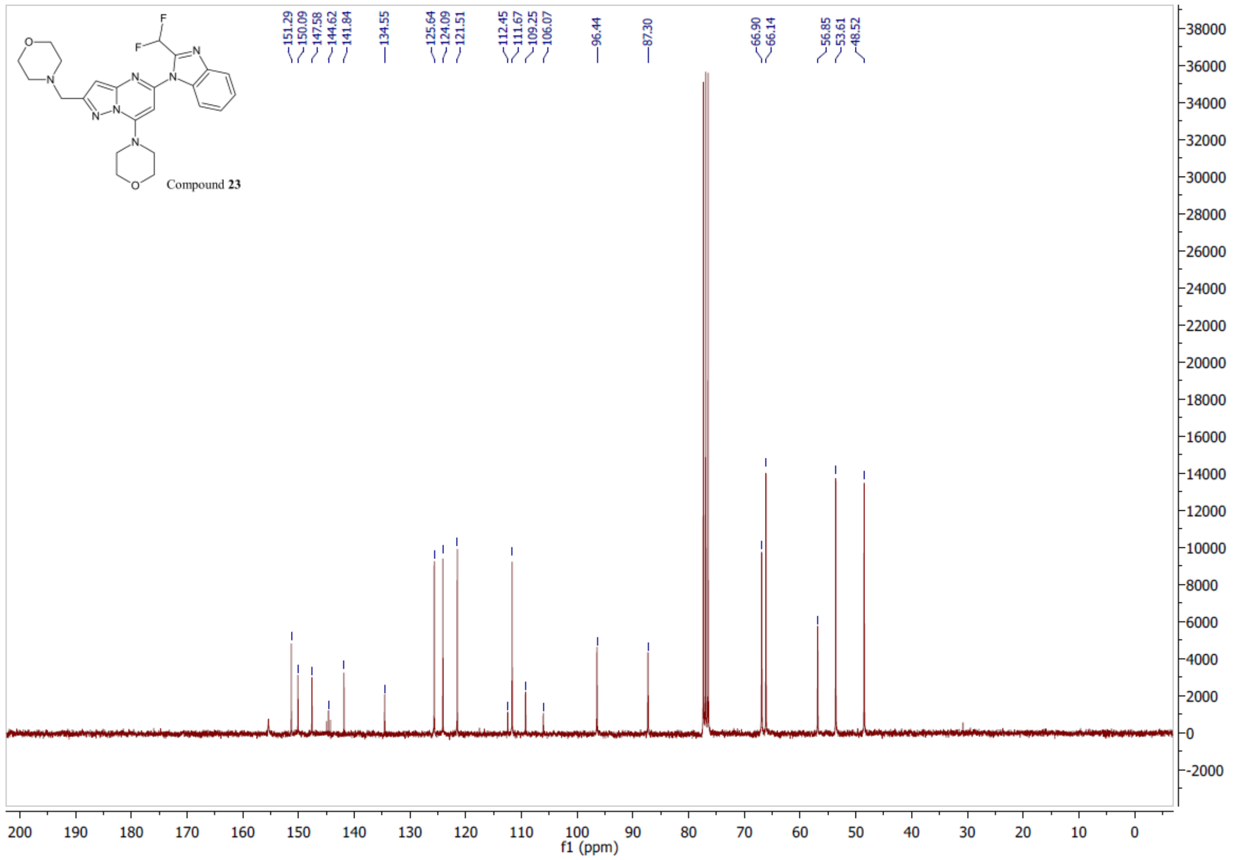

Compound 23

Name	*Obs. m/z*	Obs. RT	Obs. Mass	Tgt Formula	Tgt Mass	Tgt Mass Error	Find Cpds Algorit
Compound **23**	470.21136	1.21	469.20417	C23 H25 F2 N7 O2	469.20378	0.84	Find by Formula

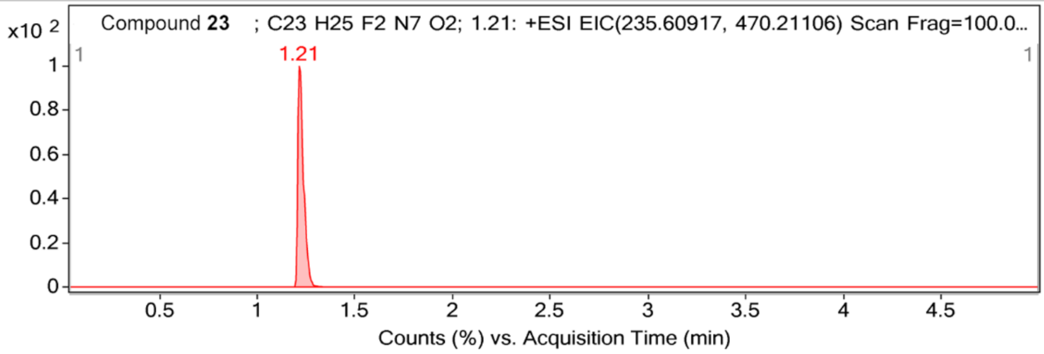

MS Zoomed Spectrum

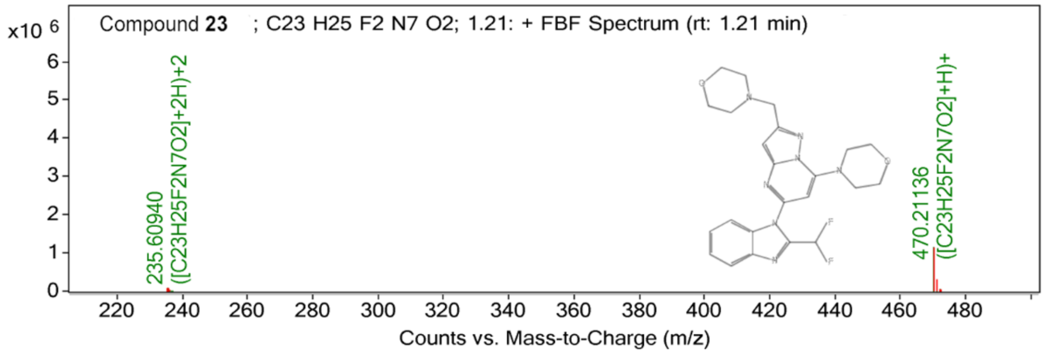

MS Zoomed Spectrum

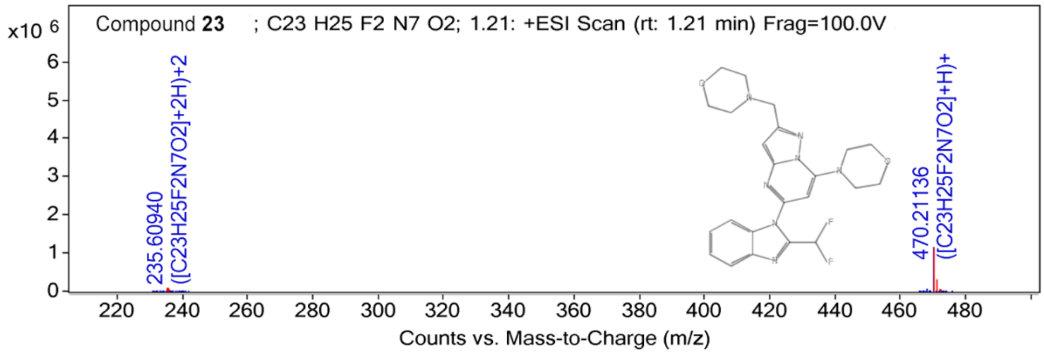

MS Spectrum Peak List

Obs. m/z	Charge	Abund	Formula	Ion/Isotope	Tgt Mass Error (ppm)
235.6094	2	80044.54	C23H25F2N7O2	(M+2H)+2	
236.11123	2	24413.55	C23H25F2N7O2	(M+2H)+2	
236.61164	2	3186	C23H25F2N7O2	(M+2H)+2	
237.11175	2	553.57	C23H25F2N7O2	(M+2H)+2	
470.21136	1	1140152.63	C23H25F2N7O2	(M+H)+	
471.21459	1	286698.06	C23H25F2N7O2	(M+H)+	
472.21642	1	41395.54	C23H25F2N7O2	(M+H)+	
235.6094	2	80044.54	C23H25F2N7O2	(M+2H)+2	0.99
236.11123	2	24413.55	C23H25F2N7O2	(M+2H)+2	2.78
236.61164	2	3186	C23H25F2N7O2	(M+2H)+2	-1.08
237.11175	2	553.57	C23H25F2N7O2	(M+2H)+2	-5.98
470.21136	1	1140152.63	C23H25F2N7O2	(M+H)+	0.65
471.21459	1	286698.06	C23H25F2N7O2	(M+H)+	1.53
472.21642	1	41395.54	C23H25F2N7O2	(M+H)+	-0.2

--- End Of Report ---

Compound 24

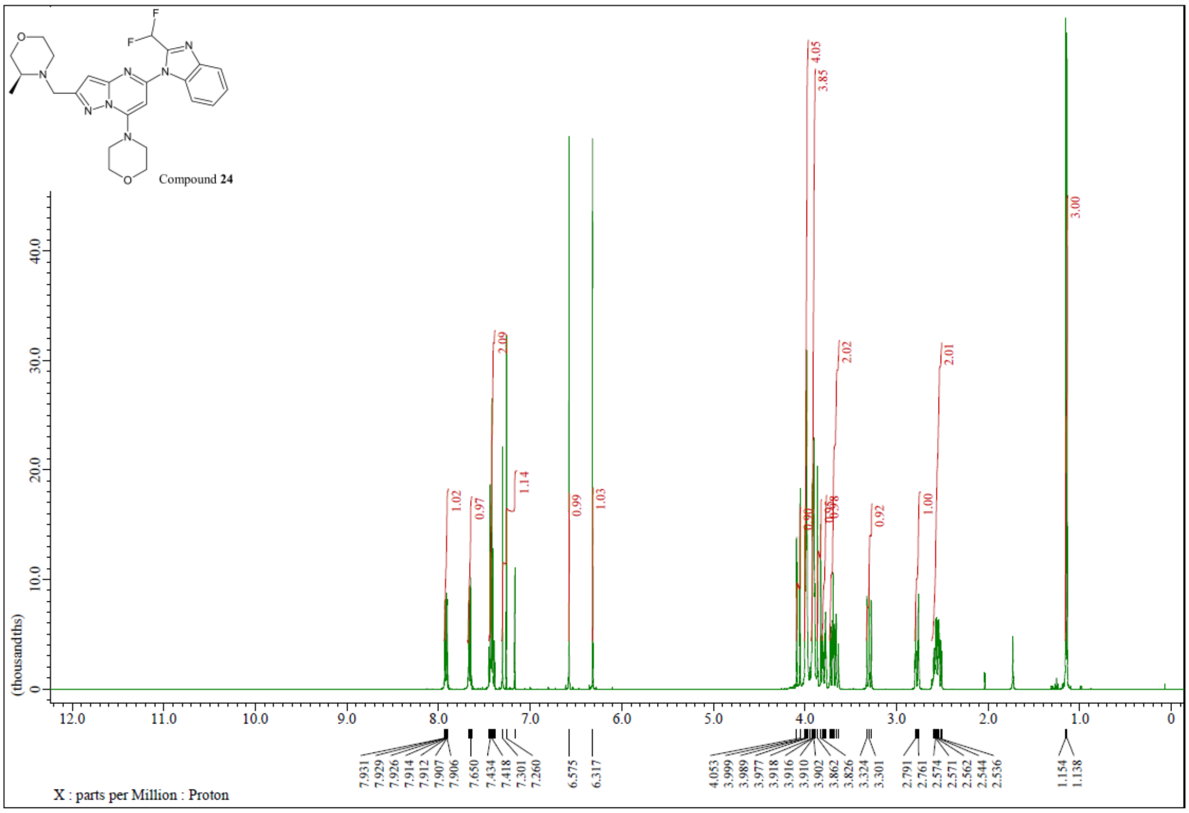

Compound 24
(thousandths)
X : parts per Million : Proton
7.931
7.929
7.926
7.914
7.912
7.907
7.906
7.650
7.434
7.418
7.301
7.260
6.575
6.317
4.053
3.999
3.989
3.977
3.918
3.916
3.910
3.902
3.862
3.826
3.324
3.301
2.791
2.761
2.574
2.571
2.562
2.544
2.536
1.154
1.138

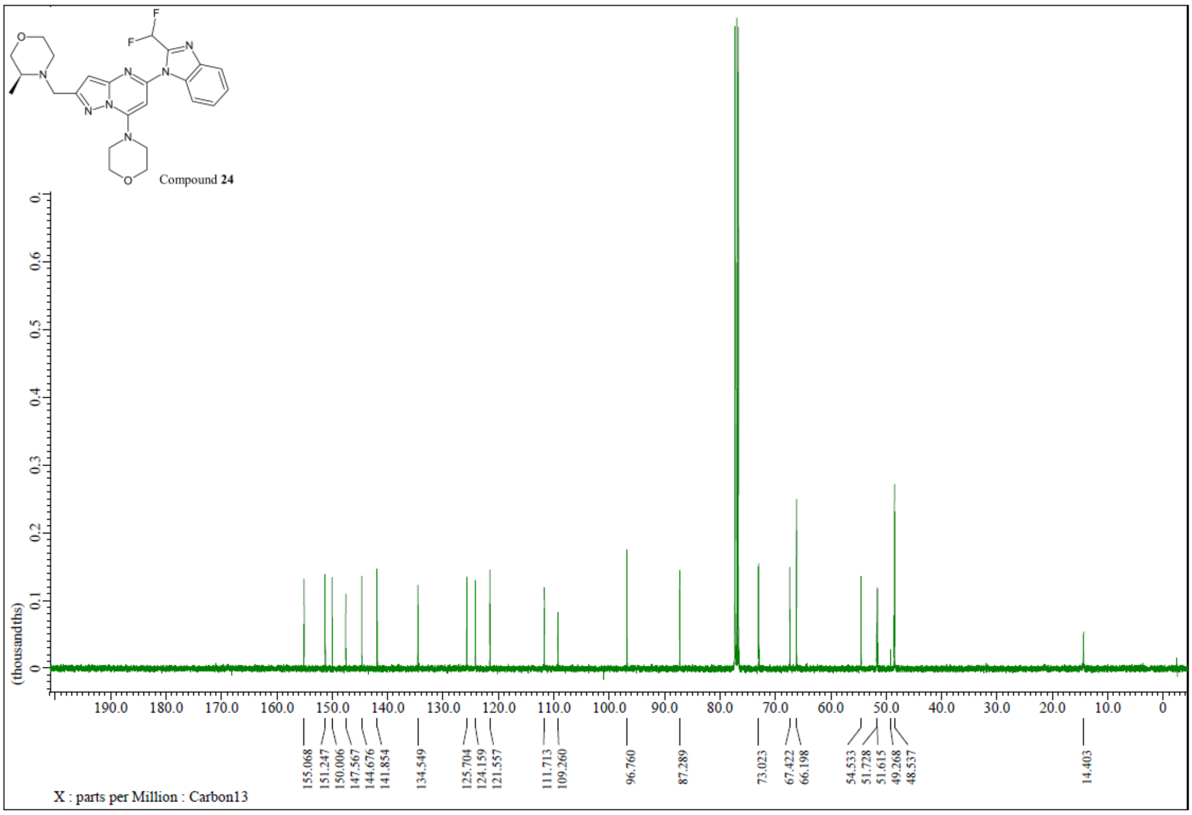

Compound 24
(thousandths)
X : parts per Million : Carbon13
155.068
151.247
150.006
147.567
144.676
141.854
134.549
125.704
124.159
121.557
111.713
109.260
96.760
87.289
73.023
67.422
66.198
54.533
51.728
51.615
49.268
48.537
14.403

Compound 24

Name	*Obs. m/z*	Obs. RT	Obs. Mass	Tgt Formula	Tgt Mass	Tgt Mass Error	Find Cpds Algorith
Compound **24**	484.2269	1.18	483.2196	C24 H27 F2 N7 O2	483.2194	0.28	Find by Formula

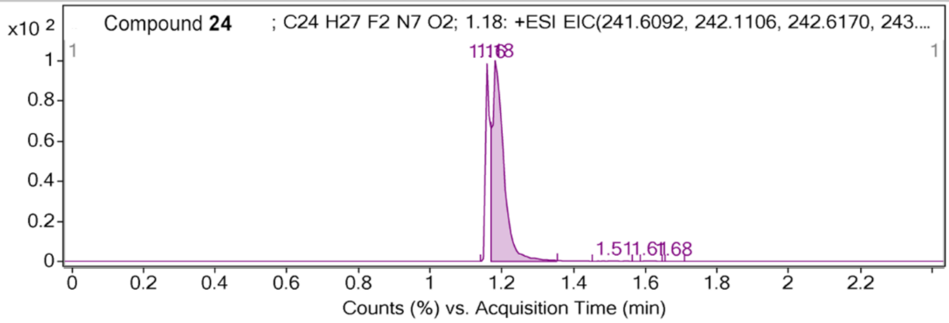

MS Zoomed Spectrum

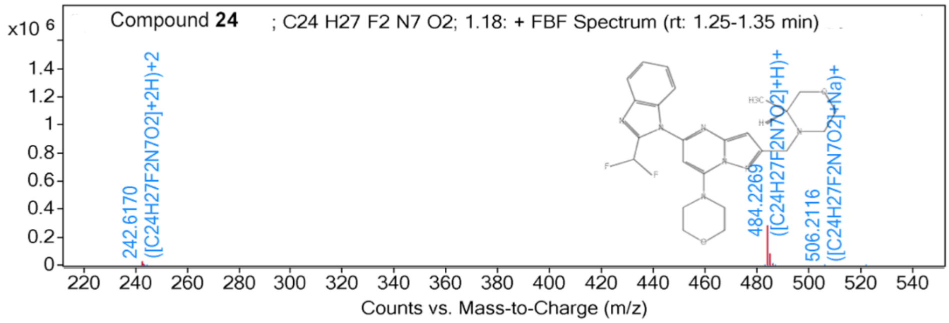

MS Zoomed Spectrum

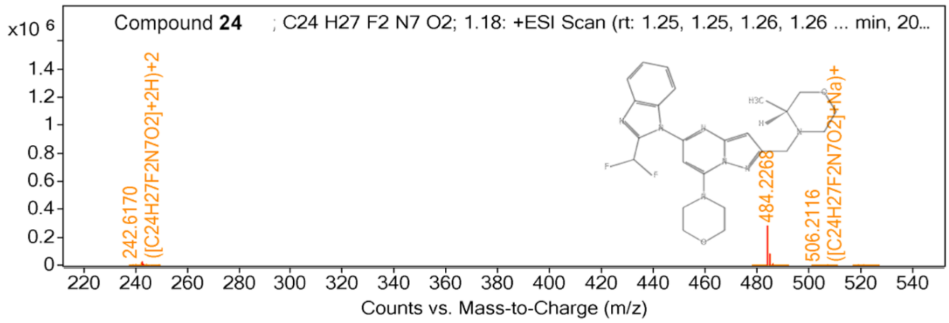

MS Spectrum Peak List

Obs. m/z	Charge	Abund	Formula	Ion/Isotope	Tgt Mass Error (ppm)
242.617	2	23954.76	C24H27F2N7O2	(M+2H)+2	
243.1188	2	7445.91	C24H27F2N7O2	(M+2H)+2	
243.6196	2	1176.52	C24H27F2N7O2	(M+2H)+2	
483.219	1	463.12	C24H27F2N7O2	M+	
484.2269	1	282509	C24H27F2N7O2	(M+H)+	
485.2295	1	79724.67	C24H27F2N7O2	(M+H)+	
486.2323	1	12347.87	C24H27F2N7O2	(M+H)+	
487.2351	1	1594.3	C24H27F2N7O2	(M+H)+	
506.2116	1	253.18	C24H27F2N7O2	(M+Na)+	
522.1799	1	175.05	C24H27F2N7O2	(M+K)+	
242.617	2	23954.76	C24H27F2N7O2	(M+2H)+2	0.23
243.1188	2	7445.91	C24H27F2N7O2	(M+2H)+2	1.66
243.6196	2	1176.52	C24H27F2N7O2	(M+2H)+2	-0.52
483.219	1	463.12	C24H27F2N7O2	M+	0.26
484.2269	1	282509	C24H27F2N7O2	(M+H)+	0.33
485.2295	1	79724.67	C24H27F2N7O2	(M+H)+	0.02
486.2323	1	12347.87	C24H27F2N7O2	(M+H)+	0.15
487.2351	1	1594.3	C24H27F2N7O2	(M+H)+	0.7
506.2116	1	253.18	C24H27F2N7O2	(M+Na)+	5.76
522.1799	1	175.05	C24H27F2N7O2	(M+K)+	-5.13

--- End Of Report ---

Compound 25

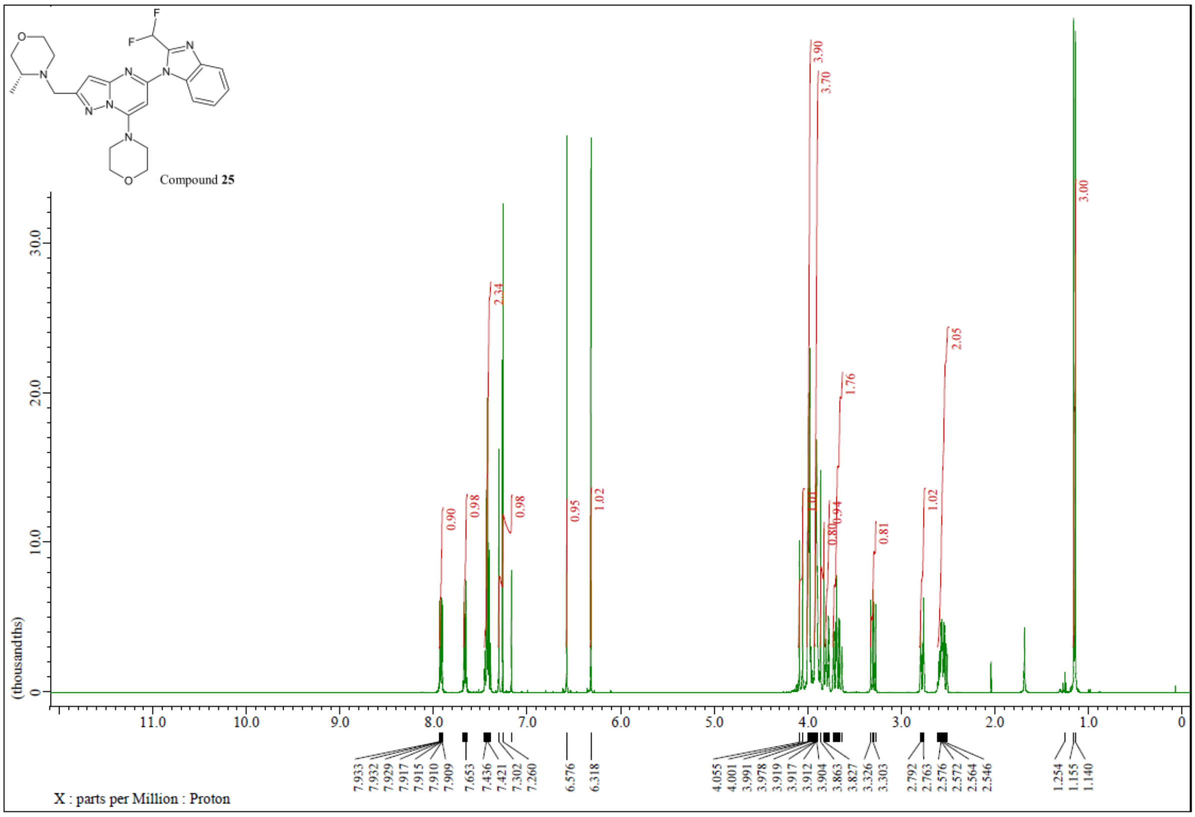

Compound 25
(thousandths)
X : parts per Million : Proton

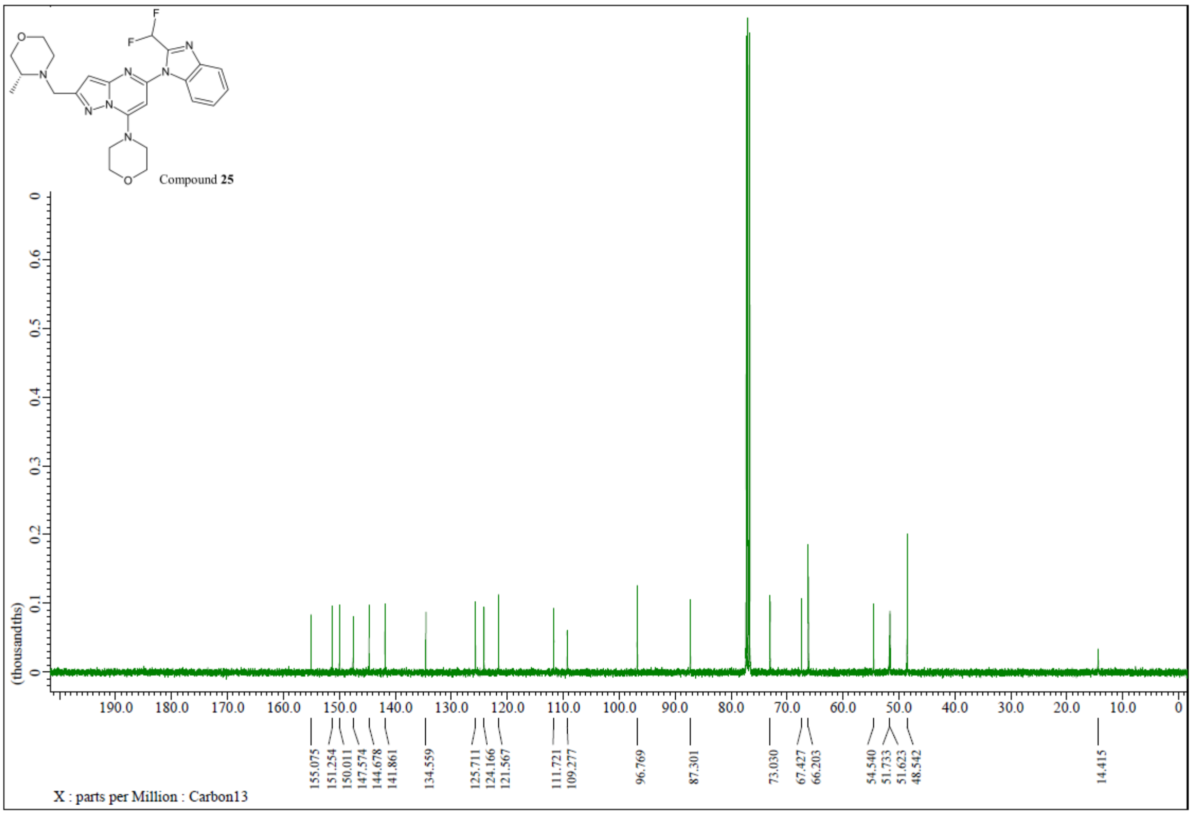

Compound 25
(thousandths)
X : parts per Million : Carbon13

Compound 25

Name	*Obs. m/z*	Obs. RT	Obs. Mass	Tgt Formula	Tgt Mass	Tgt Mass Error	Find Cpds Algorith
Compound **25**	484.2268	1.18	483.2195	C24 H27 F2 N7 O2	483.2194	0.06	Find by Formula

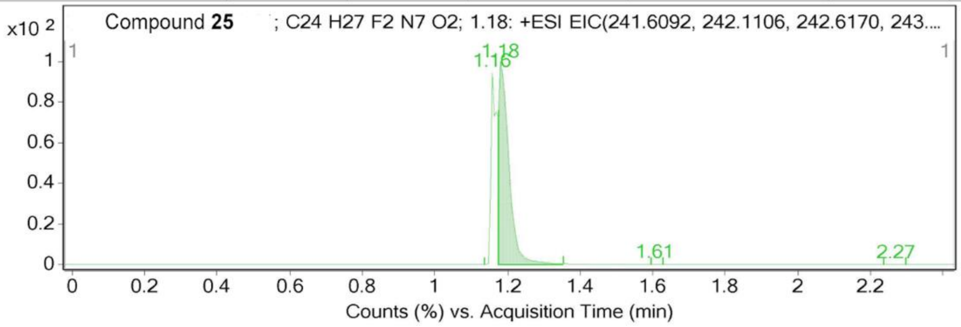

MS Zoomed Spectrum

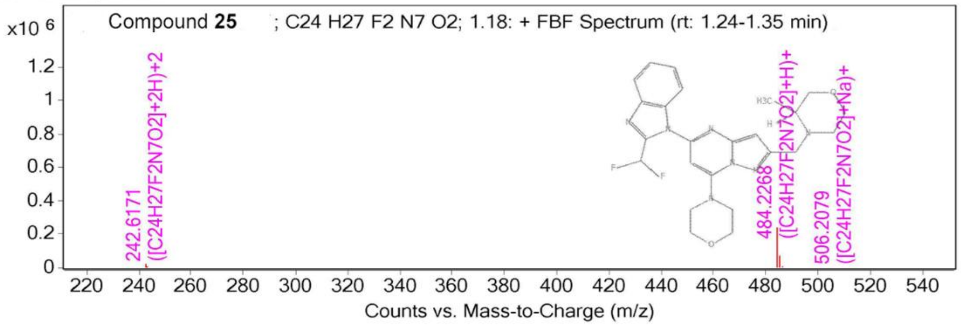

MS Zoomed Spectrum

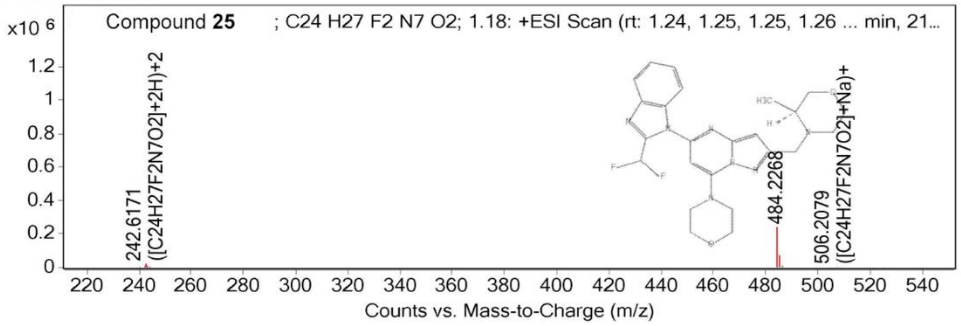

MS Spectrum Peak List

Obs. m/z	Charge	Abund	Formula	Ion/Isotope	Tgt Mass Error (ppm)
241.6083	2	349.53	C24H27F2N7O2	M+2	
242.6171	2	19071.85	C24H27F2N7O2	(M+2H)+2	
243.119	2	6438.57	C24H27F2N7O2	(M+2H)+2	
243.619	2	918.06	C24H27F2N7O2	(M+2H)+2	
484.2268	1	239466.45	C24H27F2N7O2	(M+H)+	
485.2293	1	67776.46	C24H27F2N7O2	(M+H)+	
486.2318	1	9900.11	C24H27F2N7O2	(M+H)+	
487.2347	1	1392.6	C24H27F2N7O2	(M+H)+	
506.2079	1	302.17	C24H27F2N7O2	(M+Na)+	
522.1879	1	143.76	C24H27F2N7O2	(M+K)+	
241.6083	2	349.53	C24H27F2N7O2	M+2	-3.76
242.6171	2	19071.85	C24H27F2N7O2	(M+2H)+2	0.44
243.119	2	6438.57	C24H27F2N7O2	(M+2H)+2	2.36
243.619	2	918.06	C24H27F2N7O2	(M+2H)+2	-2.94
484.2268	1	239466.45	C24H27F2N7O2	(M+H)+	0.15
485.2293	1	67776.46	C24H27F2N7O2	(M+H)+	-0.43
486.2318	1	9900.11	C24H27F2N7O2	(M+H)+	-0.78
487.2347	1	1392.6	C24H27F2N7O2	(M+H)+	-0.16
506.2079	1	302.17	C24H27F2N7O2	(M+Na)+	-1.49
522.1879	1	143.76	C24H27F2N7O2	(M+K)+	10.09

--- End Of Report ---

Compound 26

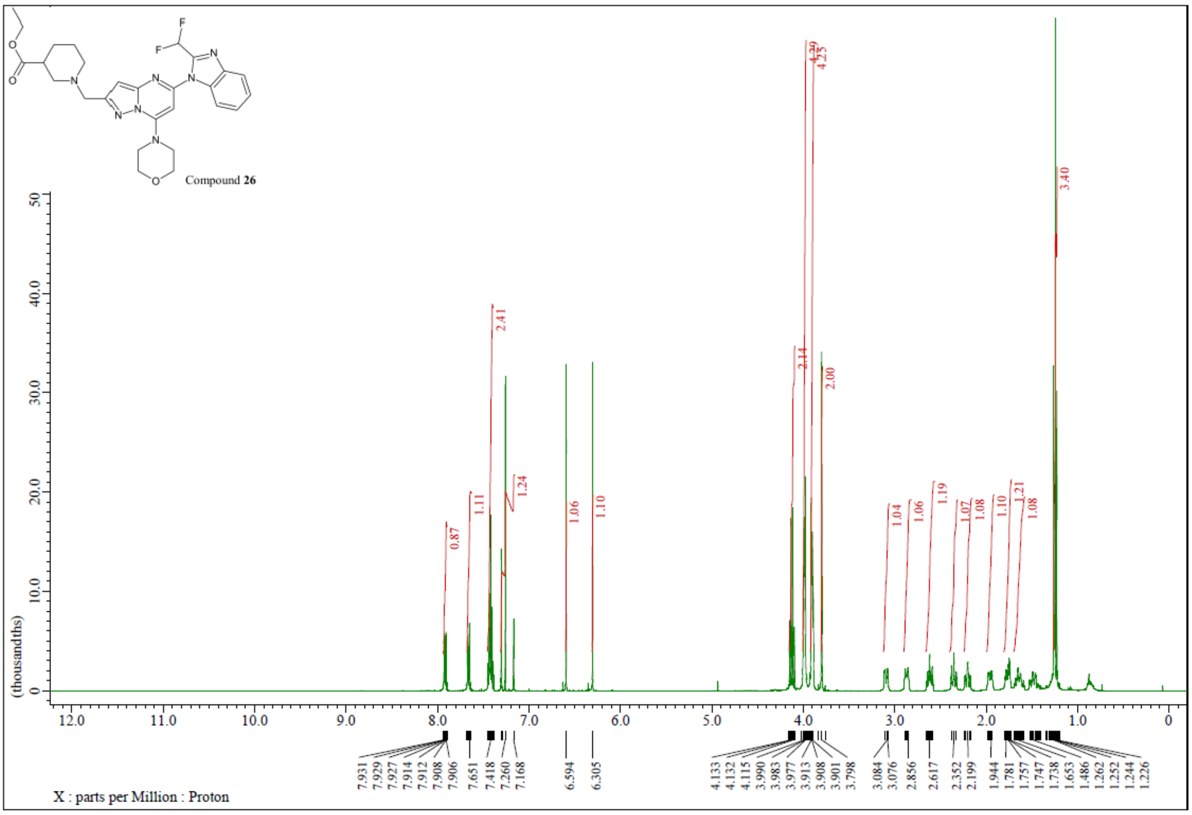

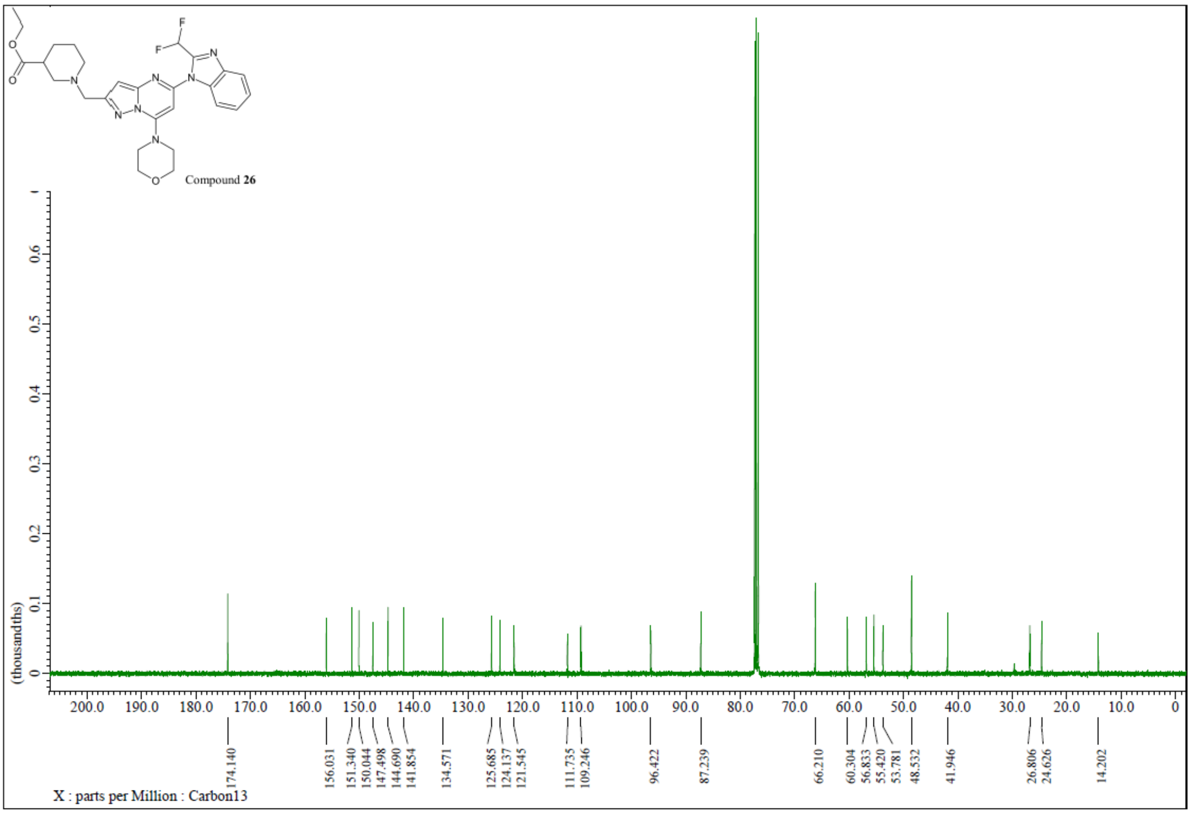

Compound 26

Name	*Obs. m/z*	Obs. RT	Obs. Mass	Tgt Formula	Tgt Mass	Tgt Mass Error	Find Cpds Algorit
Compound **26**	270.63066	1.45	539.24629	C27 H31 F2 N7 O3	539.24564	1.19	Find by Formula

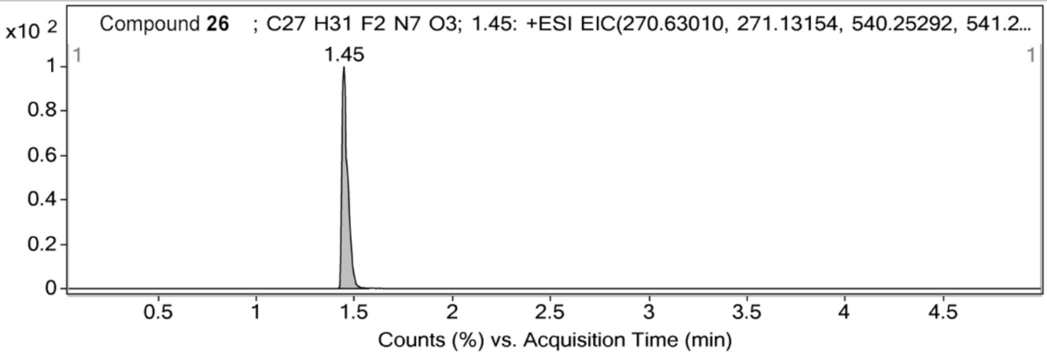

MS Zoomed Spectrum

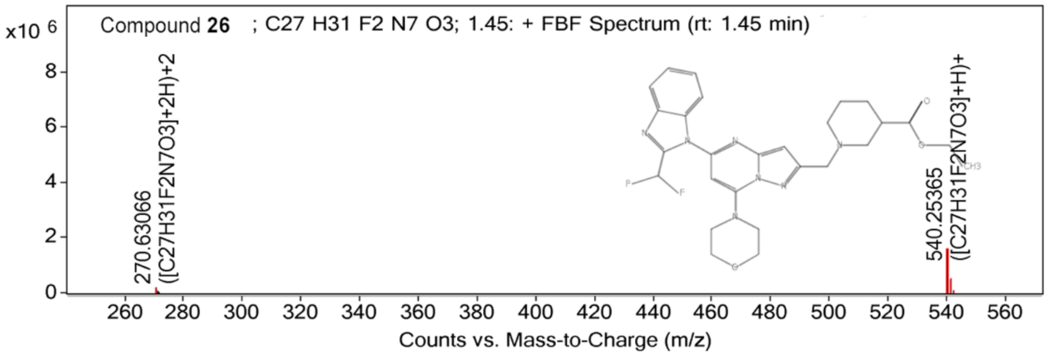

MS Zoomed Spectrum

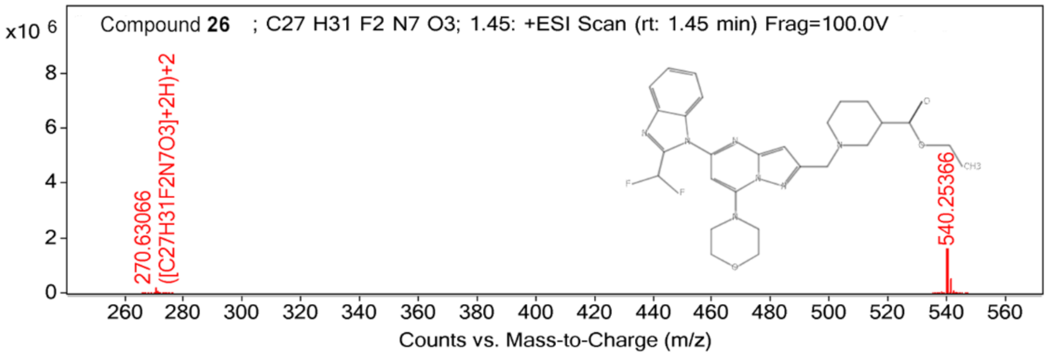

MS Spectrum Peak List

Obs. m/z	Charge	Abund	Formula	Ion/Isotope	Tgt Mass Error (ppm)
270.63066	2	179367.64	C27H31F2N7O3	(M+2H)+2	
271.13199	2	57800.48	C27H31F2N7O3	(M+2H)+2	
271.6328	2	10418.95	C27H31F2N7O3	(M+2H)+2	
540.25365	1	1591887.75	C27H31F2N7O3	(M+H)+	
541.25604	1	472964.91	C27H31F2N7O3	(M+H)+	
542.25858	1	77634.71	C27H31F2N7O3	(M+H)+	
270.63066	2	179367.64	C27H31F2N7O3	(M+2H)+2	2.06
271.13199	2	57800.48	C27H31F2N7O3	(M+2H)+2	1.64
271.6328	2	10418.95	C27H31F2N7O3	(M+2H)+2	-0.35
540.25365	1	1591887.75	C27H31F2N7O3	(M+H)+	1.36
540.25366		1593459.38			
541.25604	1	472964.91	C27H31F2N7O3	(M+H)+	0.43
542.25858	1	77634.71	C27H31F2N7O3	(M+H)+	0.12

--- End Of Report ---

Compound 27

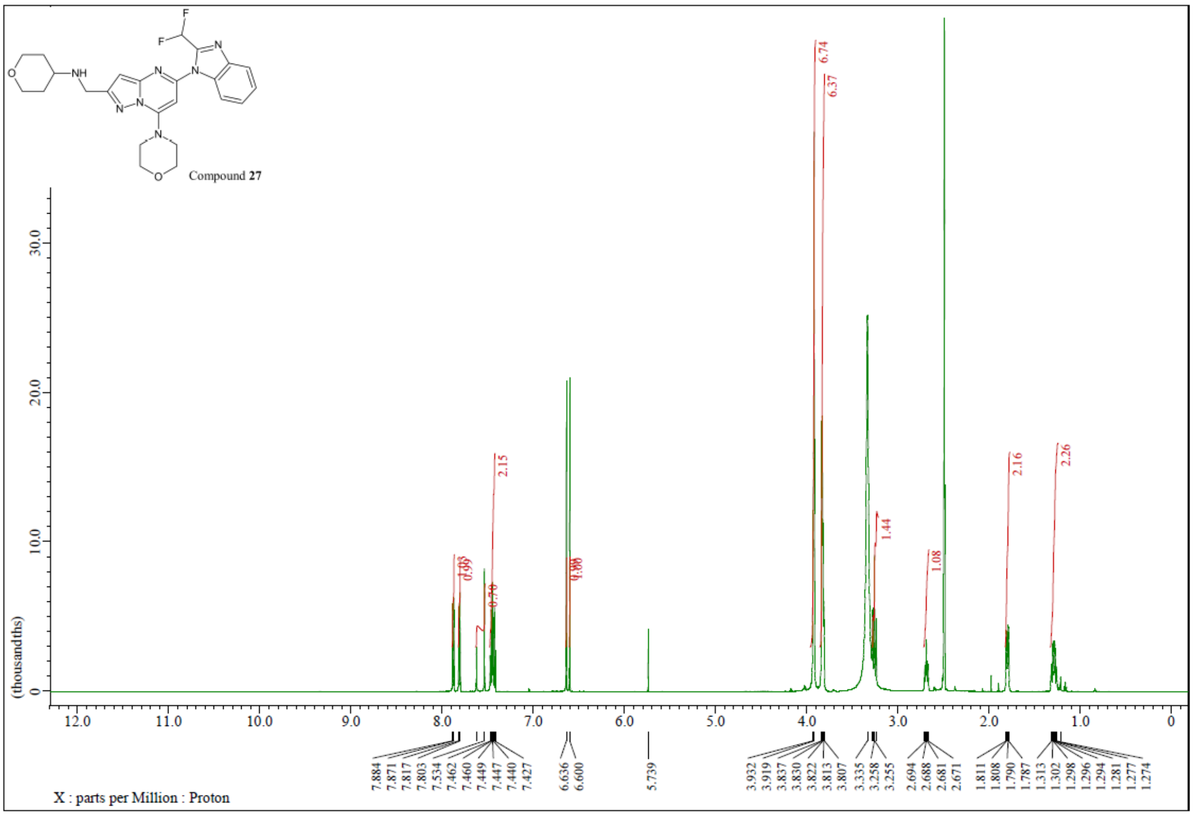
Compound 27
(thousandths)
X : parts per Million : Proton
7.884
7.871
7.817
7.803
7.534
7.462
7.460
7.449
7.447
7.440
7.427
6.636
6.600
5.739
3.932
3.919
3.837
3.830
3.822
3.813
3.807
3.335
3.258
3.255
2.694
2.688
2.681
2.671
1.811
1.808
1.790
1.787
1.313
1.302
1.298
1.296
1.294
1.281
1.277
1.274
6.74
6.37
2.15
1.44
1.08
2.16
2.26

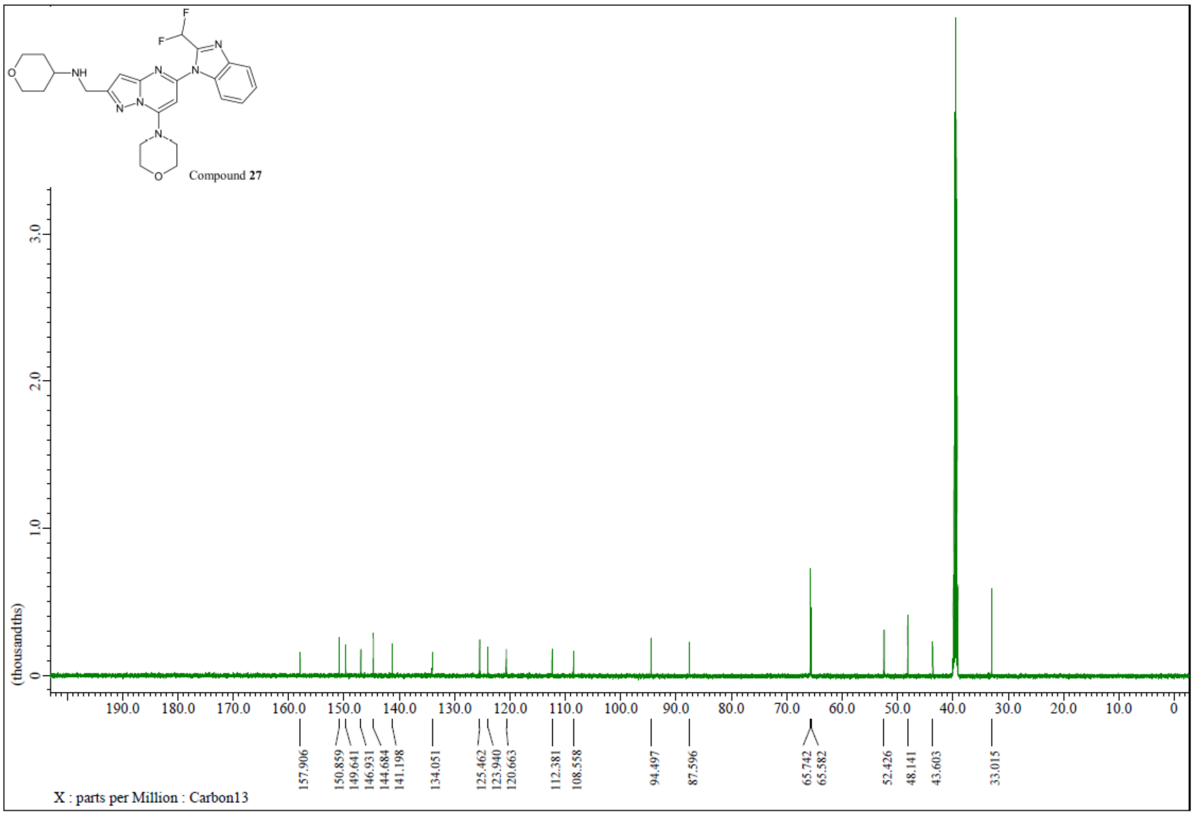
Compound 27
(thousandths)
X : parts per Million : Carbon13
157.906
150.859
149.641
146.931
144.684
141.198
134.051
125.462
123.940
120.663
112.381
108.558
94.497
87.596
65.742
65.582
52.426
48.141
43.603
33.015

Compound 27

Name	*Obs. m/z*	Obs. RT	Obs. Mass	Tgt Formula	Tgt Mass	Tgt Mass Error	Find Cpds Algorith
Compound **27**	484.2266	1.16	483.2193	C24 H27 F2 N7 O2	483.2194	-0.29	Find by Formula

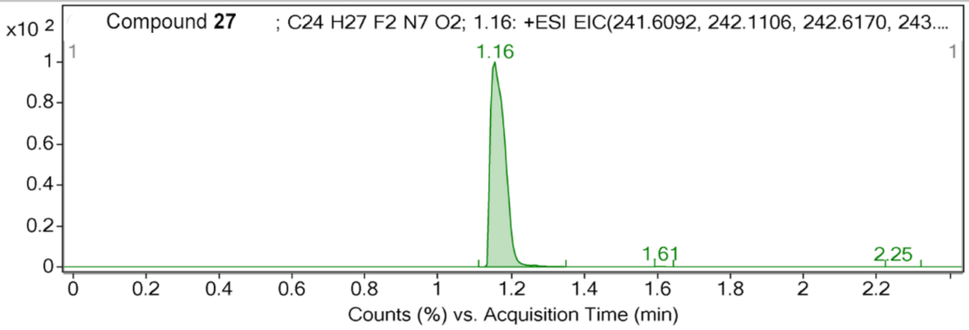

MS Zoomed Spectrum

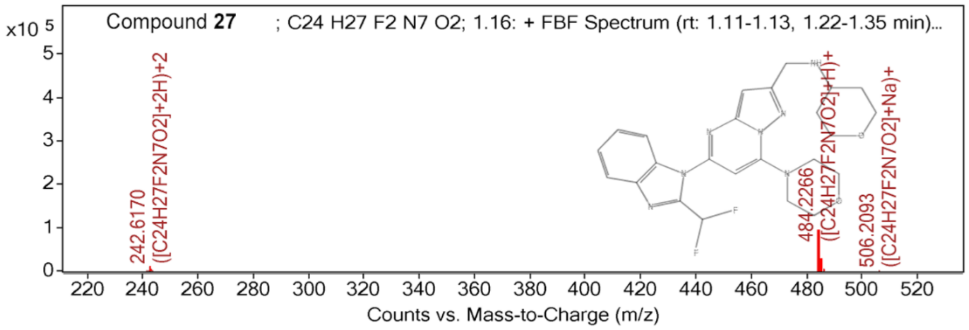

MS Zoomed Spectrum

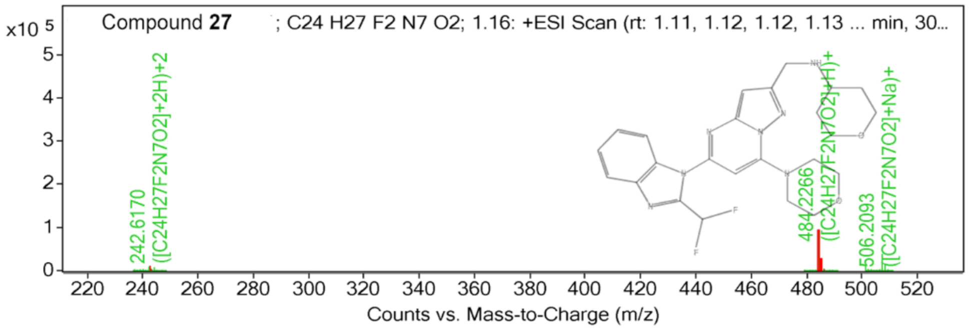

MS Spectrum Peak List

Obs. m/z	Charge	Abund	Formula	Ion/Isotope	Tgt Mass Error (ppm)
241.6093	2	641.2	C24H27F2N7O2	M+2	
242.1097	2	215.29	C24H27F2N7O2	M+2	
242.617	2	9418.48	C24H27F2N7O2	(M+2H)+2	
243.1191	2	3902.27	C24H27F2N7O2	(M+2H)+2	
243.6188	2	480.77	C24H27F2N7O2	(M+2H)+2	
484.2266	1	93398.47	C24H27F2N7O2	(M+H)+	
485.2292	1	26572.49	C24H27F2N7O2	(M+H)+	
486.2313	1	4498.85	C24H27F2N7O2	(M+H)+	
506.2093	1	299.24	C24H27F2N7O2	(M+Na)+	
241.6093	2	641.2	C24H27F2N7O2	M+2	0.56
242.1097	2	215.29	C24H27F2N7O2	M+2	-3.69
242.617	2	9418.48	C24H27F2N7O2	(M+2H)+2	0.19
243.1191	2	3902.27	C24H27F2N7O2	(M+2H)+2	2.77
243.6188	2	480.77	C24H27F2N7O2	(M+2H)+2	-3.99
484.2266	1	93398.47	C24H27F2N7O2	(M+H)+	-0.26
485.2292	1	26572.49	C24H27F2N7O2	(M+H)+	-0.67
486.2313	1	4498.85	C24H27F2N7O2	(M+H)+	-1.85
506.2093	1	299.24	C24H27F2N7O2	(M+Na)+	1.21

--- End Of Report ---

Compound 28

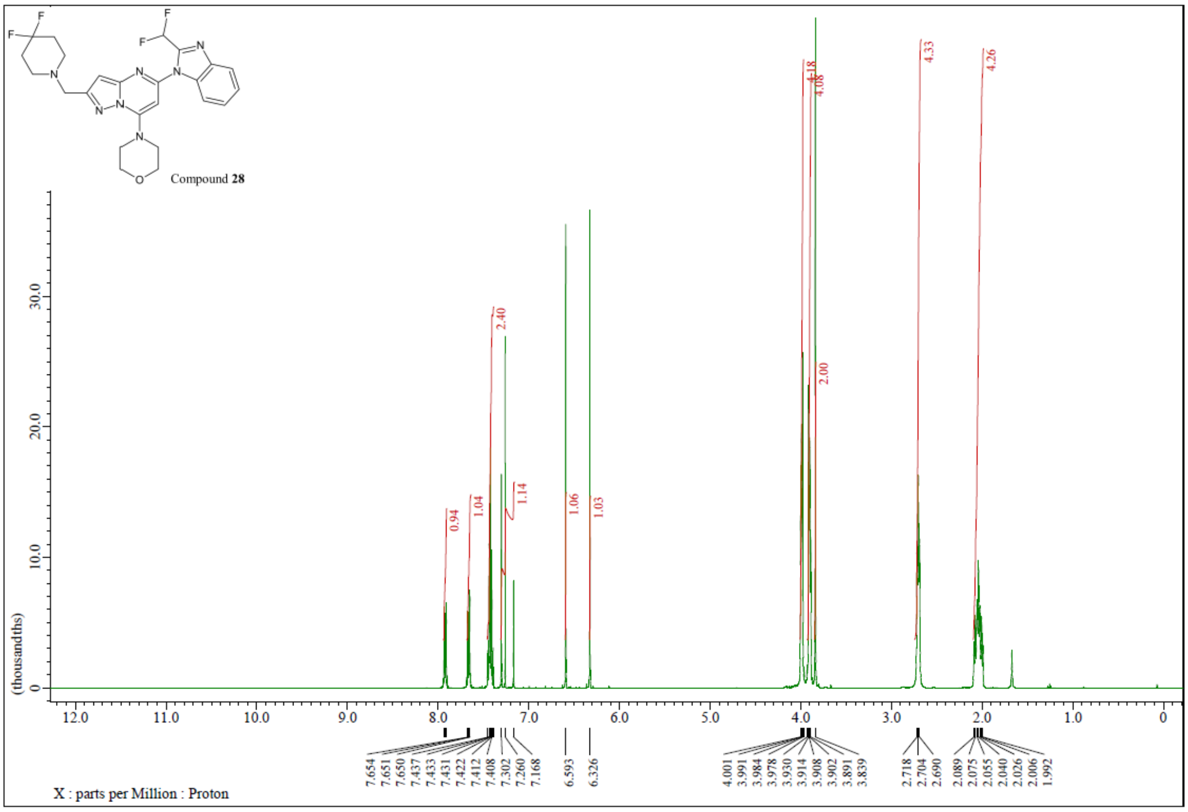

Compound 28
(thousandths)
X : parts per Million : Proton

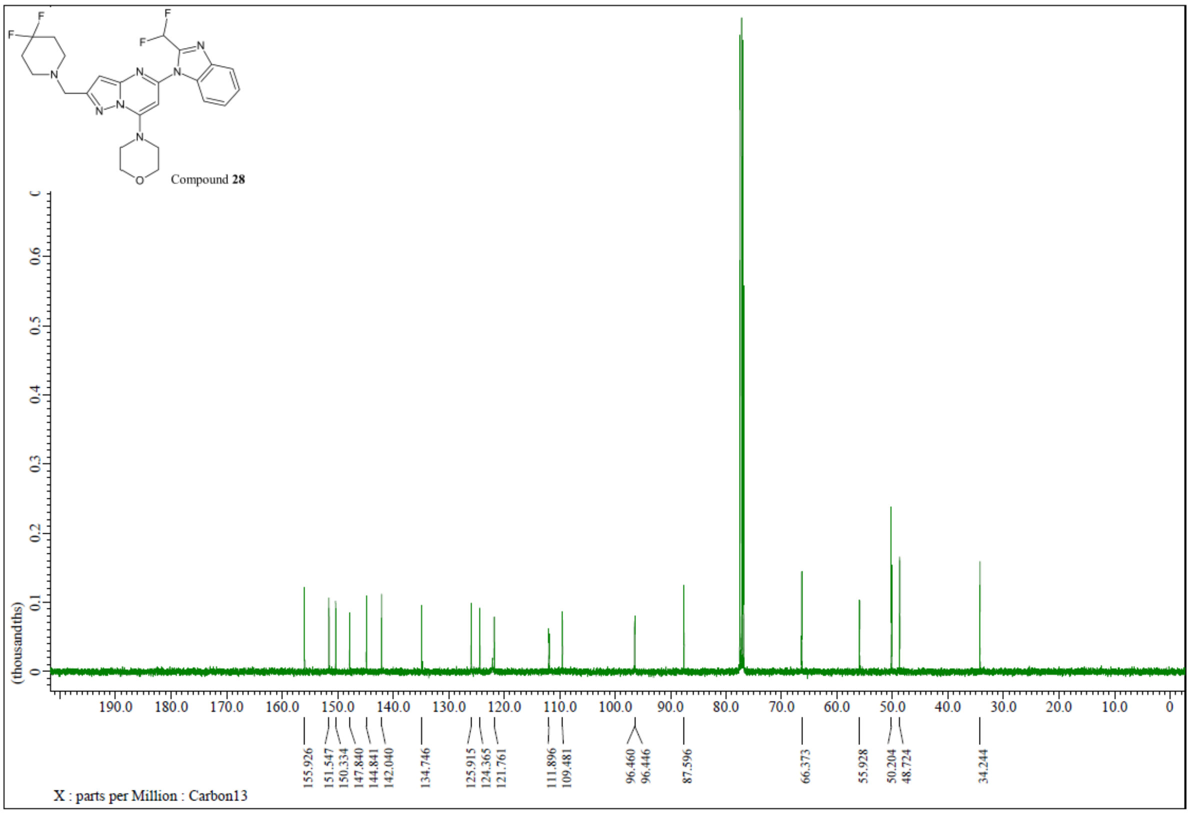

Compound 28
(thousandths)
X : parts per Million : Carbon13

Compound 28

Compound Label	Name	Algorithm	Mass
Compound **28**	Compound **28**	Spectrum Extraction	503.21022

MS Spectrum

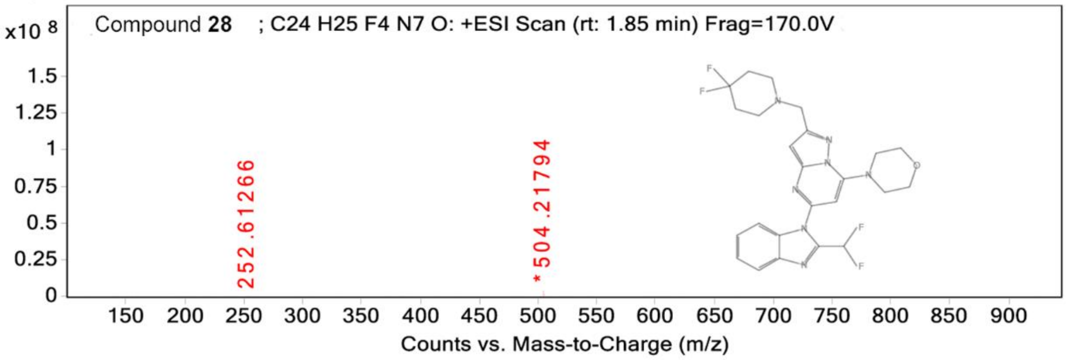

MS Zoomed Spectrum

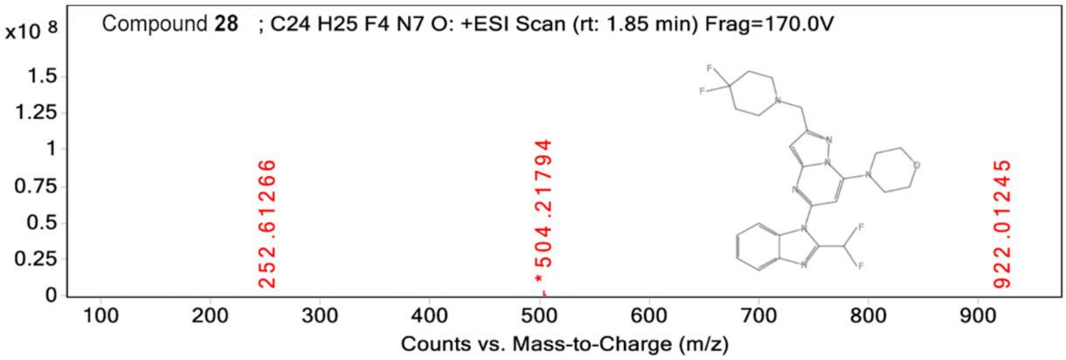

MS Spectrum Peak List

m/z	*Calc m/z*	Diff(ppm)	z	Abund	Formula	Ion
121.05149	98.06004	234462.98	1	107715.05	C5 H8 N O	(M+Na)+
134.0781	116.04321	155415.3	1	30413.49	C6 H6 F2	(M+NH4)+
242.60846	224.57262	80311.83	2	38307	C24 H18 F3 N5 O	(M+2(NH4))+2
252.61266			2	275026.94		
253.11332			2	88366.99		
502.19922	484.16292	37252.54	1	59938.18	C24 H20 F4 N6 O	(M+NH4)+
504.21794	504.21295	9.9	1	3615947.75		
505.21907	505.21577	6.53	1	1067767.5		
506.22162	506.21851	6.15	1	148034.56		
922.01245			1	133568.02		

Compound 29

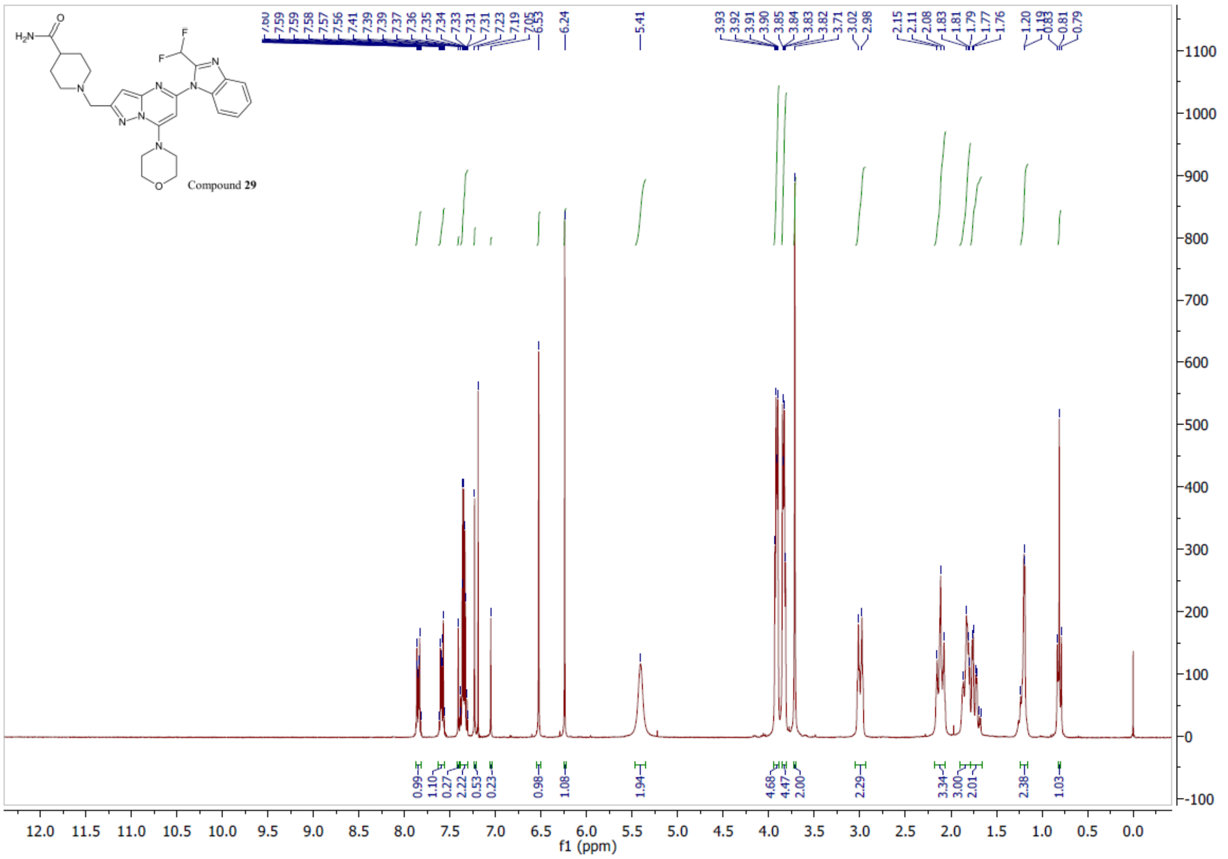

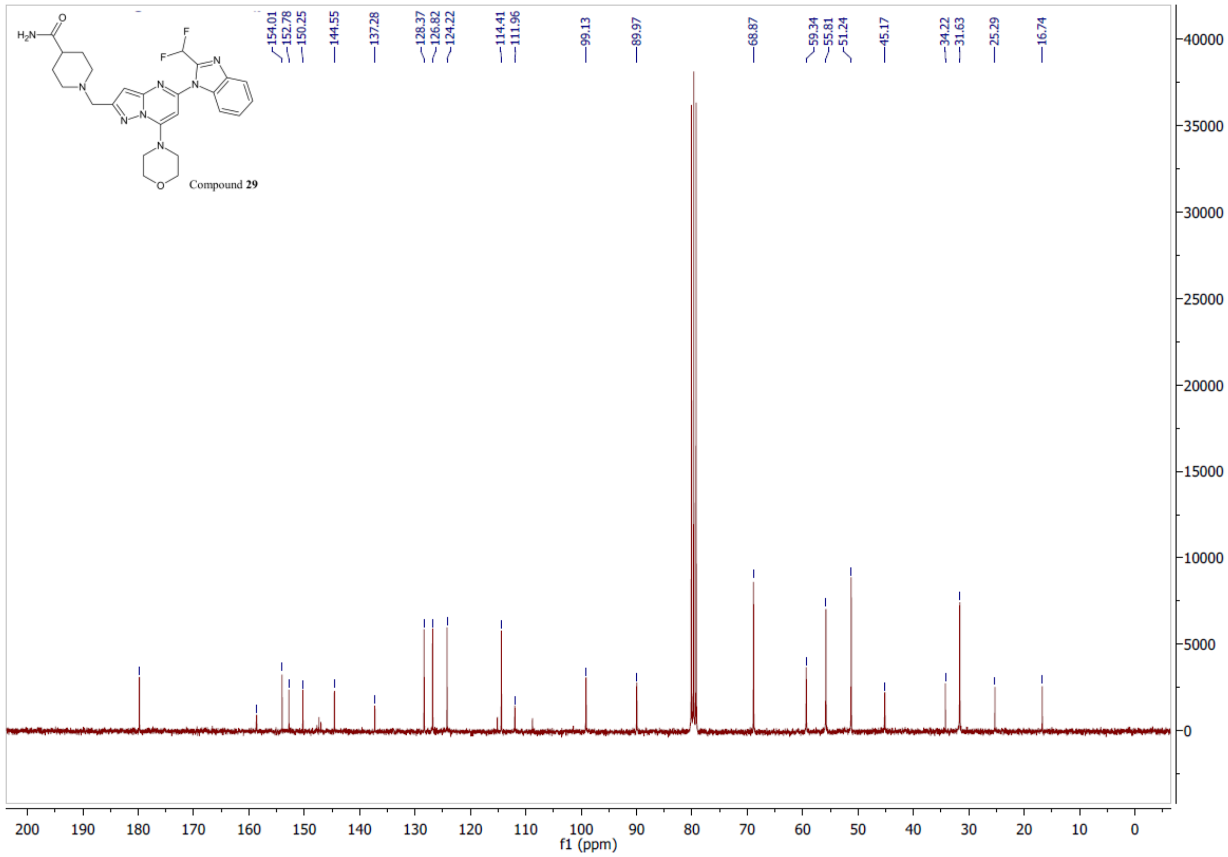

Compound 29

Compound Label	Name	Algorithm	Mass
Compound **29**	Compound **29**	Spectrum Extraction	510.2333

MS Spectrum

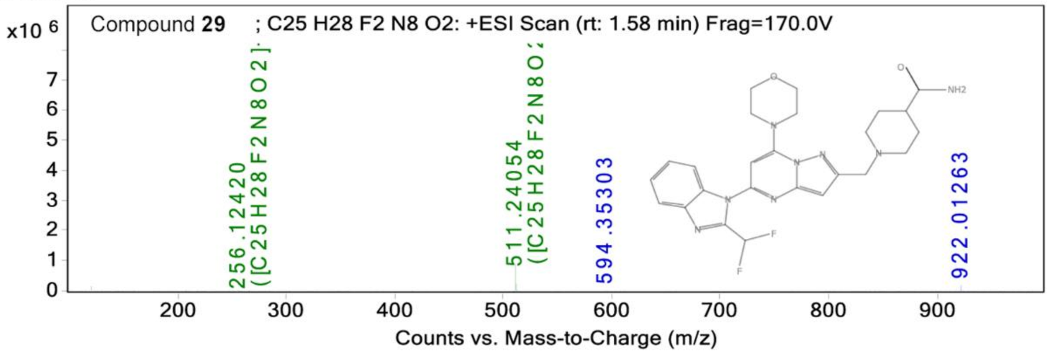

MS Zoomed Spectrum

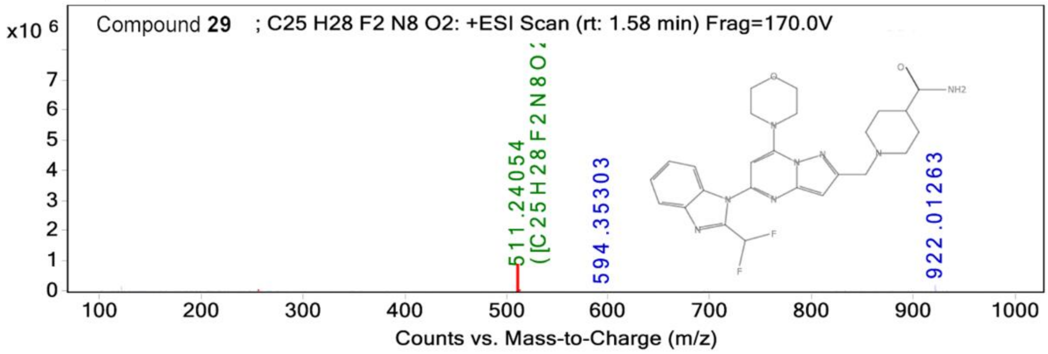

MS Spectrum Peak List

m/z	*Calc m/z*	Diff(ppm)	z	Abund	Formula	Ion
119.02898	96.03812	239392.99	1	15371.62	C3 H6 F2 O	(M+Na)+
121.05147	98.06004	234462.74	1	142227.73	C5 H8 N O	(M+Na)+
158.97415				13291.27		
194.11845	176.08318	102424.71	1	12525.6	C11 H12 O2	(M+NH4)+
256.1242	255.11462	3957.39	2	25005.71	C25 H28 F2 N8 O2	(M+2H)+2
511.24054	510.22978	1981	1	895150.94	C25 H28 F2 N8 O2	(M+H)+
512.24361	512.24039	6.28	1	231365.09	C25 H28 F2 N8 O2	(M+H)+
513.2455	513.24303	4.81	1	34448.2	C25 H28 F2 N8 O2	(M+H)+
922.01263			1	180393.22		
923.01411			1	30642.4		

Compound 30

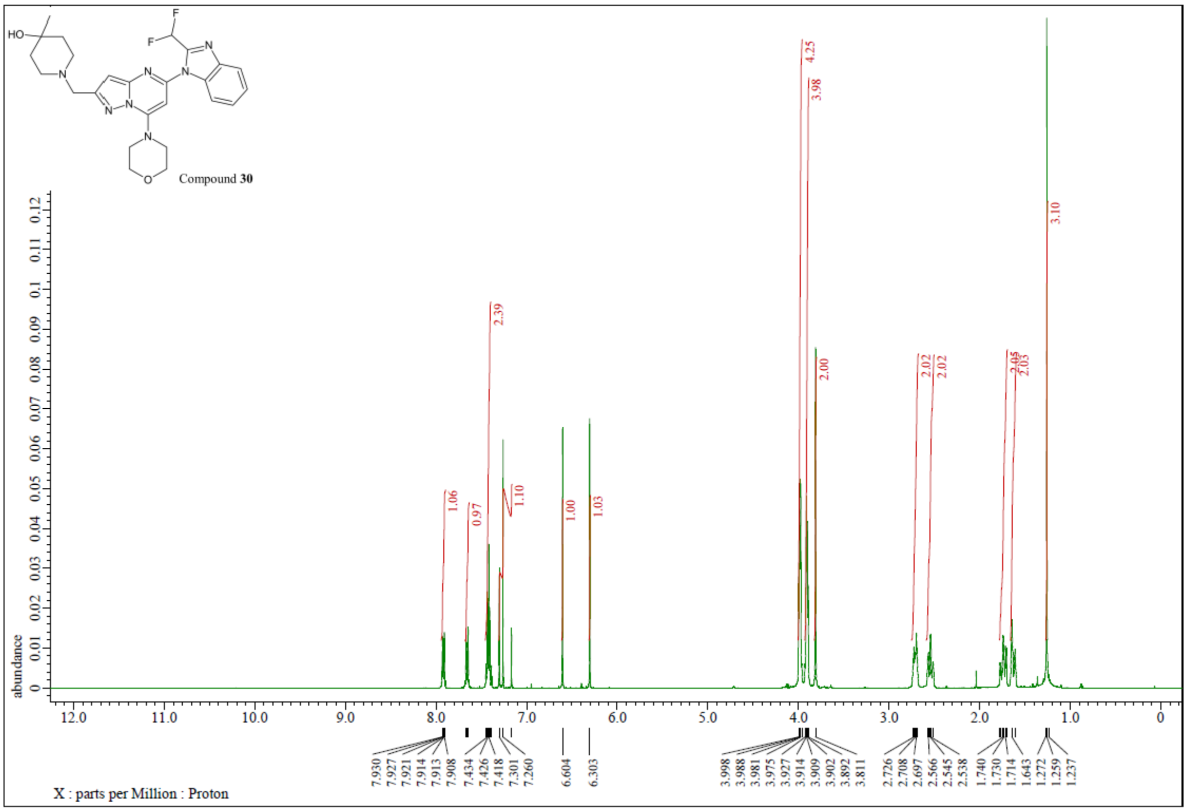

Compound 30
abundance
X : parts per Million : Proton

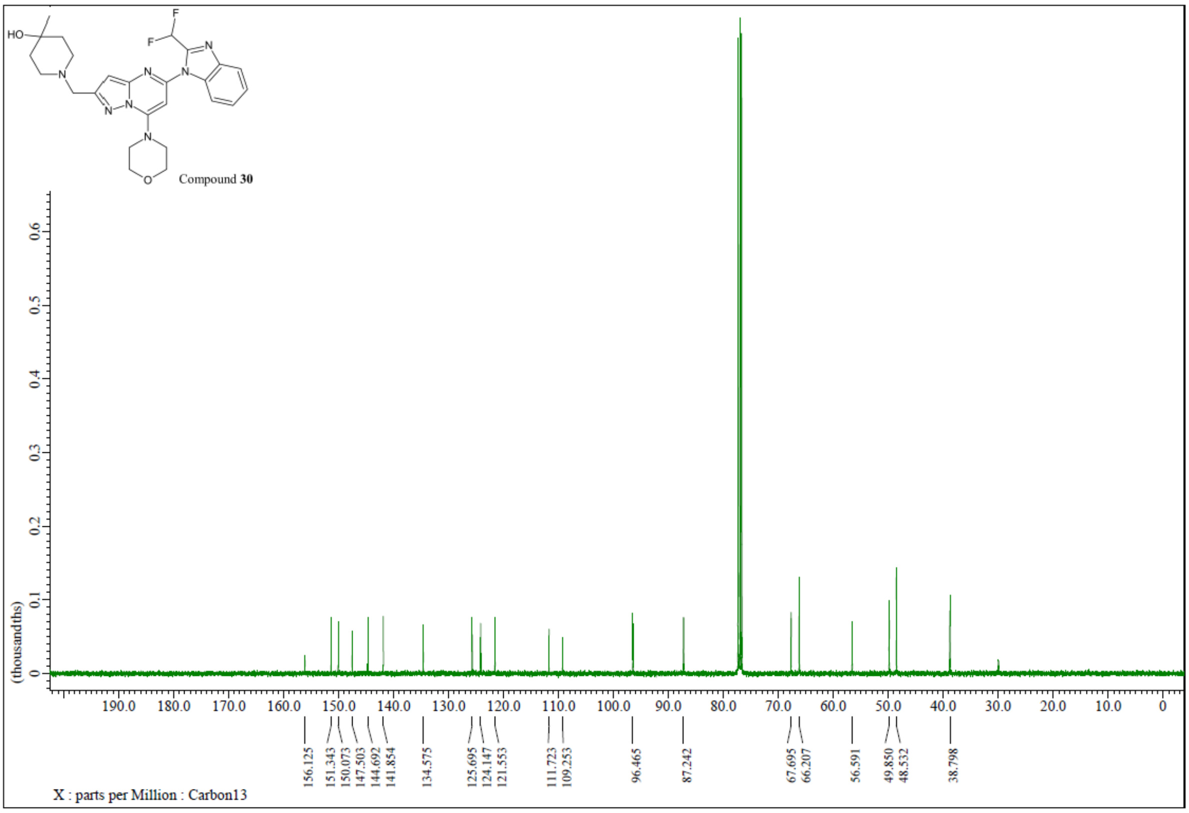

Compound 30
(thousandths)
X : parts per Million : Carbon13

Compound 30

Name	*Obs. m/z*	Obs. RT	Obs. Mass	Tgt Formula	Tgt Mass	Tgt Mass Error	Find Cpds Algorit
Compound **30**	498.24225	1.23	497.23508	C25 H29 F2 N7 O2	497.23508	-0.01	Find by Formula

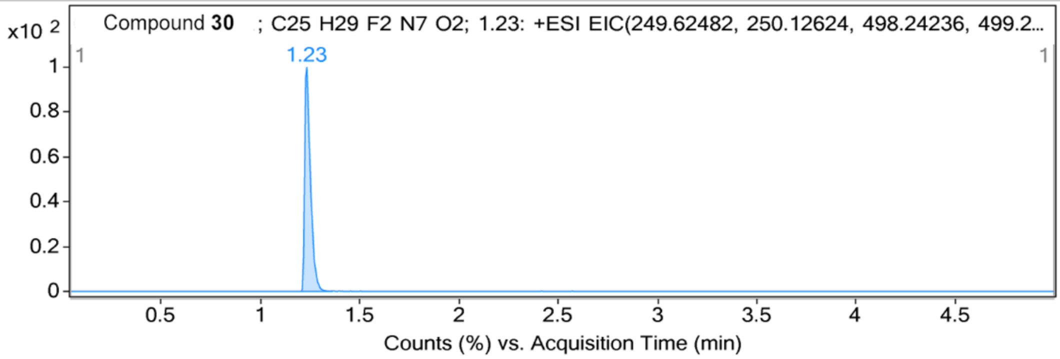

MS Zoomed Spectrum

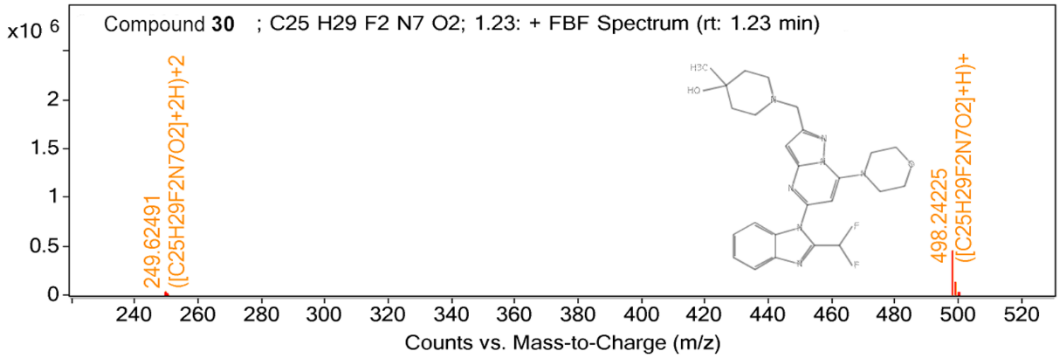

MS Zoomed Spectrum

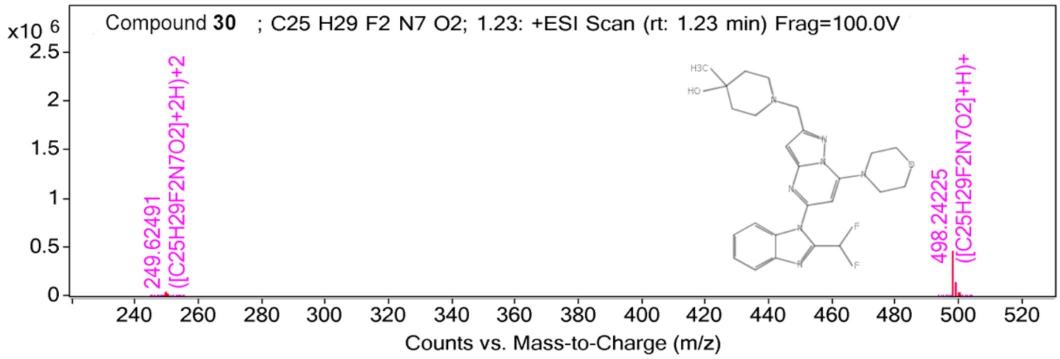

MS Spectrum Peak List

Obs. m/z	Charge	Abund	Formula	Ion/Isotope	Tgt Mass Error (ppm)
249.62491	2	32408.56	C25H29F2N7O2	(M+2H)+2	
250.12641	2	9351.96	C25H29F2N7O2	(M+2H)+2	
250.63006	2	1067.82	C25H29F2N7O2	(M+2H)+2	
498.24225	1	454326	C25H29F2N7O2	(M+H)+	
499.24548	1	127977.54	C25H29F2N7O2	(M+H)+	
500.24771	1	17402.12	C25H29F2N7O2	(M+H)+	
249.62491	2	32408.56	C25H29F2N7O2	(M+2H)+2	0.36
250.12641	2	9351.96	C25H29F2N7O2	(M+2H)+2	0.67
250.63006	2	1067.82	C25H29F2N7O2	(M+2H)+2	9.85
498.24225	1	454326	C25H29F2N7O2	(M+H)+	-0.21
499.24548	1	127977.54	C25H29F2N7O2	(M+H)+	0.55
500.24771	1	17402.12	C25H29F2N7O2	(M+H)+	-0.41

--- End Of Report ---

Compound 31

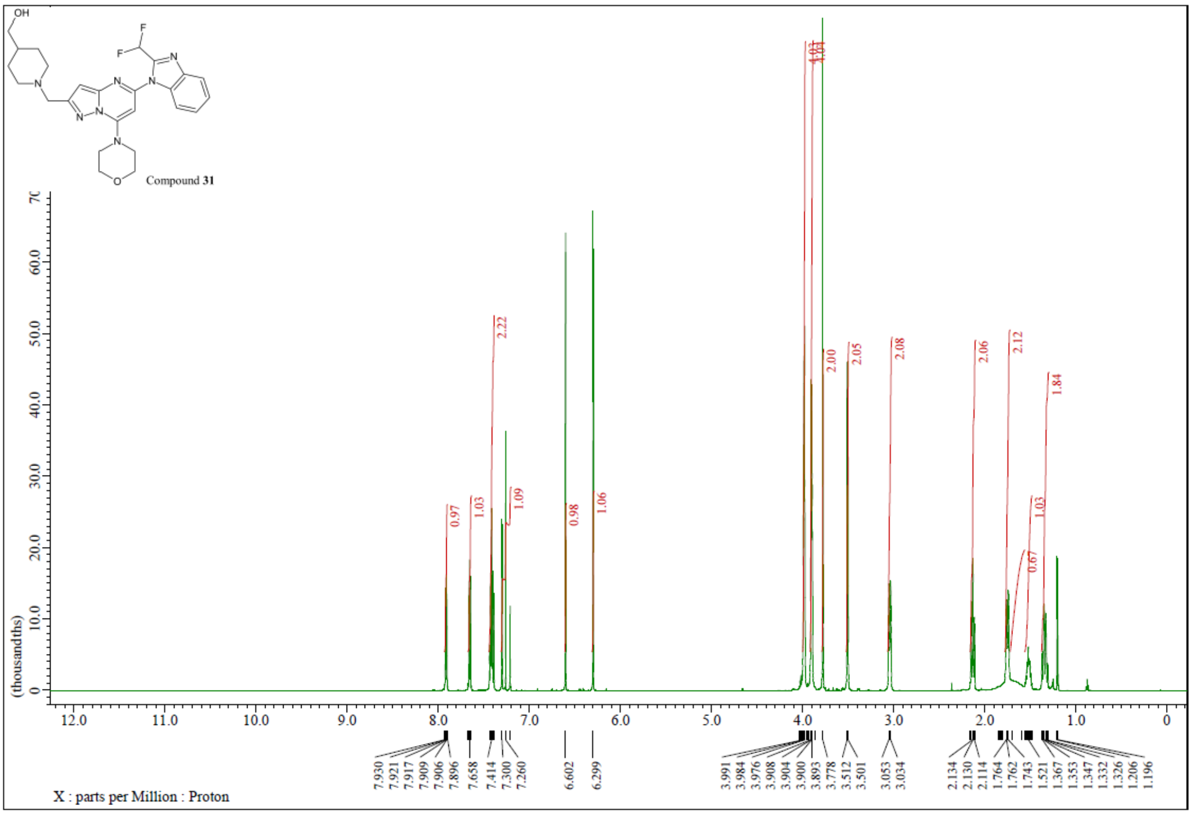
Compound 31
X : parts per Million : Proton
(thousandths)

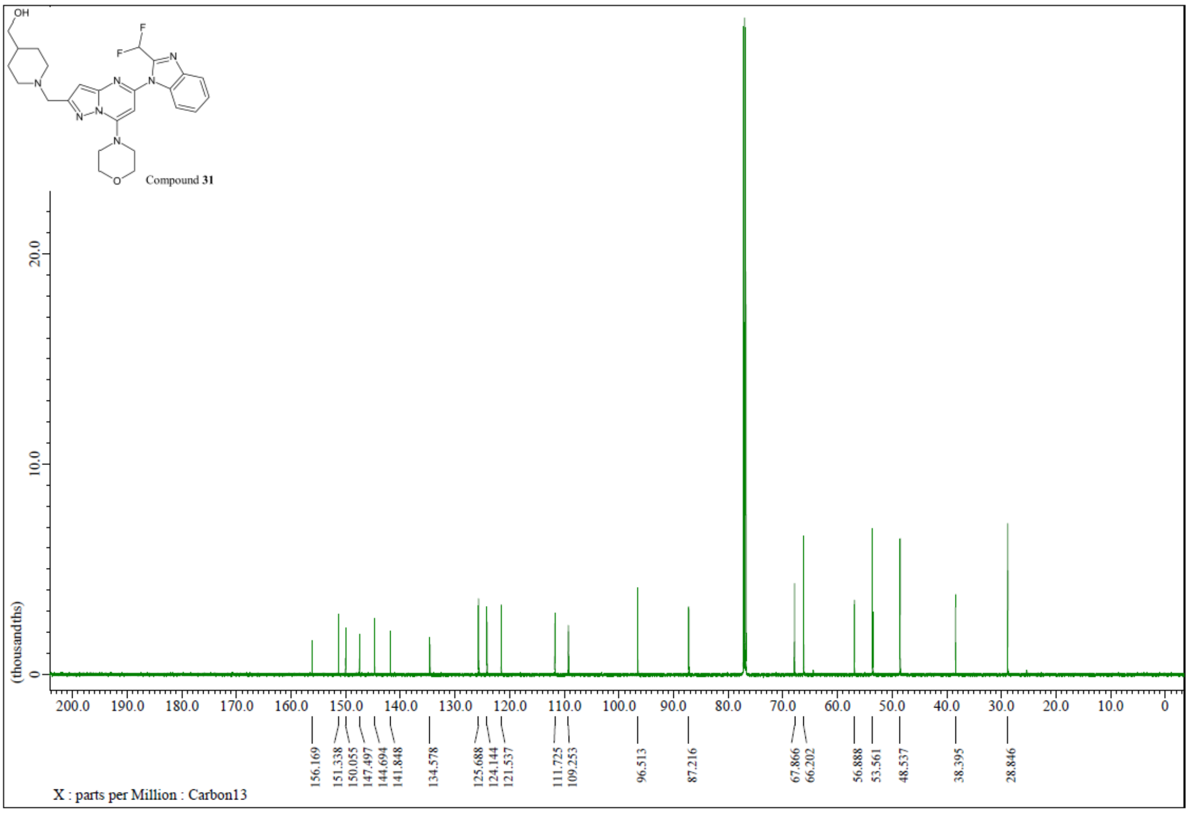
Compound 31
X : parts per Million : Carbon13
(thousandths)

Compound 31

Name	*Obs. m/z*	Obs. RT	Obs. Mass	Tgt Formula	Tgt Mass	Tgt Mass Error	Find Cpds Algorit
Compound **31**	249.62509	1.21	497.23538	C25 H29 F2 N7 O2	497.23508	0.6	Find by Formula

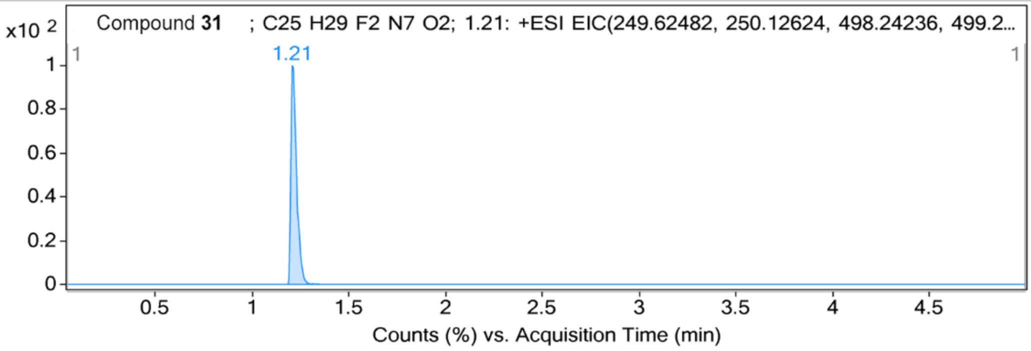

MS Zoomed Spectrum

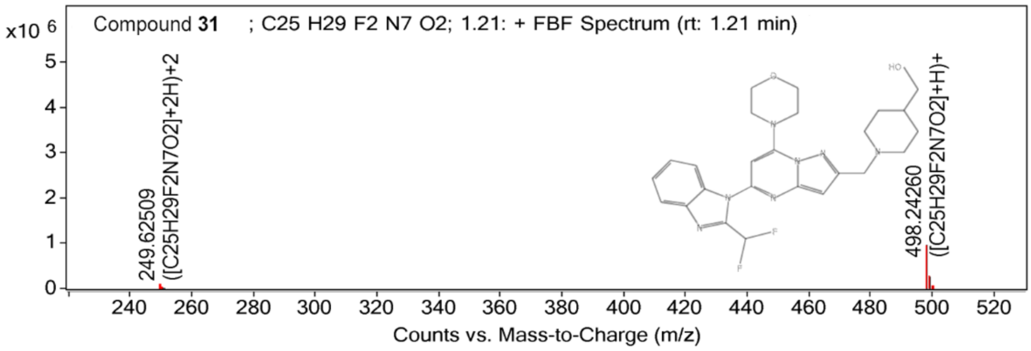

MS Zoomed Spectrum

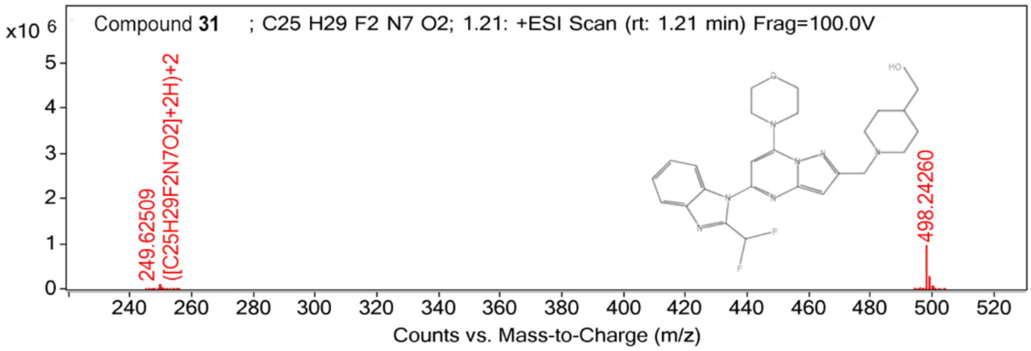

MS Spectrum Peak List

Obs. m/z	Charge	Abund	Formula	Ion/Isotope	Tgt Mass Error (ppm)
249.62509	2	93481.14	C25H29F2N7O2	(M+2H)+2	
250.12632	2	26983.34	C25H29F2N7O2	(M+2H)+2	
250.62759	2	4998.15	C25H29F2N7O2	(M+2H)+2	
251.12641	2	537.79	C25H29F2N7O2	(M+2H)+2	
498.2426	1	954572.56	C25H29F2N7O2	(M+H)+	
499.24579	1	250903.33	C25H29F2N7O2	(M+H)+	
500.24755	1	41231.06	C25H29F2N7O2	(M+H)+	
249.62509	2	93481.14	C25H29F2N7O2	(M+2H)+2	1.11
250.12632	2	26983.34	C25H29F2N7O2	(M+2H)+2	0.32
250.62759	2	4998.15	C25H29F2N7O2	(M+2H)+2	-0.03
251.12641	2	537.79	C25H29F2N7O2	(M+2H)+2	-9.93
498.2426		955096.81			0.48
498.2426	1	954572.56	C25H29F2N7O2	(M+H)+	0.48
499.24579	1	250903.33	C25H29F2N7O2	(M+H)+	1.16
500.24755	1	41231.06	C25H29F2N7O2	(M+H)+	-0.72

--- End Of Report ---

Compound 32

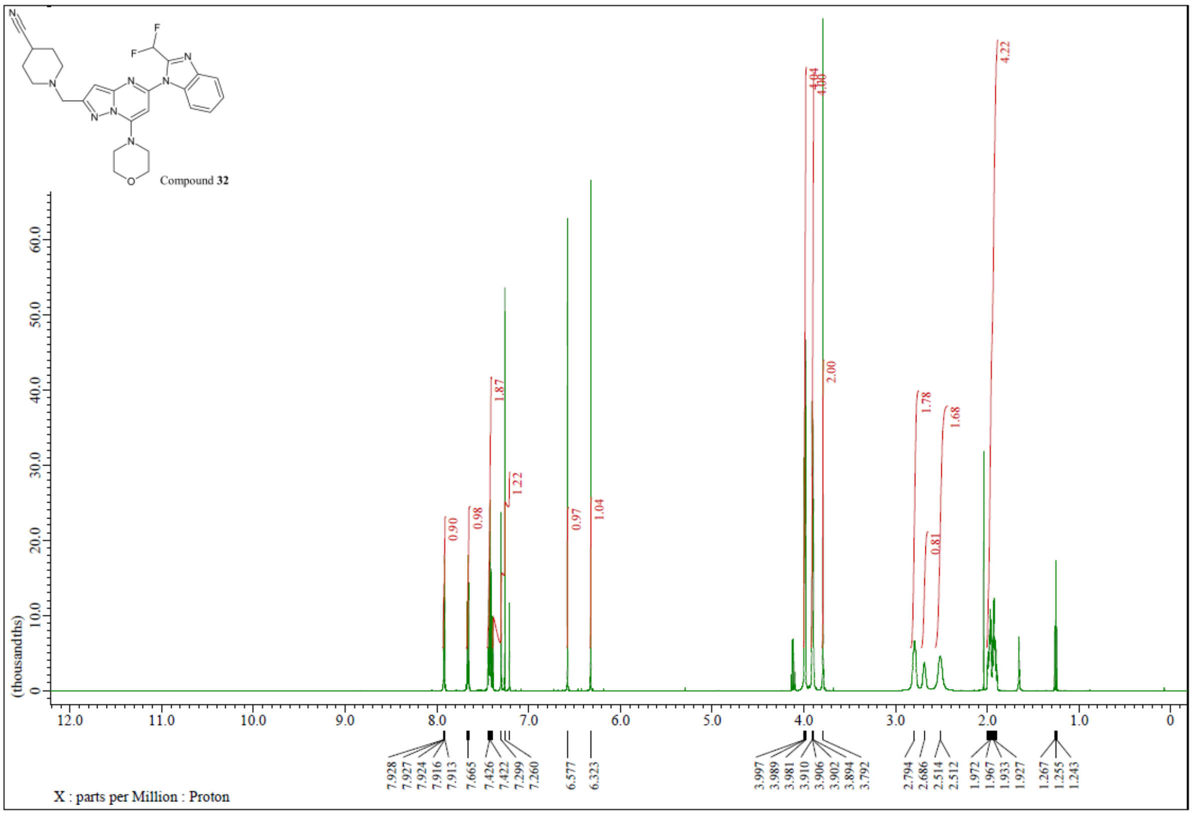
Compound 32
(thousandths)
X : parts per Million : Proton
7.928 7.927 7.924 7.916 7.913 7.665 7.426 7.422 7.299 7.260 6.577 6.323
3.997 3.989 3.981 3.910 3.906 3.902 3.894 3.792 2.794 2.686 2.514 2.512 1.972 1.967 1.933 1.927 1.267 1.255 1.243
0.90 0.98 1.87 1.22 0.97 1.04 4.04 4.00 2.00 1.78 0.81 1.68 4.22

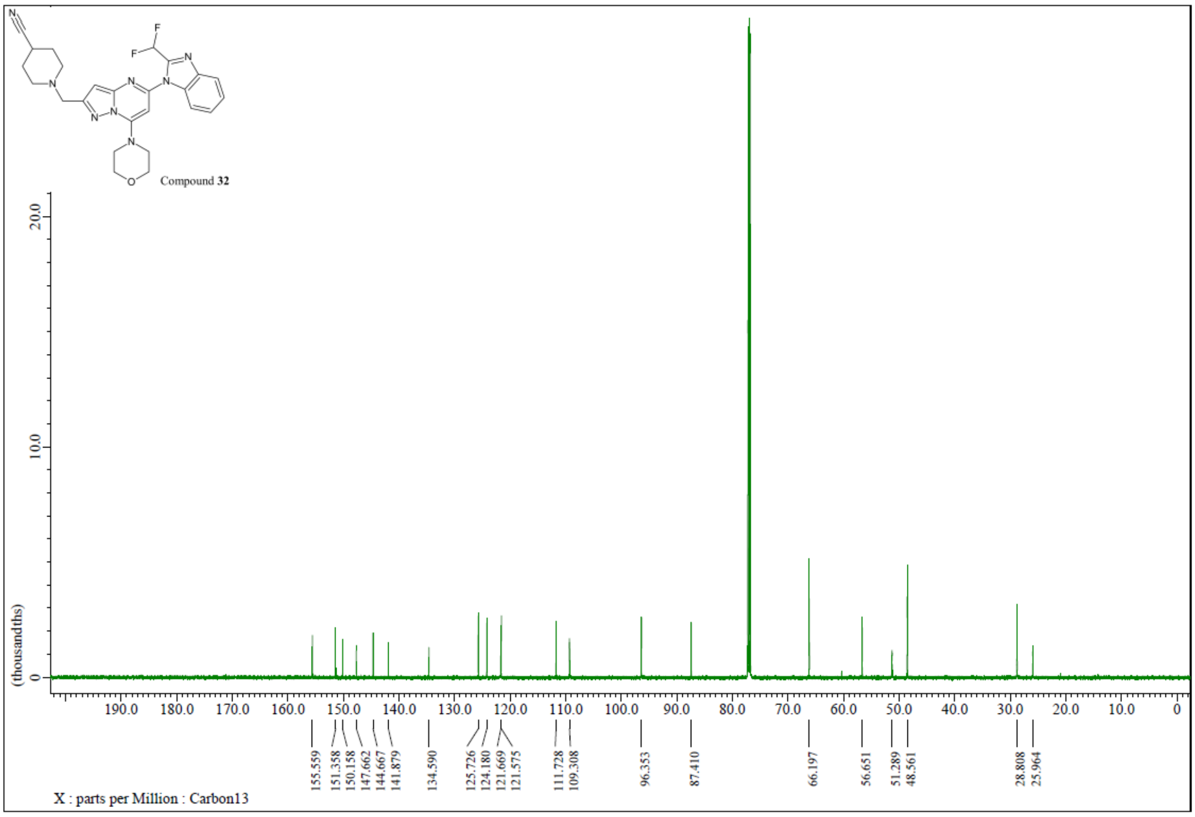
Compound 32
(thousandths)
X : parts per Million : Carbon13
155.559 151.358 150.158 147.662 144.667 141.879 134.590 125.726 124.180 121.669 121.575 111.728 109.308 96.353 87.410 66.197 56.651 51.289 48.561 28.808 25.964

Compound 32

Name	*Obs. m/z*	Obs. RT	Obs. Mass	Tgt Formula	Tgt Mass	Tgt Mass Error	Find Cpds Algorit
Compound **32**	247.11734	1.28	492.22059	C25 H26 F2 N8 O	492.21976	1.67	Find by Formula

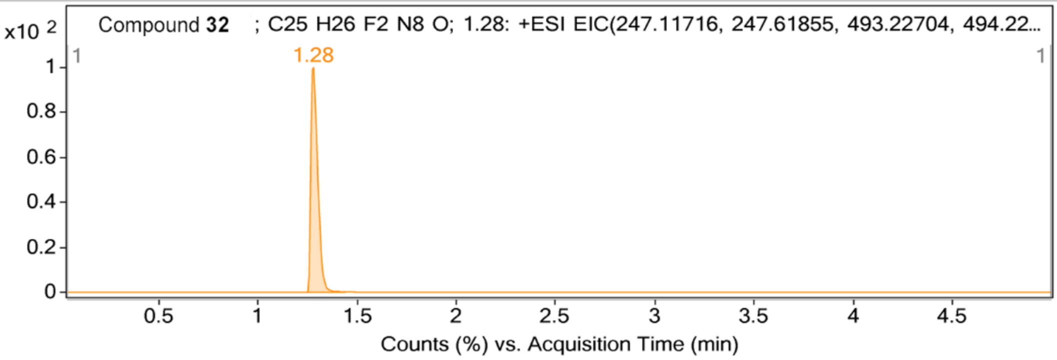

MS Zoomed Spectrum

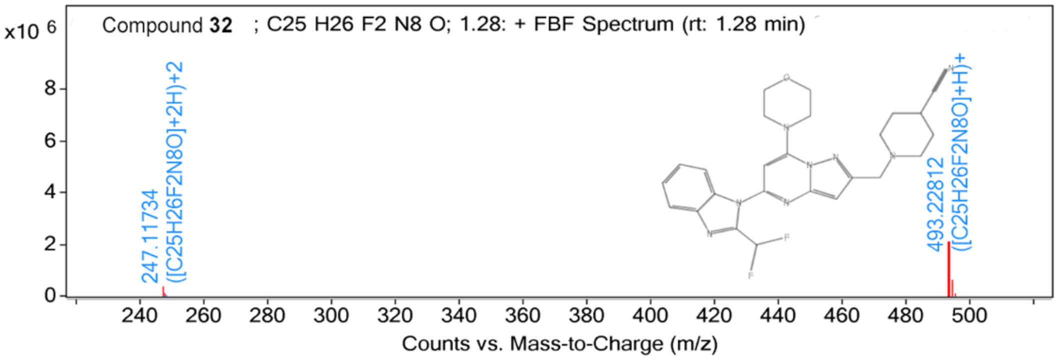

MS Zoomed Spectrum

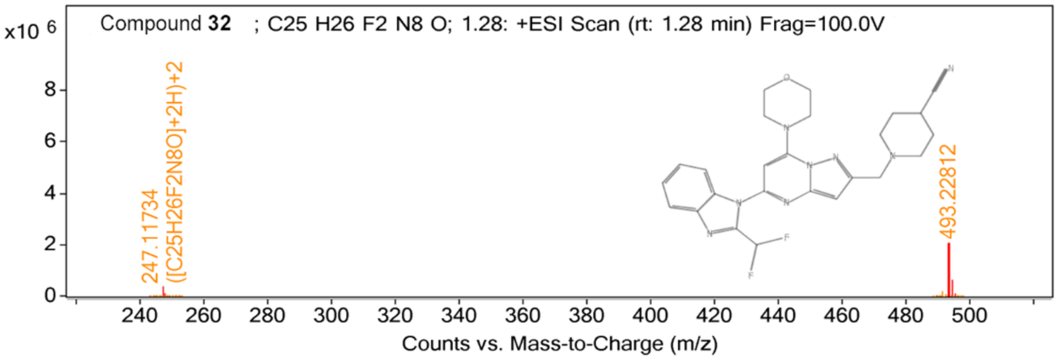

MS Spectrum Peak List

Obs. m/z	Charge	Abund	Formula	Ion/Isotope	Tgt Mass Error (ppm)
247.11734	2	344030.63	C25H26F2N8O	(M+2H)+2	
247.61913	2	93506.33	C25H26F2N8O	(M+2H)+2	
248.11993	2	15524.01	C25H26F2N8O	(M+2H)+2	
493.22812	1	2066621	C25H26F2N8O	(M+H)+	
494.23008	1	564287.69	C25H26F2N8O	(M+H)+	
495.23248	1	79639.52	C25H26F2N8O	(M+H)+	
247.11734	2	344030.63	C25H26F2N8O	(M+2H)+2	0.72
247.61913	2	93506.33	C25H26F2N8O	(M+2H)+2	2.36
248.11993	2	15524.01	C25H26F2N8O	(M+2H)+2	0.14
493.22812	1	2066621	C25H26F2N8O	(M+H)+	2.18
493.22812		2068672.75			
494.23008	1	564287.69	C25H26F2N8O	(M+H)+	0.52
495.23248	1	79639.52	C25H26F2N8O	(M+H)+	-0.06

--- End Of Report ---

Compound 33

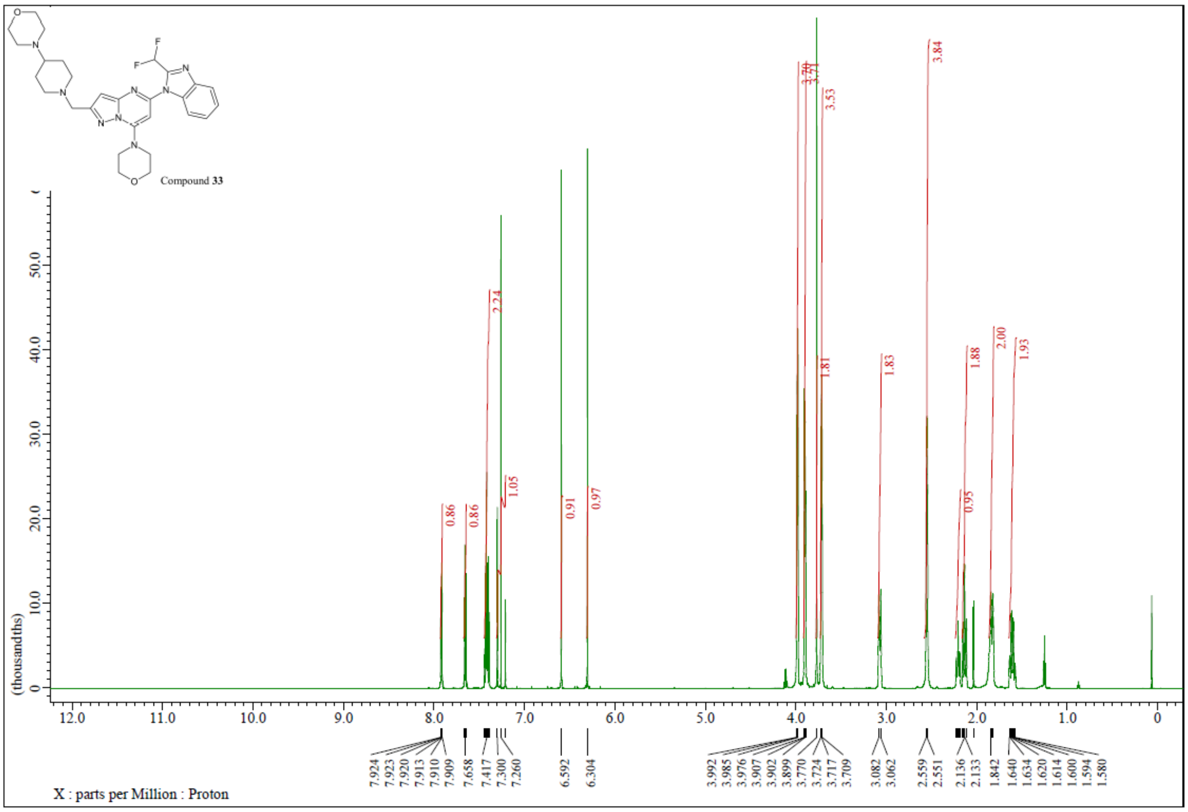

Compound 33
(thousandths)
X : parts per Million : Proton
7.924
7.923
7.920
7.913
7.910
7.909
7.658
7.417
7.300
7.260
6.592
6.304
3.992
3.985
3.976
3.907
3.902
3.899
3.770
3.724
3.717
3.709
3.082
3.062
2.559
2.551
2.136
2.133
1.842
1.640
1.634
1.620
1.614
1.600
1.594
1.580

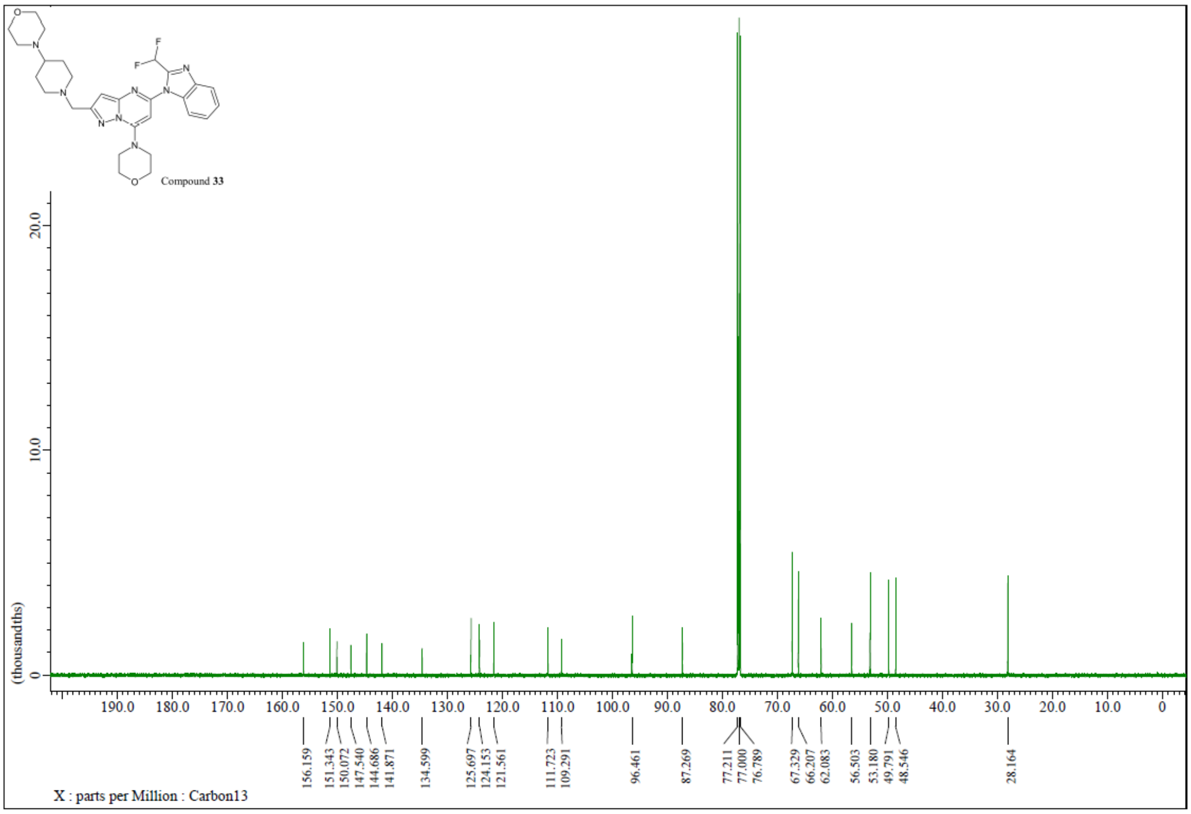

Compound 33
(thousandths)
X : parts per Million : Carbon13
156.159
151.343
150.072
147.540
144.686
141.871
134.599
125.697
124.153
121.561
111.723
109.291
96.461
87.269
77.211
77.000
76.789
67.329
66.207
62.083
56.503
53.180
49.791
48.546
28.164

Compound 33

Name	Obs. m/z	Obs. RT	Obs. Mass	Tgt Formula	Tgt Mass	Tgt Mass Error	Find Cpds Algorit
Compound **33**	553.28461	0.96	552.27741	C28 H34 F2 N8 O2	552.27728	0.24	Find by Formula

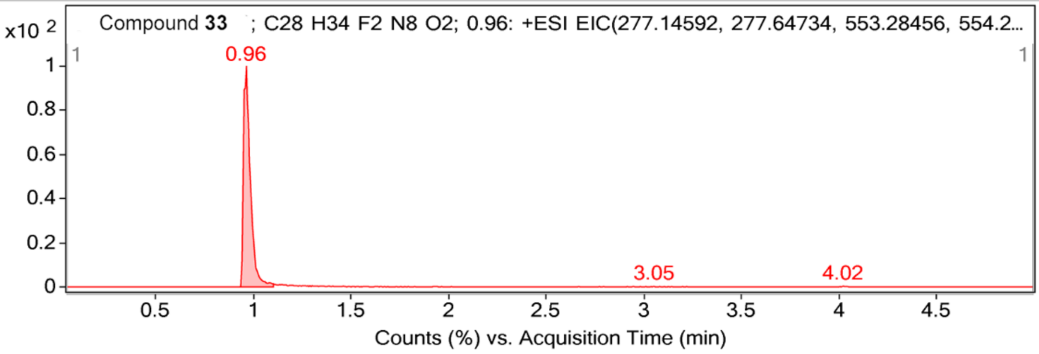

MS Zoomed Spectrum

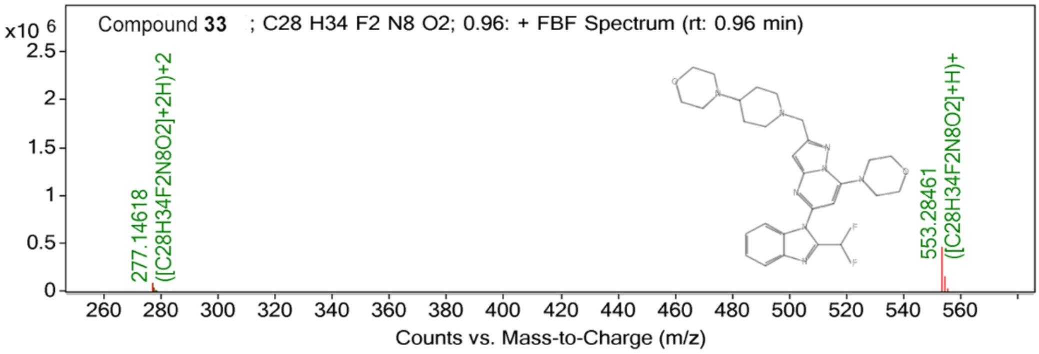

MS Zoomed Spectrum

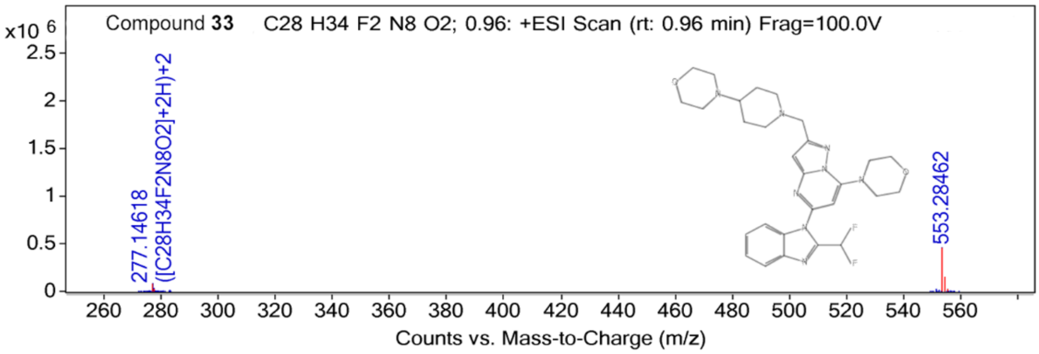

MS Spectrum Peak List

Obs. m/z	Charge	Abund	Formula	Ion/Isotope	Tgt Mass Error (ppm)
277.14618	2	79318.7	C28H34F2N8O2	(M+2H)+2	
277.64732	2	29703.31	C28H34F2N8O2	(M+2H)+2	
278.14977	2	5296.34	C28H34F2N8O2	(M+2H)+2	
278.65038	2	521.16	C28H34F2N8O2	(M+2H)+2	
553.28461	1	456124.09	C28H34F2N8O2	(M+H)+	
554.28756	1	135750.58	C28H34F2N8O2	(M+H)+	
555.28985	1	23141.17	C28H34F2N8O2	(M+H)+	
277.14618	2	79318.7	C28H34F2N8O2	(M+2H)+2	0.96
277.64732	2	29703.31	C28H34F2N8O2	(M+2H)+2	-0.06
278.14977	2	5296.34	C28H34F2N8O2	(M+2H)+2	3.85
278.65038	2	521.16	C28H34F2N8O2	(M+2H)+2	1.29
553.28461	1	456124.09	C28H34F2N8O2	(M+H)+	0.11
553.28462		458500.22			
554.28756	1	135750.58	C28H34F2N8O2	(M+H)+	0.29
555.28985	1	23141.17	C28H34F2N8O2	(M+H)+	-0.5

--- End Of Report ---

Compound 34

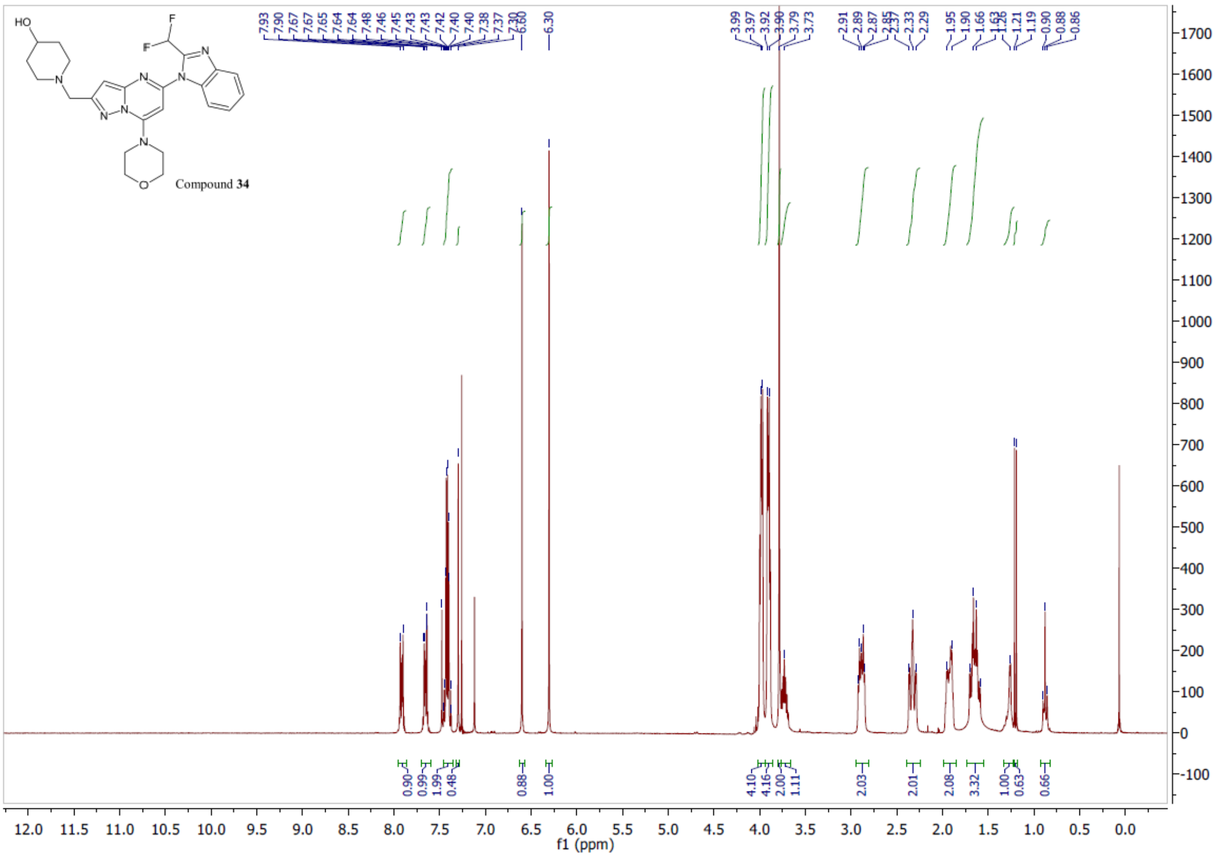

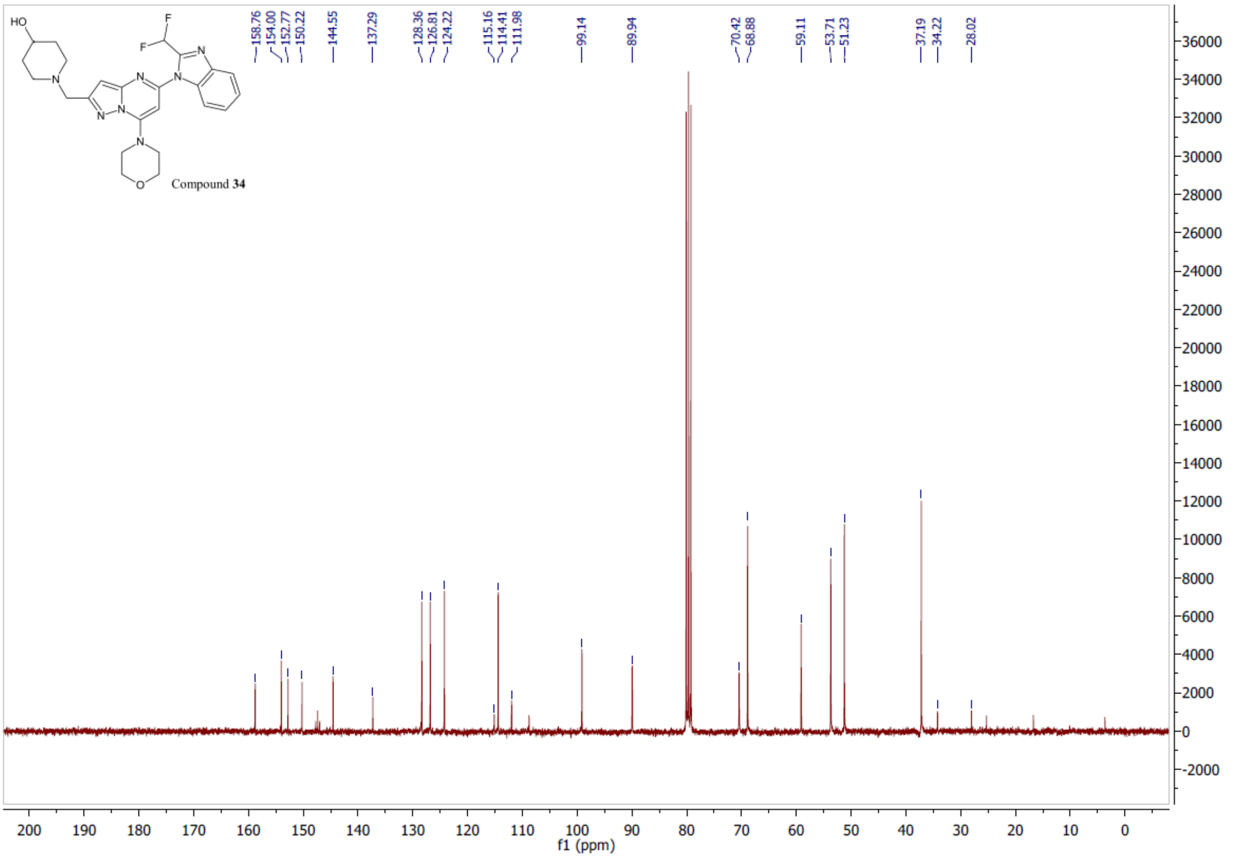

Compound 34

Name	*Obs. m/z*	Obs. RT	Obs. Mass	Tgt Formula	Tgt Mass	Tgt Mass Error	Find Cpds Algorit
Compound **34**	242.61746	1.19	483.21994	C24 H27 F2 N7 O2	483.21943	1.06	Find by Formula

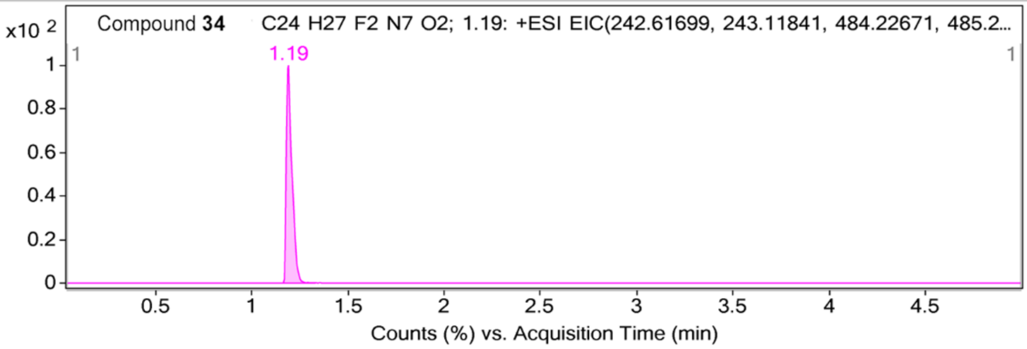

MS Zoomed Spectrum

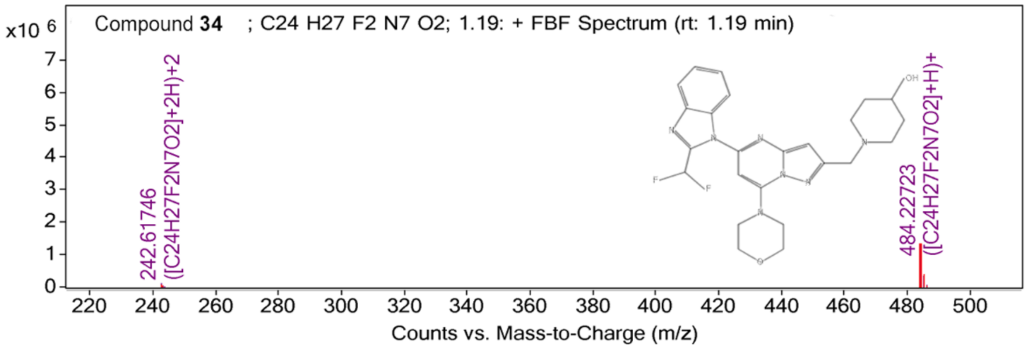

MS Zoomed Spectrum

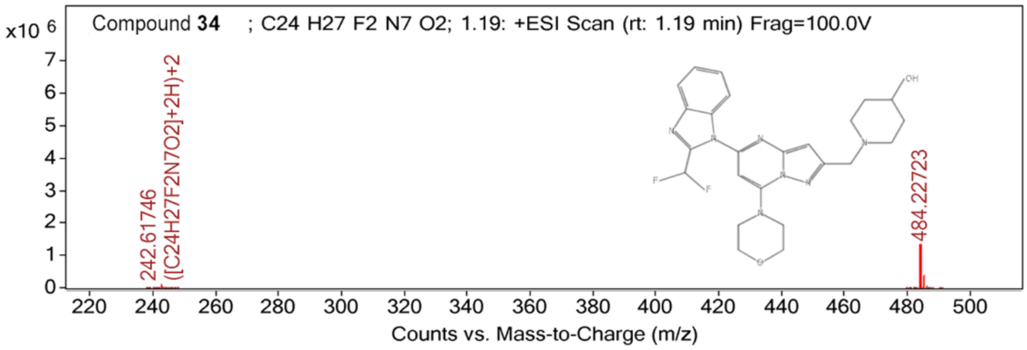

MS Spectrum Peak List

Obs. m/z	Charge	Abund	Formula	Ion/Isotope	Tgt Mass Error (ppm)
242.61746	2	100150.59	C24H27F2N7O2	(M+2H)+2	
243.1188	2	28050.54	C24H27F2N7O2	(M+2H)+2	
243.62017	2	4474.7	C24H27F2N7O2	(M+2H)+2	
484.22723	1	1327642.88	C24H27F2N7O2	(M+H)+	
485.22988	1	339432.13	C24H27F2N7O2	(M+H)+	
486.23245	1	46026.59	C24H27F2N7O2	(M+H)+	
242.61746	2	100150.59	C24H27F2N7O2	(M+2H)+2	1.93
243.1188	2	28050.54	C24H27F2N7O2	(M+2H)+2	1.6
243.62017	2	4474.7	C24H27F2N7O2	(M+2H)+2	1.74
484.22723	1	1327642.88	C24H27F2N7O2	(M+H)+	1.09
484.22723		1328394.5			
485.22988	1	339432.13	C24H27F2N7O2	(M+H)+	0.7
486.23245	1	46026.59	C24H27F2N7O2	(M+H)+	0.48

--- End Of Report ---

Compound 35

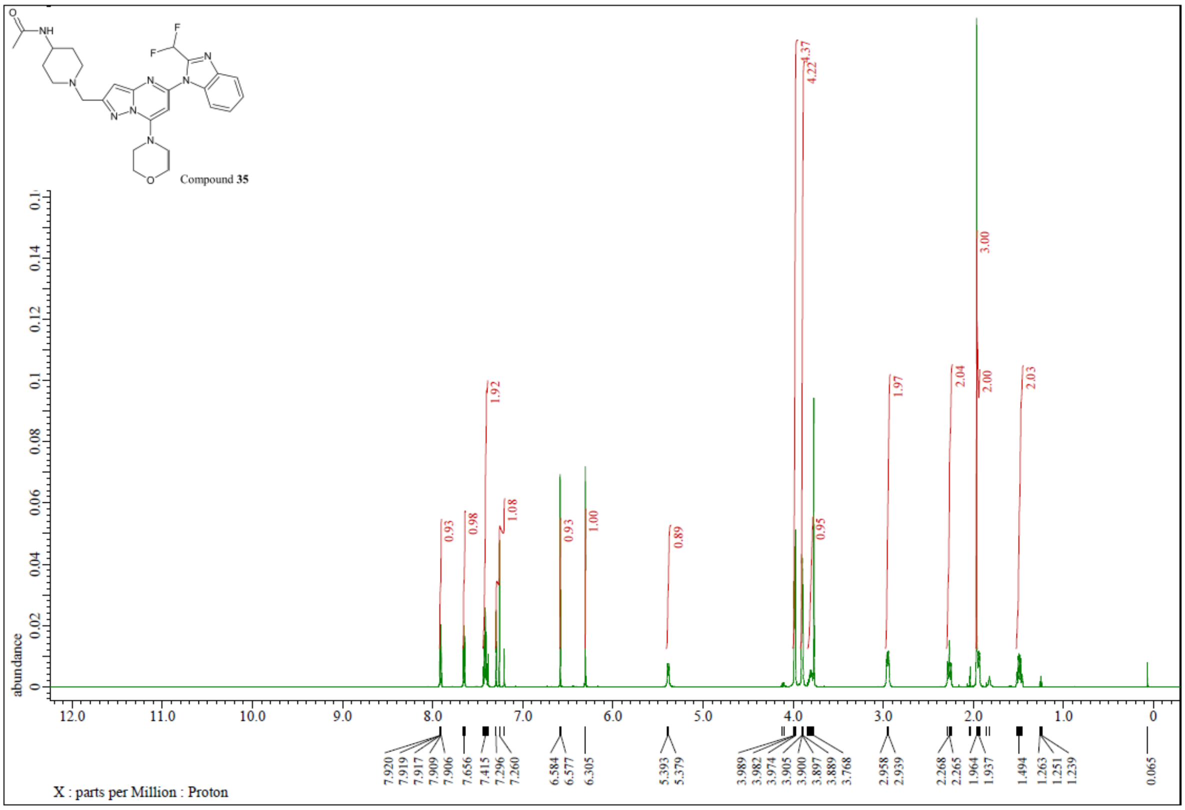
Compound 35
abundance
X : parts per Million : Proton

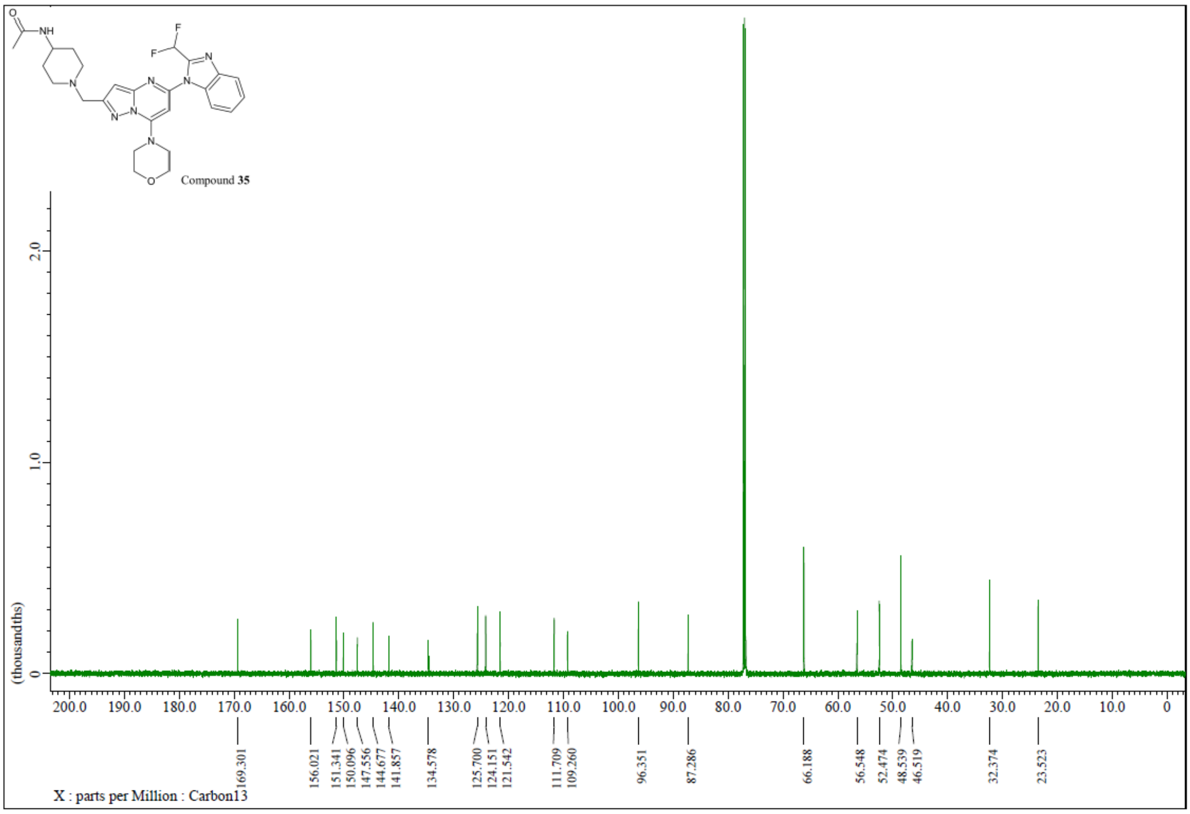
Compound 35
(thousandths)
X : parts per Million : Carbon13

Compound 35

Fragmentor Voltage	170	Collision Energy	0	Ionization Mode	ESI

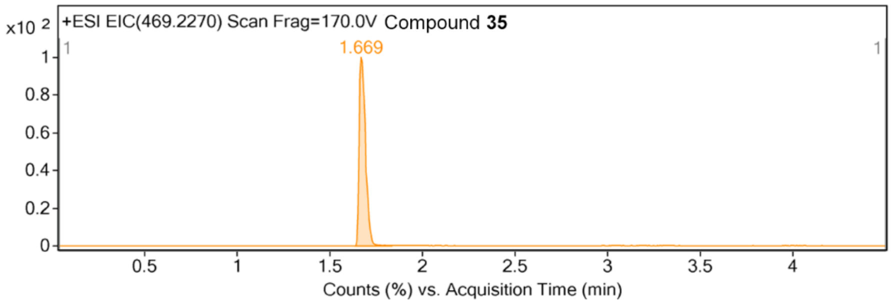

Integration Peak List

Peak	Start	RT	End	Height	Area	Area %
1	1.634	1.669	1.835	3781886.04	9105302.42	100

Spectra

Spectrum Source	Fragmentor Voltage	Collision Energy	Ionization Mode
Peak (1) in "+ EIC(469.2270) Scan"	170	0	ESI

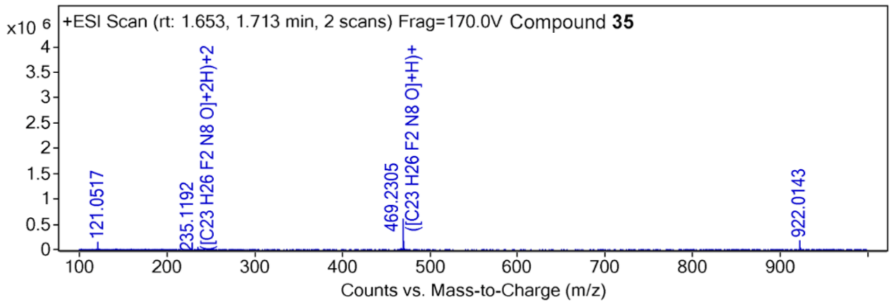

Peak List

m/z	z	Abund	Name	Formula	Ion	Score (DB)	Hits (DB)
469.2305	1	601001.5	Compound **35**	C23 H26 F2 N8 O	(M+H)+	97.11	2

--- End Of Report ---

Compound 36

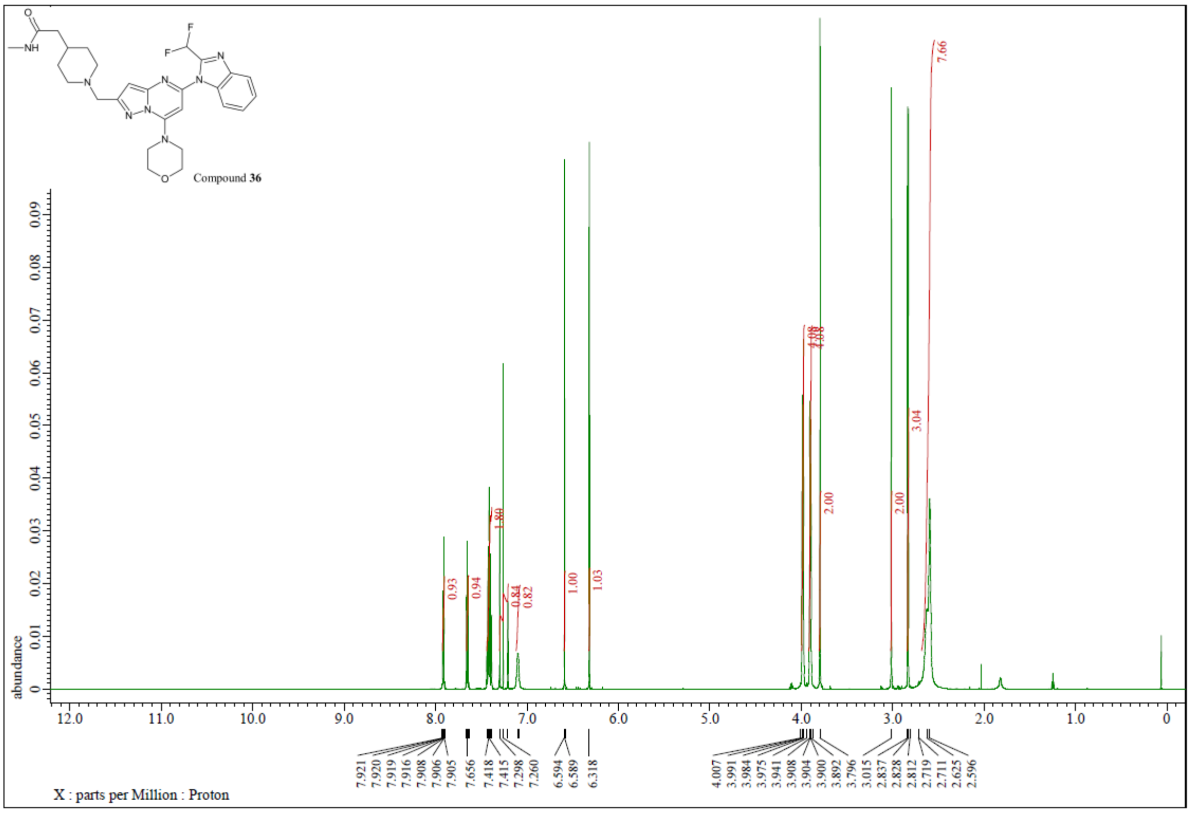
Compound 36
abundance
X : parts per Million : Proton
7.921 7.920 7.919 7.916 7.908 7.906 7.905 7.656 7.418 7.415 7.298 7.260 6.594 6.589 6.318
4.007 3.991 3.984 3.975 3.941 3.908 3.904 3.900 3.892 3.796 3.015 2.837 2.828 2.812 2.719 2.711 2.625 2.596
0.93 0.94 1.89 0.84 0.82 1.00 1.03 2.00 2.00 3.04 7.66

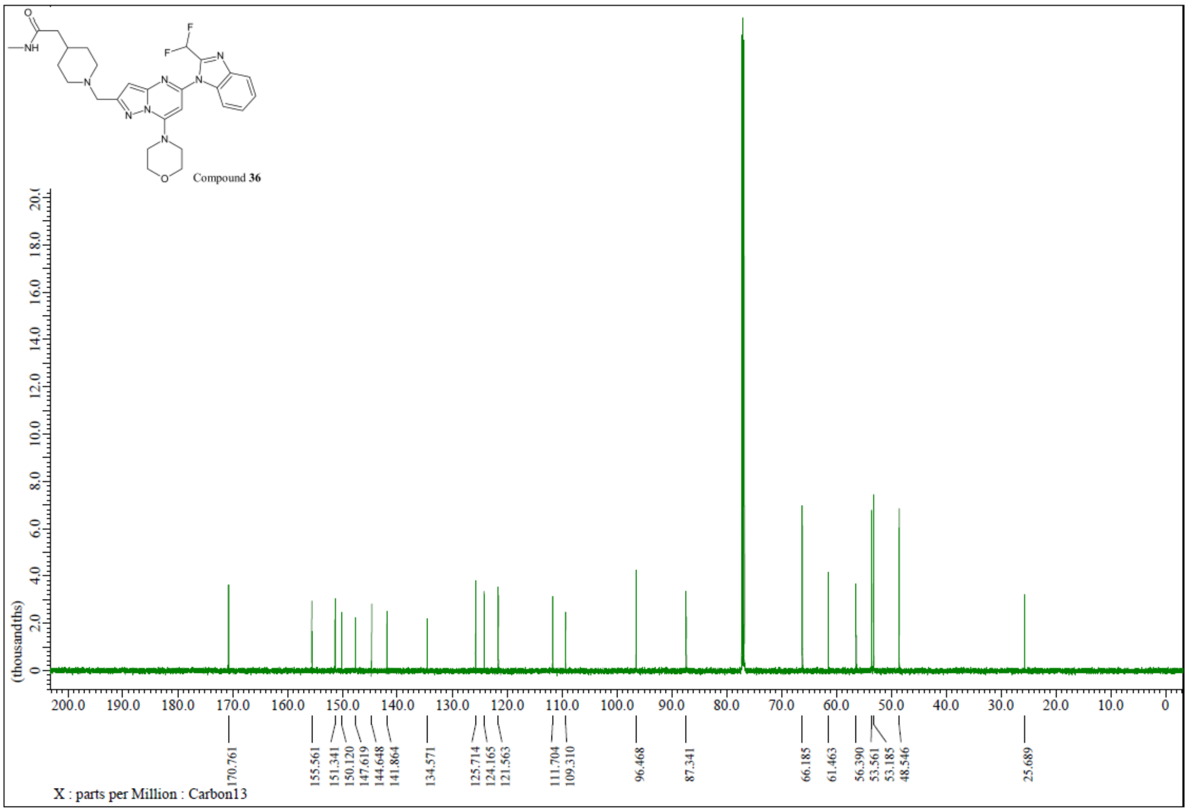
Compound 36
(thousandths)
X : parts per Million : Carbon13
170.761 155.561 151.341 150.120 147.619 144.648 141.864 134.571 125.714 124.165 121.563 111.704 109.310 96.468 87.341 66.185 61.463 56.390 53.561 53.185 48.546 25.689

Compound 36

Name	*Obs. m/z*	Obs. RT	Obs. Mass	Tgt Formula	Tgt Mass	Tgt Mass Error	Find Cpds Algorit
Compound **36**	270.63589	1.2	539.2572	C26 H31 F2 N9 O2	539.25688	0.59	Find by Formula

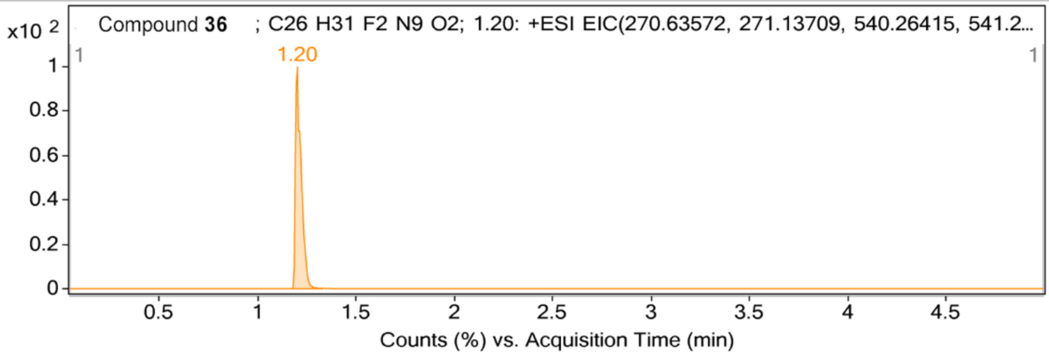

MS Zoomed Spectrum

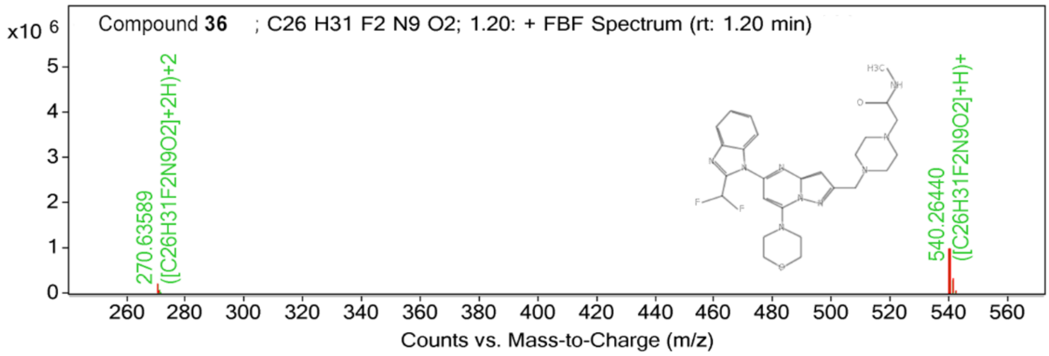

MS Zoomed Spectrum

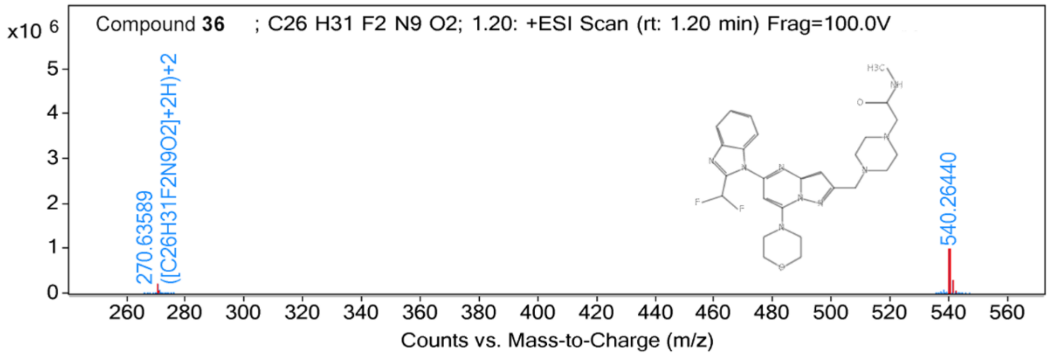

MS Spectrum Peak List

Obs. m/z	Charge	Abund	Formula	Ion/Isotope	Tgt Mass Error (ppm)
270.63589	2	194345.84	C26H31F2N9O2	(M+2H)+2	
271.13742	2	59180.25	C26H31F2N9O2	(M+2H)+2	
271.63847	2	11750.46	C26H31F2N9O2	(M+2H)+2	
540.2644	1	976966.31	C26H31F2N9O2	(M+H)+	
541.26743	1	275591.88	C26H31F2N9O2	(M+H)+	
542.2695	1	40836.08	C26H31F2N9O2	(M+H)+	
270.63589	2	194345.84	C26H31F2N9O2	(M+2H)+2	0.64
271.13742	2	59180.25	C26H31F2N9O2	(M+2H)+2	1.23
271.63847	2	11750.46	C26H31F2N9O2	(M+2H)+2	0.28
540.2644	1	976966.31	C26H31F2N9O2	(M+H)+	0.46
540.2644		977749.31			
541.26743	1	275591.88	C26H31F2N9O2	(M+H)+	1
542.2695	1	40836.08	C26H31F2N9O2	(M+H)+	-0.02

--- End Of Report ---

Compound 37

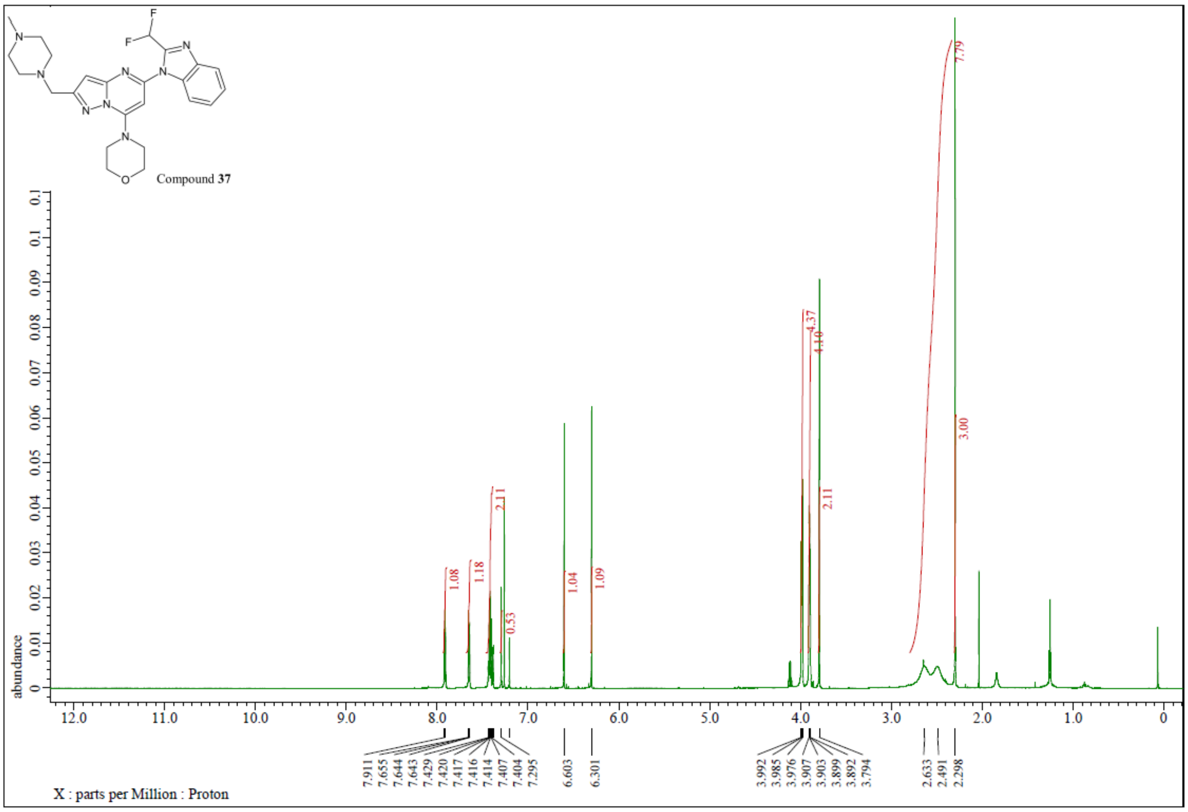
Compound 37
abundance
X : parts per Million : Proton
7.911
7.655
7.644
7.643
7.429
7.420
7.417
7.416
7.414
7.407
7.404
7.295
6.603
6.301
3.992
3.985
3.976
3.907
3.903
3.899
3.892
3.794
2.633
2.491
2.298
1.08
1.18
2.11
0.53
1.04
1.09
4.37
4.10
2.11
3.00
7.79

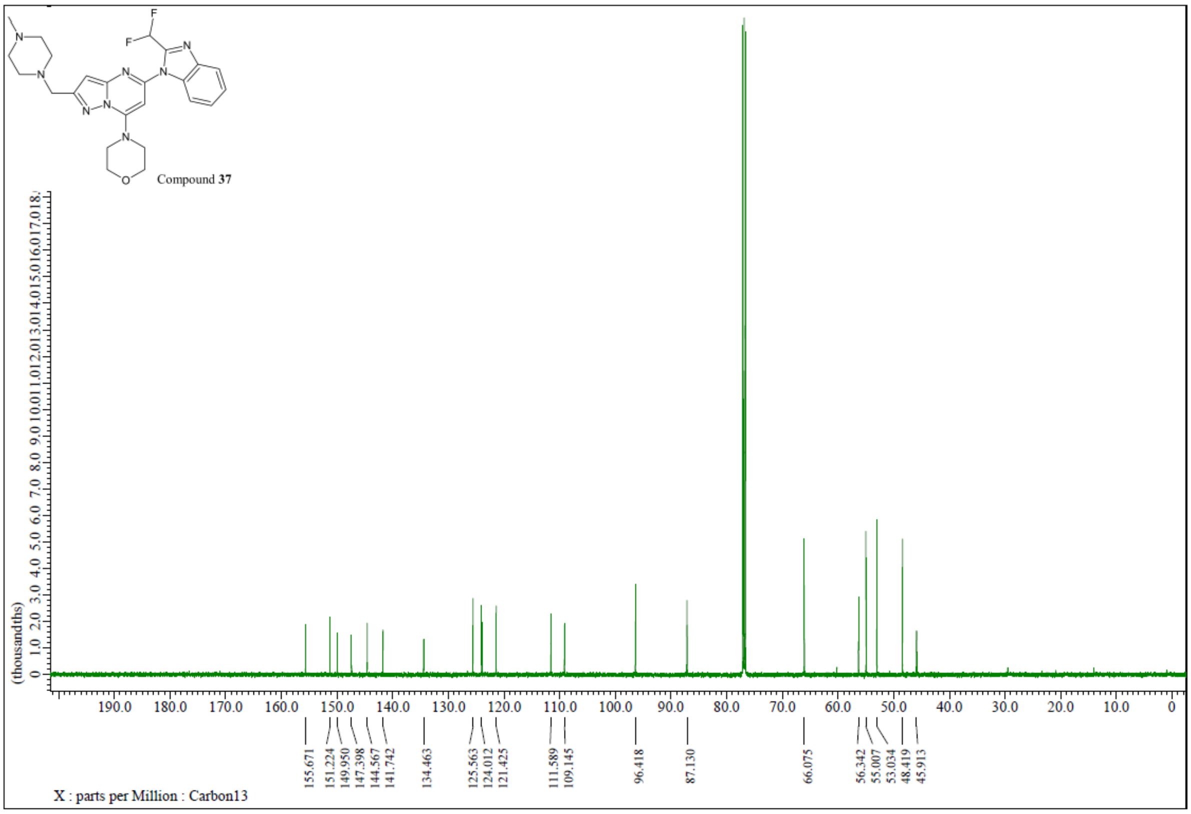
Compound 37
(thousandths)
X : parts per Million : Carbon13
155.671
151.224
149.950
147.398
144.567
141.742
134.463
125.563
124.012
121.425
111.589
109.145
96.418
87.130
66.075
56.342
55.007
53.034
48.419
45.913

Compound 37

Name	*Obs. m/z*	Obs. RT	Obs. Mass	Tgt Formula	Tgt Mass	Tgt Mass Error	Find Cpds Algorith
Compound **37**	242.12535	1.74	482.23576	C24 H28 F2 N8 O	482.23541	0.72	Find by Formula

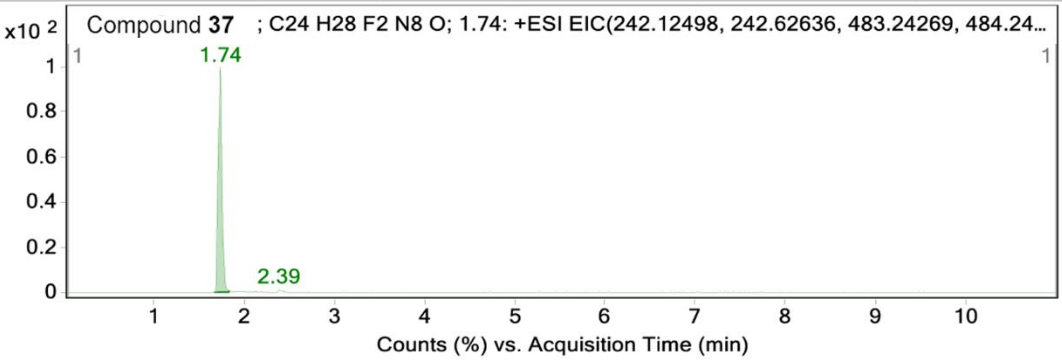

MS Zoomed Spectrum

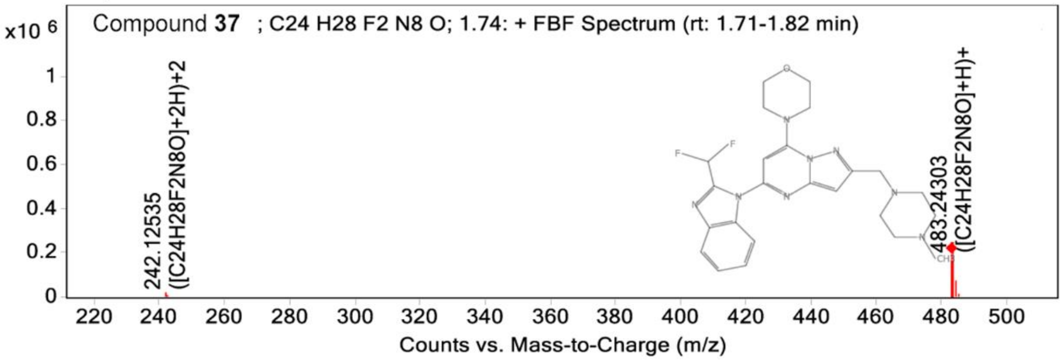

MS Zoomed Spectrum

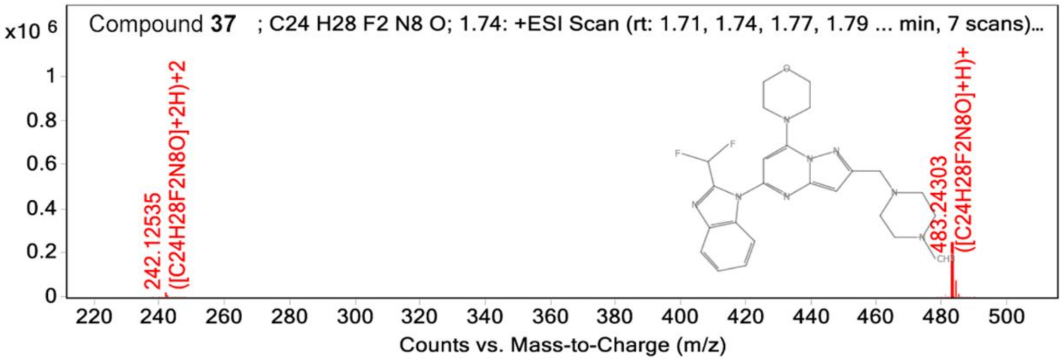

MS Spectrum Peak List

Obs. m/z	Charge	Abund	Formula	Ion/Isotope	Tgt Mass Error (ppm)
242.12535	2	16177.19	C24H28F2N8O	(M+2H)+2	
242.62664	2	4814.55	C24H28F2N8O	(M+2H)+2	
243.12655	2	892.06	C24H28F2N8O	(M+2H)+2	
483.24303	1	242894.67	C24H28F2N8O	(M+H)+	
484.24583	1	62366.9	C24H28F2N8O	(M+H)+	
485.24787	1	8281.58	C24H28F2N8O	(M+H)+	
242.12535	2	16177.19	C24H28F2N8O	(M+2H)+2	1.5
242.62664	2	4814.55	C24H28F2N8O	(M+2H)+2	1.15
243.12655	2	892.06	C24H28F2N8O	(M+2H)+2	-4.71
483.24303	1	242894.67	C24H28F2N8O	(M+H)+	0.7
483.24303		242894.67			
484.24583	1	62366.9	C24H28F2N8O	(M+H)+	0.79
485.24787	1	8281.58	C24H28F2N8O	(M+H)+	-0.51

Compound 38

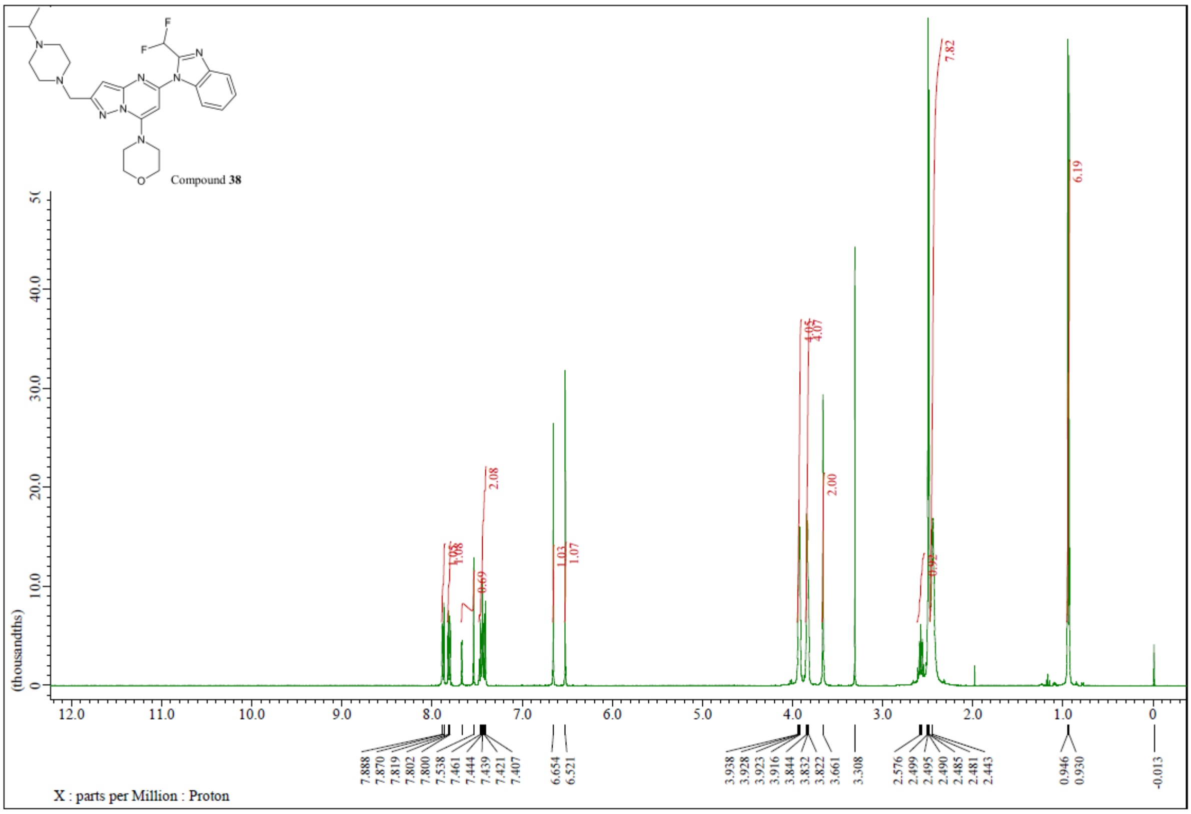
Compound 38
X : parts per Million : Proton
(thousands)

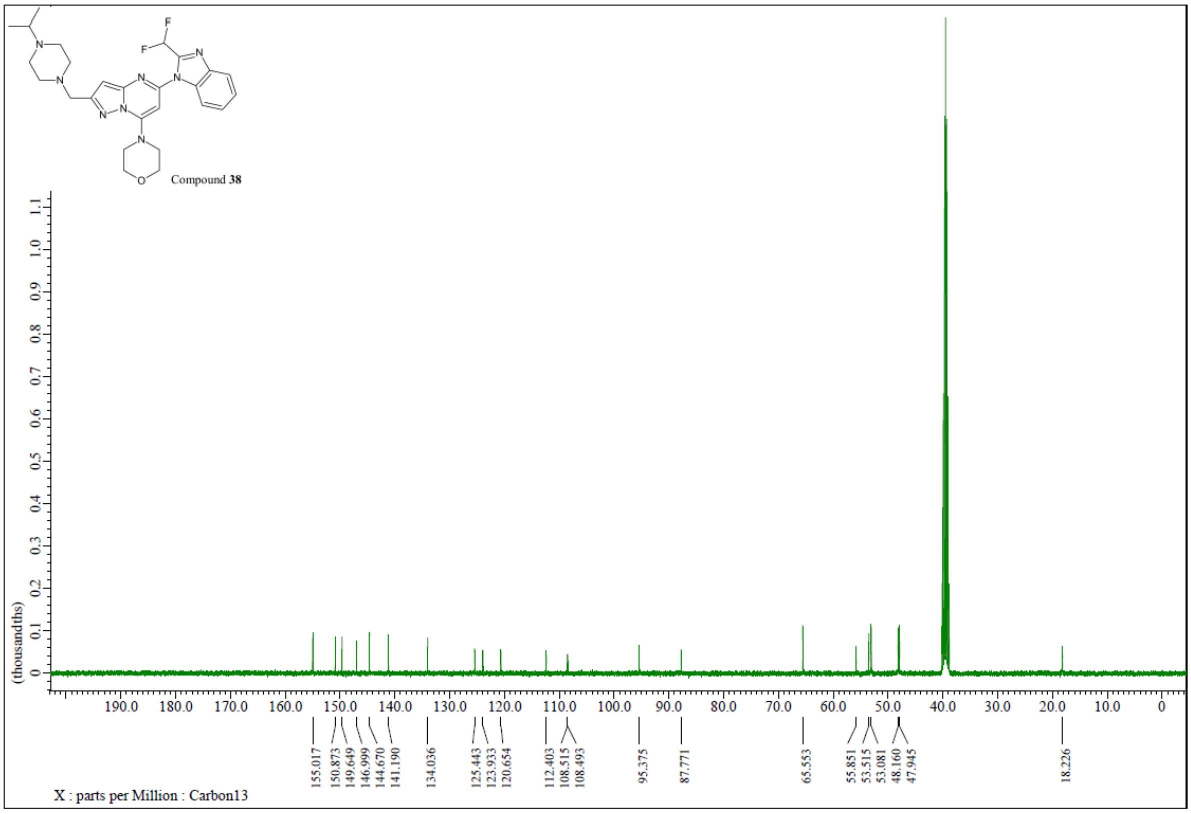
Compound 38
X : parts per Million : Carbon13
(thousands)

Compound 38

Name	*Obs. m/z*	Obs. RT	Obs. Mass	Tgt Formula	Tgt Mass	Tgt Mass Error	Find Cpds Algorith
Compound **38**	511.2743	1.91	510.26697	C26 H32 F2 N8 O	510.26671	0.51	Find by Formula

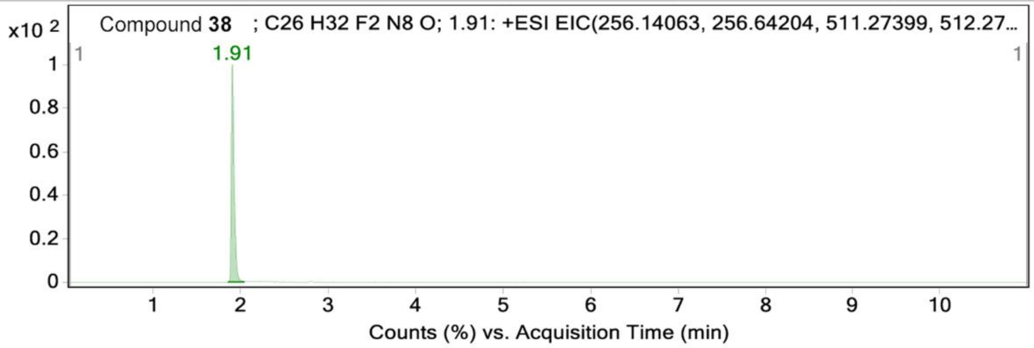

MS Zoomed Spectrum

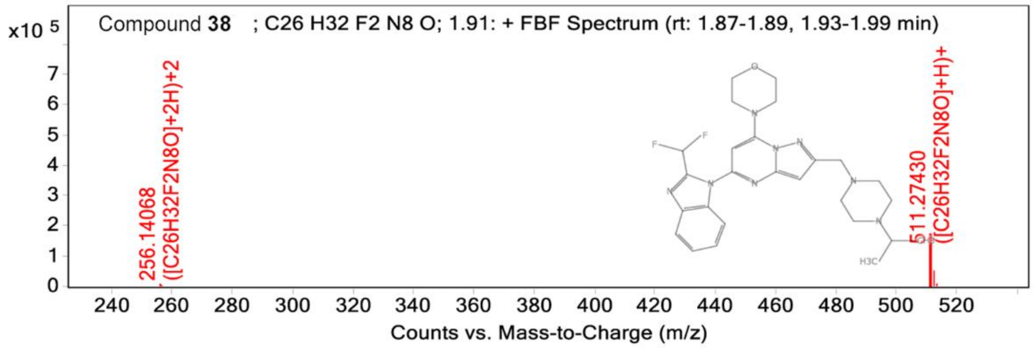

MS Zoomed Spectrum

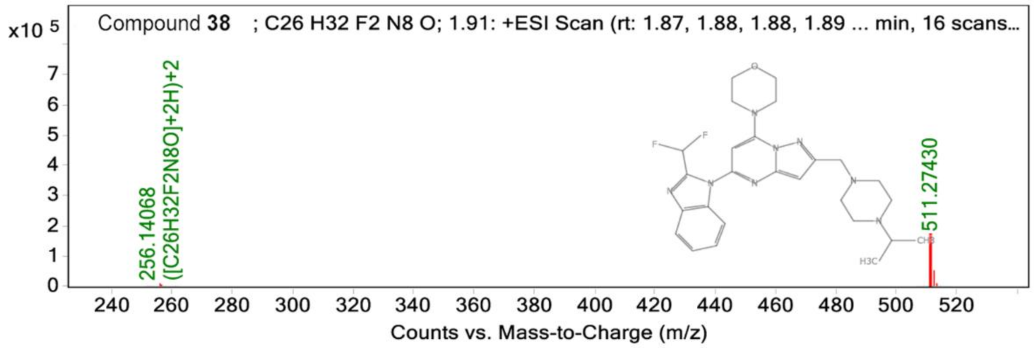

MS Spectrum Peak List

Obs. m/z	Charge	Abund	Formula	Ion/Isotope	Tgt Mass Error (ppm)
256.14068	2	7563.88	C26H32F2N8O	(M+2H)+2	
256.64215	2	2152.23	C26H32F2N8O	(M+2H)+2	
257.14261	2	324.73	C26H32F2N8O	(M+2H)+2	
511.2743	1	170137	C26H32F2N8O	(M+H)+	
512.27694	1	48697.73	C26H32F2N8O	(M+H)+	
513.27954	1	7568.92	C26H32F2N8O	(M+H)+	
256.14068	2	7563.88	C26H32F2N8O	(M+2H)+2	0.17
256.64215	2	2152.23	C26H32F2N8O	(M+2H)+2	0.44
257.14261	2	324.73	C26H32F2N8O	(M+2H)+2	-3.07
511.2743		170137			0.61
511.2743	1	170137	C26H32F2N8O	(M+H)+	0.61
512.27694	1	48697.73	C26H32F2N8O	(M+H)+	0.28
513.27954	1	7568.92	C26H32F2N8O	(M+H)+	0.03

--- End Of Report ---

Compound 40

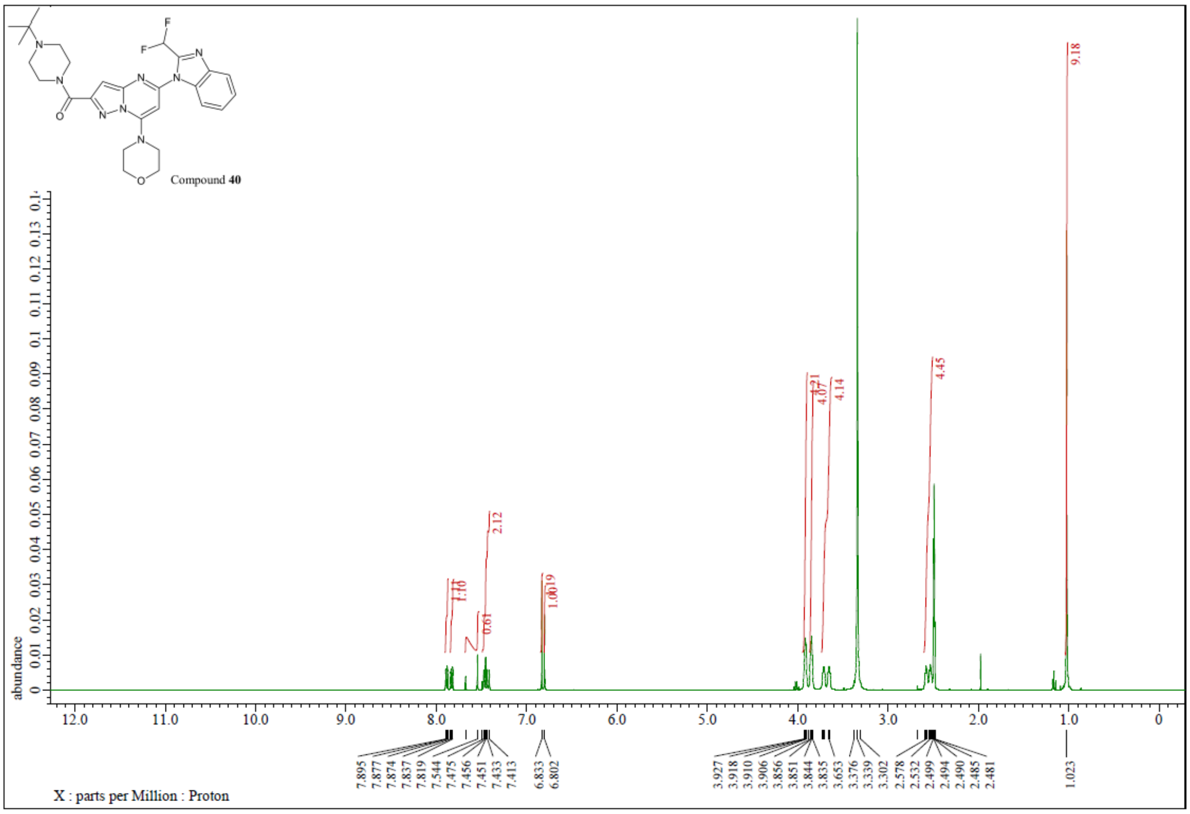

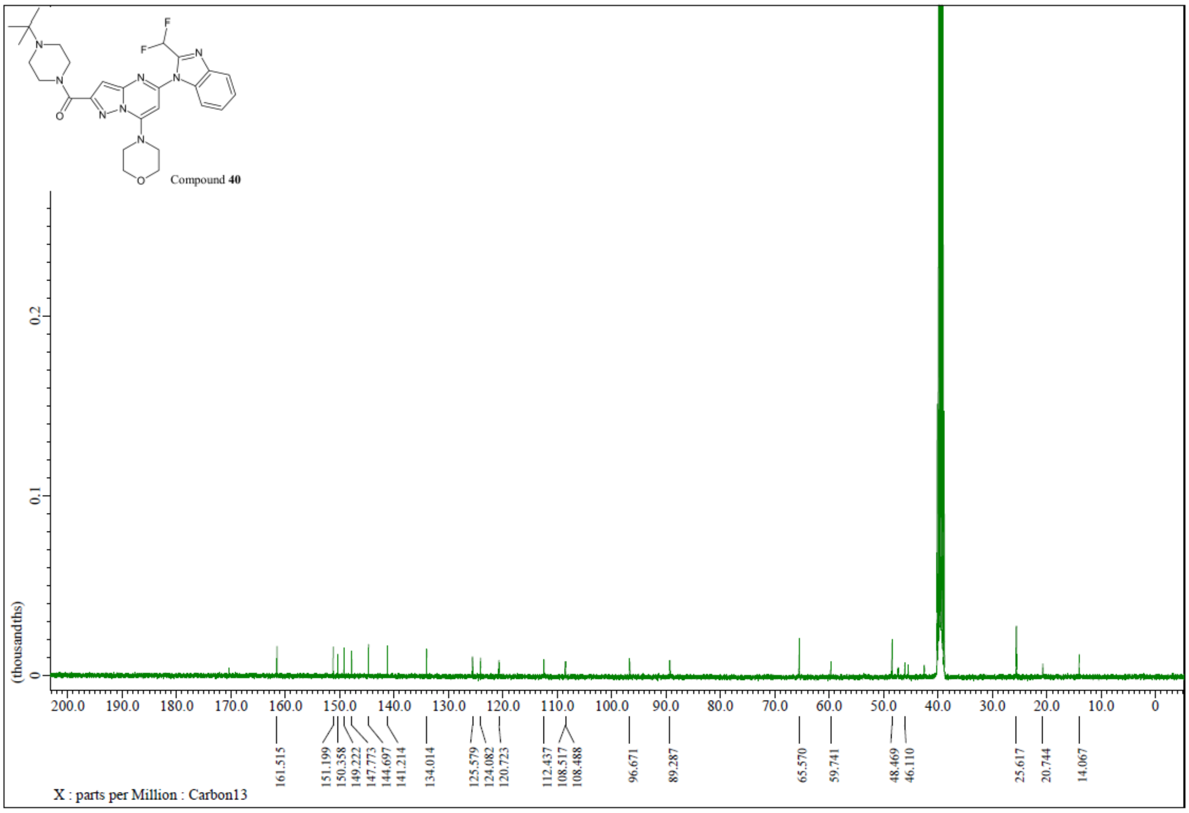

Compound 40

Name	*Obs. m/z*	Obs. RT	Obs. Mass	Tgt Formula	Tgt Mass	Tgt Mass Error	Find Cpds Algorit
Compound **40**	539.26912	1.31	538.26195	C27 H32 F2 N8 O2	538.26163	0.6	Find by Formula

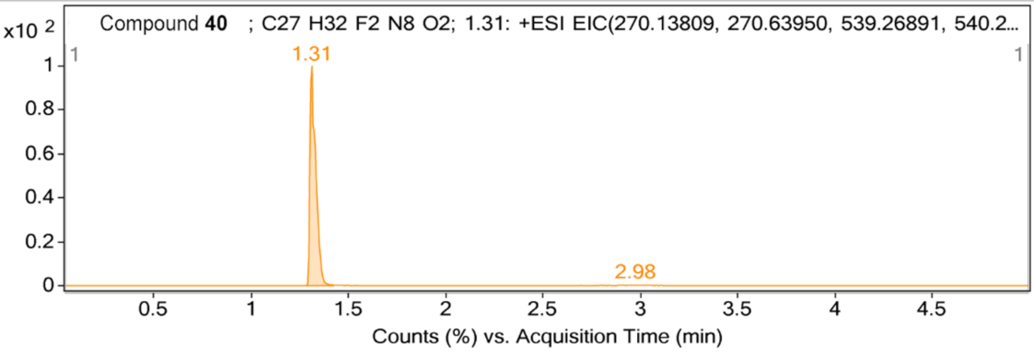

MS Zoomed Spectrum

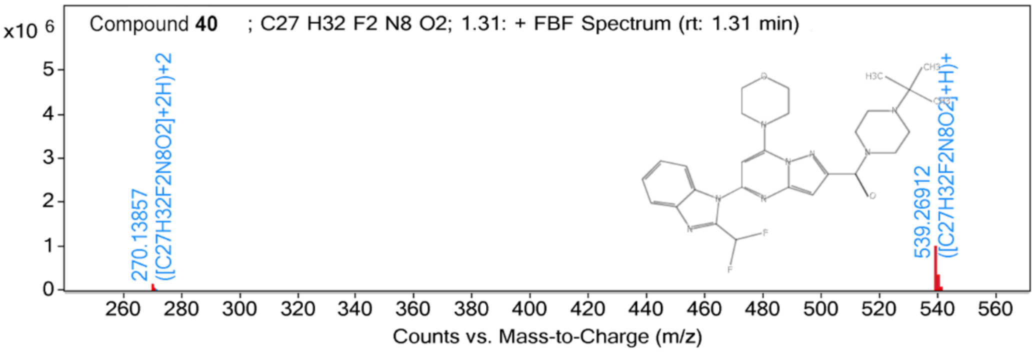

MS Zoomed Spectrum

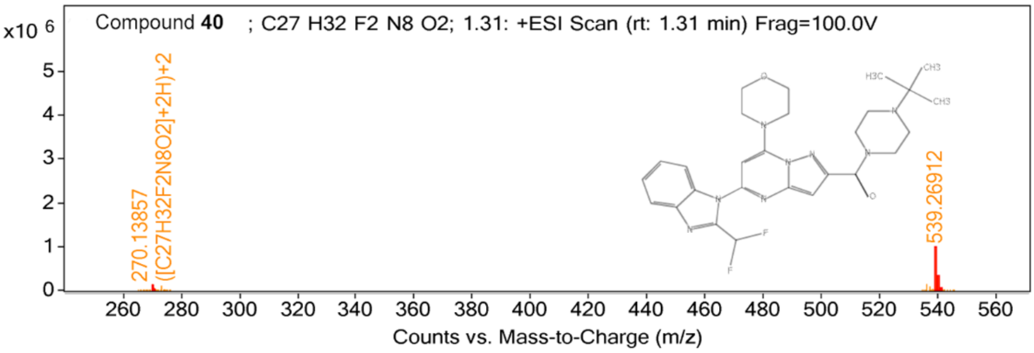

MS Spectrum Peak List

Obs. m/z	Charge	Abund	Formula	Ion/Isotope	Tgt Mass Error (ppm)
270.13857	2	125455.36	C27H32F2N8O2	(M+2H)+2	
270.63974	2	46195.77	C27H32F2N8O2	(M+2H)+2	
271.14284	2	8525.27	C27H32F2N8O2	(M+2H)+2	
271.64284	2	714.39	C27H32F2N8O2	(M+2H)+2	
539.26912	1	989648.63	C27H32F2N8O2	(M+H)+	
540.27212	1	296316.03	C27H32F2N8O2	(M+H)+	
541.27406	1	46140.58	C27H32F2N8O2	(M+H)+	
270.13857	2	125455.36	C27H32F2N8O2	(M+2H)+2	1.76
270.63974	2	46195.77	C27H32F2N8O2	(M+2H)+2	0.86
271.14284	2	8525.27	C27H32F2N8O2	(M+2H)+2	7.33
271.64284	2	714.39	C27H32F2N8O2	(M+2H)+2	2.5
539.26912	1	989648.63	C27H32F2N8O2	(M+H)+	0.39
539.26912		991037.31			
540.27212	1	296316.03	C27H32F2N8O2	(M+H)+	0.73
541.27406	1	46140.58	C27H32F2N8O2	(M+H)+	-0.68

--- End Of Report ---

Compound 41

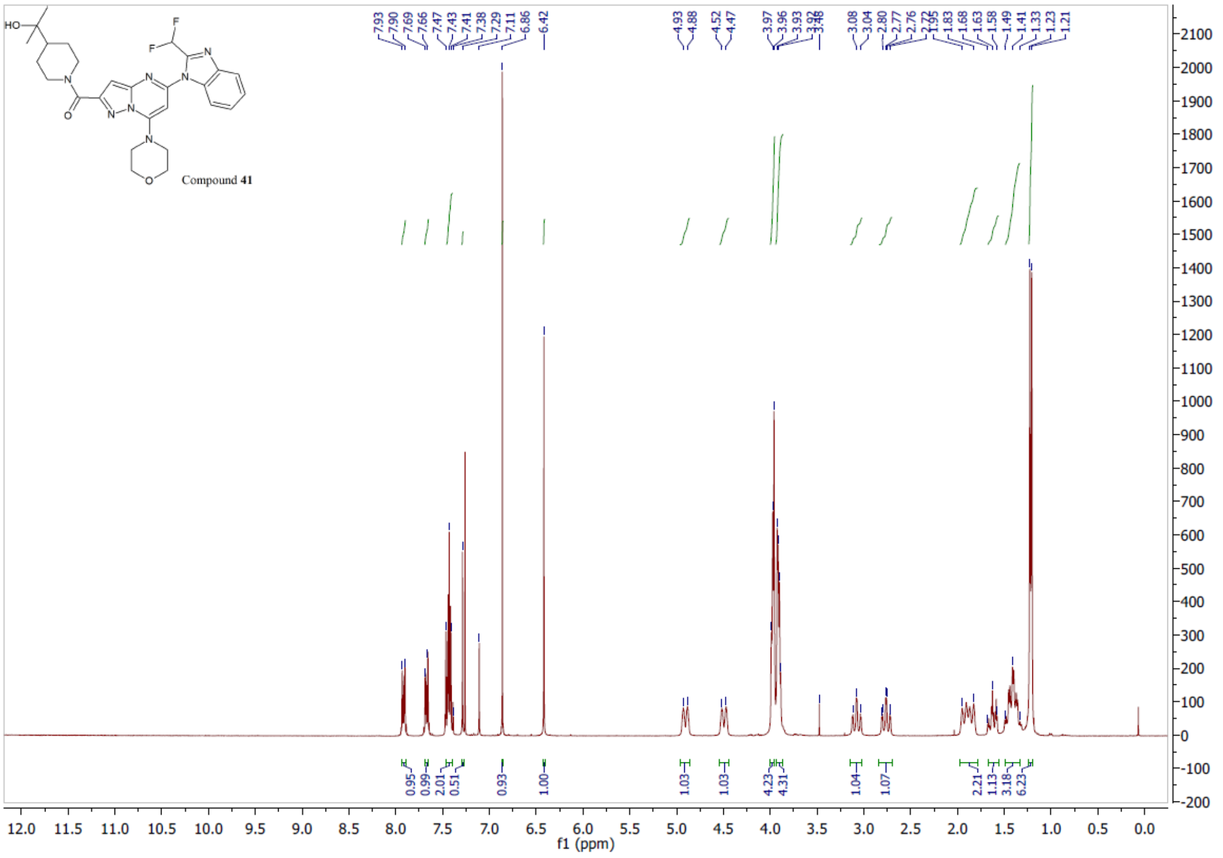

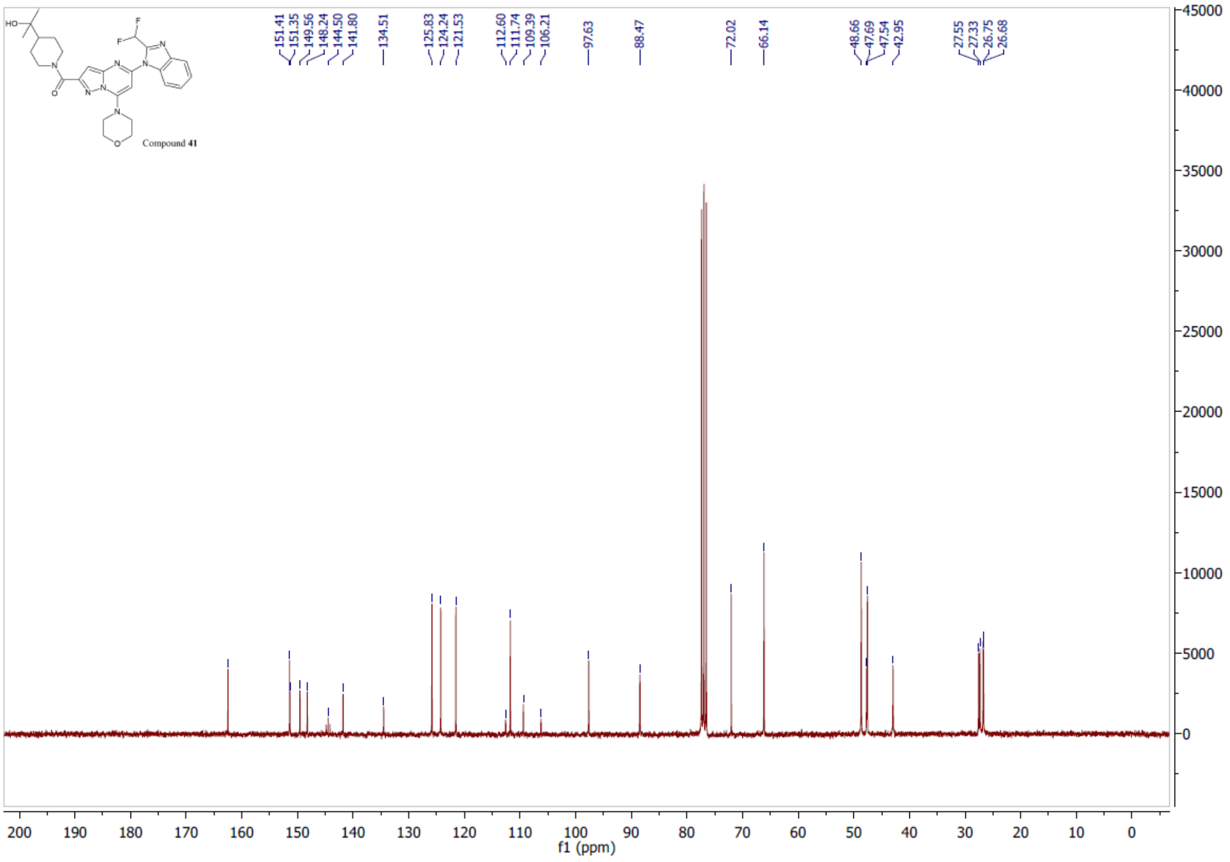

Compound 41

Compound Label	Name	*m/z*	RT	Algorithm	Mass
Compound **41**	Compound **41**	540.25488	2.18	Find by Formula	539.24756

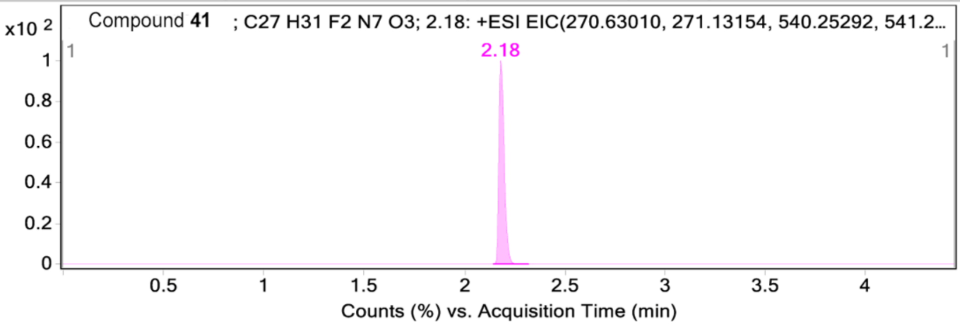

MS Zoomed Spectrum

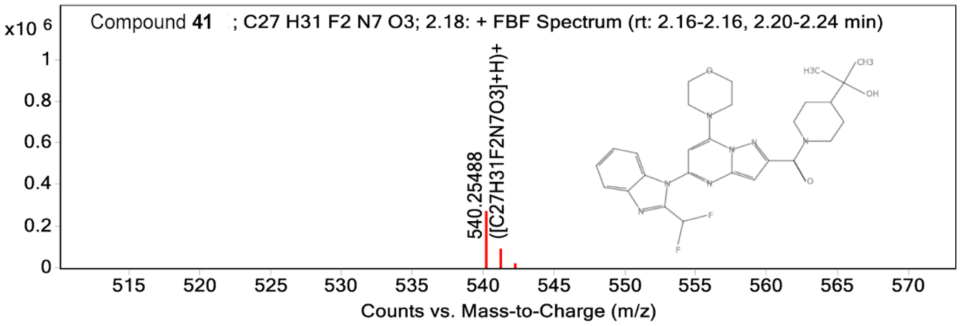

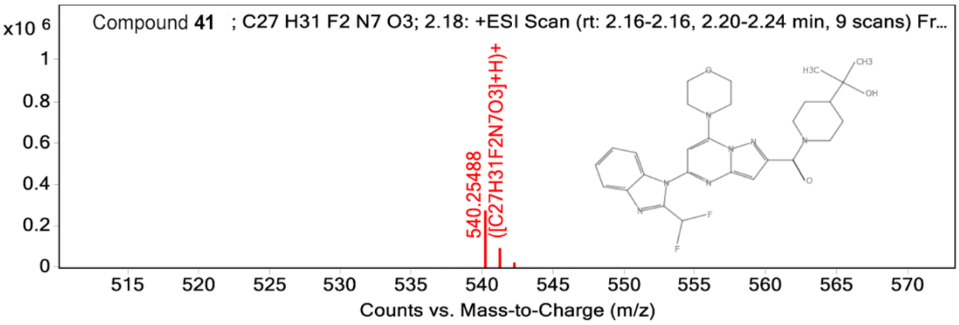

MS Spectrum Peak List

m/z	*Calc m/z*	Diff(ppm)	z	Abund	Formula	Ion
540.25488	540.25292	3.63	1	270345.84	C27H31F2N7O3	(M+H)+
541.25773	541.25581	3.55	1	82090.26	C27H31F2N7O3	(M+H)+
542.25958	542.25852	1.95	1	12735.37	C27H31F2N7O3	(M+H)+
543.26188	543.26112	1.4	1	1682.81	C27H31F2N7O3	(M+H)+

--- End Of Report ---

Compound 42

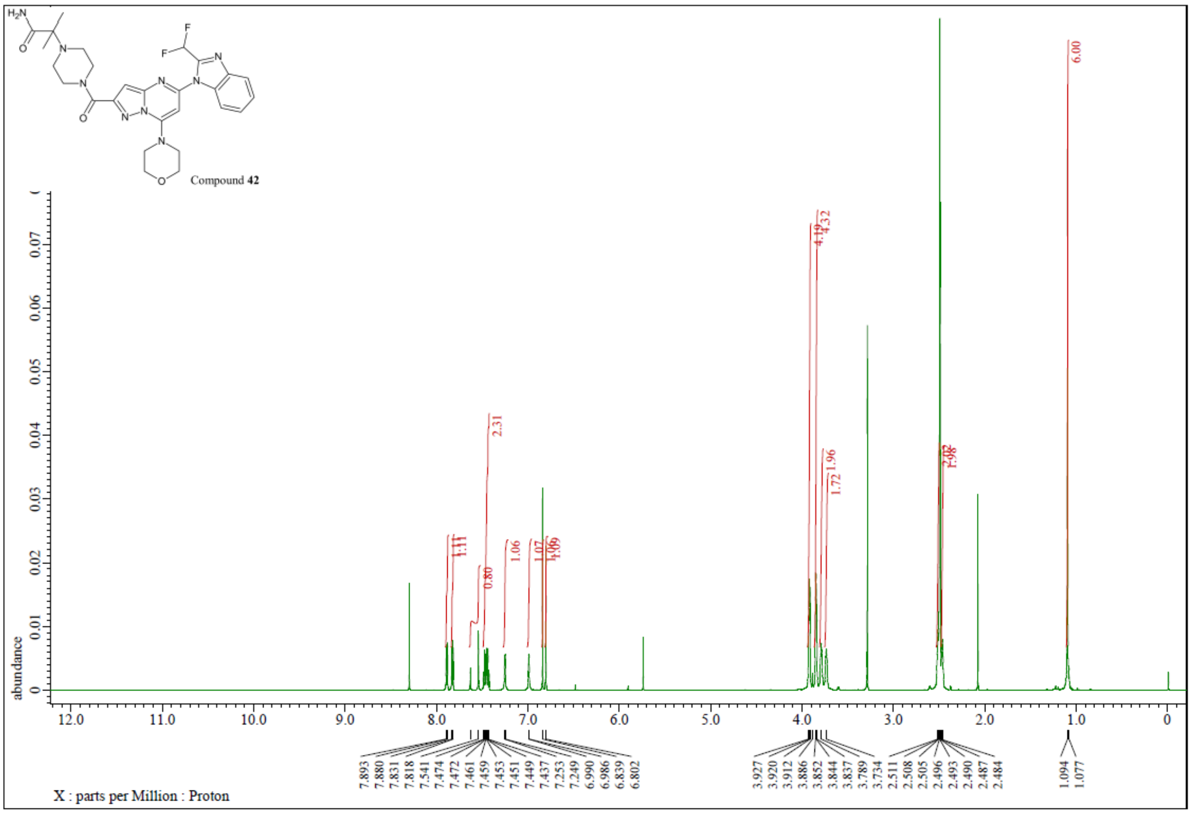

Compound 42
abundance
X : parts per Million : Proton

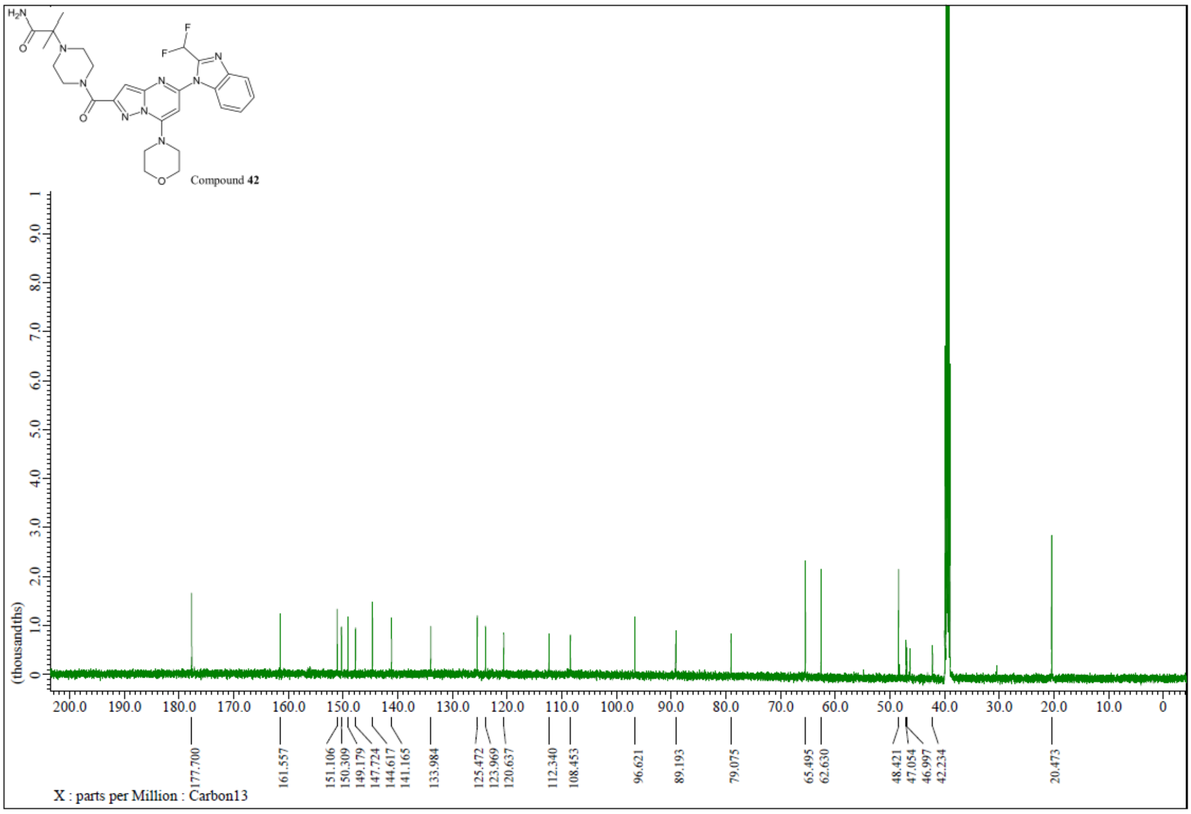

Compound 42
(thousandths)
X : parts per Million : Carbon13

Compound 42

Name	*Obs. m/z*	Obs. RT	Obs. Mass	Tgt Formula	Tgt Mass	Tgt Mass Error	Find Cpds Algorith
Compound **42**	568.25867	1.312	567.25125	C27 H31 F2 N9 O3	567.25179	-0.95	Find by Formula

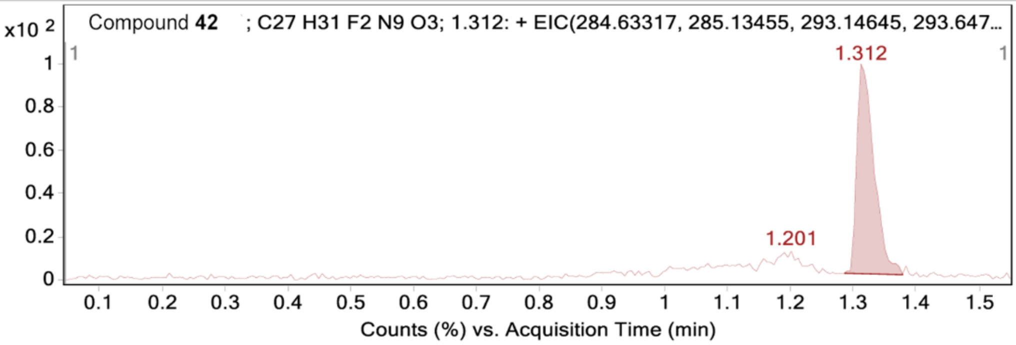

MS Zoomed Spectrum

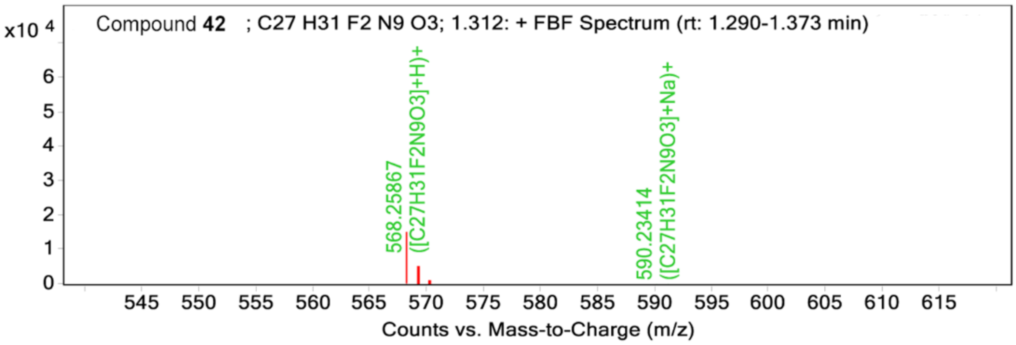

MS Zoomed Spectrum

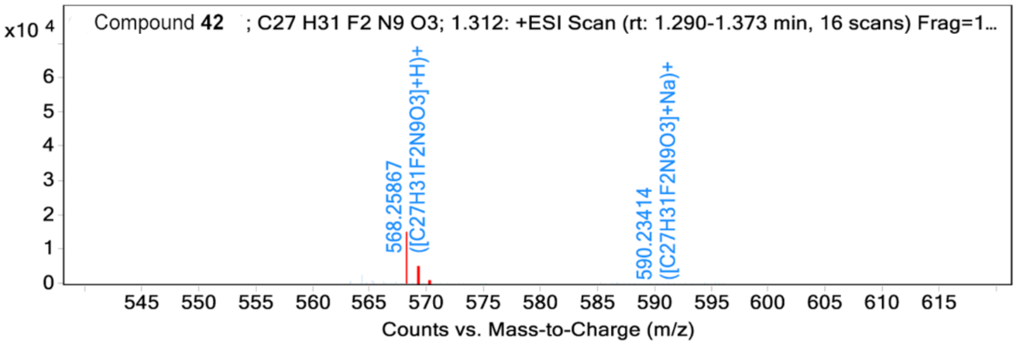

MS Spectrum Peak List

Obs. m/z	Charge	Abund	Formula	Ion/Isotope	Tgt Mass Error (ppm)
568.25867	1	14840.44	C27H31F2N9O3	(M+H)+	
569.26124	1	4302.48	C27H31F2N9O3	(M+H)+	
570.26212	1	858.69	C27H31F2N9O3	(M+H)+	
590.23414	1	59.04	C27H31F2N9O3	(M+Na)+	
591.24508	1	29.53	C27H31F2N9O3	(M+Na)+	
568.25867	1	14840.44	C27H31F2N9O3	(M+H)+	-0.7
569.26124	1	4302.48	C27H31F2N9O3	(M+H)+	-1.03
570.26212	1	858.69	C27H31F2N9O3	(M+H)+	-4.07
590.23414	1	59.04	C27H31F2N9O3	(M+Na)+	-11.65
591.24508	1	29.53	C27H31F2N9O3	(M+Na)+	2.21

--- End Of Report ---

Compound 43

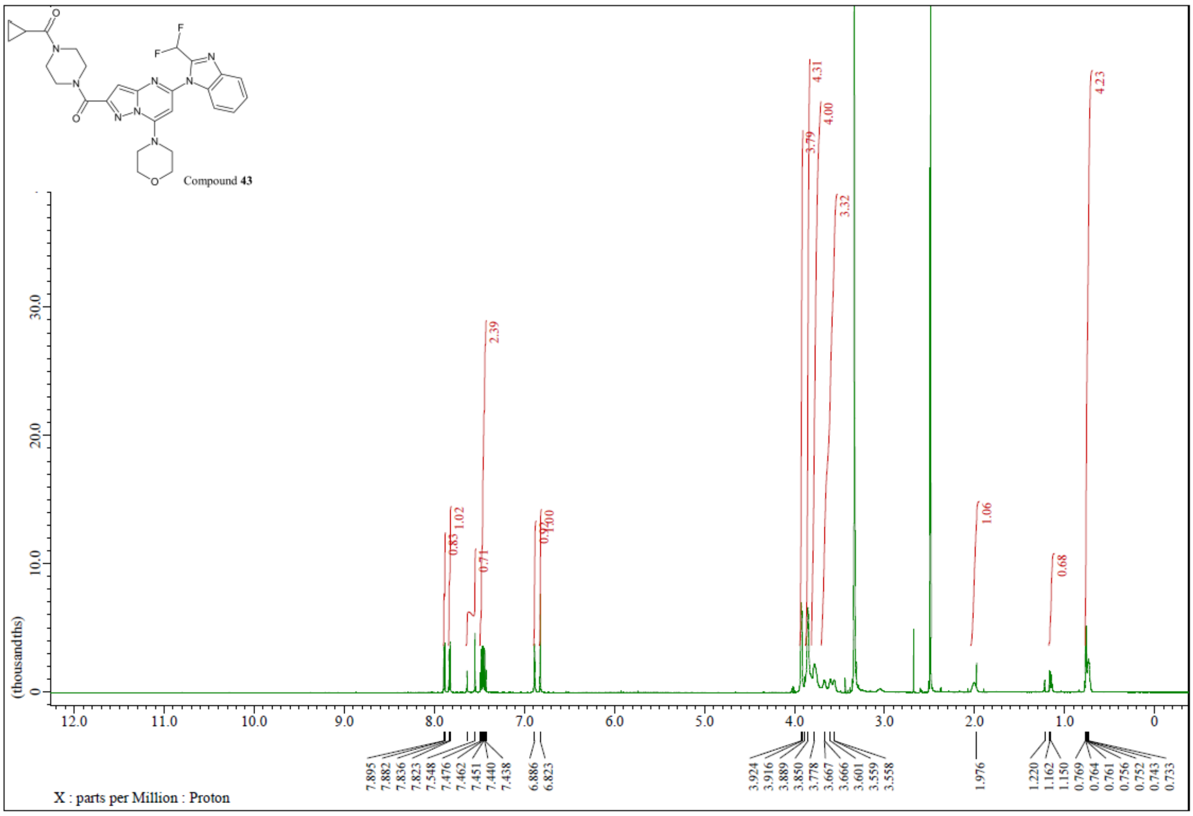
Compound 43
(thousandths)
X : parts per Million : Proton
7.895
7.882
7.836
7.823
7.548
7.476
7.462
7.451
7.440
7.438
6.886
6.823
3.924
3.916
3.889
3.850
3.778
3.667
3.666
3.601
3.559
3.558
1.976
1.220
1.162
1.150
0.769
0.764
0.761
0.756
0.752
0.743
0.733

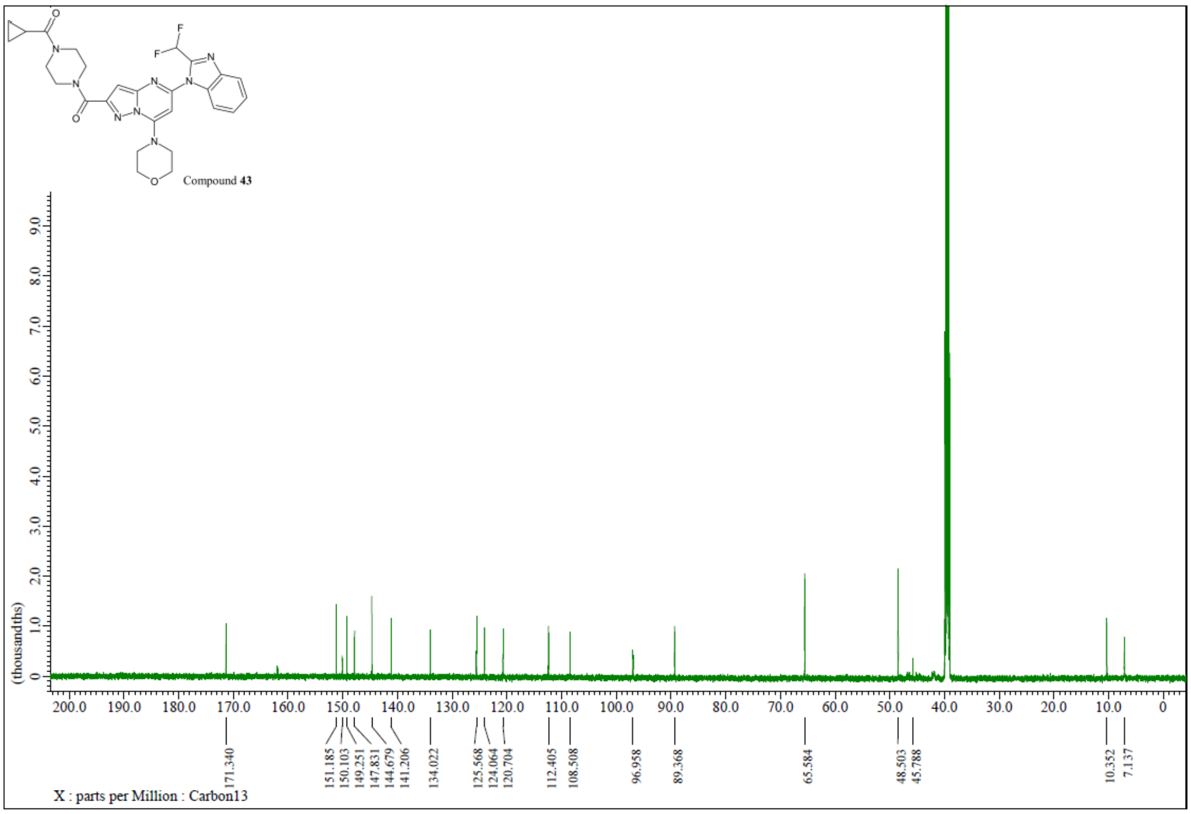
Compound 43
(thousandths)
X : parts per Million : Carbon13
171.340
151.185
150.103
149.251
147.831
144.679
141.206
134.022
125.568
124.064
120.704
112.405
108.508
96.958
89.368
65.584
48.503
45.788
10.352
7.137

Compound 43

Compound Label	Name	*m/z*	RT	Algorithm	Mass
Compound **43**	Compound **43**	276.1201	1.97	Find by Formula	550.22601

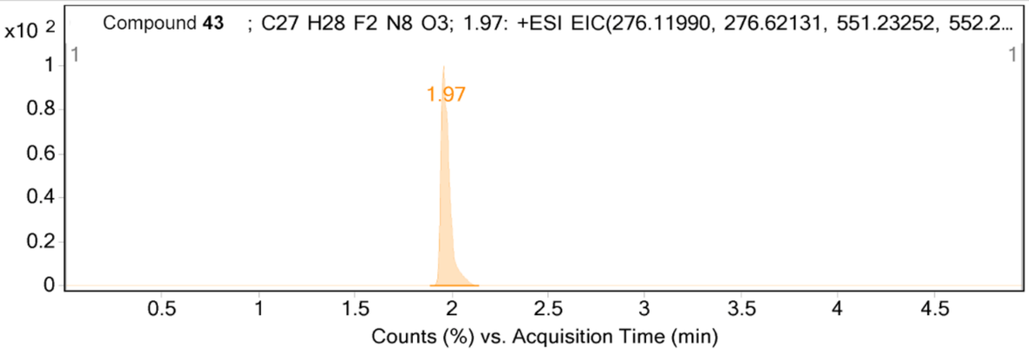

MS Zoomed Spectrum

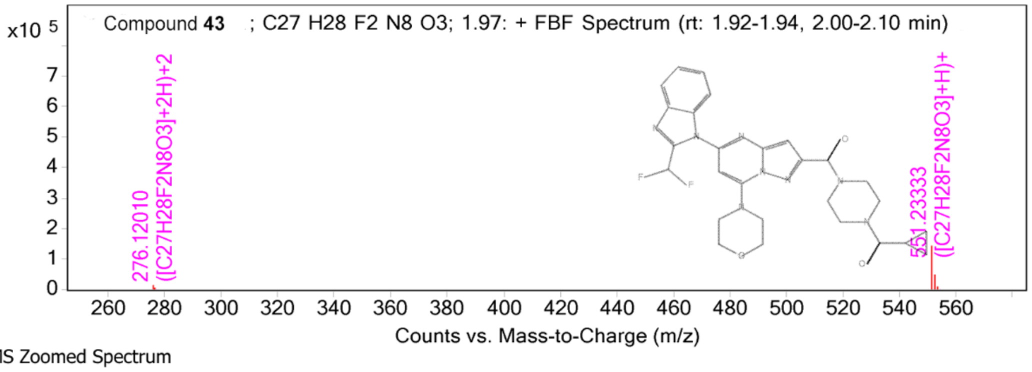

MS Zoomed Spectrum

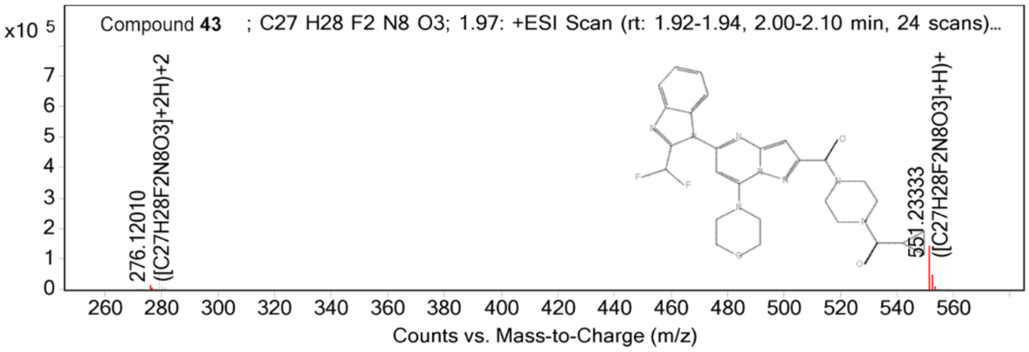

MS Spectrum Peak List

m/z	*Calc m/z*	Diff(ppm)	z	Abund	Formula	Ion
276.1201	276.1199	0.75	2	12256.77	C27H28F2N8O3	(M+2H)+2
276.6216	276.62131	1.04	2	3979.86	C27H28F2N8O3	(M+2H)+2
277.12341	277.12264	2.79	2	963.82	C27H28F2N8O3	(M+2H)+2
277.62135	277.62391	-9.23	2	52.08	C27H28F2N8O3	(M+2H)+2
551.23333	551.23252	1.47	1	142347.36	C27H28F2N8O3	(M+H)+
552.23614	552.23534	1.45	1	44714.86	C27H28F2N8O3	(M+H)+
553.23847	553.23799	0.85	1	7254.93	C27H28F2N8O3	(M+H)+
554.24149	554.24055	1.71	1	890.85	C27H28F2N8O3	(M+H)+

--- End Of Report ---

Compound 44

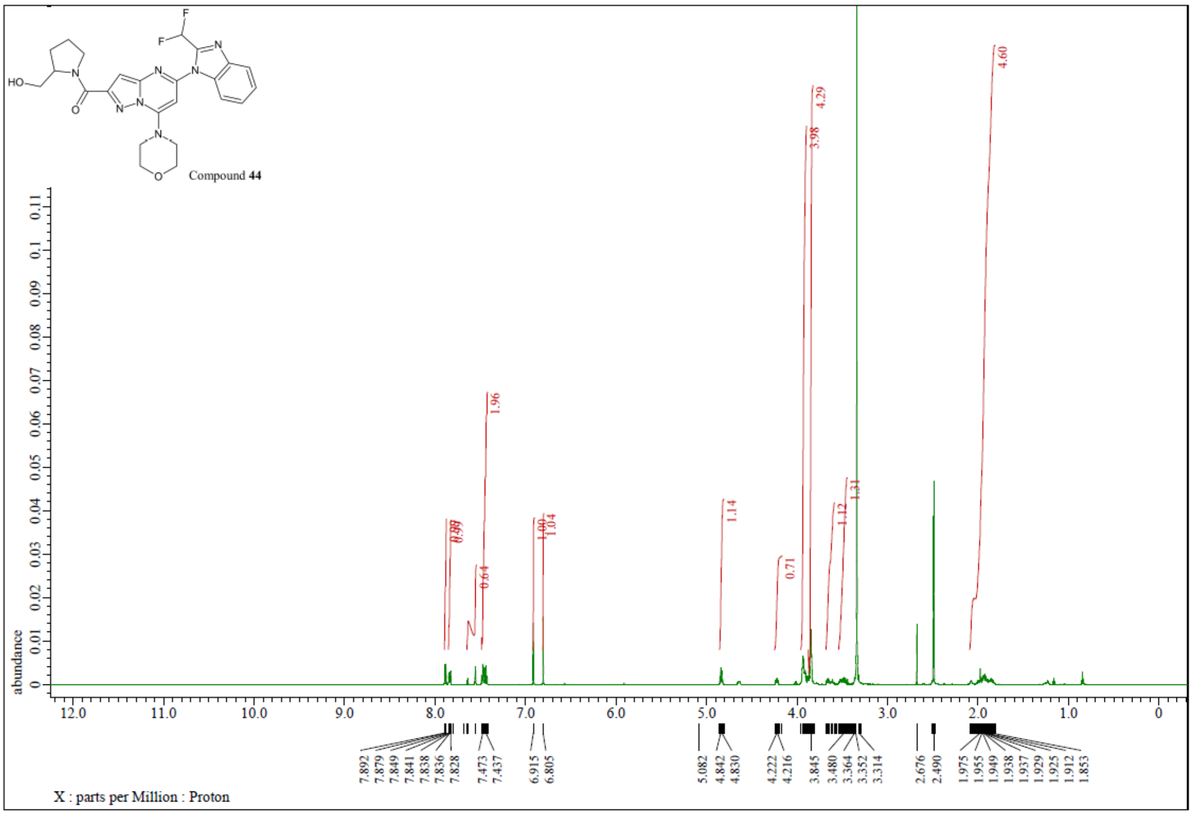

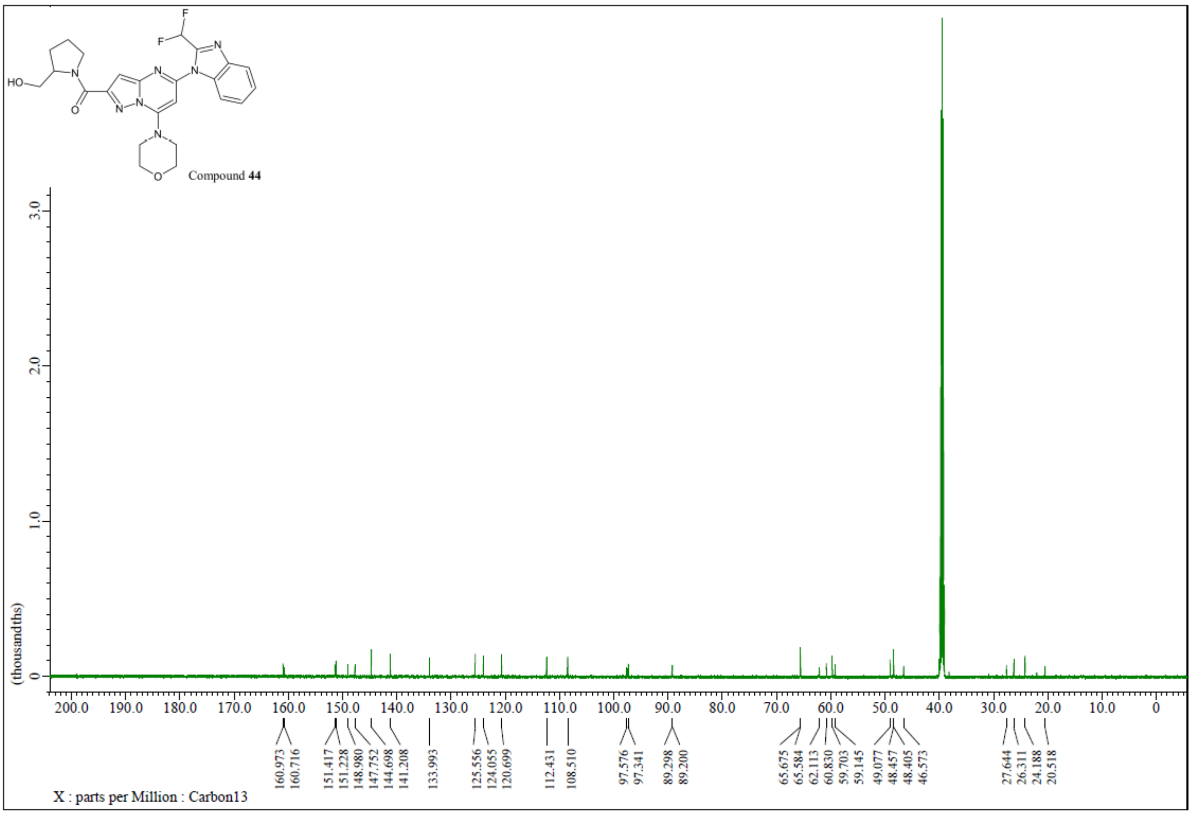

Compound 44

Compound Label	Name	*m/z*	RT	Algorithm	Mass
Compound **44**	Compound **44**	498.20662	1.32	Find by Formula	497.19931

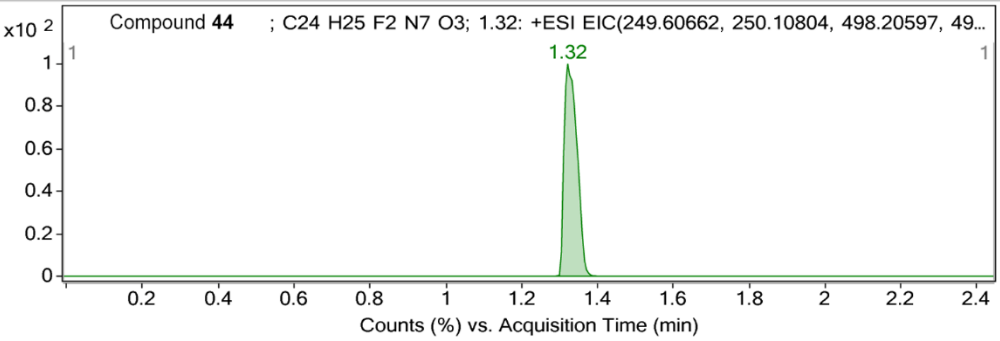

MS Zoomed Spectrum

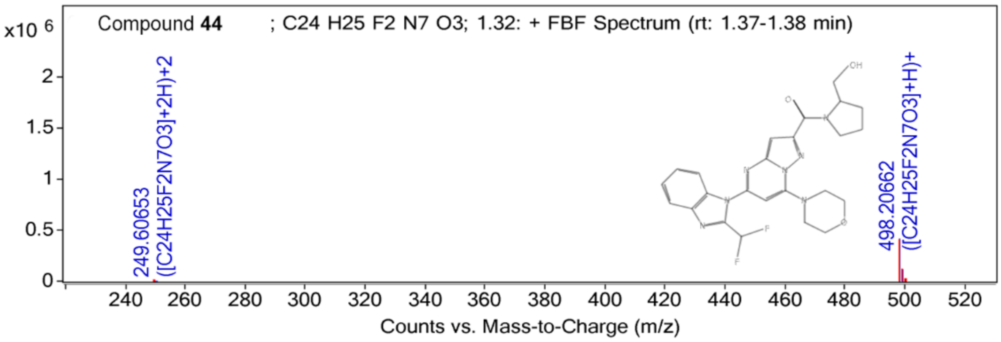

MS Zoomed Spectrum

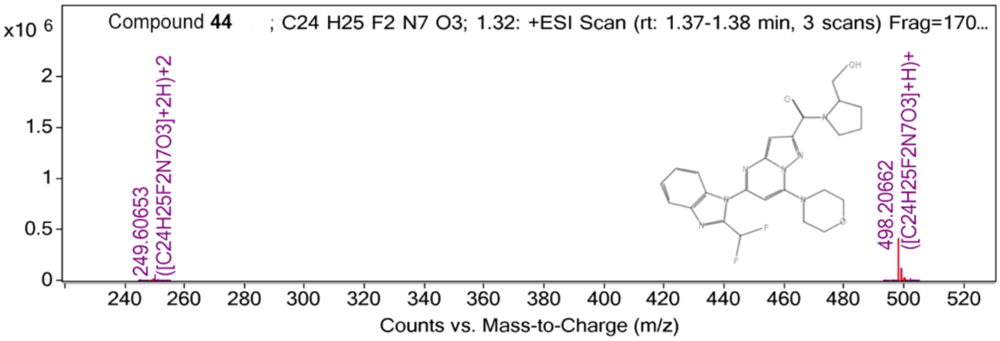

MS Spectrum Peak List

m/z	*Calc m/z*	Diff(ppm)	z	Abund	Formula	Ion
249.60653	249.60662	-0.37	2	7637.68	C24H25F2N7O3	(M+2H)+2
250.11133	250.10804	13.14	2	1938.11	C24H25F2N7O3	(M+2H)+2
250.60717	250.60935	-8.69	2	380.05	C24H25F2N7O3	(M+2H)+2
498.20662	498.20597	1.31	1	412028.53	C24H25F2N7O3	(M+H)+
499.20935	499.2088	1.09	1	111505.13	C24H25F2N7O3	(M+H)+
500.21156	500.21142	0.27	1	17153.88	C24H25F2N7O3	(M+H)+

--- End Of Report ---

Compound 45

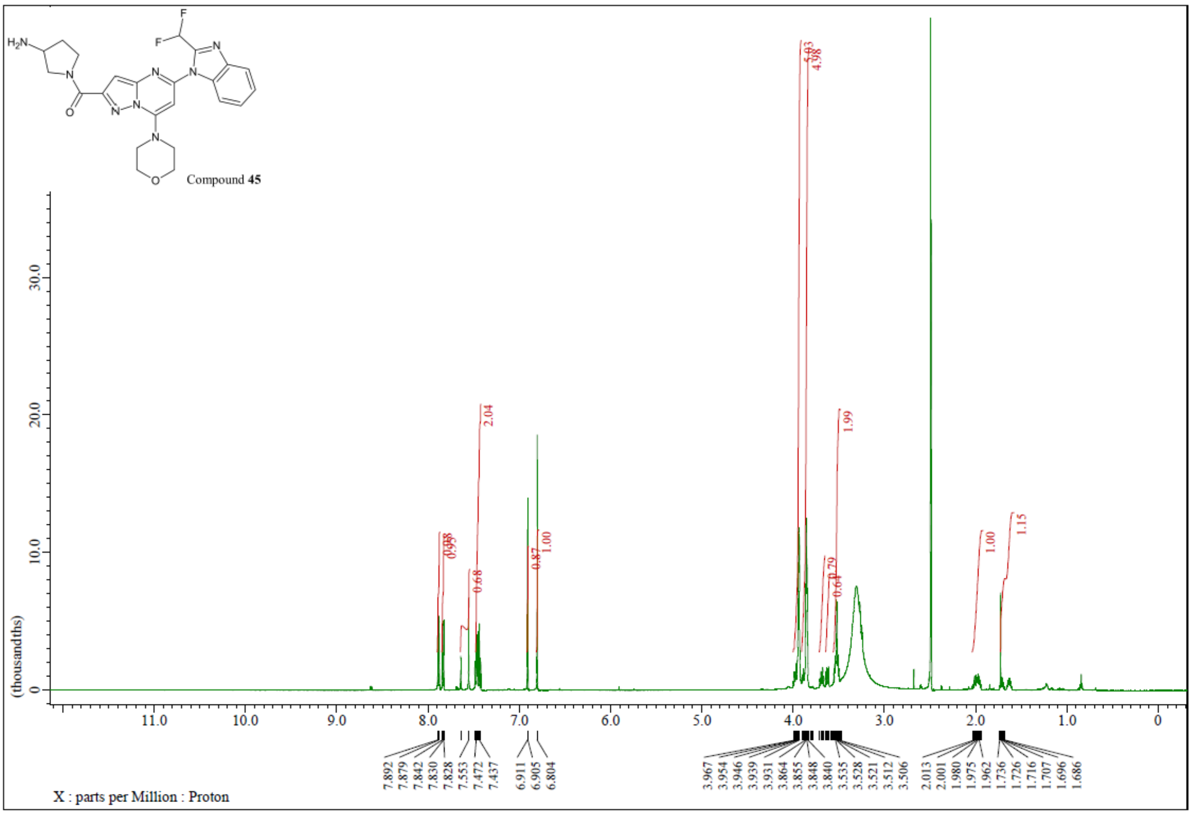
Compound 45
X : parts per Million : Proton
(thousandths)
7.892
7.879
7.842
7.830
7.828
7.553
7.472
7.437
6.911
6.905
6.804
3.967
3.954
3.946
3.939
3.931
3.864
3.855
3.848
3.840
3.535
3.528
3.521
3.512
3.506
2.013
2.001
1.980
1.975
1.962
1.736
1.726
1.716
1.707
1.696
1.686

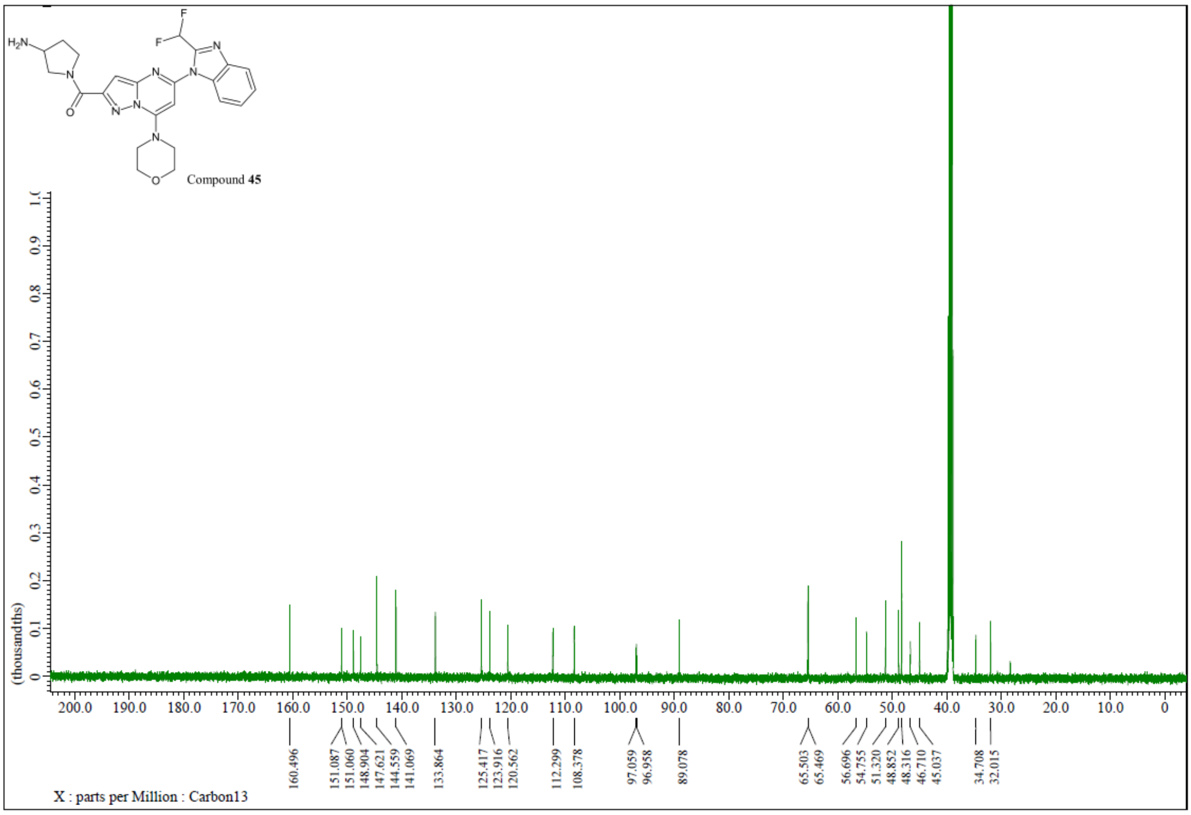
Compound 45
X : parts per Million : Carbon13
(thousandths)
160.496
151.087
151.060
148.904
147.621
144.559
141.069
133.864
125.417
123.916
120.562
112.299
108.378
97.059
96.958
89.078
65.503
65.469
56.696
54.755
51.320
48.852
48.316
46.710
45.037
34.708
32.015

Compound 45

Name	*Obs. m/z*	Obs. RT	Obs. Mass	Tgt Formula	Tgt Mass	Tgt Mass Error	Find Cpds Algorit
Compound **45**	505.1876	1.14	482.1996	C23 H24 F2 N8 O2	482.199	1.11	Find by Formula

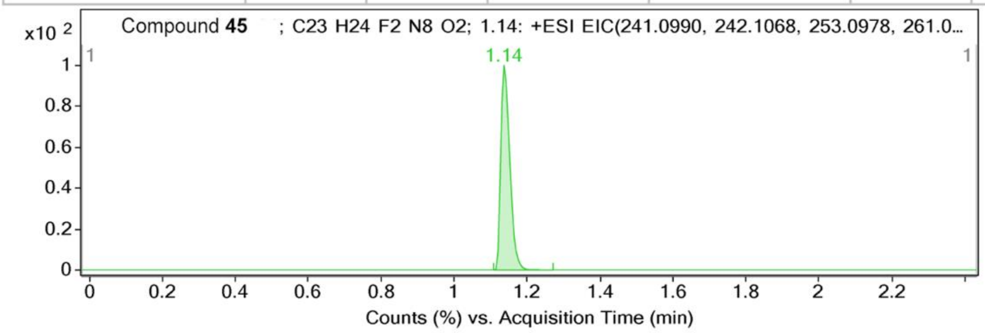

MS Zoomed Spectrum

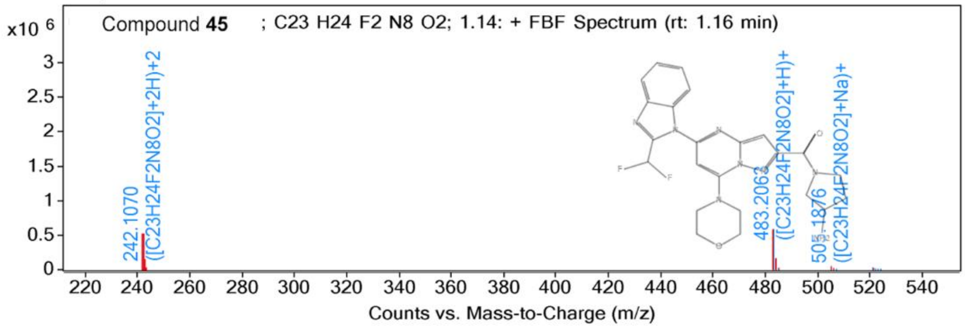

MS Zoomed Spectrum

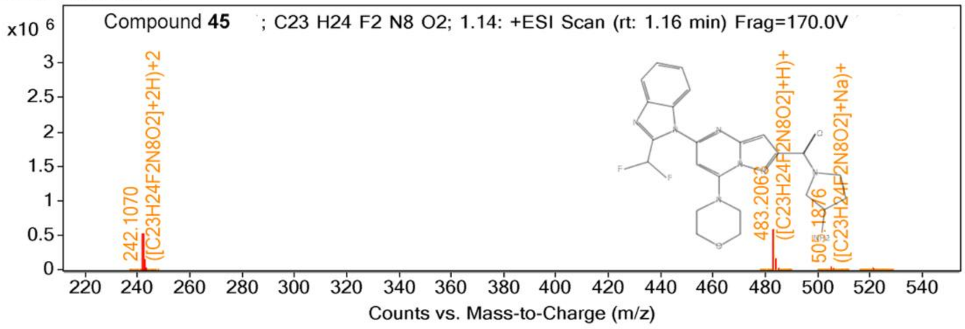

MS Spectrum Peak List

Obs. m/z	Charge	Abund	Formula	Ion/Isotope	Tgt Mass Error (ppm)
242.107	2	512912.66	C23H24F2N8O2	(M+2H)+2	
242.6087	2	129371.14	C23H24F2N8O2	(M+2H)+2	
243.1117	2	17811.2	C23H24F2N8O2	(M+2H)+2	
483.2063	1	585879.56	C23H24F2N8O2	(M+H)+	
484.2113	1	161792.5	C23H24F2N8O2	(M+H)+	
485.2155	1	23468.76	C23H24F2N8O2	(M+H)+	
505.1876	1	42200.06	C23H24F2N8O2	(M+Na)+	
506.1906	1	12088.44	C23H24F2N8O2	(M+Na)+	
521.1614	1	26443.12	C23H24F2N8O2	(M+K)+	
522.164	1	7111.54	C23H24F2N8O2	(M+K)+	
242.107	2	512912.66	C23H24F2N8O2	(M+2H)+2	0.88
242.6087	2	129371.14	C23H24F2N8O2	(M+2H)+2	2.24
243.1117	2	17811.2	C23H24F2N8O2	(M+2H)+2	9.44
483.2063	1	585879.56	C23H24F2N8O2	(M+H)+	-0.06
484.2113	1	161792.5	C23H24F2N8O2	(M+H)+	4.6
485.2155	1	23468.76	C23H24F2N8O2	(M+H)+	7.94
505.1876	1	42200.06	C23H24F2N8O2	(M+Na)+	-1.34
506.1906	1	12088.44	C23H24F2N8O2	(M+Na)+	-0.77
521.1614	1	26443.12	C23H24F2N8O2	(M+K)+	-1.6
522.164	1	7111.54	C23H24F2N8O2	(M+K)+	-1.83

--- End Of Report ---

Compound 46

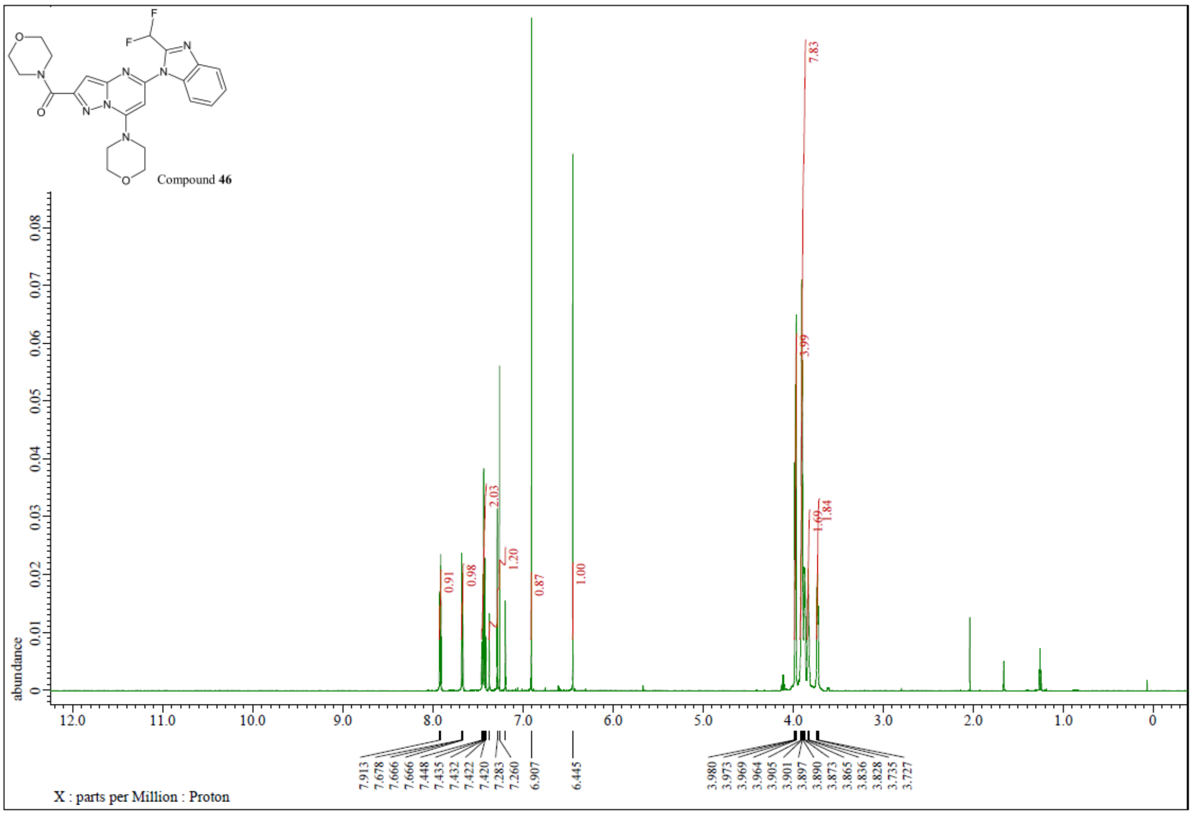
Compound 46
abundance
X : parts per Million : Proton
7.913 7.678 7.666 7.666 7.448 7.435 7.432 7.422 7.420 7.283 7.260 6.907 6.445
3.980 3.973 3.969 3.964 3.905 3.901 3.897 3.890 3.873 3.865 3.836 3.828 3.735 3.727
0.91 0.98 2.03 1.20 0.87 1.00 7.83 3.99 1.69 1.84

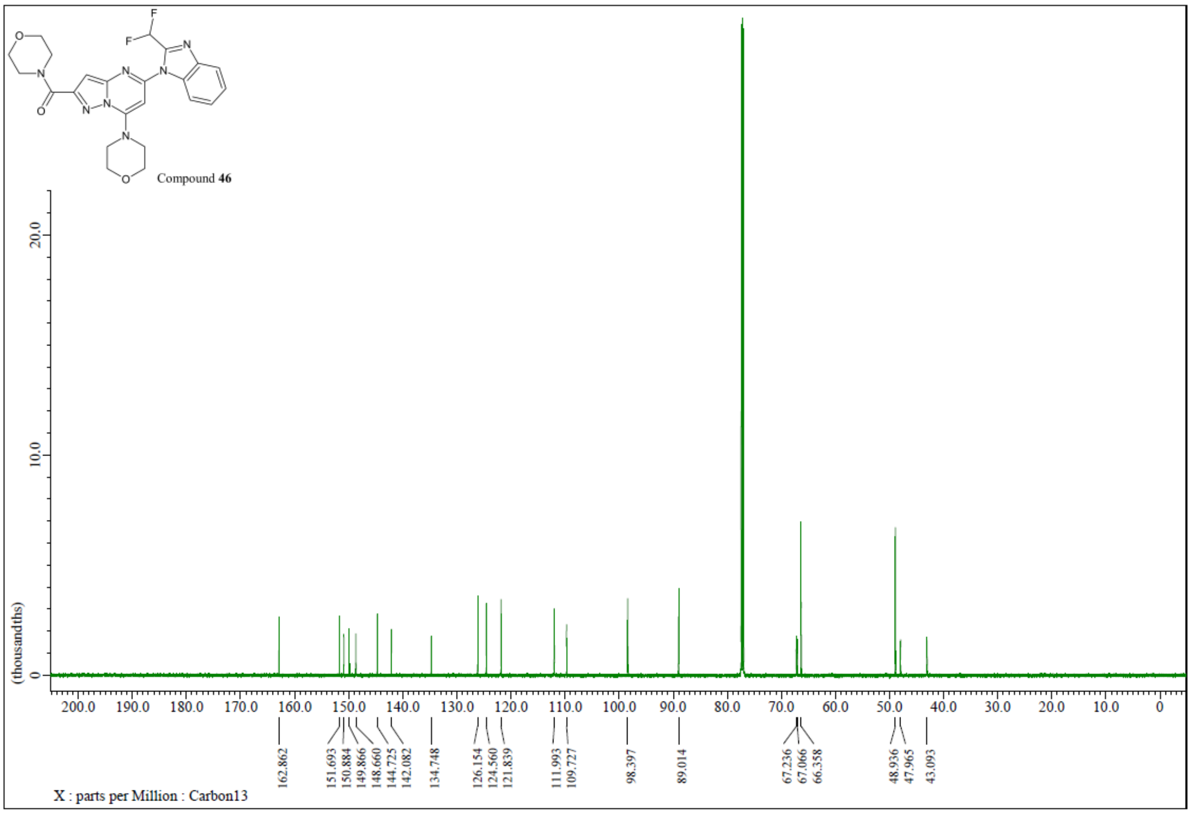
Compound 46
(thousandths)
X : parts per Million : Carbon13
162.862 151.693 150.884 149.866 148.660 144.725 142.082 134.748 126.154 124.560 121.839 111.993 109.727 98.397 89.014 67.236 67.066 66.358 48.936 47.965 43.093

Compound 46

Fragmentor Voltage 170 **Collision Energy** 0 **Ionization Mode** ESI

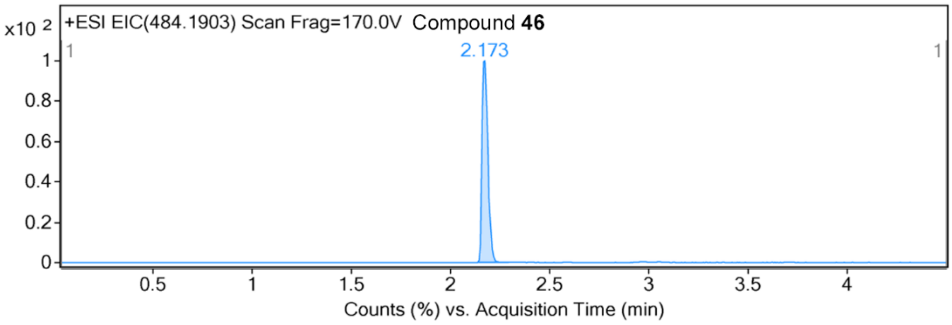

Integration Peak List

Peak	Start	RT	End	Height	Area	Area %
1	2.135	2.173	2.295	3035891.34	6209015.39	100

Spectra

Spectrum Source	Fragmentor Voltage	Collision Energy	Ionization Mode
Peak (1) in "+ EIC(484.1903) Scan"	170	0	ESI

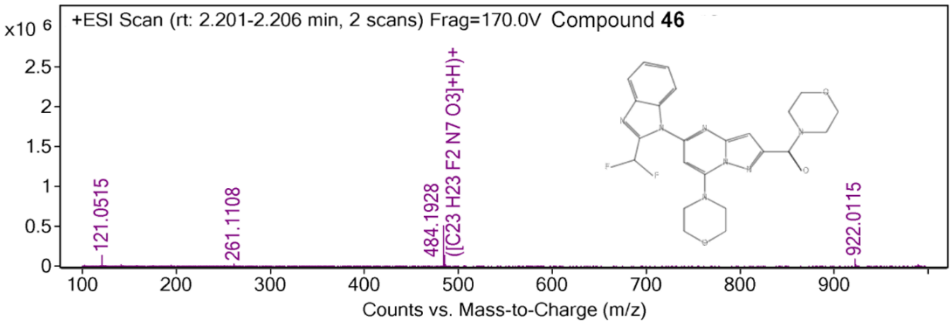

Peak List

m/z	z	Abund	Name	Formula	Ion	Score (DB)	Hits (DB)
484.1928	1	508306.75	Compound **46**	C23 H23 F2 N7 O3	(M+H)+	98.27	1

Spectrum Structure

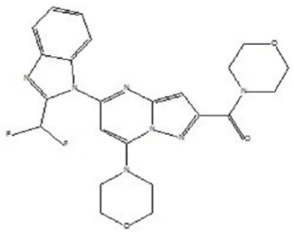

--- End Of Report ---

Compound 47

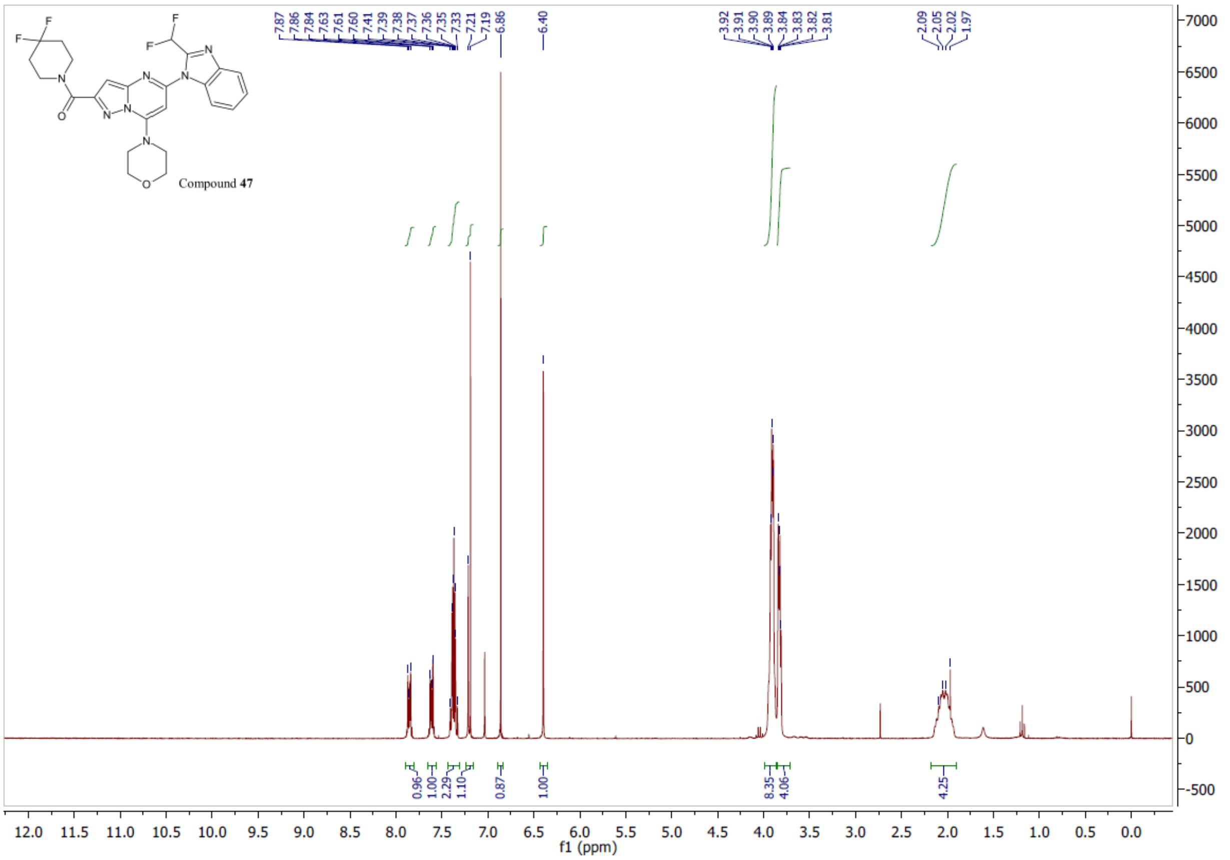
Compound 47
7.87 7.86 7.84 7.63 7.61 7.60 7.41 7.39 7.38 7.37 7.36 7.35 7.33 7.21 7.19 6.86 6.40
3.92 3.91 3.90 3.89 3.84 3.83 3.82 3.81
2.09 2.05 2.02 1.97
0.96 1.00 2.29 1.10 0.87 1.00 8.35 4.06 4.25
f1 (ppm)

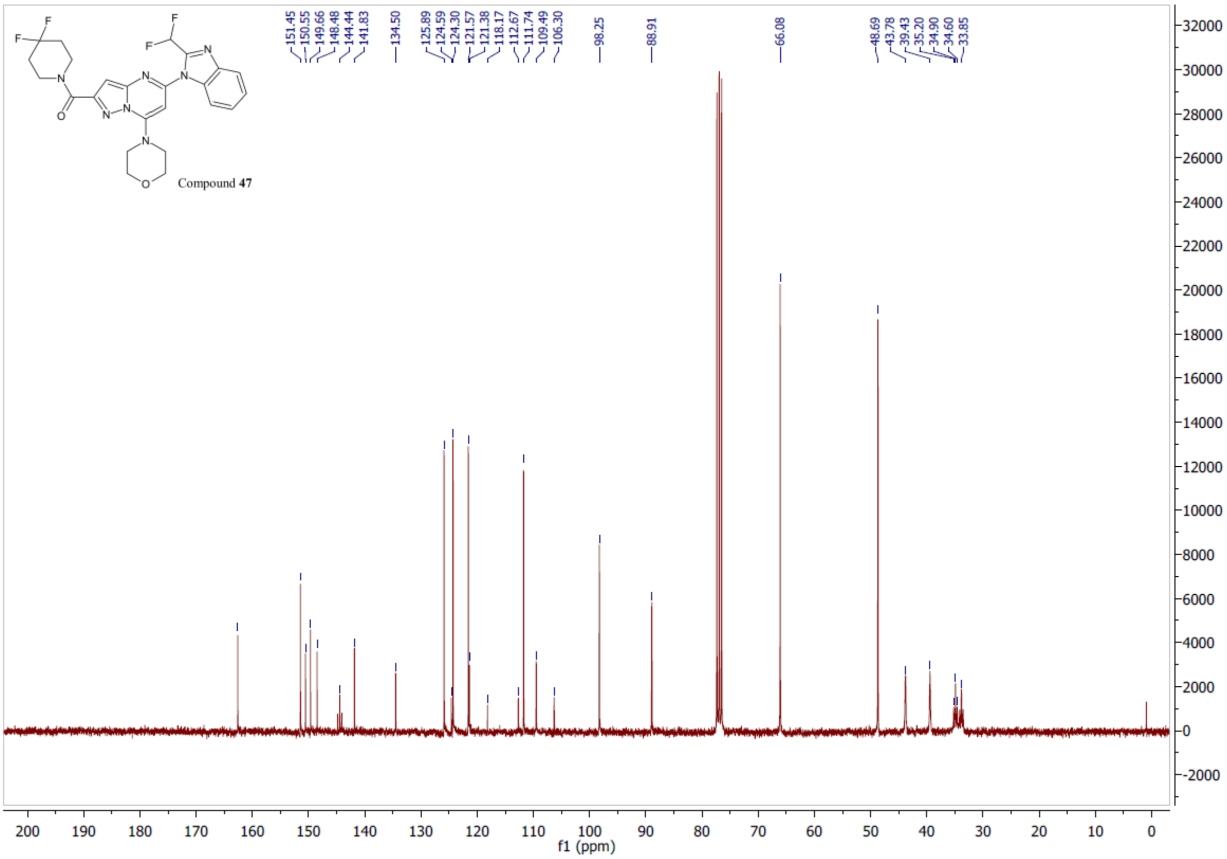
Compound 47
151.45 150.55 149.66 148.48 144.44 141.83 134.50 125.89 124.59 124.30 121.57 121.38 118.17 112.67 111.74 109.49 106.30 98.25 88.91 66.08 48.69 43.78 39.43 35.20 34.90 34.60 33.85
f1 (ppm)

Compound 47

Name	*Obs. m/z*	Obs. RT	Obs. Mass	Tgt Formula	Tgt Mass	Tgt Mass Error	Find Cpds Algorit
Compound **47**	540.1761	1.39	517.187	C24 H23 F4 N7 O2	517.1849	4.06	Find by Formula

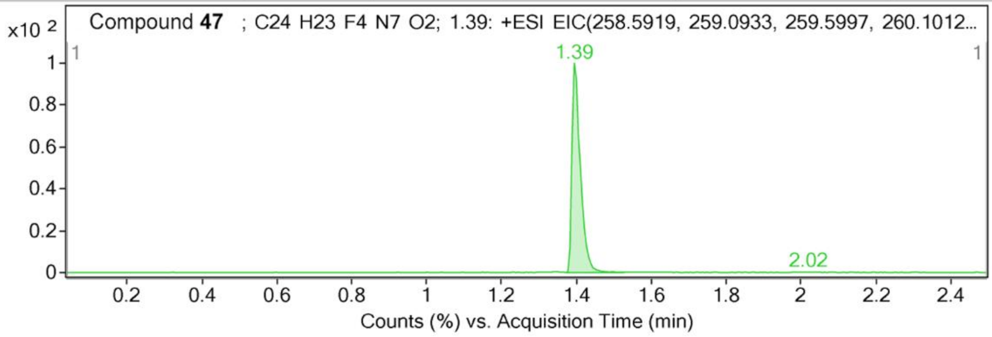

MS Zoomed Spectrum

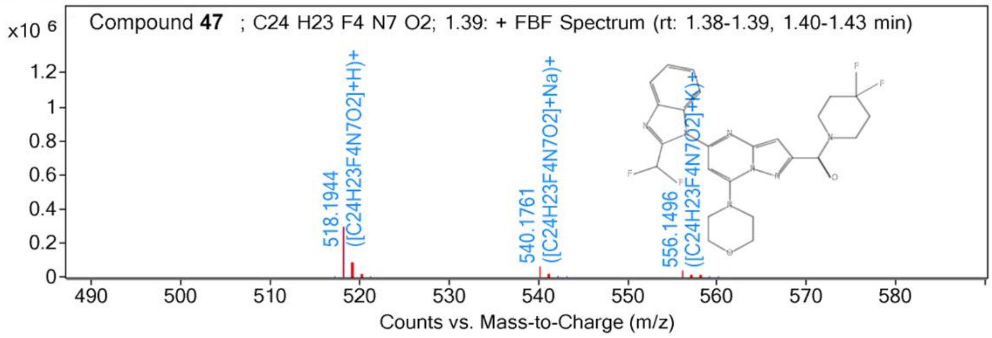

MS Zoomed Spectrum

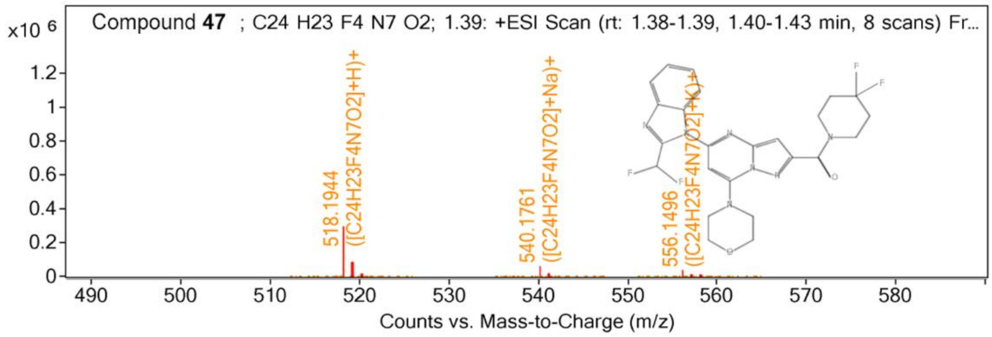

MS Spectrum Peak List

Obs. m/z	Charge	Abund	Formula	Ion/Isotope	Tgt Mass Error (ppm)
518.1944	1	291918.13	C24H23F4N7O2	(M+H)+	
518.1944	1	291918.13	C24H23F4N7O2	(M+H)+	4.27

--- End Of Report ---

Compound 48

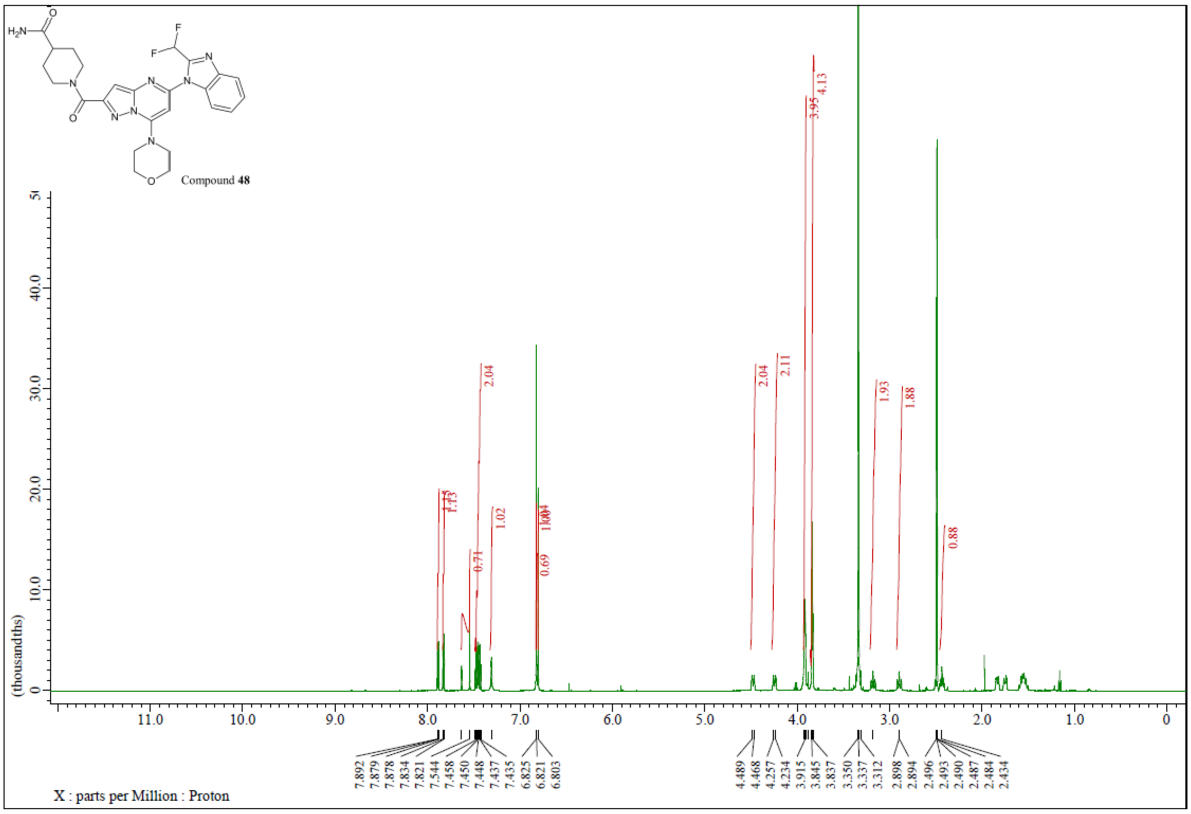

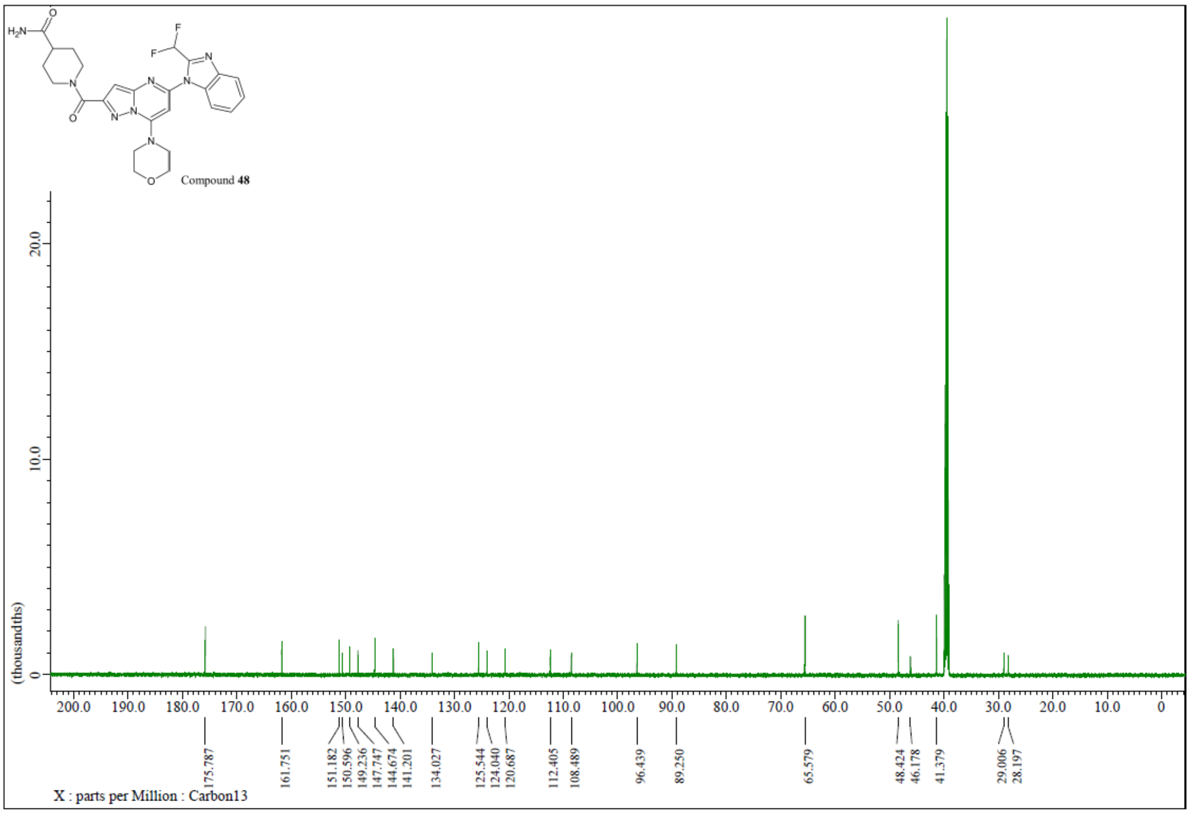

Compound 48

Name	*Obs. m/z*	Obs. RT	Obs. Mass	Tgt Formula	Tgt Mass	Tgt Mass Error	Find Cpds Algorit
Compound **48**	547.1994	1.921	524.2111	C25 H26 F2 N8 O3	524.2096	2.9	Find by Formula

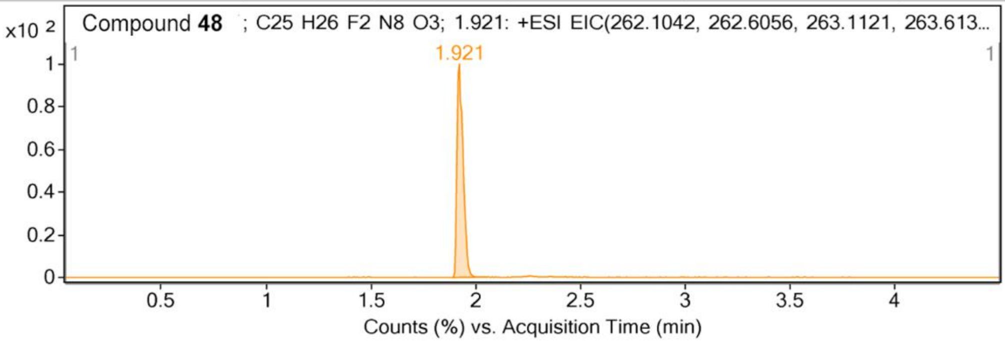

MS Zoomed Spectrum

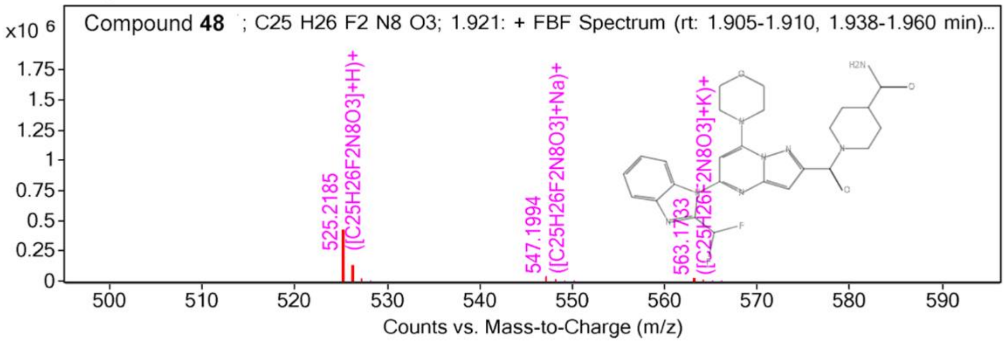

MS Zoomed Spectrum

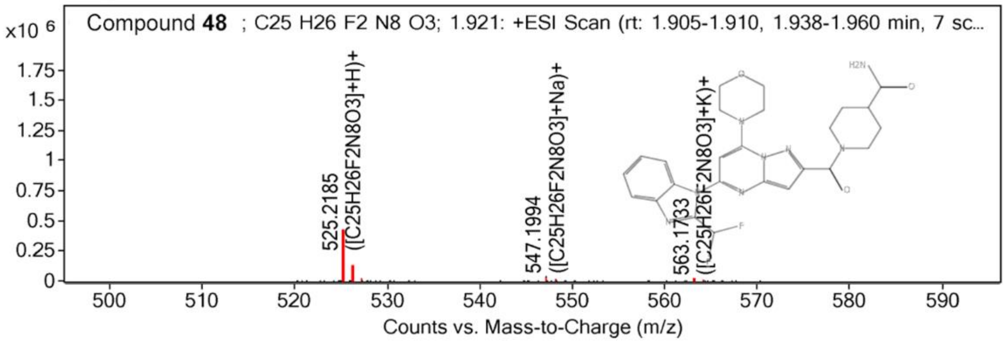

MS Spectrum Peak List

Obs. m/z	Charge	Abund	Formula	Ion/Isotope	Tgt Mass Error (ppm)
525.2185	1	418105.22	C25H26F2N8O3	(M+H)+	
525.2185	1	418105.22	C25H26F2N8O3	(M+H)+	3.18

--- End Of Report ---

Compound 49

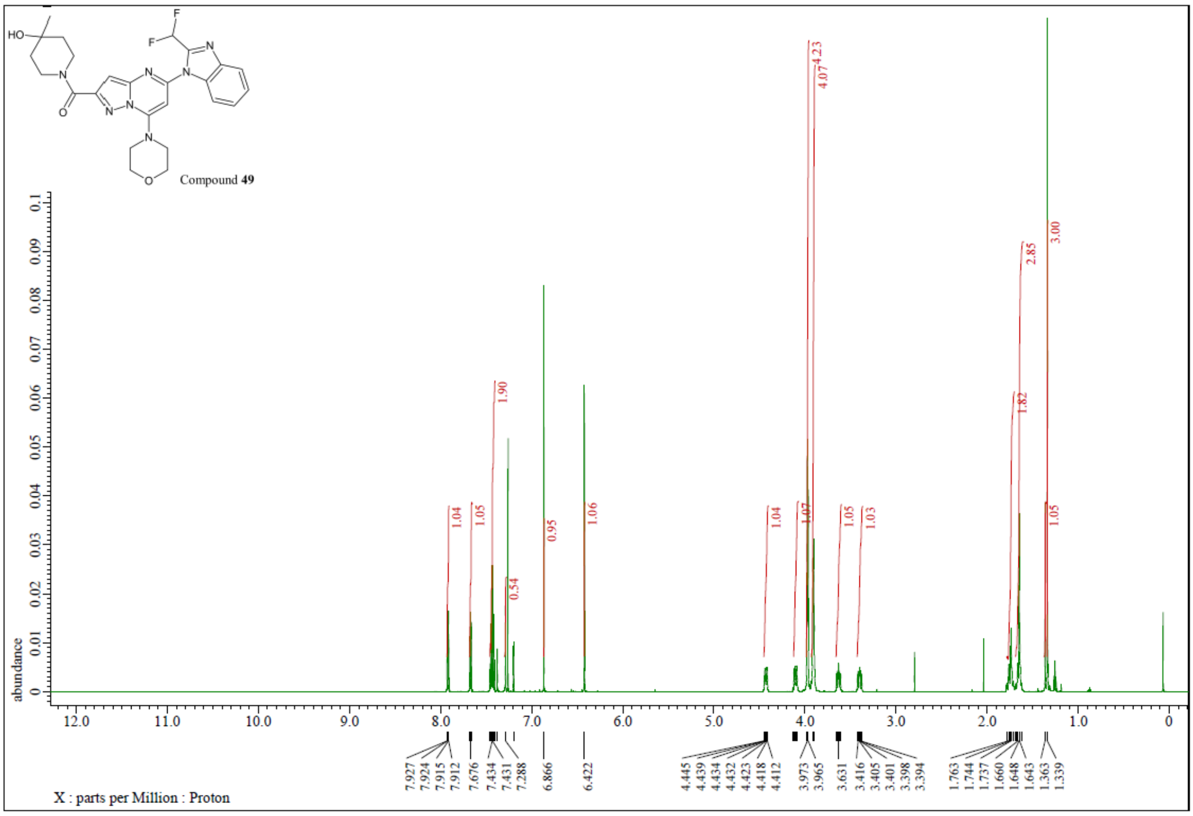
Compound 49
abundance
X : parts per Million : Proton

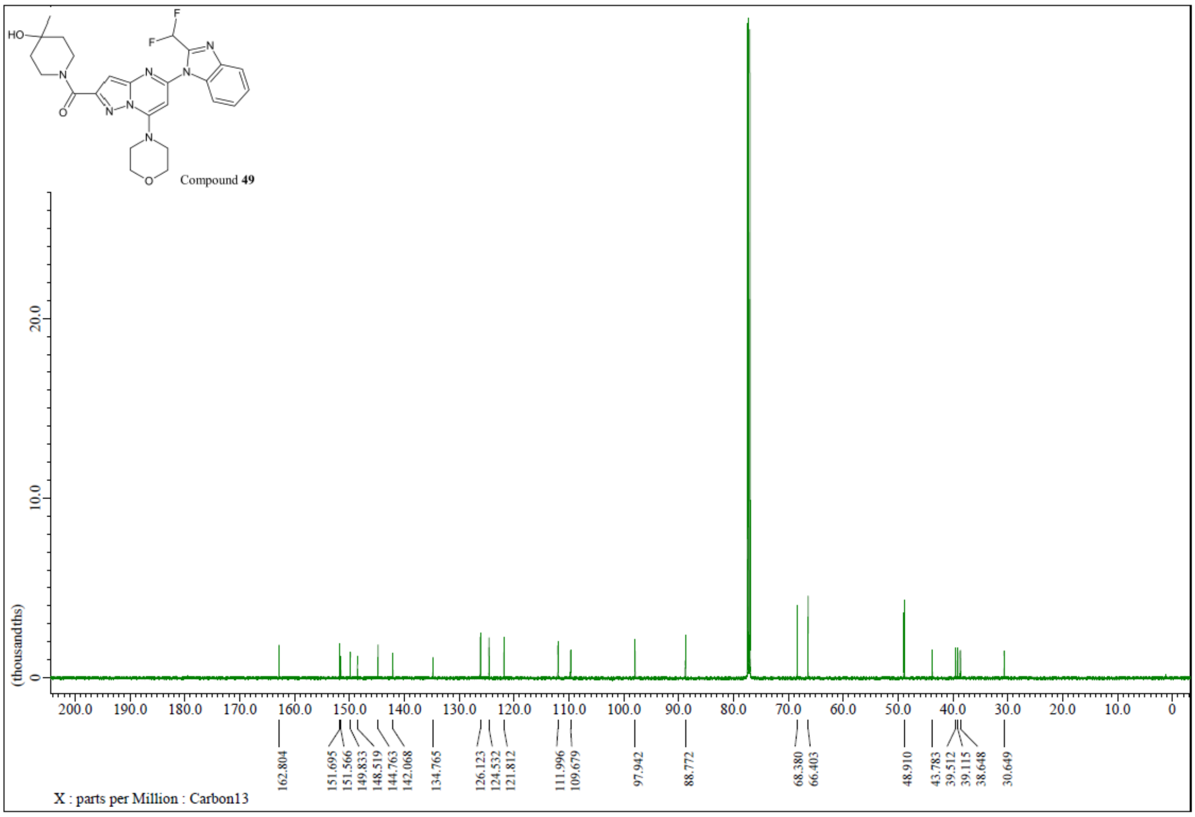
Compound 49
(thousandths)
X : parts per Million : Carbon13

Compound 49

Name	*Obs. m/z*	Obs. RT	Obs. Mass	Tgt Formula	Tgt Mass	Tgt Mass Error	Find Cpds Algorit
Compound **49**	534.2049	2.111	511.2166	C25 H27 F2 N7 O3	511.2143	4.48	Find by Formula

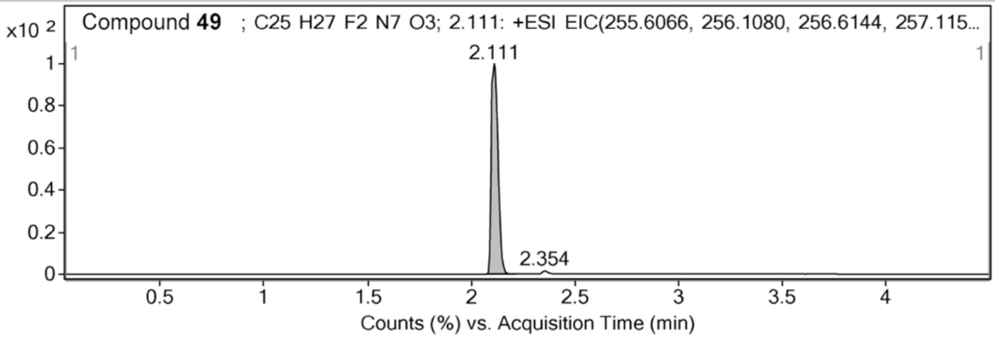

MS Zoomed Spectrum

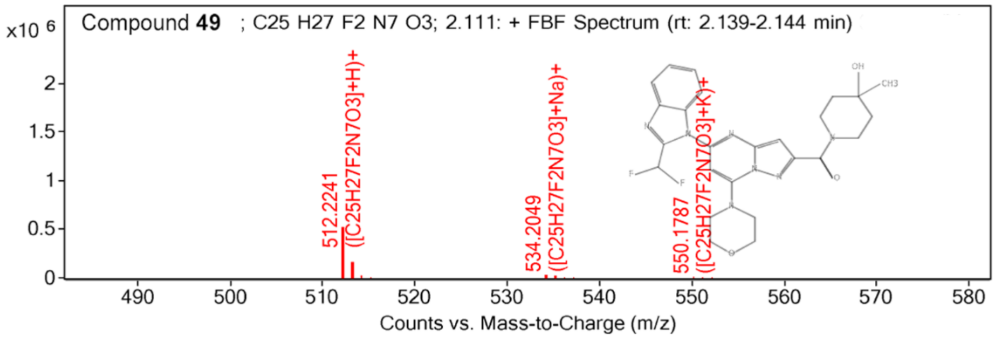

MS Zoomed Spectrum

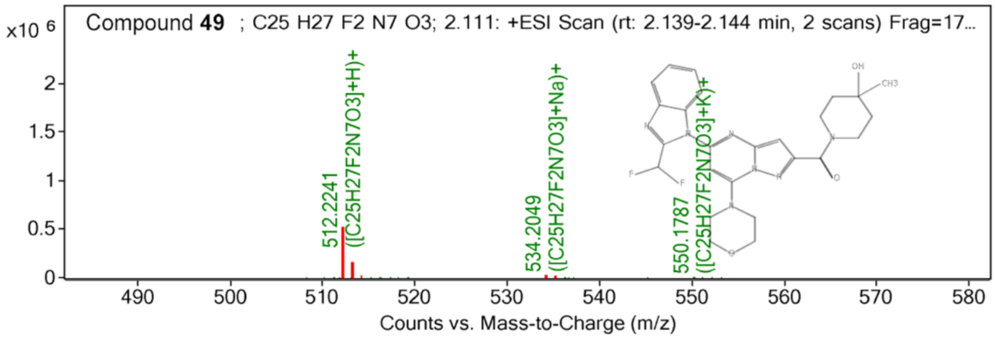

MS Spectrum Peak List

Obs. m/z	Charge	Abund	Formula	Ion/Isotope	Tgt Mass Error (ppm)
512.2241	1	513087.13	C25H27F2N7O3	(M+H)+	
512.2241	1	513087.13	C25H27F2N7O3	(M+H)+	4.76

--- End Of Report ---

Compound 50

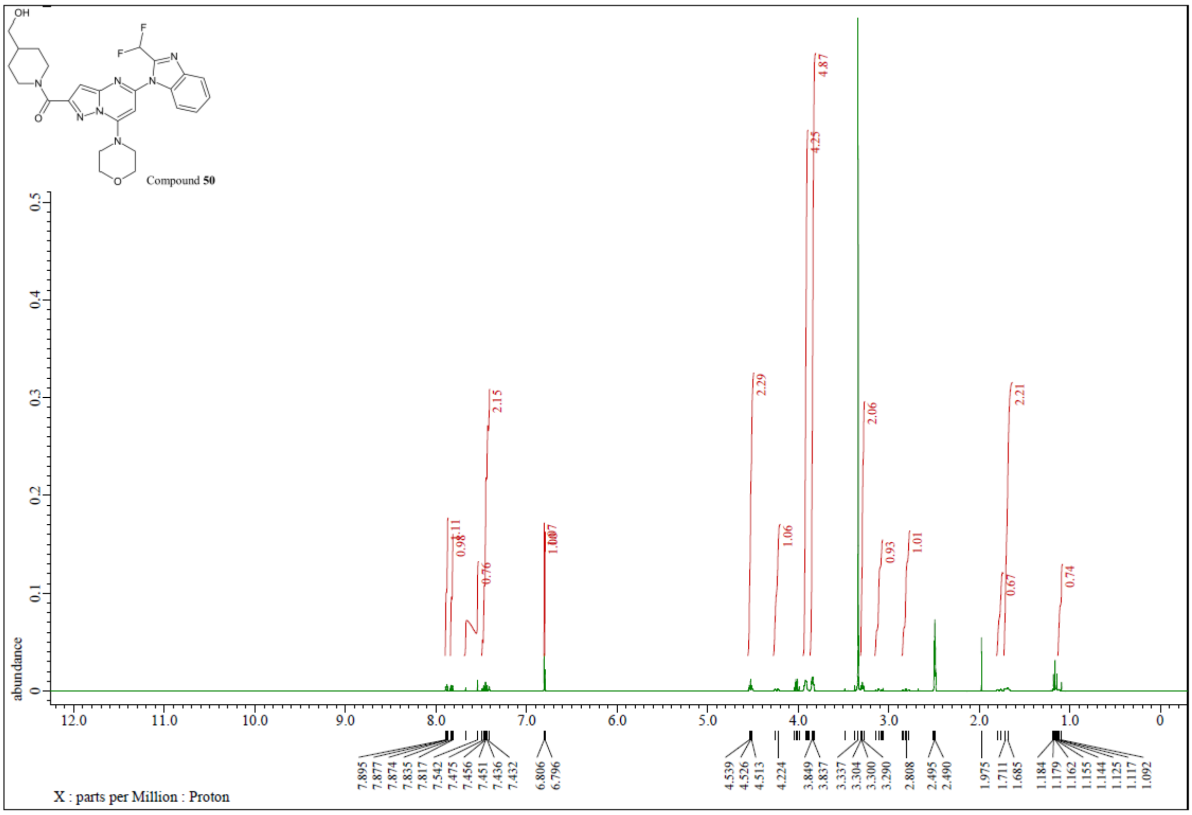
Compound 50
abundance
X : parts per Million : Proton

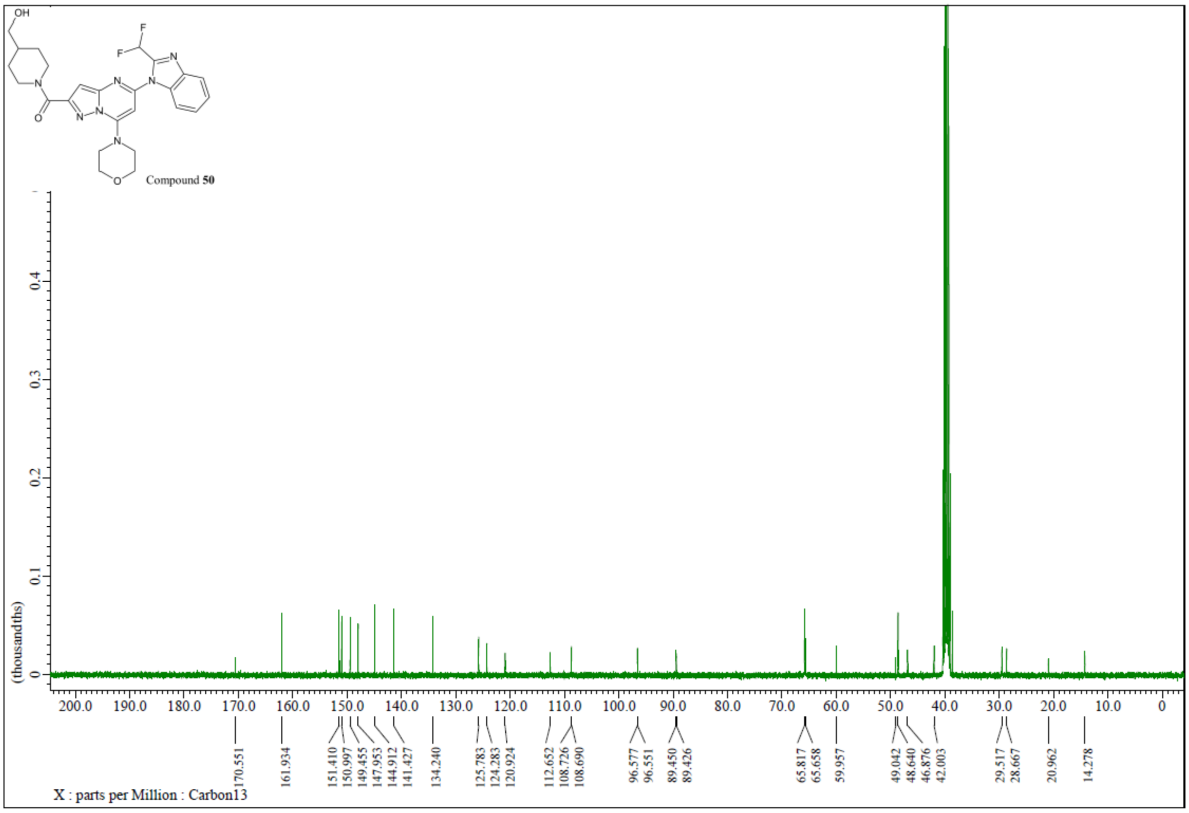
Compound 50
(thousandths)
X : parts per Million : Carbon13

Compound 50

Name	*Obs. m/z*	Obs. RT	Obs. Mass	Tgt Formula	Tgt Mass	Tgt Mass Error	Find Cpds Algorit
Compound **50**	534.2048	2.061	511.2169	C25 H27 F2 N7 O3	511.2143	4.98	Find by Formula

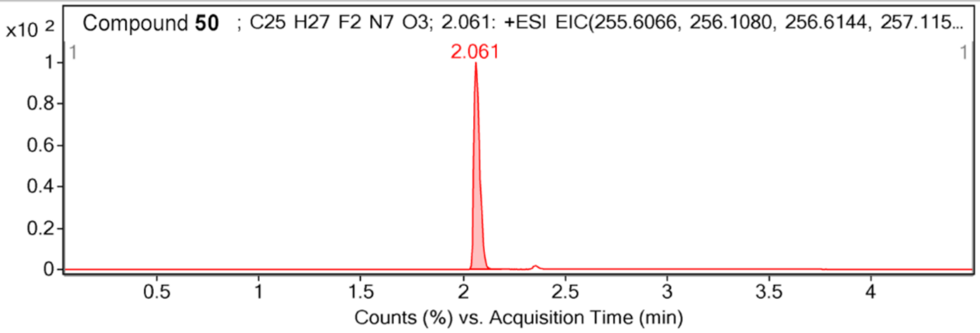

MS Zoomed Spectrum

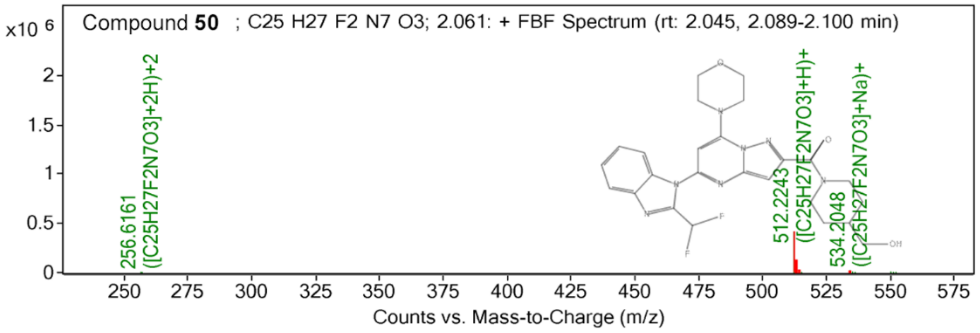

MS Zoomed Spectrum

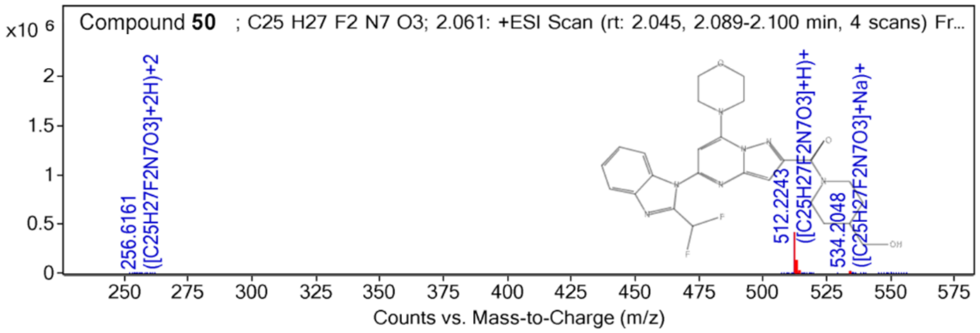

MS Spectrum Peak List

Obs. m/z	Charge	Abund	Formula	Ion/Isotope	Tgt Mass Error (ppm)
512.2243	1	414545.78	C25H27F2N7O3	(M+H)+	
512.2243	1	414545.78	C25H27F2N7O3	(M+H)+	5.32

--- End Of Report ---

Compound 51

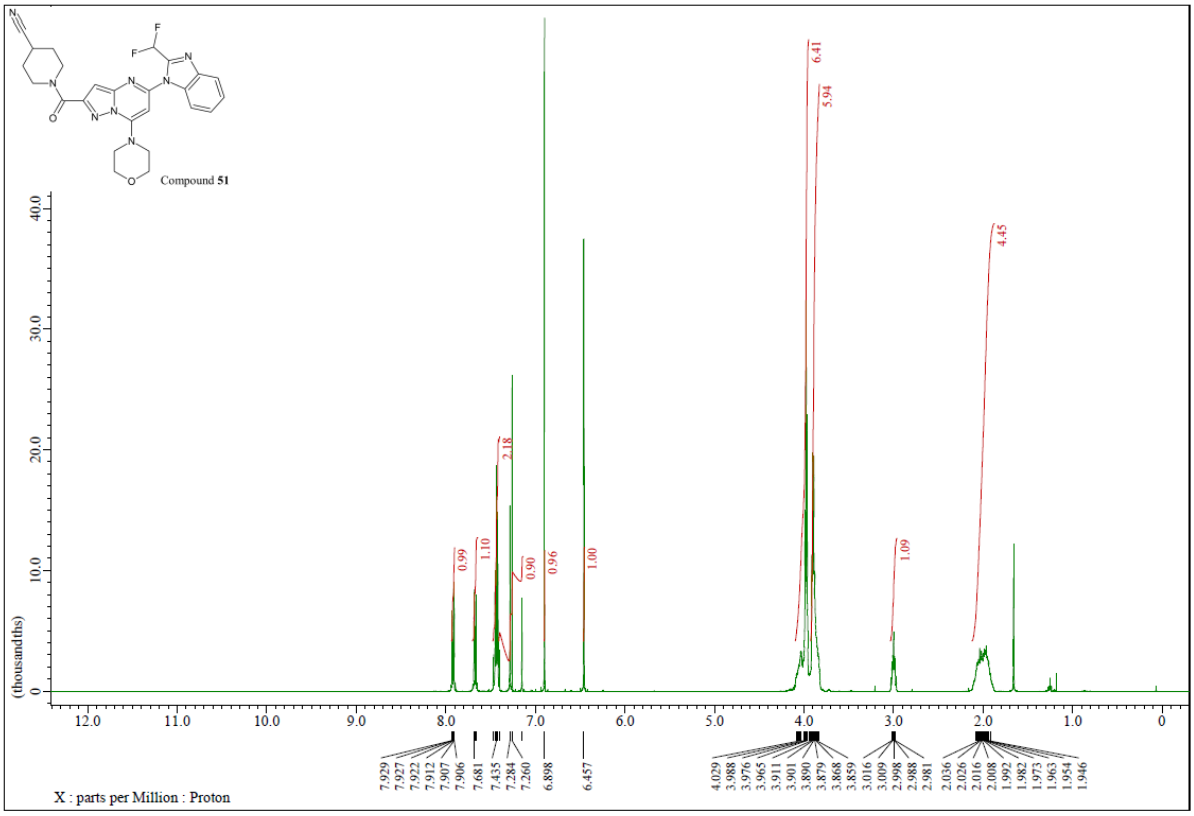
Compound 51
X : parts per Million : Proton
(thousandths)
7.929 7.927 7.922 7.912 7.907 7.906 7.681 7.435 7.284 7.260 6.898 6.457
4.029 3.988 3.976 3.965 3.911 3.901 3.890 3.879 3.868 3.859 3.016 3.009 2.998 2.988 2.981 2.036 2.026 2.016 2.008 1.992 1.982 1.973 1.963 1.954 1.946
0.99 1.10 2.18 0.90 0.96 1.00 6.41 5.94 1.09 4.45

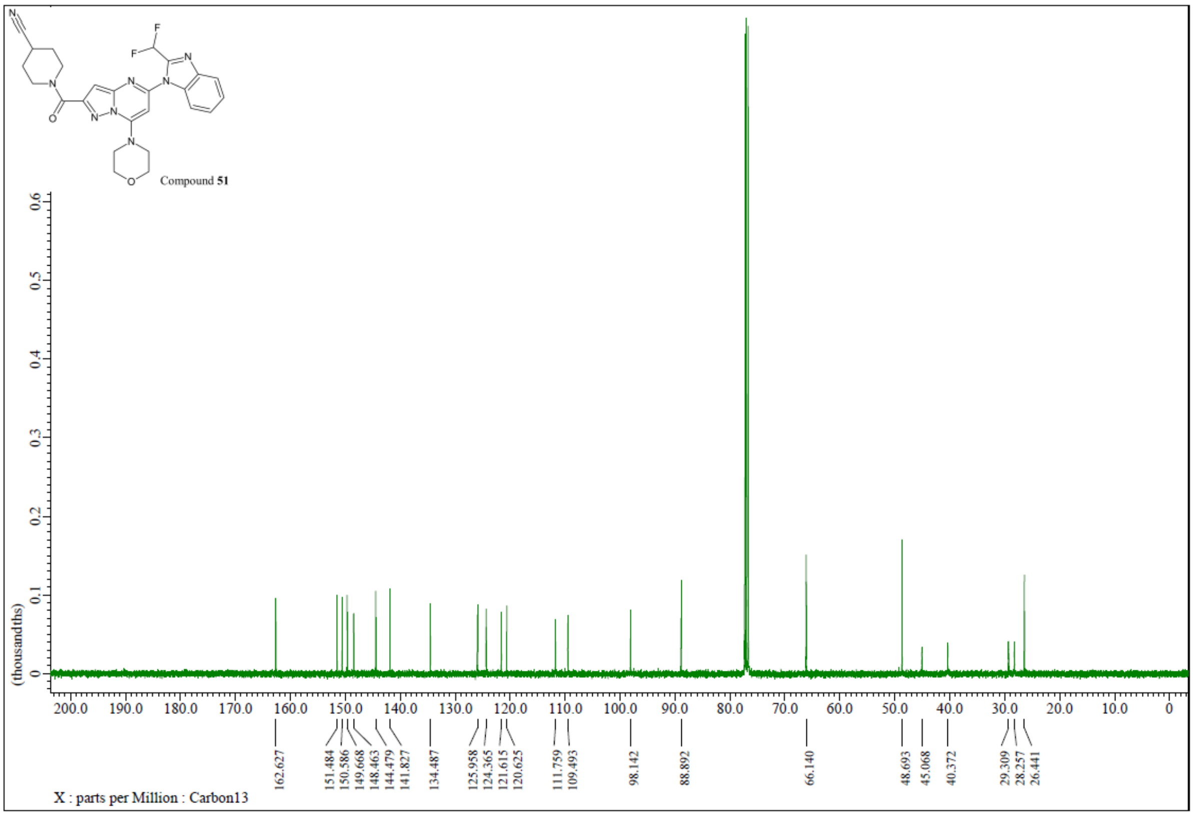
Compound 51
X : parts per Million : Carbon13
(thousandths)
162.627 151.484 150.586 149.668 148.463 144.479 141.827 134.487 125.958 124.365 121.615 120.625 111.759 109.493 98.142 88.892 66.140 48.693 45.068 40.372 29.309 28.257 26.441

Compound 51

Name	*Obs. m/z*	Obs. RT	Obs. Mass	Tgt Formula	Tgt Mass	Tgt Mass Error	Find Cpds Algorith
Compound **51**	245.1528	2.343	488.2908	C28 H36 N6 O2	488.29	1.65	Find by Formula

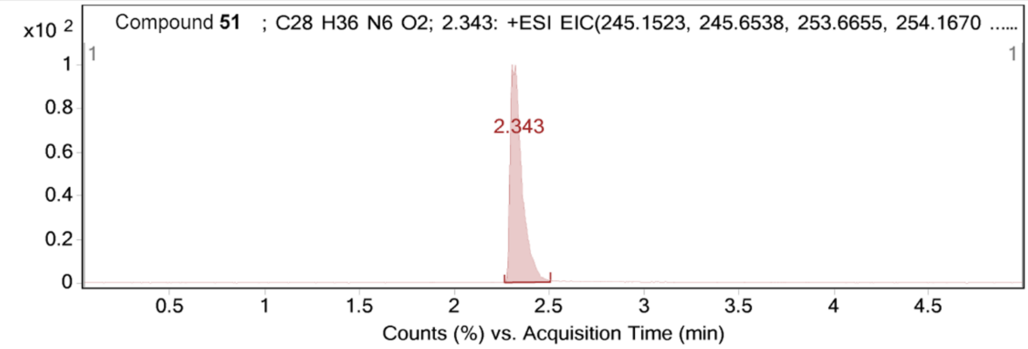

MS Zoomed Spectrum

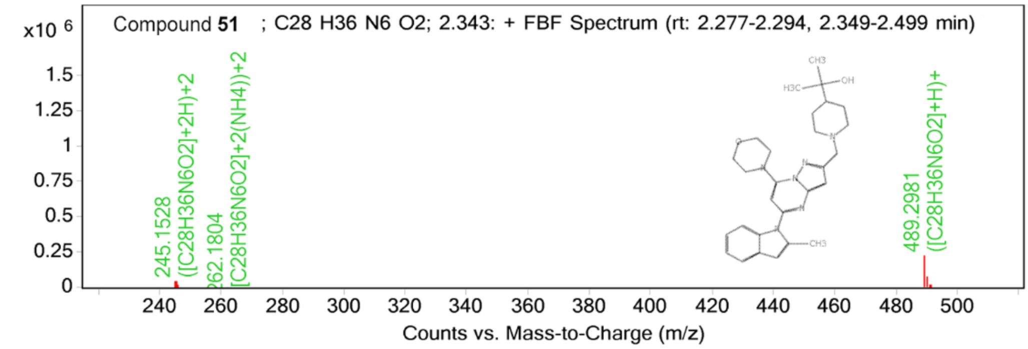

MS Zoomed Spectrum

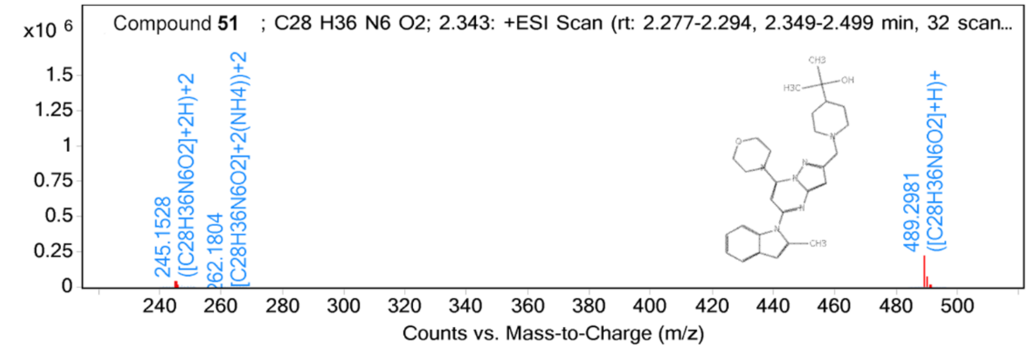

MS Spectrum Peak List

Obs. m/z	Charge	Abund	Formula	Ion/Isotope	Tgt Mass Error (ppm)
245.1528	2	39305.1	C28H36N6O2	(M+2H)+2	
245.6541	2	13220.31	C28H36N6O2	(M+2H)+2	
246.1556	2	2237.2	C28H36N6O2	(M+2H)+2	
246.656	2	264.83	C28H36N6O2	(M+2H)+2	
262.1804	2	2396.03	C28H36N6O2	(M+2(NH4))+2	
263.1846	2	484.19	C28H36N6O2	(M+2(NH4))+2	
489.2981	1	220426.55	C28H36N6O2	(M+H)+	
490.3008	1	64901.46	C28H36N6O2	(M+H)+	
491.3026	1	10140.32	C28H36N6O2	(M+H)+	
245.1528	2	39305.1	C28H36N6O2	(M+2H)+2	2.01
245.6541	2	13220.31	C28H36N6O2	(M+2H)+2	1.29
246.1556	2	2237.2	C28H36N6O2	(M+2H)+2	1.57
246.656	2	264.83	C28H36N6O2	(M+2H)+2	-2.37
262.1804	2	2396.03	C28H36N6O2	(M+2(NH4))+2	6
263.1846	2	484.19	C28H36N6O2	(M+2(NH4))+2	11.24
489.2981	1	220426.55	C28H36N6O2	(M+H)+	1.78
490.3008	1	64901.46	C28H36N6O2	(M+H)+	1.18

Compound 52

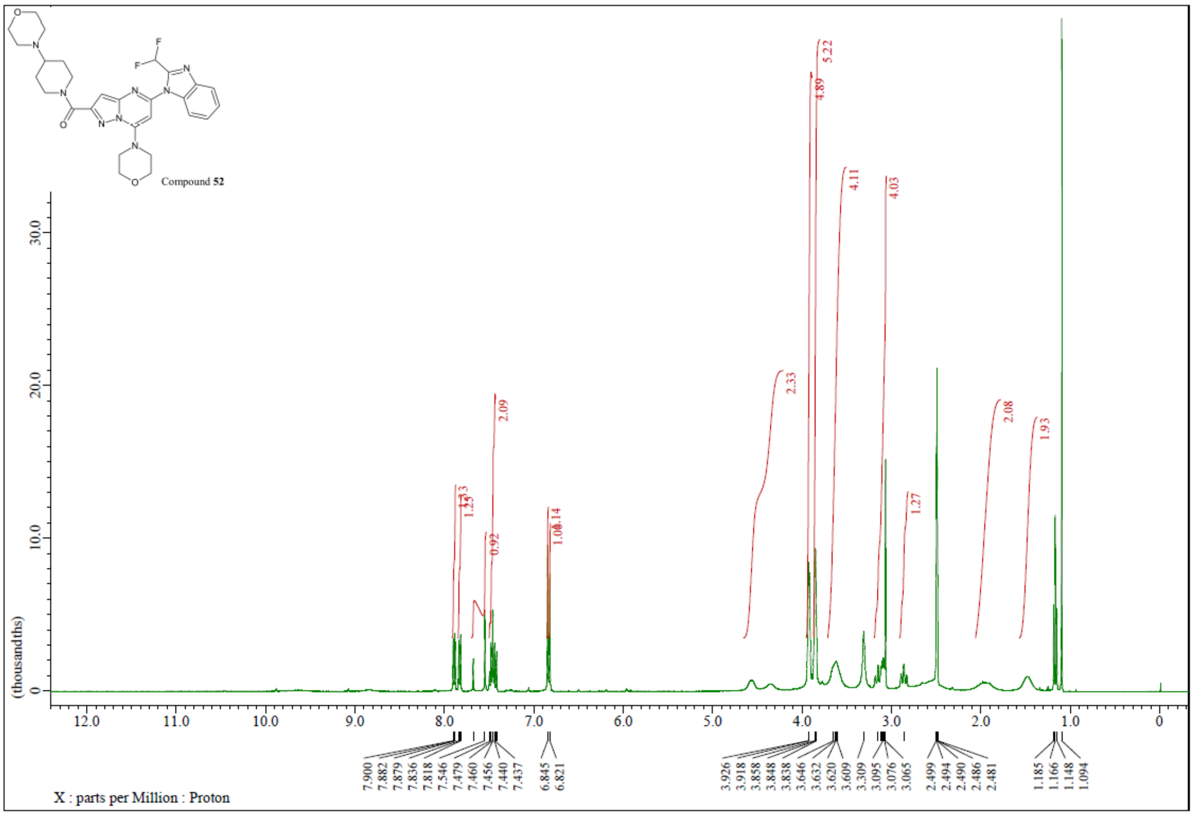

Compound 52
(thousandths)
X : parts per Million : Proton

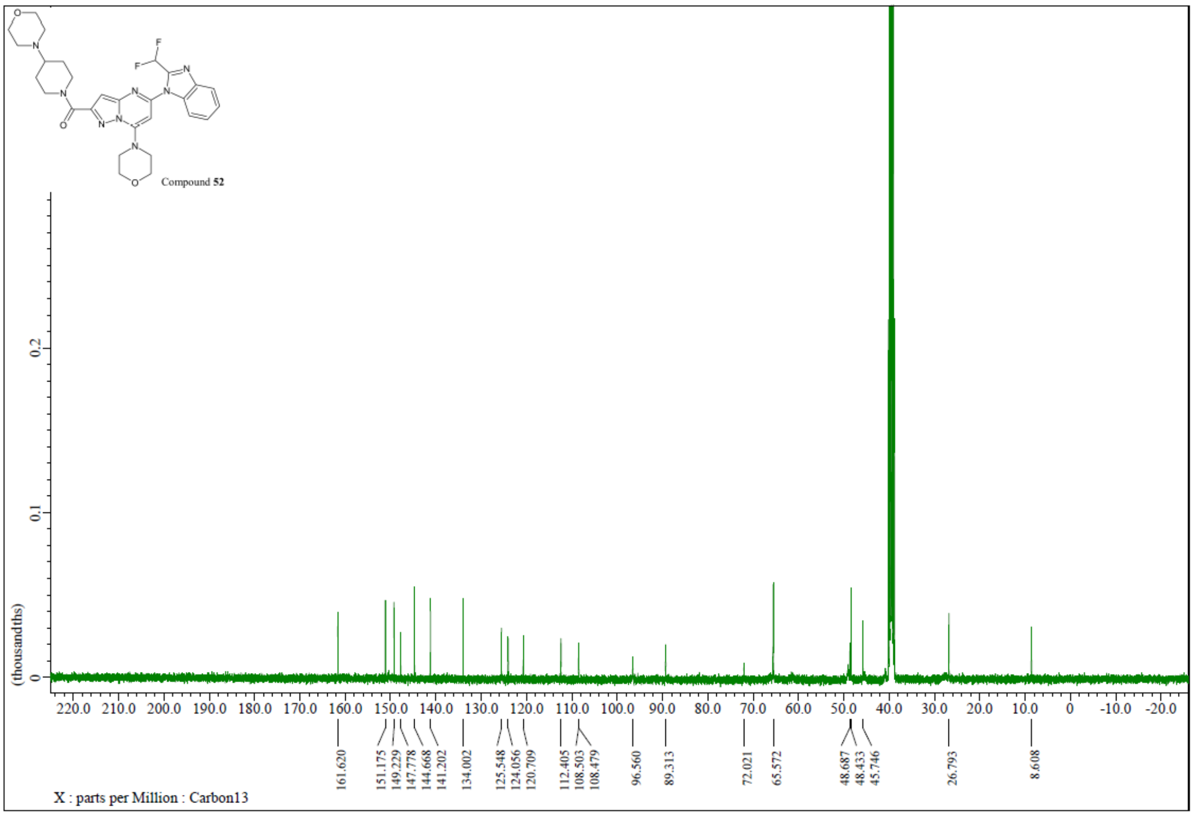

Compound 52
(thousandths)
X : parts per Million : Carbon13
161.620
151.175
149.229
147.778
144.668
141.202
134.002
125.548
124.056
120.709
112.405
108.503
108.479
96.560
89.313
72.021
65.572
48.687
48.433
45.746
26.793
8.608

Compound 52

Name	*Obs. m/z*	Obs. RT	Obs. Mass	Tgt Formula	Tgt Mass	Tgt Mass Error	Find Cpds Algorit
Compound **52**	567.2635	1.715	566.2563	C28 H32 F2 N8 O3	566.2565	-0.37	Find by Formula

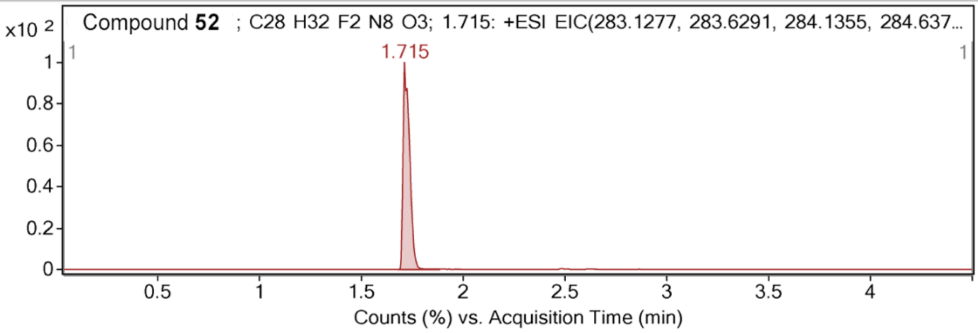

MS Zoomed Spectrum

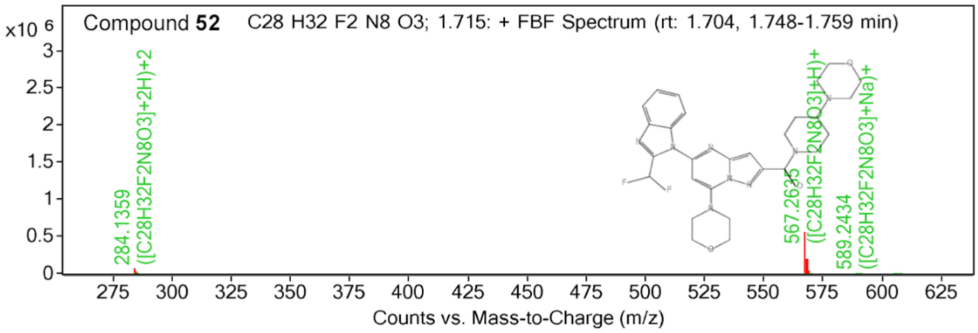

MS Zoomed Spectrum

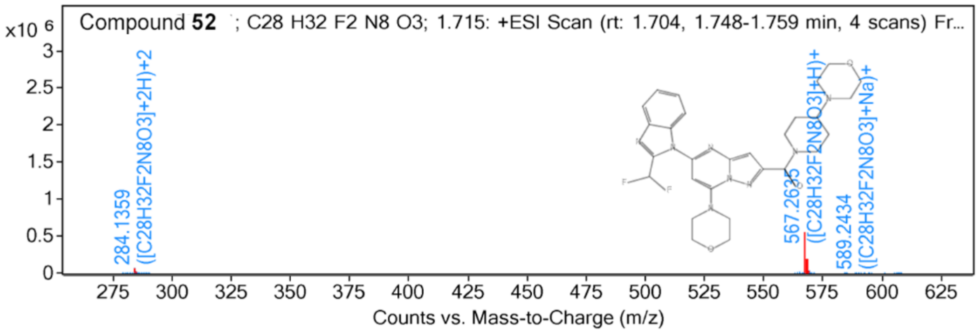

MS Spectrum Peak List

Obs. m/z	Charge	Abund	Formula	Ion/Isotope	Tgt Mass Error (ppm)
567.2635	1	550689.94	C28H32F2N8O3	(M+H)+	
567.2635	1	550689.94	C28H32F2N8O3	(M+H)+	-0.54

--- End Of Report ---

Compound 53

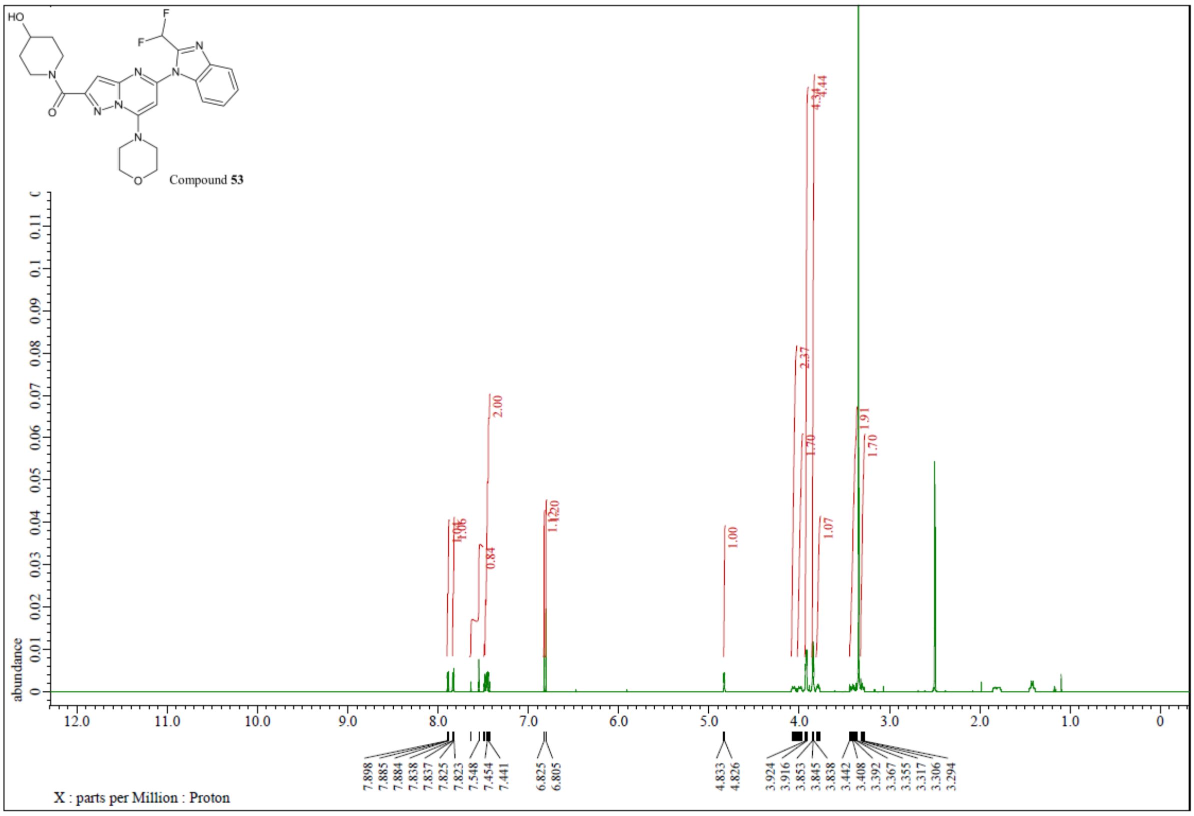

Compound 53
abundance
X : parts per Million : Proton
7.898
7.885
7.884
7.838
7.837
7.825
7.823
7.548
7.454
7.441
6.825
6.805
4.833
4.826
3.924
3.916
3.853
3.845
3.838
3.442
3.408
3.392
3.367
3.355
3.317
3.306
3.294

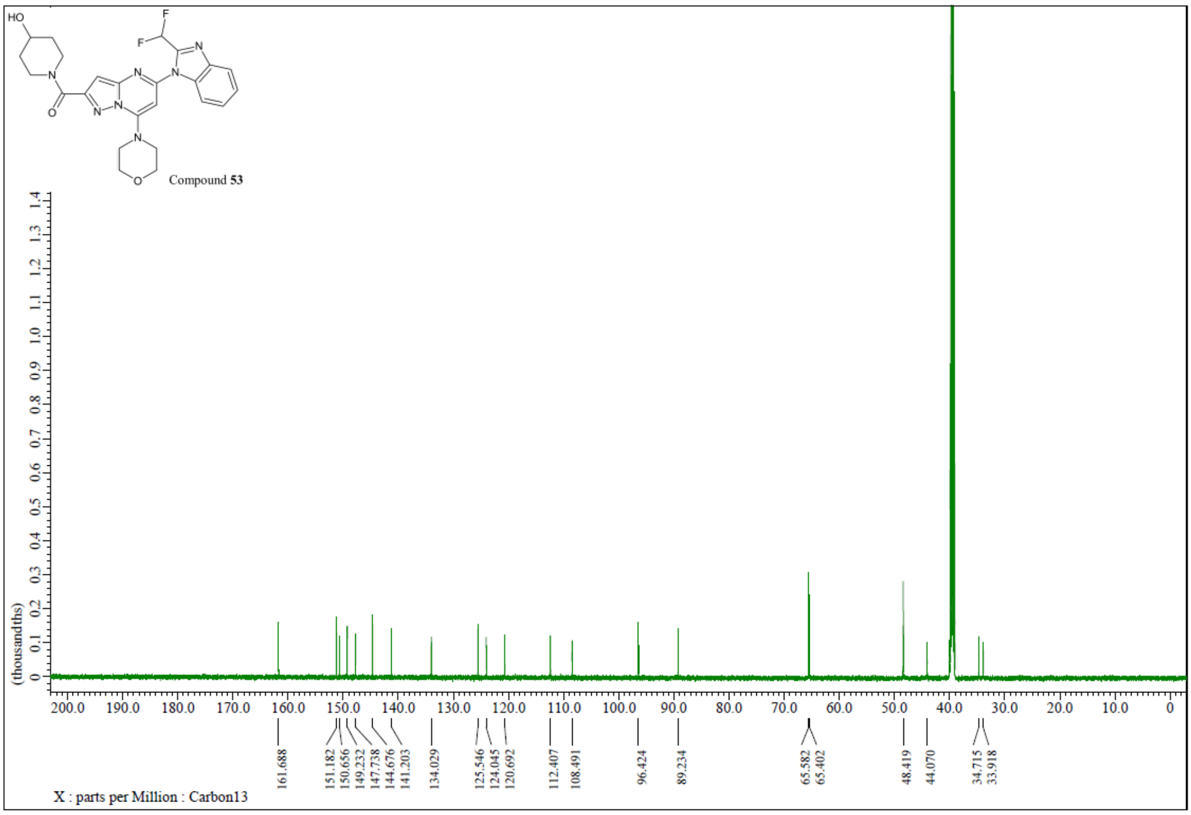

Compound 53
(thousandths)
X : parts per Million : Carbon13
161.688
151.182
150.656
149.232
147.738
144.676
141.203
134.029
125.546
124.045
120.692
112.407
108.491
96.424
89.234
65.582
65.402
48.419
44.070
34.715
33.918

Compound 53

Name	*Obs. m/z*	Obs. RT	Obs. Mass	Tgt Formula	Tgt Mass	Tgt Mass Error	Find Cpds Algorit
Compound **53**	256.12283	1.17	510.23062	C25 H28 F2 N8 O2	510.23033	0.57	Find by Formula

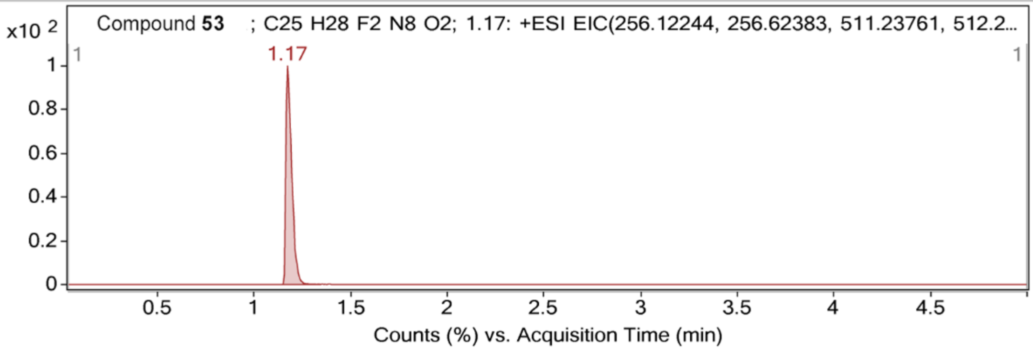

MS Zoomed Spectrum

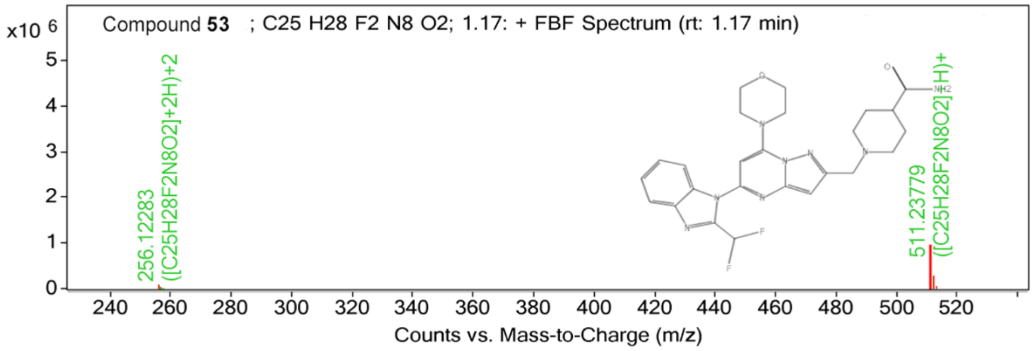

MS Zoomed Spectrum

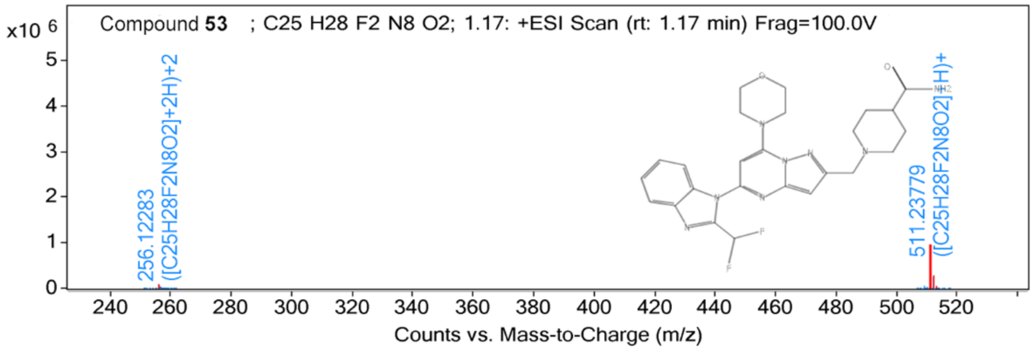

MS Spectrum Peak List

Obs. m/z	Charge	Abund	Formula	Ion/Isotope	Tgt Mass Error (ppm)
256.12283	2	72301.37	C25H28F2N8O2	(M+2H)+2	
256.62429	2	24112.44	C25H28F2N8O2	(M+2H)+2	
257.12683	2	2715.31	C25H28F2N8O2	(M+2H)+2	
257.6261	2	442.92	C25H28F2N8O2	(M+2H)+2	
511.23779	1	947387.88	C25H28F2N8O2	(M+H)+	
512.24099	1	244045.55	C25H28F2N8O2	(M+H)+	
513.24241	1	39640.75	C25H28F2N8O2	(M+H)+	
256.12283	2	72301.37	C25H28F2N8O2	(M+2H)+2	1.52
256.62429	2	24112.44	C25H28F2N8O2	(M+2H)+2	1.8
257.12683	2	2715.31	C25H28F2N8O2	(M+2H)+2	6.5
257.6261	2	442.92	C25H28F2N8O2	(M+2H)+2	-1.28
511.23779	1	947387.88	C25H28F2N8O2	(M+H)+	0.36
512.24099	1	244045.55	C25H28F2N8O2	(M+H)+	1.18
513.24241	1	39640.75	C25H28F2N8O2	(M+H)+	-1.22

--- End Of Report ---

Compound 54

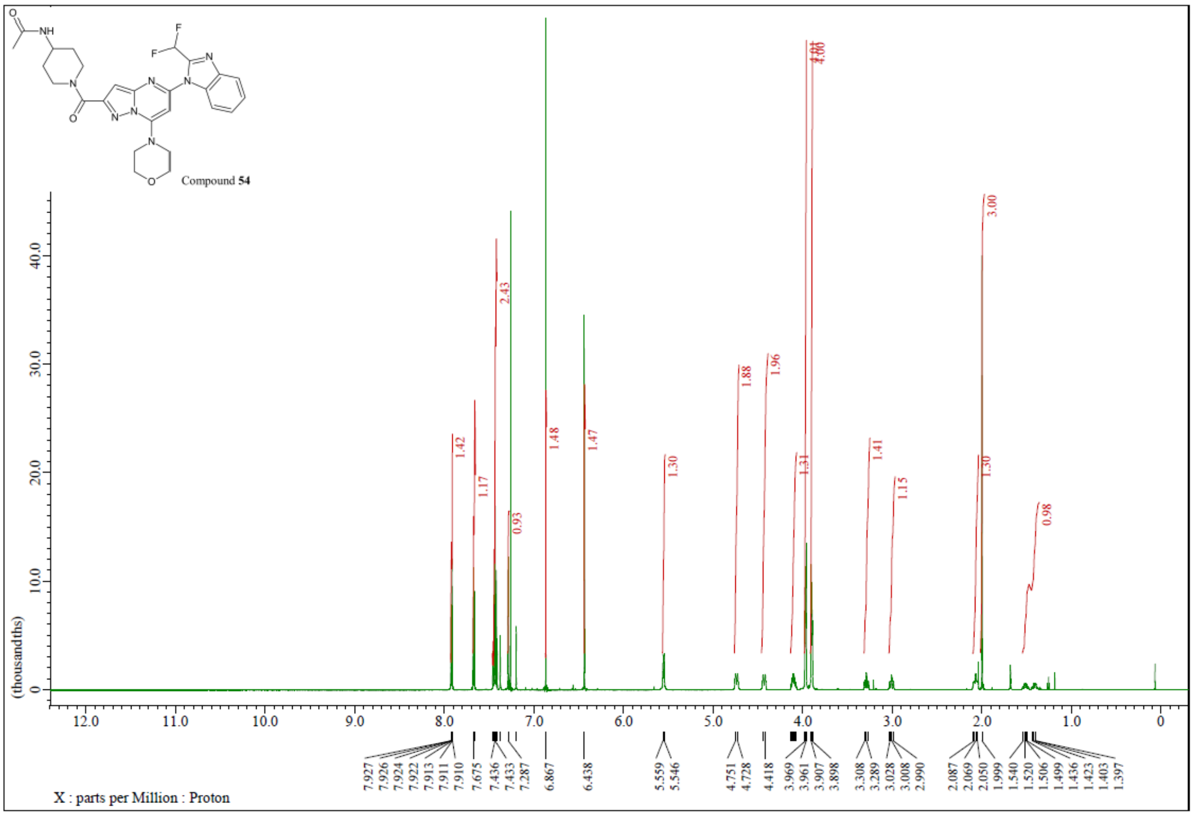
Compound 54
X : parts per Million : Proton
(thousandths)

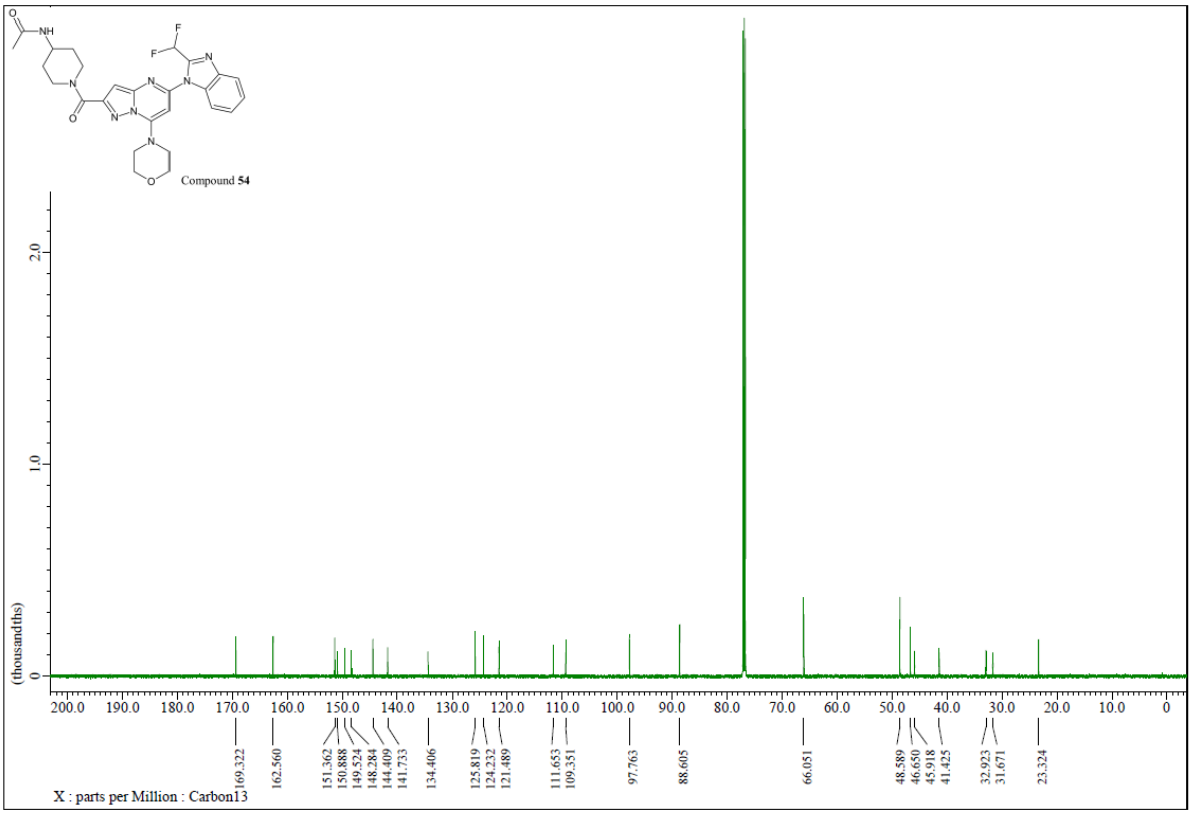
Compound 54
X : parts per Million : Carbon13
(thousandths)

Compound 54

Name	*Obs. m/z*	Obs. RT	Obs. Mass	Tgt Formula	Tgt Mass	Tgt Mass Error	Find Cpds Algorit
Compound **54**	561.2167	1.24	538.2275	C26 H28 F2 N8 O3	538.2252	4.19	Find by Formula

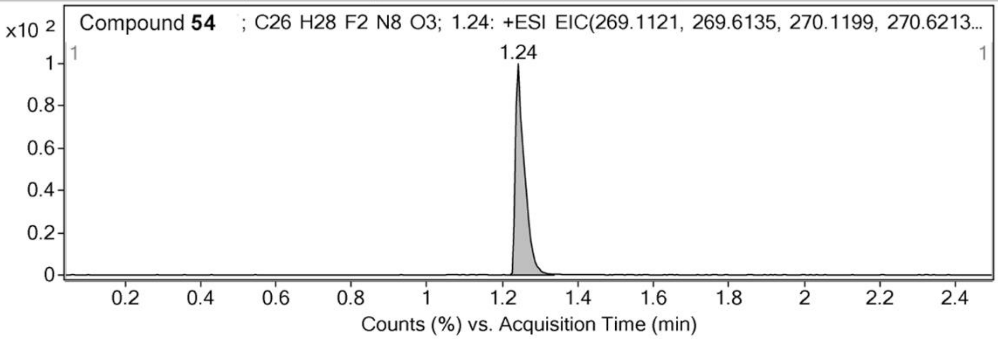

MS Zoomed Spectrum

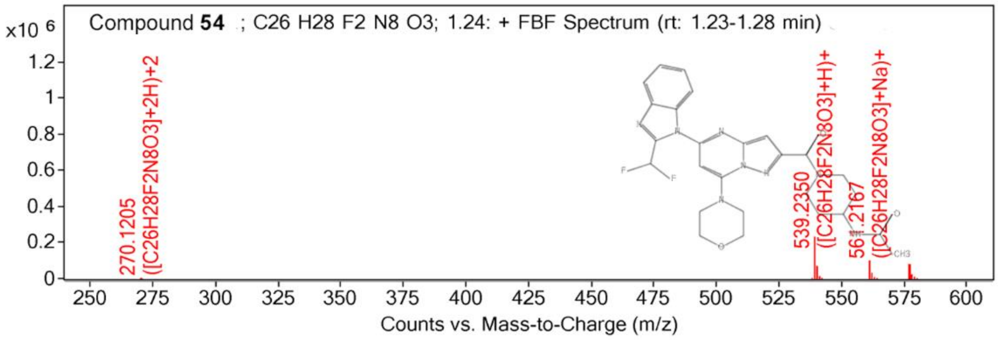

MS Zoomed Spectrum

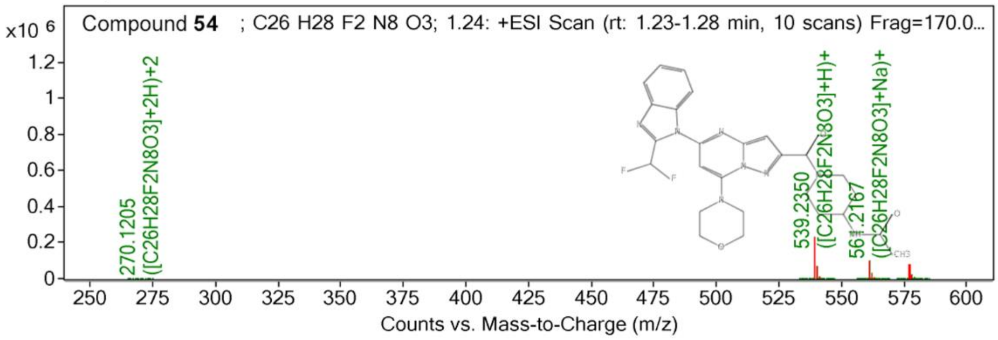

MS Spectrum Peak List

Obs. m/z	Charge	Abund	Formula	Ion/Isotope	Tgt Mass Error (ppm)
539.235	1	231005.8	C26H28F2N8O3	(M+H)+	
539.235	1	231005.8	C26H28F2N8O3	(M+H)+	4.65

--- End Of Report ---

Compound 55

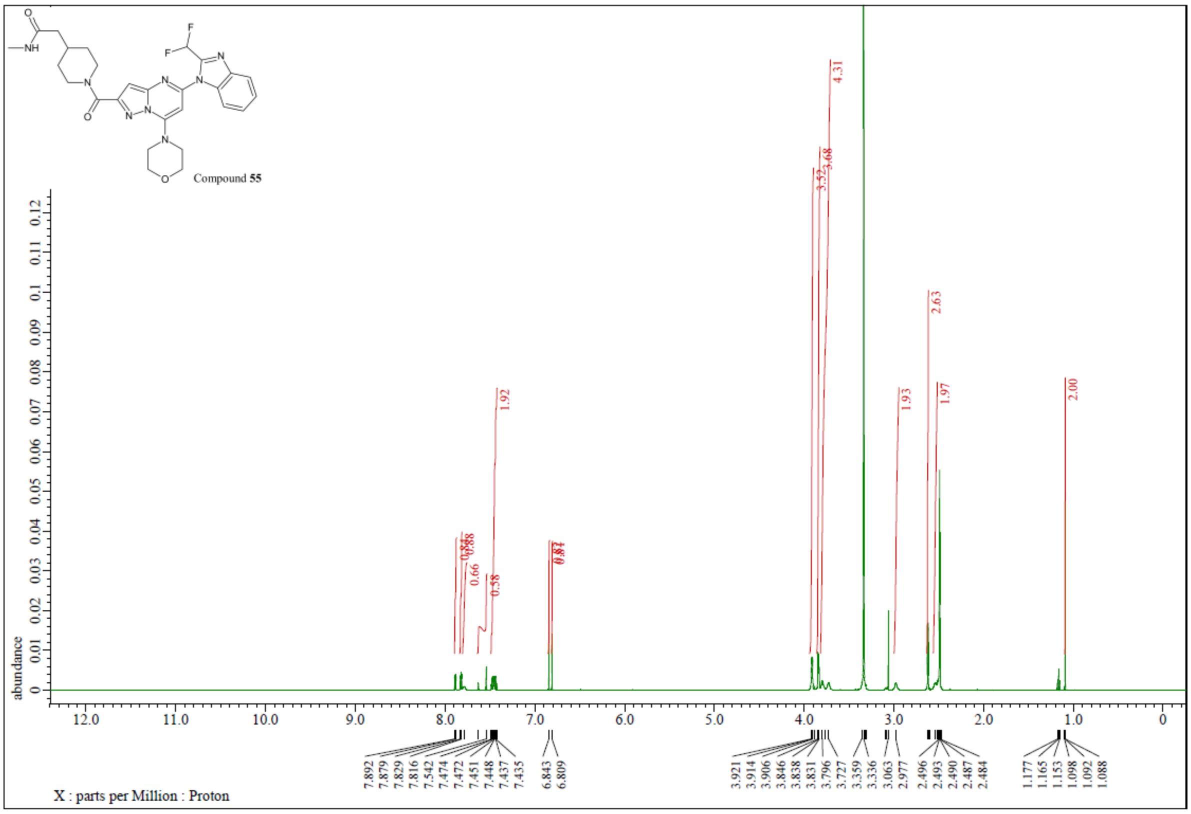
Compound 55
abundance
X : parts per Million : Proton
7.892
7.879
7.829
7.816
7.542
7.474
7.472
7.451
7.448
7.437
7.435
6.843
6.809
3.921
3.914
3.906
3.846
3.838
3.831
3.796
3.727
3.359
3.336
3.063
2.977
2.496
2.493
2.490
2.487
2.484
1.177
1.165
1.153
1.098
1.092
1.088
1.92
0.66
0.58
4.31
1.93
2.63
1.97
2.00

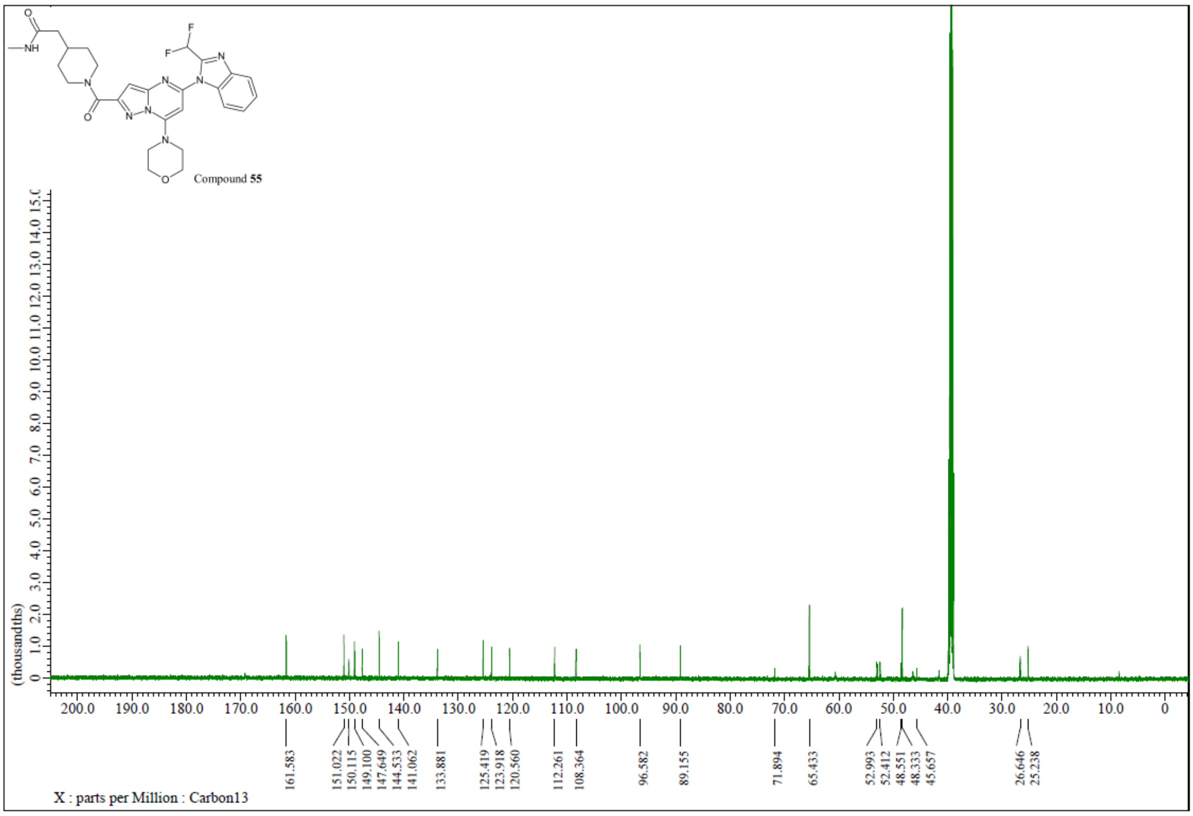
Compound 55
(thousandths)
X : parts per Million : Carbon13
161.583
151.022
150.115
149.100
147.649
144.533
141.062
133.881
125.419
123.918
120.560
112.261
108.364
96.582
89.155
71.894
65.433
52.993
52.412
48.551
48.333
45.657
26.646
25.238

Compound 55

Compound Label	Name	*m/z*	RT	Algorithm	Mass
Compound **55**	Compound **55**	554.24424	1.37	Find by Formula	553.23715

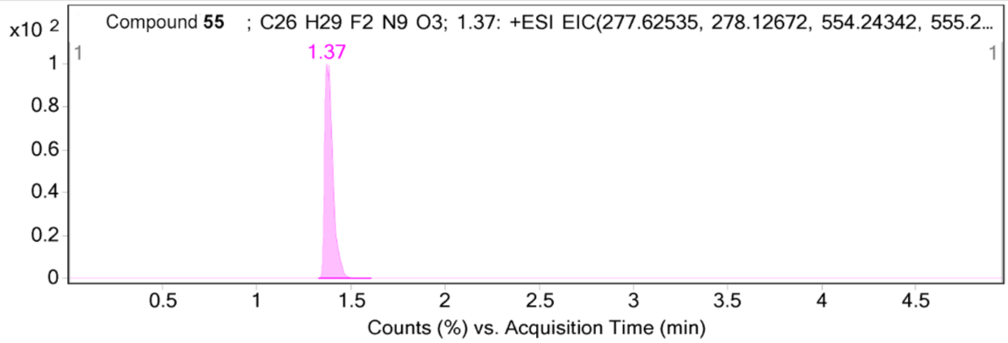

MS Zoomed Spectrum

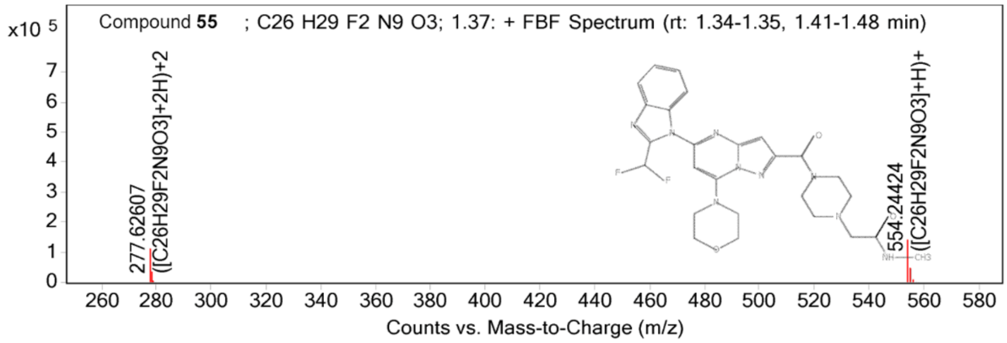

MS Zoomed Spectrum

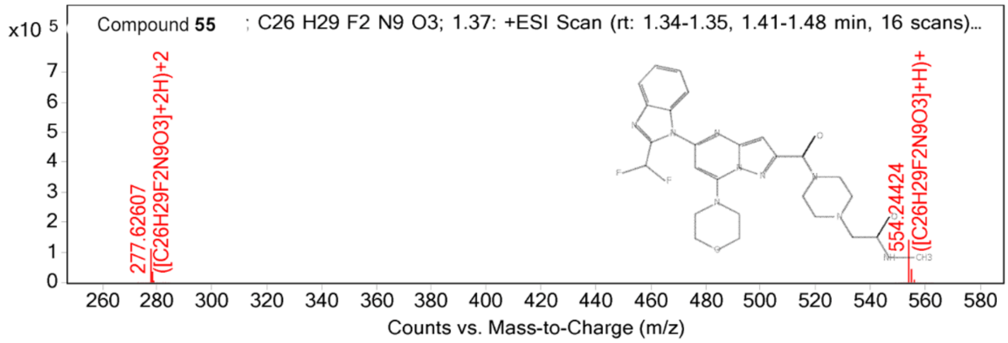

MS Spectrum Peak List

m/z	*Calc m/z*	Diff(ppm)	z	Abund	Formula	Ion
277.62607	277.62535	2.62	2	112238.74	C26H29F2N9O3	(M+2H)+2
278.1273	278.12672	2.09	2	35826.95	C26H29F2N9O3	(M+2H)+2
278.62837	278.62801	1.3	2	6205.88	C26H29F2N9O3	(M+2H)+2
279.12947	279.12925	0.76	2	958.56	C26H29F2N9O3	(M+2H)+2
279.62869	279.63048	-6.4	2	61.35	C26H29F2N9O3	(M+2H)+2
554.24424	554.24342	1.47	1	140783.94	C26H29F2N9O3	(M+H)+
555.24673	555.24616	1.03	1	43994.18	C26H29F2N9O3	(M+H)+
556.24896	556.24874	0.41	1	6954.29	C26H29F2N9O3	(M+H)+
557.25101	557.25123	-0.39	1	888.06	C26H29F2N9O3	(M+H)+
558.25274	558.25367	-1.68	1	85.26	C26H29F2N9O3	(M+H)+

--- End Of Report ---

Compound 56

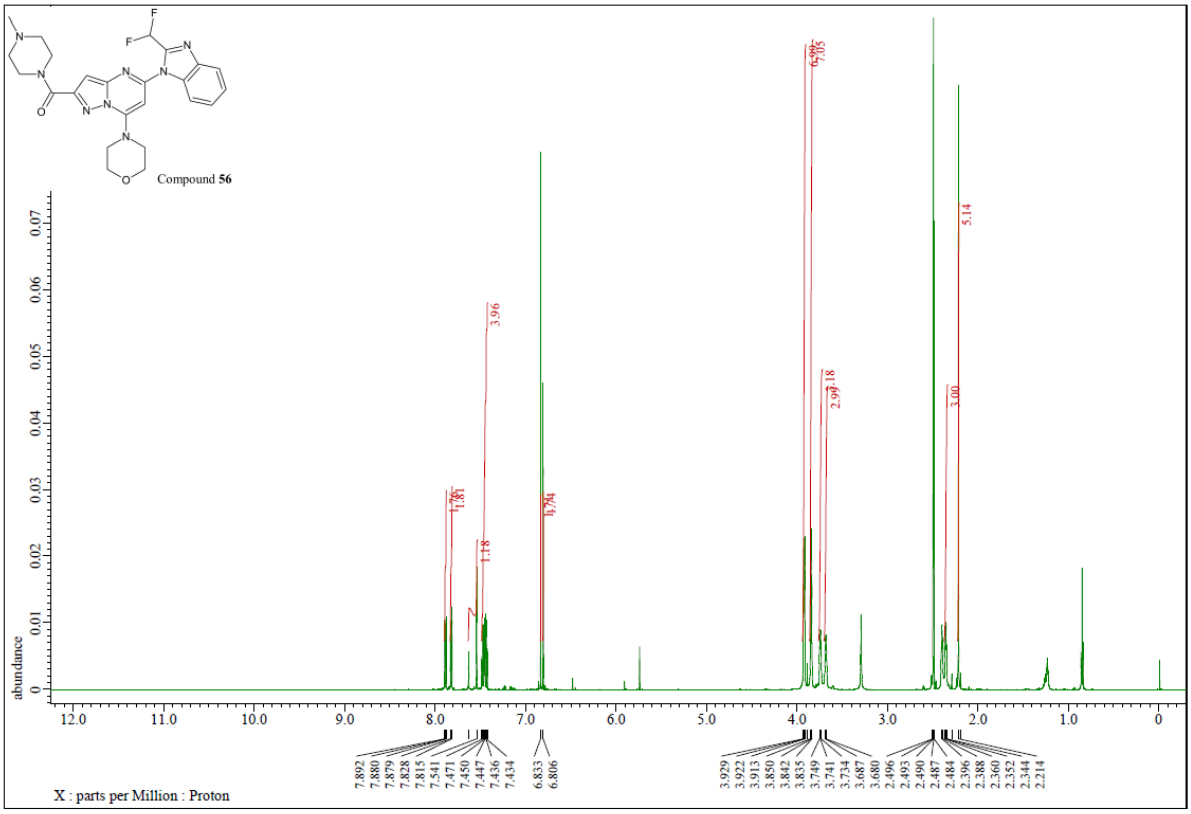

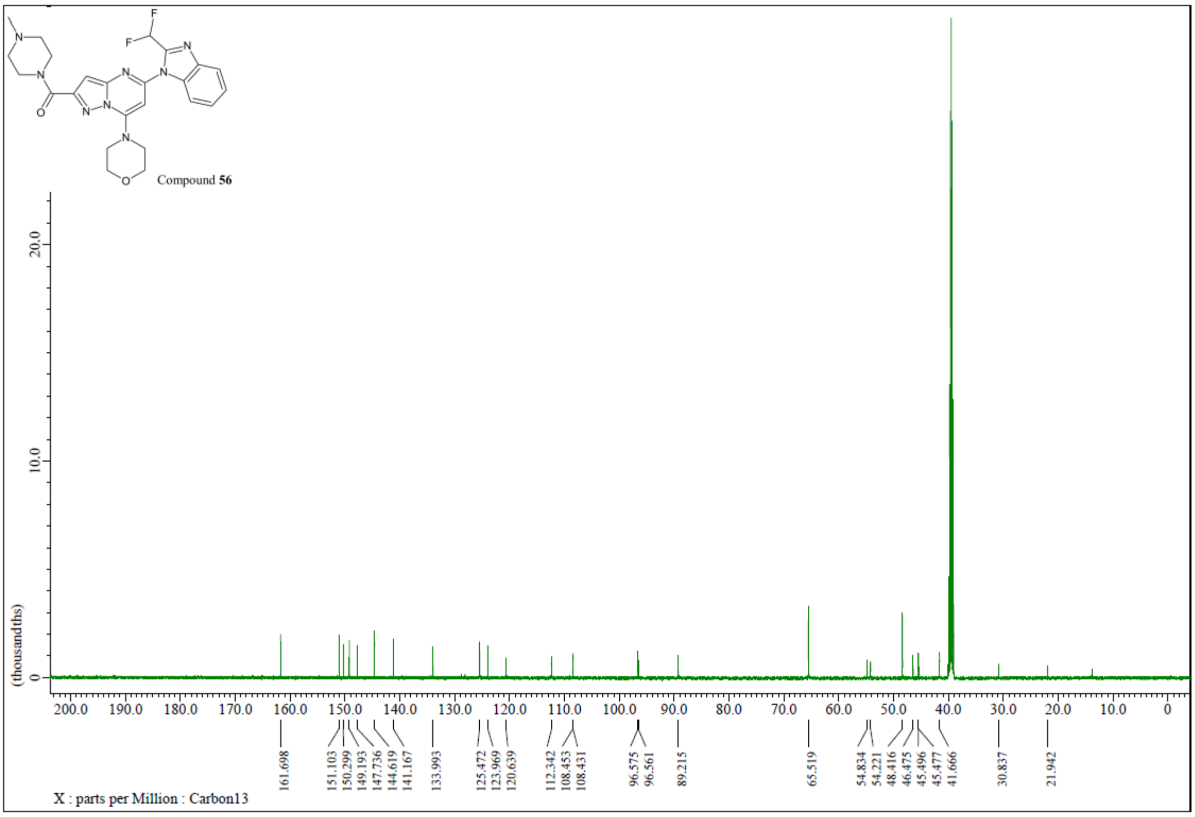

Compound 56

Name	*Obs. m/z*	Obs. RT	Obs. Mass	Tgt Formula	Tgt Mass	Tgt Mass Error	Find Cpds Algorith
Compound **56**	497.22291	1.187	496.21558	C24 H26 F2 N8 O2	496.21468	1.82	Find by Formula

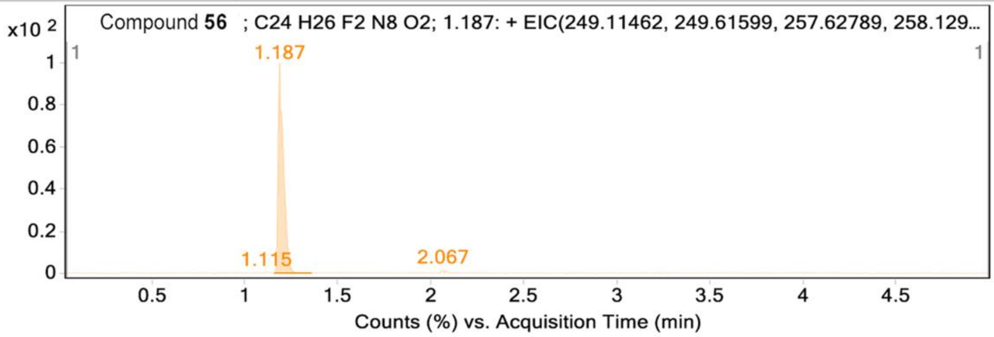

MS Zoomed Spectrum

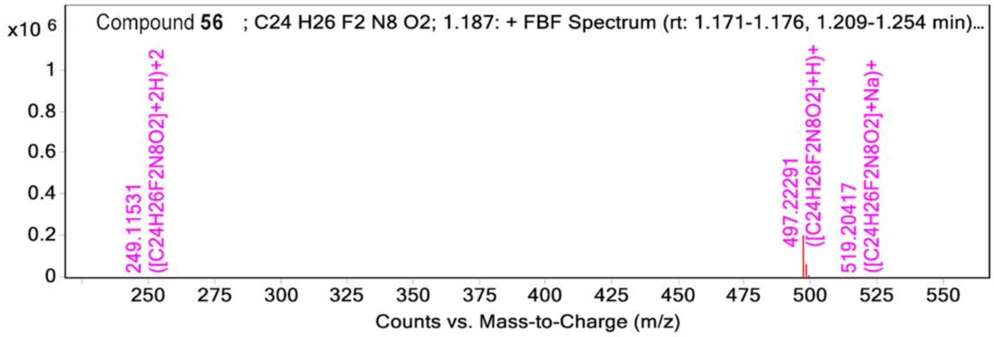

MS Zoomed Spectrum

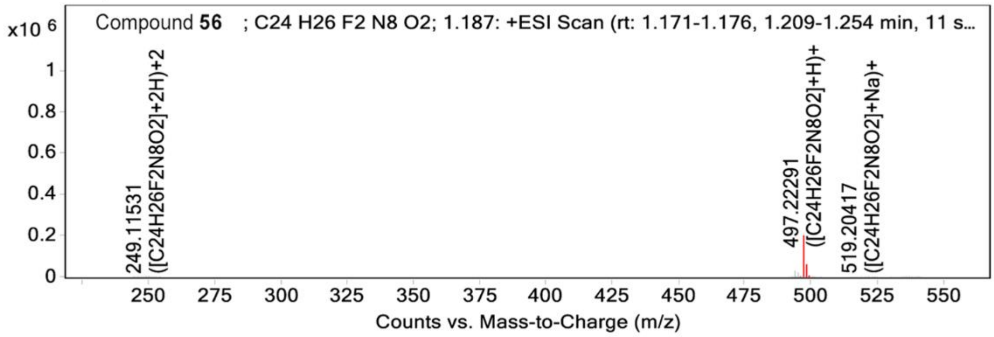

MS Spectrum Peak List

Obs. m/z	Charge	Abund	Formula	Ion/Isotope	Tgt Mass Error (ppm)
249.11531	2	828.16	C24H26F2N8O2	(M+2H)+2	
249.61516	2	252.8	C24H26F2N8O2	(M+2H)+2	
497.22291	1	200608.11	C24H26F2N8O2	(M+H)+	
498.22553	1	53191.16	C24H26F2N8O2	(M+H)+	
499.22792	1	7197.28	C24H26F2N8O2	(M+H)+	
519.20417	1	1904.99	C24H26F2N8O2	(M+Na)+	
520.20623	1	407.84	C24H26F2N8O2	(M+Na)+	
535.17578	1	731.13	C24H26F2N8O2	(M+K)+	
536.18331	1	308.3	C24H26F2N8O2	(M+K)+	
537.17809	1	91.6	C24H26F2N8O2	(M+K)+	
249.11531	2	828.16	C24H26F2N8O2	(M+2H)+2	2.8
249.61516	2	252.8	C24H26F2N8O2	(M+2H)+2	-3.36
497.22291	1	200608.11	C24H26F2N8O2	(M+H)+	1.92
498.22553	1	53191.16	C24H26F2N8O2	(M+H)+	1.63
499.22792	1	7197.28	C24H26F2N8O2	(M+H)+	1.18
519.20417	1	1904.99	C24H26F2N8O2	(M+Na)+	0.53
520.20623	1	407.84	C24H26F2N8O2	(M+Na)+	-0.82
535.17578	1	731.13	C24H26F2N8O2	(M+K)+	-3.85
536.18331	1	308.3	C24H26F2N8O2	(M+K)+	5.08

Compound 57

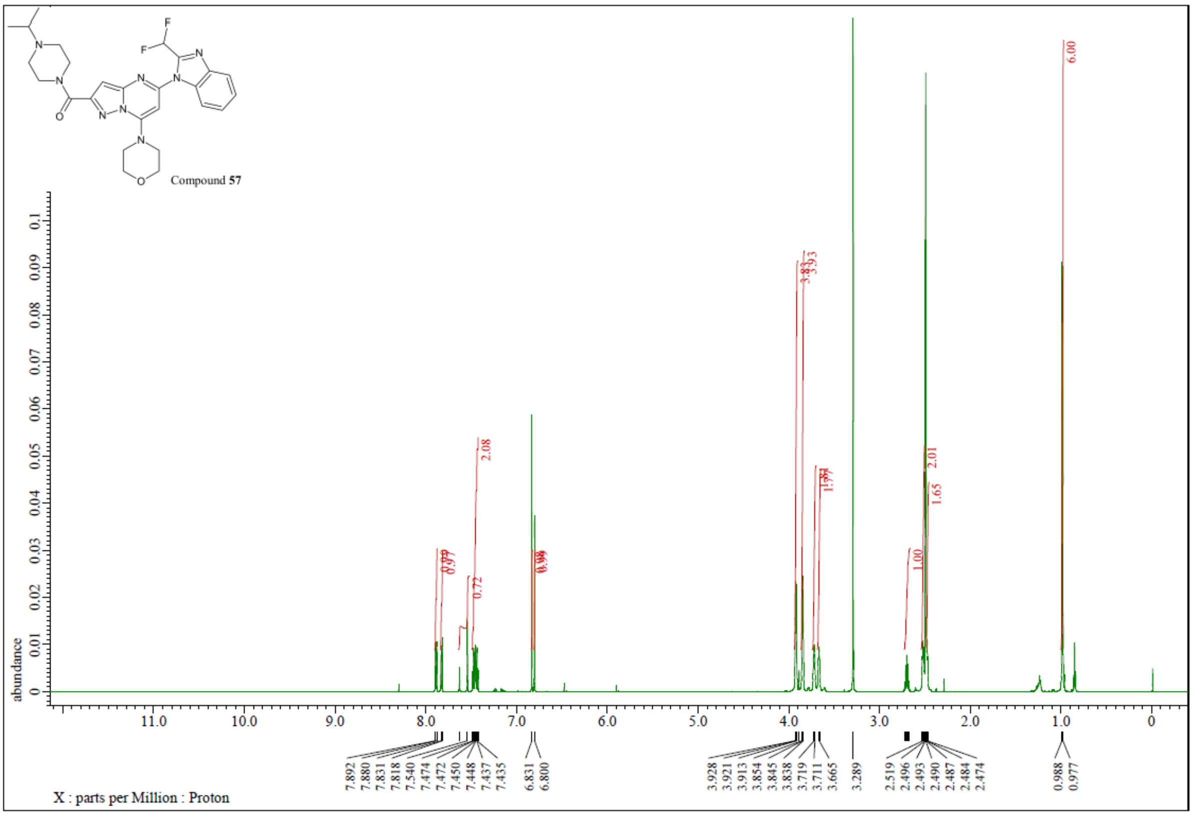

Compound 57
abundance
X : parts per Million : Proton
7.892
7.880
7.831
7.818
7.540
7.474
7.472
7.450
7.448
7.437
7.435
6.831
6.800
3.928
3.921
3.913
3.854
3.845
3.838
3.719
3.711
3.665
3.289
2.519
2.496
2.493
2.490
2.487
2.484
2.474
0.988
0.977
2.08
0.72
1.00
2.01
1.65
6.00

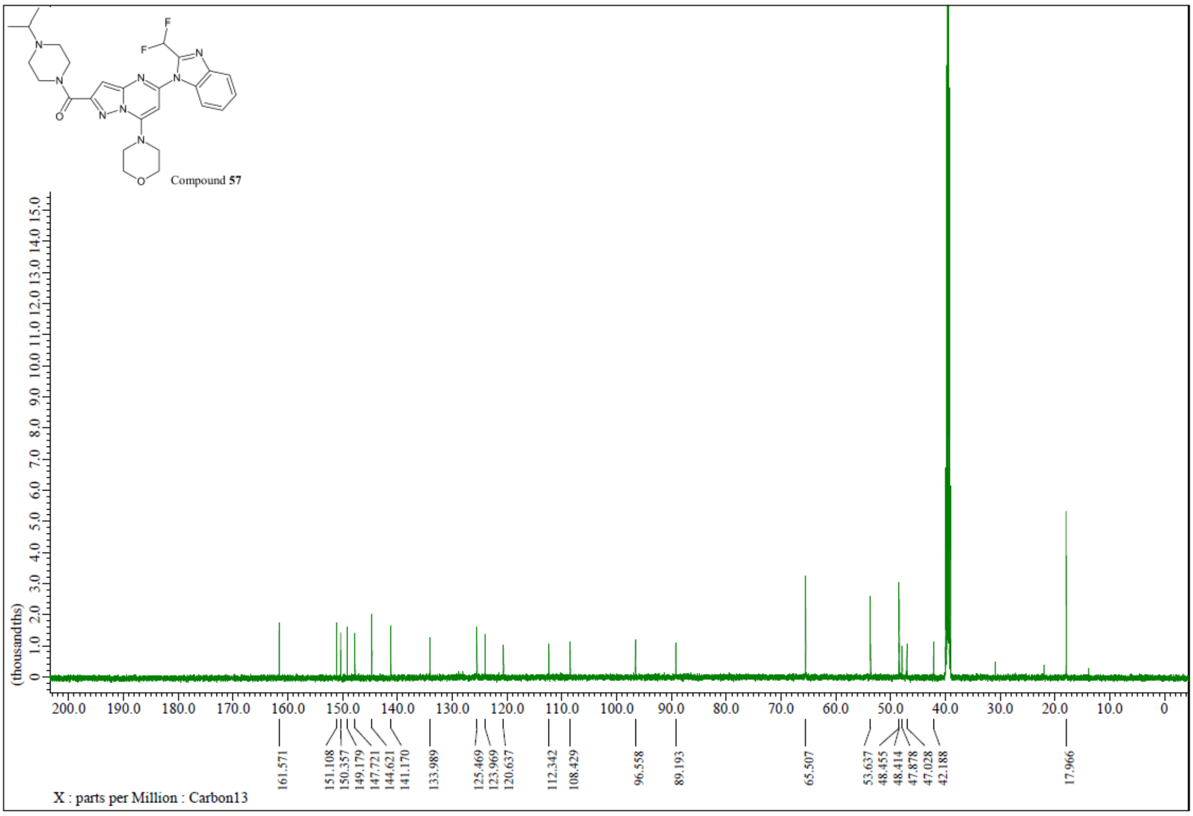

Compound 57
(thousandths)
X : parts per Million : Carbon13
161.571
151.108
150.357
149.179
147.721
144.621
141.170
133.989
125.469
123.969
120.637
112.342
108.429
96.558
89.193
65.507
53.637
48.455
48.414
47.878
47.028
42.188
17.966

Compound 57

Name	*Obs. m/z*	Obs. RT	Obs. Mass	Tgt Formula	Tgt Mass	Tgt Mass Error	Find Cpds Algorit
Compound **57**	525.25441	1.26	524.24697	C26 H30 F2 N8 O2	524.24598	1.88	Find by Formula

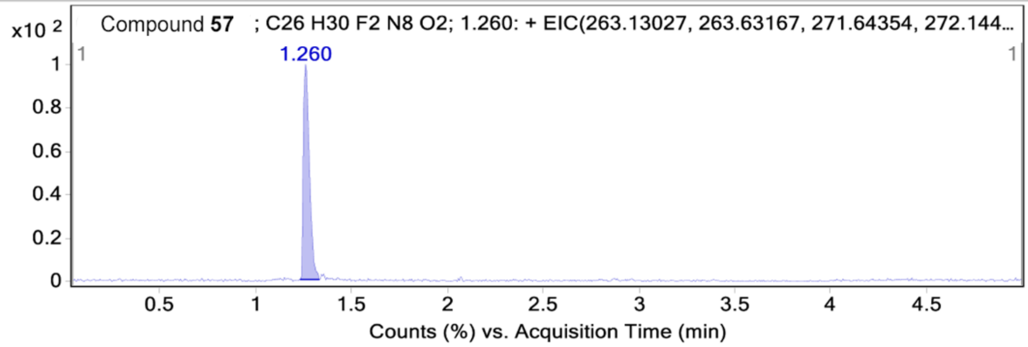

MS Zoomed Spectrum

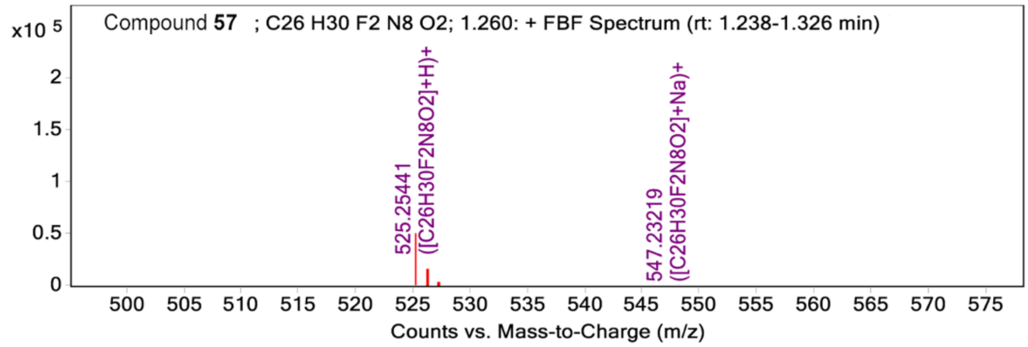

MS Zoomed Spectrum

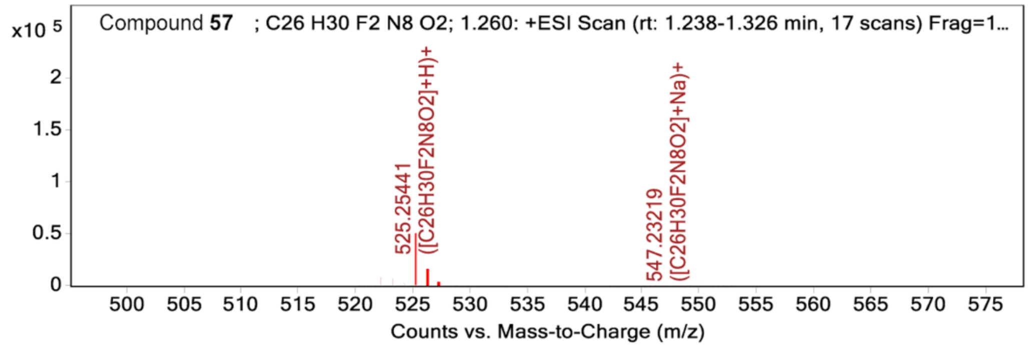

MS Spectrum Peak List

Obs. m/z	Charge	Abund	Formula	Ion/Isotope	Tgt Mass Error (ppm)
525.25441	1	49401.69	C26H30F2N8O2	(M+H)+	
526.2567	1	14426.26	C26H30F2N8O2	(M+H)+	
527.25916	1	2182.63	C26H30F2N8O2	(M+H)+	
528.25892	1	364.03	C26H30F2N8O2	(M+H)+	
547.23219	1	209.02	C26H30F2N8O2	(M+Na)+	
548.2421	1	55.47	C26H30F2N8O2	(M+Na)+	
525.25441	1	49401.69	C26H30F2N8O2	(M+H)+	2.19
526.2567	1	14426.26	C26H30F2N8O2	(M+H)+	1.21
527.25916	1	2182.63	C26H30F2N8O2	(M+H)+	0.8
528.25892	1	364.03	C26H30F2N8O2	(M+H)+	-4.53
547.23219	1	209.02	C26H30F2N8O2	(M+Na)+	-5.5
548.2421	1	55.47	C26H30F2N8O2	(M+Na)+	7.47

--- End Of Report ---
